# Prevalence of active convulsive epilepsy in Dunukofia County in South East Nigeria: a door-to-door survey

**DOI:** 10.1186/s42494-024-00200-4

**Published:** 2025-03-24

**Authors:** Obiora Daniel Anaje, Paul Osemeke Nwani, Maduaburochukwu C. Nwosu, Lasbrey Azuoma Asomugha, Chetanna Chioma Anaje, Ifeoma Adaigwe Amaechi, Uzoamaka Nwakaego Akobundu, Olisaeloka Ginikachi Ebeogu, Linda Ifunanya Eze, Nnamdi Joseph Morah, Sunday Onyemaechi Oriji, Chinwe Chioma Ndukwe, Imelda chinenye Omaga, Adesola Ogunniyi

**Affiliations:** 1 Department of Internal Medicine, Neurology Unit, Nnamdi Azikiwe University Teaching Hospital, P.M.B. 5025, Nnewi, 435101 Anambra State Nigeria; 2 Department of Internal Medicine, Neurology Unit, NnamdiAzikiwe University, P.M.B. 5001, Awka, 420001 Anambra State Nigeria; 3 Department of Medical Rehabilitation, Nnamdi Azikiwe University, P.M.B. 5001, Awka, 420001 Anambra State Nigeria; 4 Department of Neurology, Leeds General Infirmary, Great George Street, LS1 3EX Leeds, England; 5 Neurology Unit, Department of Internal Medicine, Enugu State University of Science and Technology Teaching Hospital, 1030 Parklane Enugu, P.M.B, Enugu State, Nigeria; 6 Musgrove Park Hospital, Somerset NHS Foundation, Stroke UnitPark Drive Way, TA1 5DA Taunton, England; 7 Department of Mental Health, Nnamdi Azikiwe University, P.M.B. 5001, Awka, 420001 Anambra State Nigeria; 8 Department of Internal Medicine, University College Hospital/ University of Ibadan, P.M.B. 5017 G.P.O Ibadan 200285, Oyo State, Nigeria

**Keywords:** Intraregional variation, Prevalence, Active convulsive epilepsy, Comparison, Six towns

## Abstract

**Background:**

Regional variations in the prevalence of epilepsy in Nigeria have been validated. We determined the prevalence of active convulsive epilepsy in six towns of Dunukofia County and compared the findings with existing regional prevalence data.

**Methods:**

Patients with active convulsive epilepsy were identified in a two-phase cross-sectional descriptive community-based door-to-door study using a validated questionnaire in the first phase and a modified epilepsy questionnaire developed for tropical countries in the second phase after clinical assessment and electroencephalogram.

**Results:**

A total of 9000 persons were surveyed in the first stage, of which 56 had active convulsive epilepsy. The highest point prevalence was found in Nawgu, 7.3 per 1000 (95% confidence interval [CI]: 2.7–15.8) while the lowest point prevalence of 5.0 per 1000 (95% CI: 2.0–10.3) was obtained in Ukpo. The observed rates after age adjustment to the Nigeria standard population of 4.9–5.7 per 1000 in this study, which was comparable to 4.6–5.7 per 1000 reported in previous studies, besides two isolated reports of rates as low as 2.7 per 1000 and as high as 20.0 per 1000 reported in the past from two sites in the northern section of the region.

**Conclusions:**

The burden of epilepsy is high in this region, and intra-regional differences in prevalence rates exist. The implications of this finding do not only border on the care of people living with epilepsy but also highlight the need to identify local risk factors as well as appropriate and locally acceptable approaches to reduce the epilepsy burden.

**Supplementary Information:**

The online version contains supplementary material available at 10.1186/s42494-024-00200-4.

## Background

Epilepsy affects approximately 70 million people worldwide, with 90% of the affected population living in developing countries [1]. The prevalence of epilepsy is estimated to be higher in tropical countries, particularly in Africa, with a male preponderance [2]. Early community-based epidemiologic studies in Nigeria were conducted in Southwest Nigeria, reporting prevalence rates of 5.3 per 1000 and 37 per 1000 in Aiyete Ibarapa North local government area (LGA) and Igbo-Ora (Ibarapa Central LGA), respectively, and 6.2 per 1,000 in Udo, a rural Edo-speaking community in South-south Nigeria [3–5]. However, in the last decade, other community-based prevalence studies in Nigeria have emerged from other regions of the country, including the southeastern and northern parts, with prevalence rates ranging from 4.3–20.8 per 1000 people [6–9]. These recent studies have indicated regional variations in the prevalence of epilepsy in the country, as well as the long-known difference in prevalence between rural and urban areas.

A recent community-based study utilizing the same methodology and study protocol reported regional variation in the crude prevalence of epilepsy among the three main regions and people groups in the country: the northern Hausa-speaking people, the western Yoruba-speaking people, and the eastern Igbo-speaking people of Nigeria [10]. The crude prevalence of active epilepsy in the study were 2.3 (95% CI: 1.6–3.5) per 1000 in Ijebu-Jesa, Southwest Nigeria (Yoruba speaking), 2.7 (95% CI: 2.0–3.7) per 1000 in Afikpo Southeast Nigeria (Igbo speaking) and 11.5 (95% CI: 10.0–13.2) per 1000 in Gwandu, Northwest Nigeria (Hausa speaking) [10]. Similarly, Osakwe et al. [7] utilized the same study protocol and reported a crude prevalence of 20.0 (95% CI: 15.7–27.4) in Ochiohu, Southeast Nigeria, an Igbo speaking community and 4.7 (95% CI: 3.2–6.9) in Ogobia, North-central Nigeria [7]. These inter-regional variation may, in part, be explained by the Nigerian multi-ethnic nature as well as the wide socio-cultural and religious diversities of the country. Nigeria is divided into six geopolitical zones based in part on ethnic similarities and/or common political history. The Southeast zone is one of the geopolitical regions in the country and is the home to the Igbo-speaking Nigerians, previously referred to as Eastern Nigeria, and consists of five states: Anambra, Enugu, Ebonyi, Imo, and Abia. Of all these zones, the southeast zone is unique in that it is the only region that has people of one ethnic extraction (the Igbo-speaking people), while the other regions are mixed, although an ethnic group may be more predominant. No recent study has compared the prevalence of epilepsy within the same region of the country using the same research methodology, case definitions, and protocols.

The present study aimed to compare the prevalence of epilepsy among the six towns that constitute Dunukofia County (local government area) in Southeast Nigeria using similar methodology, case definition, and protocol, and to compare the findings with existing prevalence data in Southeast Nigeria. Such data will reveal the presence or absence of intraregional variations in the prevalence of epilepsy and will serve as a prelude to encourage further research on the possible causes of such intraregional differences. Furthermore, information from such studies will guide the formulation of appropriate, culturally acceptable policies that will help reduce the prevalence and burden of epilepsy in the region and the nation in general.

## Methods

### Study location

This was a community-based door-to-door study conducted in the six autonomous communities (towns) that constitute Dunukofia County (local government area) in Anambra State, Southeast Nigeria. Dunukofia County is one of the twenty one local government areas of Anambra state [11]. The six towns that make up the Dunukofia Local government area surveyed were: Ukpo, Ifitedunu, Umudioka, Ukwulu, Nawgu, and Umunachi. These towns are referred to as autonomous communities because they exist as independent village units and are self-governing. Each town has its own laws, even though they are in the same local government area and are Igbo-speaking.

Dunukofia LGA occupies an area of approximately 64 square kilometers and shares boundaries with Awka-North, Idemili-North, Njikoka and Oyi Local Governments. According to the 2006 National Population Census, the population of the Dunukofia LGA is approximately 95,517 [12]. Dunukofia was chosen because the six towns that make up the county are in close proximity to one another, share a common ancestry, and have relatively similar cultural and religious practices. The people of this area are also not new to epidemiological studies of this nature and have consistently demonstrated readiness for such studies. Ukpo, the headquarters of the local government area, is a semi-urban community with two government hospitals, including one of the community medicine outstations of the Nnamdi Azikiwe University Teaching Hospital (NAUTH), and has two government secondary schools, several private schools, and a good road network ensuring accessibility. Similarly, Ifitedunu, Umunachi, and Umudioka, each with at least one government or mission secondary school, a government health center, and some private schools and hospitals, are also considered semi-urban. The remaining two towns, Ukwulu and Nawgu, are rural areas.

### Sample size calculation and study population selection

The sample size was calculated using the formula: N = DZ_1-α/2_^2^ P (1-P)/ d^2^. (D = design effect = 2; Z_1-α/2_ = standard normal deviate corresponding to 5% level of significance (two-sided test) = 1.96; P = prevalence = 4.3 per 1000 [6] = 0.0043; d = absolute precision = 0.00215. For sample sizes lower than 5%, it is recommended that the precision be approximately half the prevalence; hence, a precision of 2.15 per 1000 was used [13, 14]. A minimum sample size of approximately 7115 was obtained. Assuming a response rate of 90% to compensate for attrition, the estimated sample size was 7905.

The Dunukofia LGA has a population of 95,517 according to the 2006 population census, but this population is expected to have increased. To select an adequate representative sample, we estimated the population to be studied by using national data stating that each census enumeration area has an average of 47 households and that the average household size in the country is 5.0 persons per household [15]. Dunukofia has 471 census enumeration areas: Ukwulu (127), Ifitedunu (112), Ukpo (88), Umunachi (55), Umudioka (54), and Nawgu (35). These areas were identified and listed, and using computer-generated random numbers, one out of every cluster of seven (approximately 15%) of the enumeration areas was selected for the survey. The population was estimated to be 15,980. Since persons under 10 years old, who accounted for 30.9% of the Nigerian population, were excluded from the study, the estimated population to be surveyed was 11,043 persons [15]. As the calculated minimum sample size was 7905, everyone aged 10 years and above in the selected enumeration areas who consented was surveyed to accommodate the expected increase in the population and improve the power of the study.

### Inclusion criteria

Everyone aged 10 years and above who was resident in the study communities prior to the study were interviewed in the first phase. Those selected in the first phase of the study who met the definition of active epilepsy stated above were included in the second phase of the study.

### Exclusion criteria

Individuals with acute symptomatic seizures (provoked seizures), single unprovoked seizures or isolated seizures, febrile seizures, neonatal seizures, non-epileptic events (such as disturbances in brain functions, e.g., vertigo or dizziness, syncope), and pseudo-seizures were excluded from cases of active convulsive seizures. Likewise, a cluster of seizures occurring in a 24-h period was excluded if there was no history of a repeat seizure episode. Persons who were less than 10 years old were excluded from the study because the characteristics of epilepsy required the participant’s ability to make assessments and recall information, such as health-related quality of life and epilepsy complications, which were part of the study protocol. It further reduced some inherent challenges associated with diagnosing seizures disorders in younger children, as they may be unable to describe subjective features that might be associated with or precede the actual convulsive events. However, excluding this age group is a limitation of this study, as it removes a population with a high prevalence of epilepsy, thereby limiting the direct comparison of the findings with those of other studies that included the age group. People living with epilepsy (PLWE) who were not residents of the villages and had only visited during the study period were also excluded from the study.

### The survey protocol

#### Community engagement, enumeration area demarcation and training of research assistants

This was a two-phase cross-sectional descriptive study. The study was preceded by community engagement activities that involved visiting traditional rulers (“Igwes”) and opinion leaders in the six towns by the research team. Research networks were established in each town, comprising volunteer church leaders, village group leaders, and town criers (those who makes public announcement in the village and market square). They were involved in the sensitization of the people, including church members and age grade members, and dissemination of information about the purpose and procedures of the study in the individual community.

One month prior to the commencement of the study, an initial identification and demarcation of the enumeration areas to be studied in different communities. These enumeration areas were appropriately identified with the aid of the National Population Commission staff. Training workshops were conducted for research assistants in different towns to educate them on the procedures and use of the study instruments.

#### First phase of the study

The study was conducted between 6th May and 20th September in 2019. The first phase of the study was from 6th May to 27th July in 2019. This phase was a door-to-door survey, during which every member of the selected households above the age of 10 years was interviewed using a validated questionnaire adapted from the modified World Health Organization questionnaire for detecting neurological diseases [16]. The aim of the first stage was to identify patients with possible active convulsive epilepsy. The adapted questionnaire was translated into the Igbo language and back-translated into English by two bilingual Igbo West African Examination Council examiners. Furthermore, the translated version was reviewed by an expert panel consisting of the translators, researchers versed in Igbo language, and some laypersons to assess the suitability of the instrument within the Igbo context and culture, and a number of changes were made to come up with the harmonized Igbo version. The harmonized Igbo version was evaluated before the actual study, and local validation of the instrument at the Neurology Clinic of Nnamdi Azikiwe University Teaching Hospital (NAUTH) yielded a sensitivity of 100% and specificity of 65%.

During the first stage of the study, if the research team did not meet the household occupants during the first visit, a repeat visit was arranged.

#### Second phase of the study

In the second stage of the study, all those identified as possibly having active convulsive epilepsy during the first stage of the study were interviewed by a team of research neurologists and senior residents in neurology. This second stage was held at the community health center in each town. A semi-structured epilepsy specific questionnaire, which was a modification of an epilepsy questionnaire developed for tropical countries, was used for interviews in the second stage of the study, with the aim of diagnosing of active convulsive epilepsy [17]. In addition to information on other essential socio-demographic data, the clinical profile of epilepsy such as age at the onset of seizure, seizure frequency, seizure duration, health-related quality of life, and seizure-related complications were also obtained. Active epilepsy was diagnosed after a detailed clinical history and examinations were conducted independently by two neurologists, who then reached a consensus diagnosis. Electroencephalographic (EEG) recordings were performed for those diagnosed with active convulsive epilepsy by a trained technician. A mobile EEG machine was used, with an average EEG study period of 45 min that included both hyperventilation and photic stimulations. The technician provided the technical report, while the clinical report was performed by the research neurologists.

#### Definition of active epilepsy

For the purpose of this study, active epilepsy was defined as the occurrence of two or more unprovoked seizures on different days in the previous year or currently on anti-seizure medication [18–21]. The definition used in this study has more recently been proposed to inform treatment better than the definition of two or more unprovoked seizures in the prior five years, as proposed by the International League Against Epilepsy (ILAE) Epidemiology Commission and may help reduce recall bias [10, 22, 23].

### Statistical analysis

Data were analyzed using the Statistical Package for the Social Sciences (SPSS™) version 22.0 (SPSS Inc. software, Illinois, USA). Relevant percentages, frequencies, means, and standard deviations were calculated. Prevalence values with 95% confidence intervals (95% CI) were calculated. The prevalence was age-adjusted to the 2006 standard population of Anambra and Nigeria using the direct method. The Nigerian standard population was used to enable direct comparisons with prevalence rates from previous studies, as age-adjusted rates being compared must all be based on the same standard population [24].

## Results

**Fig. 1:**
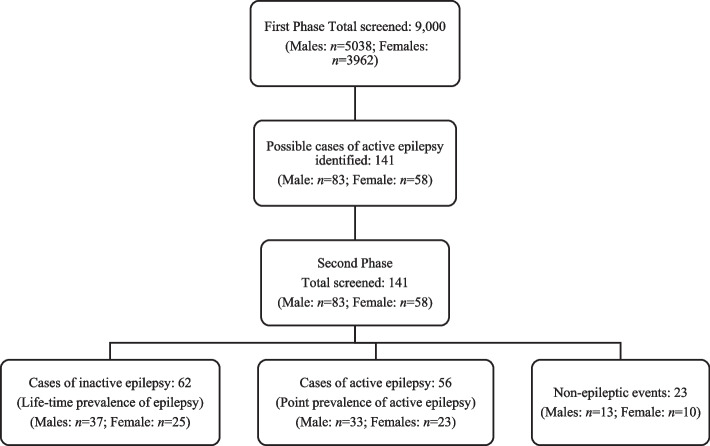
Flow chart showing patient recruitment and the survey procedure

A total of 9000 people, comprising 5038 males (56.0%) and 3962 females (44.0%), were surveyed in the first phase of the study, with a response rate of about 81.5% of the estimated population (see *supplementary material 1: demographics of population screened in the first phase of the study and outcome of second phase screening and supplementary material 2: age and sex distribution of the study population*). One hundred and forty-one persons were identified at the first phase as possibly having active convulsive epilepsy and were all interviewed in the second phase, with a response rate of 100%. In the second phase, 62 patients were diagnosed with lifetime epilepsy, whereas 56 were diagnosed with active convulsive epilepsy. Figure 1 is the flow chart showing patient recruitment and the survey procedure.

The prevalence of active convulsive epilepsy in the communities ranged from 5.0 per 1000 (95% CI: 2.0–10.3) recorded in Ukpo to 7.3 per 1000 (95% CI: 2.7–15.8) recorded in Nawgu, with a percentage difference of 37.4% (Table 1). The pooled prevalence of active convulsive epilepsy from the six towns of Dunukofia LGA was 6.2 per 1000 (95% CI: 4.7–8.1). When the prevalence rates were further age-adjusted to the Nigerian 2006 standard population, the observed prevalence ranged from 4.9 per 1000 (95% CI:1.5–8.3) in Umunachi to 5.7 per 1000 (95% CI:1.2–10.2) in Nawgu (Table 1).

**Table 1:** The prevalence of active convulsive epilepsy in the towns studied and the pooled prevalence in Dunukofia local government area

**Community**	**Population screened** (*n*)	**Lifetime Prevalence (%)** **[*n*]**^**a**^** (95% CI)**	**Point Prevalence (%) [*n*]**^**a**^** (95% CI)**	Age adjusted Prevalence AS* (%) **(95% CI)**	Age adjusted Prevalence NG** (%) (95% CI)
Nawgu	821	8.5 [7] ( 3.4–17.5)	7.3 [6] (2.7–15.8)	6.0 (1.3–10.7)	5.7 (1.2–10.2)
Umudioka	1030	7.8 [8] ( 3.4–15.3)	6.8 [7] (2.7 – 14.0)	5.7 (1.6–9.8)	5.2 (1.3–9.1)
Umunachi	1198	7.5 [9] (3.4–14.2)	6.7 [8] (2.9–13.1)	5.2 (1.6–8.8)	4.9 (1.5–8.3)
Ifitedunu	1518	7.2 [11] (3.6–12.9)	6.6 [10] (3.2–12.1)	5.5 (2.1–8.9)	5.0 (1.9–8.1)
Ukwulu	3033	6.6 [20] (4.0–10.2)	5.9 [18] (3.5 – 9.4)	5.8 (3.3–8.3)	5.0 (2.5–7.5)
Ukpo	1400	5.0 [7] ( 2.0–10.3)	5.0 [7] (2.0–10.3)	5. 5 (1.3–9.7)	5.2 (1.3–9.1)
DLGA	9000	6.9 [62] (5.3–8.8)	6.2 [56] (4.6—8.1)	6.2 (4.6–7.8)	5.5 (4.0–7.0)

^a^Actual number of patients is shown in parentheses. *CI* = Confidence interval, AS*Anambra State 2006 Population, NG** Nigeria 2006 Population, DLGA-Dunukofia local government area

Table 2 shows the comparison of prevalence rates obtained in the present study with existing regional prevalence rates obtained from community-based studies and age-adjusted to the Nigerian standard population 2006. The point prevalence in this study ranged from 5.0 per 1000 (95% CI: 2.0–10.3) in Ukpo to 7.3 per 1000 (95% CI: 2.7–15.8) in Nawgu, while existing point prevalence rates range from 2.7 per 1000 (95% CI: 2.0–3.7) in Afikpo to 20.8 per 1000 (95% CI: 15.7–27.4) in Ochiohu. The age-adjusted prevalence rates obtained in the present study ranged from 4.9 per 1000 (95% CI: 1.5–8.3) in Umunachi to 5.7 per 1000 (95% CI: 1.2–10.2) in Nawgu, while the age-adjusted prevalence in previous regional existing studies ranged from 2.5 per 1000 (95% CI: 1.8–3.5) in Afikpo to 5.4 per 1000 (95% CI: 3.4–7.4) in Enugu.

**Table 2:** Comparison of prevalence rates obtained in this study with existing regional prevalence rates obtained from community-based studies

Study	Year of survey	Location [U]/[SU]/[R]^a^	Age range studied	Population surveyed [*n*]^b^	Point prevalence of epilepsy (95% CI)	Age adjusted prevalence (%)^c^ (95% CI)
Nwani et al [6]	2010	Ukpo [SU]	All	6800 [29]	4.3 (2.7-5.9)	4.6 (2.9 - 6.3)
Ezeala-Adikaibe et al [8]	2013	Enugu [U]	≥15years	8228 [49]	6.0 (5.9–6.0)	5.4 (3.4–7.4)
^d^Osakwe et al [7]	2007	Ochiohu [R]	>6 years	2500 [52]	20.8 (15.7—27.4)	NA^e^
^d^Watila et al [10]	2018	Afikpo[SU]	≥6 years	15,738 [43]	2.7 (2.0–3.7)	2.5 (1.8–3.5)
Present study	2019	Nawgu [R]	≥10 years	821 [6]	7.3 (2.7-15.8)	5.7 (1.2-10.2)
Present study	2019	Ukwulu [R]	≥10 years	3033 [18]	5.9 (3.5 – 9.4)	5.0 (2.5-7.5)
Present study	2019	Umunachi [SU]	≥10 years	1198 [8]	6.7 (2.9-13.1)	4.9 (1.5-8.3 )
Present study	2019	Ifitedunu [SU]	≥10 years	1518 [10]	6.5 (3.2 –12.1)	5.0 (1.9-8.1)
Present study	2019	Umudioka [SU]	≥10 years	1030 [7]	6.8 (2.7 – 14.0)	5.2 (1.3-9.1)
Present study	2019	Ukpo [SU]	≥10 years	1400 [7]	5.0 (2.0-10.3)	5.2 (1.3-9.1)

^a^[U] urban, [SU] semi-urban, [R] rural

^b^Actual number of cases in parentheses

^c^Age adjusted prevalence to Nigeria standard population 2006

^d^Regional prevalence extracted from combined studies involving other regions of the country

^e^Not available and insufficient data online to adjust

The sex differences in the prevalence of active convulsive epilepsy in the county and the six towns that make up the county (LGA) are shown in Table 3. In five towns, there was a higher male sex-specific prevalence ranging from 5.0 per 1000 (95% CI: 1.4–12.7) to 9.1 per 1000 (95% CI: 3.0–21.0). The female sex-specific prevalence ranged from to 4.2 per 1000 (95% CI: 0.5–15.0) to 7.0 per 1000 (95% CI: 3.4–12.9). However, this observed difference was not statistically significant (*P* > 0.05; Fishers’s exact test = 0.786).

**Table 3:** Sex-specific prevalence in Dunukofia local government area and the six towns

Community	Male		Female		Total	
	Population screened (*n*)	Prevalence **(%)** [*n*]^a^ 95% CI	Population screened (*n*)	Prevalence **(%)** [n]^a^ 95% CI	Population screened (*n*)	Prevalence **(%)** [n]^a^ 95% CI
**Umunachi**	662	7.6 [5] 2.5–17.5	536	5.6 [3] 1.2–16.2	1198	6.7 [8] 2.9–13.1
**Umudioka**	551	9.1 [5] 3.0–21.0	479	4.2 [2] 0.5–15.0	1030	6.8 [7] 2.7 – 14.0
**Ifitedunu**	965	7.3 [7] 2.9–14.9	553	5.4 [3] 1.1–15.7	1518	6.5 [10] 3.2 –12.1
**Ukwulu**	1902	5.6 [8] 2.2–9.8	1431	7.0 [10] 3.4–12.9	3033	5.9 [18] 3.5 – 9.4
**Ukpo**	806	5.0 [4] 1.4–12.7	594	5.1 [3] 1.0–14.7	1400	5.0 [7] 2.0–10.3
**Nwagu**	452	8.8 [4] 2.4–22.4	369	5.4 [2] 0.7–19.4	821	7.3 [6] 2.7–15.8
**DLGA**	5038	6.6 [33] 4.5–9.2	3962	5.8 [23] 3.7–8.7	9000	6.2 [56] 4.6 – 8.1

*DLGA* Dunukofia local government area; *n*, Actual number of cases; Fisher’s exact test = 0.786

The age-specific prevalence of active epilepsy in the LGA and the six towns is shown in Table 4. Peak age-specific prevalence rates of 11.2 per 1000 to 21.7 per 1000 were observed in the age group of 20–29 in three towns, while peak age-specific prevalence rates of 13.7 per 1000 to 15.5 per 1000 were observed in the age group of 30–39 in the remaining three towns.

**Table 4:** Age specific prevalence in the six towns of Dunukofia LGA and the LGA

**Age range**	**Umunachi** [*n*] (prevalence per 1000, %)	**Umudioka** [*n*] (prevalence per 1000, %)	**Ifitedunu** [*n*] (prevalence per 1000, %)	**Ukwulu** [*n*] (prevalence per 1000, %)	**Ukpo** [*n*] (prevalence per 1000, %)	**Nawgu** [*n*] (prevalence per 1000, %)	**DLGA** [n] (prevalence per 1000, %)
10–19	257 [2] (7.8)	193 [1] (5.2)	228 [3] (13.2)	710 [3] (4.2)	183 [1] (5.5)	140 [1] (7.1)	1711 [11] (6.4)
20–29	178 [2] (11.2)	136 [1] (7.4)	144 [1] (6.9)	453 [6] (13.2)	138 [3] (21.7)	109 [1] (9.1)	1158 [14](12.1)
30–39	181 [0] (0.0)	145 [2] (13.8)	258 [4] (15.5)	461 [5] (10.8)	185 [1] (5.4)	146 [2] (13.7)	1376 [14](10.2)
40–49	206 [2] (9.7)	164 [2] (12.2)	245 [1] (4.1)	429 [3] (7.0)	228 [1] (4.4)	138 [1] (7.2)	1410 [10] (7.1)
50–59	135 [1] (7.4)	163 [1] (6.1)	216 [-]	366 [1] (2.7)	255 [-]	104 [-]	1239 [3] (1.6)
60–69	147 [1] (6.8)	138 [-]	248 [1] (4.0)	348 [-]	222 [1] (4.5)	100 [1] (10.0)	1203 [4] (3.3)
≥ 70	94 [-]	91 [-]	179 [-]	266 [-]	189 [-]	84 [-]	903 [-]
Total	1198 [8] (6.7)	1030 [7] (6.8)	1518[10] (6.6)	3033 [18](5.9)	1400 [7] (5.0)	821[6] (7.3)	9000 [56] (6.2)

*DLGA* Dunukofia local government area; *n*, Actual number of cases

The classification of the epileptic seizures is shown in Table 5. Seizure types identifiable on clinical assessment included only generalized onset seizures (64.2%, *n* = 36) and focal onset seizures (35.7%, *n* = 20). However, using electro-clinical criteria, 44.2% (*n* = 23) and 40.4% (*n* = 21) of the patients had generalized onset seizure and focal onset seizure, respectively. Over 90% of the patients had EEG (*n* = 52); however, the interictal EEG recordings were normal in 15.4% (*n* = 8), and a repeat EEG could not be obtained. Those with normal interictal EEG and those who did not have an EEG record were excluded from the classification based on the electro-clinical criteria.

**Table 5:** Seizure classification in people with active convulsive epilepsy

Type of seizure	Clinical classification (based on clinical assessment only) (%)	Electro-clinical classification (based on inter-ictal EEG recording and clinical assessment) (%)
Generalised onset seizures Tonic–clonic Absence	*n* = 36 34 (60.7%) 2 (3.6%)	*n* = 23 21 (40.4%) 2 (3.8%)
Focal onset Focal onset aware Focal onset impaired awareness Focal to bilateral tonic–clonic	*n* = 20 11 (19.6%) 6 (10.7%) 3 (5.4%)	*n* = 21 10 (19.2%) 6 (11.5%) 5 (9.6%)
Unclassified	-	8 (15.4%)^a^
Total	56 (100%)	52 (100)^b^

^a^Normal interictal EEG recording

^b^Four persons did not have EEG recordings

Table 6 shows the profile of active convulsive epilepsy in this LGA. The male-to-female ratio for those with active convulsive epilepsy was 1.4:1, with a mean age of 32.9 ± 14.2 years and an age range of 11–65 years. Seizures started in the first and second decades of life in about 73.2% (*n* = 41) of cases, while seizure onset occured in the sixth decade in 3.6% (*n* = 2) of cases.

**Table 6:** The profile of people with active convulsive epilepsy

Characteristics	Number	Percentage (%)
**Age groups** 10–19 20–29 30–39 40–49 ≥ 50 **Total**	11 14 14 10 7 **56**	19.6 25.0 25.0 17.9 12.5 **100**
**Age range**	11–65 years	
**Mean age**	32.9 ± 14.2	
**Gender** Male Female **Total**	33 23 **56**	58.9 41.1 **100**
**Age at onset (years)**		
0–9	21	37.5
10–19	20	35.7
20–29	8	14.3
30–39	3	5.3
40–49	2	3.6
≥ 50	2	3.6
**Total**	**56**	**100**
**Frequency of seizures in the last 12 months**		
1–3	23	41.1
4–6	21	37.5
> 6	12	21.4
**Total**	**56**	**100**

## Discussion

Variation in the prevalence of epilepsy between developed and developing nations has long been documented [25, 26]. This is the same within the Nigerian context, where regional variations in the prevalence of epilepsy have been demonstrated in recent community-based studies [3, 4, 7, 9]. However, data on the variation in the prevalence of epilepsy within the same region or locality in the same country remains sparse. Using the same screening tool and standard protocol, we compared the prevalence of epilepsy in the six towns that constitute Dunukofia LGA in Southeast Nigeria.

The point prevalence of 7.3 per 1000 found in Nawgu, a rural community, was 37.4% higher than the prevalence of 5.0 per 1000 found in Ukpo, a semi-urban community. Prevalence rates have been reported to be higher in rural areas than in urban areas due to improved healthcare services, especially adequate maternal and childcare services, including better perinatal care and good immunization coverage for children that exit in urban areas. This difference was also observed between Ukpo (5.0 per 1000) and Ukwulu (5.9 per 1000), another rural community; though not as marked, this suggested the possibility of other local factors influencing the prevalence rates in these areas, either by reducing or increasing the chances of developing epilepsy. The prevalence rate was higher in other semi-urban towns of Ifitedunu and Umudioka than in Ukpo, thereby raising further concern about local factors accounting for the observed difference in prevalence, especially the lower prevalence rate at Ukpo. However, the contributory effect of the population structures of these areas on the observed prevalence is worth considering since the observed differences became marginal when the prevalence rates were age-adjusted to the Nigerian standard population for equitable comparisons. A genetic contribution to the higher prevalence in Nawgu is a possibility worth considering, since the natives of the other five towns are offerings of same sibling according to history, however, this will require further studies to ascertain. Further studies will also focus on the presence of prevailing etiological and risk factors for epilepsy in these communities, as well as the contributory effects of existing cultural beliefs and practices that can influence the observed prevalence of epilepsy, such as concealment of the disease and people’s concept of epilepsy. Knowledge of contributory risk factors will inform locally and culturally acceptable strategies to reduce the burden of epilepsy in this region and assist in the development of models that can be applied to other parts of the country.

Compared to existing prevalence rates in this region of the country, the findings in the present study did not differ much from the others when the prevalence was age-adjusted to the Nigerian standard population. However, the results of the present study and other existing regional data differed significantly from the two previously obtained prevalence rates in Afikpo and Ochiohu, both in the Ebonyi state, which is located in the northern part of the southeast region of the country. Local factors, such as poor obstetric care and endemicity of onchocerciasis, accounting for the high prevalence of epilepsy, were suggested in Ochiohu, a very poor rural community with a population of about 2500 people with no public or private health facility. However, no reason was given for the very low prevalence found in Afikpo [7, 10]. Therefore, local differences in the prevalence rates of epilepsy in different places in this region of the country are probably driven by local risk factors as well as methodological differences, such as variations in the age groups studied and difference in the definition of active epilepsy among the various studies.

There is need for more local epidemiological studies, as well as the establishment of a local community-based epilepsy registry to obtain adequate regional and national statistics on the epidemiology of epilepsy. This can be driven by organizations such as the Nigeria League against Epilepsy and other sister organizations with interest in epilepsy, or academic institutions in the country with interest in neuroepidemiology. The findings of the present study also highlight the limitations of regional or national epilepsy prevalence rates obtained by averaging the results from two or more isolated places within the same or different regions of the country. Studies aimed at determining regional or national prevalence data should therefore be larger-scale studies, utilizing the same study protocol and methodology, and involving multiple sites to obtain more robust data.

The prevalence of epilepsy among the different subsets of the population, as revealed by the age-specific prevalence found in this study, showed that epilepsy is still a disease affecting younger age groups in all the communities. The observed peak prevalence rates were in the second and third decades of life. There was no significant difference in the peak prevalence between semi-urban and rural communities. The peak prevalence in the second decade of life is consistent with the most previously reported peak prevalence in Nigeria. This has been attributed to factors such as the population distributions of countries with younger people and the prevailing risk factors for epilepsy in the country, such as inadequate antenatal and prenatal care, widespread malnutrition, genetic factors, untreated or insufficiently treated infections, and cerebral infections that put the younger age groups at risk [4, 6, 7]. This was evidenced in the present study, with the onset of seizures starting in the first and second decade of life in 73% (*n* = 41) of those with active convulsive seizures.

In the three communities of Umudioka, Ifitedunu, and Nawgu, the peak prevalence was observed in the third decade of life. The peak prevalence in the third decade has also been reported in fewer community studies in Nigeria [8, 9]. The peak age-specific prevalence in the entire Dunukofia local government area of 12.1 per 1000 was seen in the 20–29 years age group and declined after the fifth decade of life, a pattern that has been reported in developing nations [9, 26]. The second peak prevalence of epilepsy reported in developed nations was reported in two previous studies in Southeast Nigerian but was found only at one site in the present study. In Nawgu, a second peak prevalence of 10.1 per 1000 among those aged 60–69 years was found in this study; however, the sample size was too small to make such a categorical statement [8, 10].

In the present study, a higher, albeit statistically insignificant, male prevalence of active epilepsy was found in five towns in the local government area. This is in keeping with the largely reported slightly higher male preponderance of epilepsy compared to females [4, 6, 25, 27–29]. This higher male prevalence has been attributed to the contributions of gender-dependent risk factors, such as excess alcohol use, illicit drug abuse, and engagement in high-risk activities in males and the concealment of the condition in women for sociocultural reasons in certain regions [27, 28, 30]. However, a previous study on the prevalence of active epilepsy in Southeast Nigeria, along with some fewer studies, has reported a higher prevalence in females [8]. This pattern was observed in the Ukwulu community in the present study. The reason for this finding in the current study could not be ascertained, but suggested reasons for the higher female prevalence in other studies included a higher female health-seeking behavior and possibly increased male mortality in the studied communities [4, 8]. Furthermore, studies in this area are advocated because of the relevance of sex to epilepsy care and delivery. Females are more prone to stigmatization and isolation associated with epilepsy, and their sex also has an impact on treatment options available, given the side effect profile of anti-seizure medications in younger adult women and women of childbearing age [31].

Generalized (bilateral onset) seizures were the most prevalent seizure type found in the present study based on electro-clinical classification, accounting for 44% (*n* = 23/52) of cases of active convulsive epilepsy. This finding is at variance with that of a previous study that reported a higher prevalence of focal onset seizures using electro-clinical criteria [6]. However, other studies have reported a similar higher prevalence of bilateral onset seizures, as found in the present study [8, 28]. The seizure type(s) present in a patient with epilepsy is relevant as a determinant of the choice of anti-seizure medication needed to treat the patients and are also relevant in epilepsy nosology.

The age at seizure onset in the present study demonstrated a similar pattern to that reported in previous community-based studies in the country. The age at onset of seizure can serve as a guide to the possible etiological or risk factors for epilepsy, as well as predict the potential impact of epileptic seizures on the development and productivity of the sufferer. In this present study, the aged at onset was in the first and second decades of life for the majority of patients (73%, *n* = 41) with active convulsive epilepsy. The peak age at seizure onset was the age group 0–9 years, accounting for 37.5% (*n* = 21) of the cases. There was a marked decline in age of onset after the second decade of life.

Seizure severity is determined by factors such as the duration of each seizure episode, associated loss of consciousness, seizure frequency, and complications or injuries. In this index study, more than half 58.9% (*n* = 33) of the patients with epilepsy had four times or more seizures in the year preceding the study. A high seizure frequency predisposes PLWE to a higher rate of physical injury, psychological trauma, low quality of life, and sudden unexpected death [32–34].

However, there are limitations to this study despite its benefits. The prevalence estimates obtained may have been underestimated due to the exclusion of children aged < 10 years. Though collecting epidemiological data in children is challenging for reasons, like varied presentation of seizures, and communication barriers among young children, their exclusion from studies on the other hands also contributes to the underestimation of cases. Other limitations that can lead to underestimation of the prevalence of epilepsy include the likelihood of non-disclosure or concealment due to the stigmatization associated with epilepsy, as well as the perception of epilepsy as a spiritual attack rather than illness among some Igbo people. However, this would have been reduced by the rigorous nature of the protocol and the extent of community engagement in the study. Our inability to survey the entire community is another limitation, although the study design and calculation of sample size would have reduced this issue. However, surveying the entire population would provide better and more robust results.

## Conclusions

The burden of epilepsy is high in this region, and intra-regional differences in prevalence rates exist. The implications of this finding do not only pertain to the care of people with epilepsy but also highlight the need to identify local risk factors and develop appropriate, locally acceptable approaches to reduce the burden of epilepsy.

## Supplementary Information

Supplementary Material 1.

Supplementary Material 2.

## Data Availability

All data relevant to the study are included in the article or uploaded as supplementary information.

## Abbreviations

EEG: Electroencephalogram
ILAE: International league against epilepsy
LGA: Local government area
NAUTH: Nnamdi Azikiwe University Teaching Hospital
PLWE: People living with epilepsy
