# Supplementary material for: Prevalence of active convulsive epilepsy in Dunukofia County in South East Nigeria: a door-to-door survey
Source: Acta Epileptol. 2025 Mar 24;7:21. doi: 10.1186/s42494-024-00200-4 (PMC11960363; doi:10.1186/s42494-024-00200-4)
Supplement: Supplementary file 1 — Supplementary Material 1. [file 42494_2024_200_MOESM1_ESM.pdf]

Demographic data of the population screened at the first phase and the outcome of the second phase screening

S/No	Age	Age range	Sex	Occupation	Religion	Marital status	HLED	Residence	Shaking (8)/ Fall (9)	Outcome
1	57	10	2	1	1	4	2	3	2	0
2	39	6	1	3	1	2	2	3	1	1
3	54	9	1	1	1	1	2	3	2	0
4	16	9	1	7	1	2	3	3	1	1
5	72	13	1	1	1	1	2	3	2	0
6	18	2	2	7	1	2	2	3	1	1
7	72	13	1	3	1	1	2	6	2	0
8	30	5	1	3	1	2	2	3	1	1
9	66	12	1	4	1	1	3	3	2	0
10	29	4	2	7	1	2	3	3	1	1
11	40	7	1	3	1	1	3	3	2	0
12	30	9	2	4	1	5	4	3	1	1
13	71	13	1	2	1	1	2	3	2	0
14	38	6	1	1	1	2	3	3	1	1
15	58	10	1	2	1	1	3	3	2	0
16	15	2	1	7	1	2	3	3	2	0
17	67	12	2	6	1	4	4	3	2	0
18	45	11	1	2	1	3	3	3	1	1
19	18	2	1	3	1	2	2	3	1	1
20	40	7	1	2	1	1	4	3	2	0
21	30	5	2	4	1	1	3	3	1	0
22	37	6	1	2	1	1	3	3	2	0
23	34	6	2	5	1	2	2	3	2	0
24	91	15	1	6	1	1	2	3	2	0
25	27	4	2	5	1	2	1	3	2	0
26	50	9	2	3	1	4	3	3	2	0

27	35	6	1	3	1	2	1	3	1	2
28	65	12	2	1	1	4	2	3	2	0
29	29	4	1	3	1	2	3	3	2	0
30	68	12	2	1	1	4	2	5	2	0
31	18	2	2	7	1	2	3	5	2	0
32	12	1	1	7	1	2	2	5	2	0
33	32	5	2	4	1	1	3	5	2	0
34	37	6	1	1	1	2	2	5	2	0
35	58	10	1	4	1	1	2	5	2	0
36	43	7	2	2	1	2	2	5	2	0
37	40	7	2	2	1	1	2	5	2	0
38	74	13	2	1	1	4	1	5	2	0
39	41	7	2	2	1	4	3	5	2	0
40	13	1	1	5	1	2	1	5	2	0
41	13	1	2	7	1	2	3	5	2	0
42	16	2	2	5	1	2	1	5	2	0
43	12	1	1	7	1	2	3	5	2	0
44	46	8	1	2	1	1	3	1	2	0
45	14	1	1	7	1	2	3	4	1	1
46	46	8	1	3	1	1	3	4	2	0
47	17	2	1	7	1	2	2	4	2	0
48	55	10	2	1	1	4	2	1	2	0
49	31	5	2	5	1	2	3	4	2	0
50	47	8	1	2	1	1	3	1	2	0
51	37	6	2	2	1	1	2	1	2	0
52	19	2	2	7	1	2	3	1	2	0
53	59	10	1	1	1	1	2	1	2	0
54	42	7	2	2	1	1	2	1	2	0
55	13	1	1	7	1	2	2	1	2	0
56	60	11	1	1	1	1	2	1	2	0
57	50	9	2	1	1	1	2	1	2	0

58	10	1	1	7	1	2	2	1	2	0
59	53	9	1	2	1	1	3	6	2	0
60	47	8	2	2	1	1	3	6	2	0
61	15	2	2	7	1	2	3	6	2	0
62	12	1	1	7	1	2	2	6	2	0
63	18	2	2	7	1	2	3	6	2	0
64	21	3	1	7	1	2	3	6	2	0
65	42	7	2	4	1	2	4	1	2	0
66	37	6	2	1	1	2	3	1	2	0
67	30	5	1	4	1	1	4	1	2	0
68	28	4	2	4	1	2	4	1	2	0
69	26	4	1	2	1	2	4	1	2	0
70	40	7	1	2	1	1	3	1	2	0
71	35	6	2	2	1	1	2	1	2	0
72	12	1	1	7	1	2	2	1	2	0
73	15	2	1	7	1	2	3	1	2	0
74	10	1	1	7	1	2	2	1	2	0
75	56	10	1	1	1	1	1	1	2	0
76	48	8	2	1	1	1	3	1	2	0
77	20	3	1	5	1	2	1	1	2	0
78	16	2	2	7	1	2	3	1	2	0
79	22	3	2	7	1	2	4	1	2	0
80	45	8	1	2	1	1	3	1	2	0
81	32	5	2	4	1	1	4	1	2	0
82	12	1	1	7	1	2	3	1	2	0
83	21	3	2	5	1	2	2	1	2	0
84	44	7	1	3	1	1	3	1	2	0
85	19	2	1	7	1	2	3	1	2	0
86	40	7	2	2	1	1	3	1	2	0
87	12	2	1	7	1	2	2	1	2	0
88	48	8	1	2	1	1	3	1	2	0

89	36	6	2	2	1	1	3	1	2	0
90	69	12	1	3	1	1	3	1	2	0
91	52	9	2	1	1	1	1	1	2	0
92	28	4	1	2	1	2	3	1	2	0
93	24	3	1	2	1	2	3	1	2	0
94	22	3	1	2	1	2	3	1	2	0
95	48	8	1	3	1	1	3	1	2	0
96	32	5	2	2	1	1	3	1	2	0
97	12	1	2	7	1	2	2	1	2	0
98	43	7	1	3	1	2	2	1	2	0
99	80	15	1	5	1	1	2	1	2	0
100	68	12	2	2	1	1	2	1	2	0
101	43	7	1	4	1	1	4	1	2	0
102	40	7	1	5	1	2	3	1	2	0
103	27	4	2	4	1	1	4	1	2	0
104	51	9	1	2	1	1	2	1	2	0
105	40	7	2	2	1	1	3	1	2	0
106	18	2	2	7	1	2	2	1	2	0
107	25	4	2	7	1	2	3	6	2	0
108	11	1	1	7	1	2	2	6	2	0
109	60	11	2	1	1	4	2	6	2	0
110	49	8	1	2	1	1	2	6	2	0
111	45	8	1	4	1	1	3	6	2	0
112	56	10	1	2	1	4	2	6	2	0
113	40	7	1	4	1	1	3	6	2	0
114	50	9	1	2	1	1	3	6	2	0
115	30	5	1	4	1	1	3	6	2	0
116	40	7	1	2	1	1	3	6	2	0
117	82	15	1	2	1	1	3	6	2	0
118	60	11	1	4	1	1	3	6	2	0
119	46	8	2	2	1	4	2	6	2	0

120	67	12	1	1	1	1	2	6	2	0
121	29	4	1	7	1	2	4	6	2	0
122	65	12	1	1	1	1	2	6	2	0
123	76	14	2	1	1	4	1	6	2	0
124	40	7	1	1	1	1	2	6	2	0
125	45	8	1	3	1	1	2	6	2	0
126	58	10	2	2	1	4	2	6	2	0
127	70	13	1	2	1	1	3	6	2	0
128	75	14	2	2	1	4	2	6	2	0
129	82	15	1	2	1	1	2	6	2	0
130	36	6	1	1	1	1	2	6	2	0
131	70	13	2	1	1	4	2	6	2	0
132	52	9	1	2	1	2	2	6	2	0
133	69	12	2	1	1	4	2	6	2	0
134	35	6	2	2	1	3	3	6	2	0
135	90	15	2	1	3	4	1	6	2	0
136	77	14	1	1	3	1	3	6	2	0
137	68	12	2	1	1	4	2	6	2	0
138	55	10	1	3	1	1	3	6	2	0
139	50	9	1	2	5	1	3	6	2	0
140	55	10	1	1	3	1	2	6	2	0
141	60	11	2	1	1	4	1	6	2	0
142	40	7	1	2	1	1	3	6	2	0
143	42	7	1	2	1	1	3	6	2	0
144	75	14	2	2	1	4	1	6	2	0
145	82	15	1	1	3	1	2	6	2	0
146	45	8	1	3	1	1	3	4	2	0
147	61	11	2	1	1	4	2	4	2	0
148	74	13	1	2	1	1	2	4	2	0
149	58	10	1	1	1	1	2	4	2	0
150	50	9	2	2	1	4	2	4	2	0

151	50	9	1	1	1	1	2	4	2	0
152	50	9	1	3	1	1	3	4	2	0
153	62	11	1	3	1	1	2	4	2	0
154	39	6	1	3	1	1	3	4	2	0
155	45	8	1	2	1	1	3	4	2	0
156	65	12	1	1	1	1	1	6	2	0
157	65	12	2	1	1	4	1	6	2	0
158	80	15	2	2	1	4	2	6	2	0
159	65	12	2	6	1	4	4	6	2	0
160	75	14	1	3	1	1	2	6	2	0
161	38	6	1	3	1	1	3	6	2	0
162	60	11	2	1	1	4	2	6	2	0
163	70	13	1	2	1	1	2	6	2	0
164	72	13	2	2	1	4	2	6	2	0
165	60	11	1	2	1	1	2	6	2	0
166	62	11	2	1	1	4	1	6	2	0
167	81	15	1	1	1	1	2	6	2	0
168	60	11	1	2	1	1	1	6	2	0
169	76	14	2	1	1	4	2	6	2	0
170	70	13	2	1	1	4	1	6	2	0
171	53	9	2	5	1	4	3	6	2	0
172	33	5	1	2	1	1	3	6	2	0
173	56	10	1	3	3	1	2	6	2	0
174	65	12	2	2	1	4	2	6	2	0
175	60	11	1	1	3	1	1	6	2	0
176	72	13	2	2	1	4	1	6	2	0
177	34	5	1	3	1	1	2	6	2	0
178	38	6	1	3	1	1	3	6	2	0
179	52	9	1	4	1	1	3	6	2	0
180	67	12	2	5	1	4	2	6	2	0
181	33	5	2	3	1	4	3	6	2	0

182	73	13	1	3	1	1	2	6	2	0
183	54	9	1	1	3	1	2	6	2	0
184	59	10	1	1	1	1	2	6	2	0
185	80	15	1	5	1	1	2	4	2	0
186	68	12	2	1	3	4	2	4	2	0
187	90	15	1	5	1	4	1	4	2	0
188	82	15	2	2	3	4	2	4	2	0
189	27	4	1	3	1	1	3	4	2	0
190	75	14	2	5	1	4	1	4	2	0
191	80	15	2	1	3	4	1	4	2	0
192	96	15	2	3	1	4	1	3	2	0
193	70	13	1	4	1	1	4	3	2	0
194	65	12	2	1	1	4	2	3	2	0
195	50	9	2	1	1	1	2	6	2	0
196	29	4	2	2	1	1	3	6	2	0
197	24	3	2	1	1	1	3	6	2	0
198	54	9	2	1	1	1	2	6	2	0
199	75	14	2	1	1	4	1	6	2	0
200	80	15	2	1	1	4	2	6	2	0
201	77	14	2	1	1	4	1	6	2	0
202	32	5	2	1	1	1	3	6	2	0
203	85	15	2	1	1	4	1	6	2	0
204	50	9	2	1	1	1	3	6	2	0
205	47	8	1	1	1	1	2	6	2	0
206	80	15	1	1	1	4	1	6	2	0
207	78	14	2	1	1	4	1	6	2	0
208	70	13	1	3	1	1	3	6	2	0
209	80	15	2	5	1	4	1	6	2	0
210	92	15	2	5	1	4	1	6	2	0
211	93	15	1	5	1	1	1	6	2	0
212	45	8	1	1	1	1	2	6	2	0

213	75	14	1	1	1	1	1	6	2	0
214	50	9	2	1	1	1	1	6	2	0
215	70	13	2	1	1	1	1	6	2	0
216	78	14	1	1	1	1	2	6	2	0
217	38	6	1	2	1	1	3	6	2	0
218	70	13	1	1	1	1	2	6	2	0
219	62	11	2	1	1	1	2	6	2	0
220	40	7	2	1	1	4	3	6	2	0
221	75	14	1	1	1	1	2	6	2	0
222	75	14	1	1	1	1	2	6	2	0
223	77	14	1	1	1	1	2	6	2	0
224	65	13	2	1	1	4	2	6	2	0
225	20	3	1	2	1	2	3	6	2	0
226	65	12	1	1	1	1	2	6	2	0
227	22	3	2	2	1	2	2	6	2	0
228	80	15	1	1	3	1	2	6	2	0
229	38	6	1	2	3	1	2	6	2	0
230	65	12	2	1	3	1	2	6	2	0
231	72	13	2	1	1	1	2	6	2	0
232	45	8	1	2	1	1	3	6	2	0
233	75	14	1	1	1	1	2	6	2	0
234	37	6	2	2	1	1	2	6	2	0
235	57	10	1	1	1	1	2	6	2	0
236	72	13	1	1	1	1	2	6	2	0
237	55	10	2	2	1	4	2	6	2	0
238	35	6	1	1	1	1	2	6	2	0
239	36	6	1	2	1	1	2	6	2	0
240	75	14	1	1	1	1	2	6	2	0
241	60	11	1	3	1	1	2	6	2	0
242	62	11	1	1	1	1	3	6	2	0
243	82	15	1	1	1	1	2	6	2	0

244	84	15	1	1	3	1	1	6	2	0
245	56	10	1	1	3	1	2	6	2	0
246	83	15	1	1	1	1	2	6	2	0
247	42	7	2	1	1	4	2	6	2	0
248	70	13	1	2	1	1	2	6	2	0
249	80	15	2	1	1	4	2	6	2	0
250	65	12	1	1	1	1	3	6	2	0
251	46	8	1	3	1	1	2	6	2	0
252	32	5	1	2	1	1	3	6	2	0
253	30	5	1	2	1	2	3	6	2	0
254	25	4	1	8	1	2	2	6	2	0
255	85	15	2	1	1	4	1	6	2	0
256	42	7	1	1	1	1	2	6	2	0
257	63	11	1	2	1	1	2	6	2	0
258	56	10	2	1	1	1	2	6	2	0
259	60	11	1	1	1	1	2	6	2	0
260	58	10	2	2	1	1	2	6	2	0
261	58	10	1	1	3	1	2	6	2	0
262	50	9	2	1	1	1	2	6	2	0
263	61	11	1	1	1	1	2	6	2	0
264	52	9	2	2	1	4	2	6	2	0
265	66	12	1	1	1	1	2	6	2	0
266	58	12	1	2	1	1	2	6	2	0
267	56	12	2	2	1	1	2	6	2	0
268	78	14	1	1	3	1	2	6	2	0
269	73	12	1	1	3	1	2	6	2	0
270	65	12	2	1	1	4	2	6	2	0
271	50	9	1	2	1	1	3	6	2	0
272	88	15	1	1	1	1	2	6	2	0
273	63	11	1	2	1	1	2	6	2	0
274	68	12	1	2	1	1	2	6	2	0

275	65	12	1	1	1	1	2	6	2	0
276	70	13	1	1	1	1	2	6	2	0
277	70	13	2	1	1	4	3	6	2	0
278	75	14	2	1	1	4	1	6	2	0
279	32	5	1	2	2	1	3	6	1	0
280	75	14	1	1	3	1	2	6	2	0
281	20	3	2	8	5	1	1	6	2	0
282	50	9	1	5	3	1	3	6	2	0
283	56	10	2	1	1	4	1	6	2	0
284	70	13	1	6	1	1	4	6	2	0
285	77	14	1	2	1	4	3	6	1	0
286	63	11	1	6	1	1	2	6	2	0
287	62	11	1	1	3	1	2	6	2	0
288	35	6	1	2	3	1	3	6	2	0
289	55	10	2	1	1	4	3	6	1	2
290	50	9	1	1	3	1	2	6	2	0
291	38	6	2	1	1	4	2	6	2	0
292	33	5	1	1	1	1	3	6	2	0
293	75	14	1	1	1	1	2	3	2	0
294	36	6	1	3	1	1	3	3	2	0
295	67	12	2	2	1	1	2	3	2	0
296	35	6	1	4	1	1	4	3	2	0
297	53	9	1	3	1	1	2	3	2	0
298	67	12	1	2	1	1	3	3	2	0
299	78	14	2	1	1	1	2	3	2	0
300	40	7	1	3	1	1	3	3	2	0
301	59	10	1	4	1	1	4	3	2	0
302	70	13	1	2	1	1	3	3	2	0
303	59	10	2	2	1	4	3	3	2	0
304	36	6	1	3	1	1	3	3	2	0
305	91	15	1	5	3	4	3	3	2	0

306	56	10	1	4	1	1	4	4	2	0
307	89	15	1	5	1	4	4	3	2	0
308	61	11	2	5	1	4	2	3	2	0
309	41	7	1	3	1	1	2	3	2	0
310	65	12	2	6	1	2	4	3	2	0
311	42	7	1	3	1	1	3	3	2	0
312	49	8	1	3	3	1	3	3	2	0
313	39	6	2	2	1	4	3	3	2	0
314	80	15	2	5	1	4	3	3	2	0
315	31	5	1	2	1	1	2	3	2	0
316	77	14	2	5	3	4	2	3	2	0
317	20	3	1	2	1	2	2	3	2	0
318	74	13	1	1	1	1	3	3	2	0
319	67	12	2	2	1	4	2	3	2	0
320	59	10	2	2	1	4	3	3	2	0
321	85	15	1	5	1	4	1	3	2	0
322	36	6	1	2	1	1	3	3	2	0
323	72	13	1	1	1	1	3	3	2	0
324	42	7	2	1	1	4	3	3	2	0
325	22	3	1	3	1	1	3	3	2	0
326	26	4	1	2	1	1	3	3	2	0
327	30	5	1	2	1	1	3	3	2	0
328	60	11	1	2	1	1	3	3	2	0
329	30	5	1	3	1	2	3	3	2	0
330	70	13	1	6	1	1	4	3	2	0
331	74	13	1	6	1	1	3	3	2	0
332	89	15	1	5	1	1	2	2	2	0
333	49	8	1	2	1	2	4	3	2	0
334	37	6	1	3	1	1	3	3	2	0
335	48	8	1	2	1	1	2	3	2	0
336	60	11	2	2	1	4	2	6	2	0

337	48	8	1	3	1	1	2	6	2	0
338	61	11	1	2	1	1	2	3	2	0
339	67	12	2	2	1	1	2	3	2	0
340	56	10	1	4	1	4	2	3	2	0
341	74	13	2	1	1	4	2	1	2	0
342	54	9	2	4	1	4	4	2	2	0
343	34	5	1	4	1	1	4	3	2	0
344	37	6	1	3	1	1	3	3	2	0
345	69	12	1	3	1	1	2	3	2	0
346	70	13	2	1	1	4	2	1	2	0
347	49	8	1	3	1	1	3	3	2	0
348	55	10	1	4	3	1	2	3	2	0
349	70	13	2	1	1	4	2	6	1	0
350	52	9	2	2	1	4	3	5	2	0
351	49	8	1	2	3	1	2	3	2	0
352	53	9	1	3	1	1	2	3	2	0
353	84	15	1	1	1	1	3	3	2	0
354	61	11	1	2	1	1	3	3	2	0
355	51	9	1	3	1	1	2	3	2	0
356	37	6	1	1	3	1	2	3	2	0
357	71	13	1	1	3	4	2	3	2	0
358	57	10	2	2	1	4	2	3	2	0
359	70	13	1	3	1	4	2	3	2	0
360	87	15	1	3	3	4	2	3	2	0
361	49	8	2	2	1	4	2	3	2	0
362	54	9	2	2	1	4	2	3	2	0
363	55	10	2	2	1	4	3	6	2	0
364	60	11	1	1	1	1	2	3	2	0
365	90	15	2	2	3	4	2	3	2	0
366	61	11	1	3	1	4	2	3	2	0
367	62	11	1	6	1	1	3	3	2	0

368	52	9	1	1	1	1	2	3	2	0
369	40	7	1	3	1	1	2	3	2	0
370	39	6	1	2	1	1	2	3	2	0
371	61	11	1	3	1	1	2	3	2	0
372	60	11	1	3	1	1	2	3	2	0
373	42	7	1	3	1	1	2	3	2	0
374	64	11	1	1	1	1	2	3	2	0
375	99	15	1	5	1	4	2	3	2	0
376	40	7	1	1	1	1	3	3	2	0
377	50	9	2	4	1	4	2	3	2	0
378	38	6	1	2	1	2	2	3	2	0
379	44	7	1	3	1	1	3	3	2	0
380	54	9	1	2	1	1	4	3	2	0
381	39	6	2	2	1	4	3	3	2	0
382	30	5	1	2	1	1	3	3	2	0
383	83	15	1	5	3	1	1	3	2	0
384	59	10	1	3	1	1	3	3	2	0
385	67	12	1	1	1	1	2	3	2	0
386	75	14	1	1	1	1	2	3	2	0
387	30	5	1	1	1	1	2	3	2	0
388	65	12	2	1	1	4	2	3	2	0
389	70	13	2	1	1	4	1	3	2	0
390	50	9	1	1	1	4	3	3	2	0
391	40	7	1	1	1	1	2	3	2	0
392	48	8	1	1	1	1	2	3	2	0
393	45	8	1	1	1	1	2	3	2	0
394	51	9	1	1	1	1	2	3	2	0
395	44	7	1	2	1	1	3	3	2	0
396	73	13	2	1	1	4	2	3	2	0
397	52	9	1	1	1	1	2	3	2	0
398	64	11	2	1	1	4	1	3	2	0

399	68	12	1	1	1	1	2	3	2	0
400	48	8	1	1	1	1	2	3	2	0
401	62	11	1	2	1	1	2	3	2	0
402	68	12	1	1	1	1	2	3	2	0
403	60	11	1	1	1	1	3	3	2	0
404	37	6	1	1	3	1	2	3	2	0
405	63	11	2	1	1	4	1	3	2	0
406	53	9	1	2	1	1	2	5	2	0
407	39	6	2	1	1	4	2	5	2	0
408	62	11	2	1	1	4	2	5	2	0
409	50	9	1	1	1	1	2	5	2	0
410	68	12	1	1	1	1	3	5	2	0
411	60	11	1	2	1	1	2	5	2	0
412	76	14	1	2	1	1	2	5	2	0
413	62	12	1	1	1	1	2	5	2	0
414	83	15	2	1	1	4	1	5	2	0
415	66	12	2	1	1	4	2	5	2	0
416	81	15	2	1	1	4	1	5	2	0
417	66	12	2	2	1	4	3	5	2	0
418	68	12	2	1	1	4	3	5	2	0
419	66	12	1	2	1	1	2	3	2	0
420	80	15	1	1	1	1	2	3	2	0
421	58	10	1	1	1	1	2	3	2	0
422	70	13	1	1	1	1	2	3	2	0
423	50	9	1	1	1	1	2	3	2	0
424	56	10	2	1	1	1	3	3	2	0
425	45	8	1	1	1	1	2	3	2	0
426	38	6	1	1	1	1	3	3	2	0
427	35	6	1	1	1	1	2	3	2	0
428	46	8	1	1	1	1	3	3	2	0
429	75	14	1	1	1	1	2	3	2	0

430	49	8	1	1	1	1	3	3	2	0
431	40	7	1	1	1	1	2	3	2	0
432	32	5	1	1	1	1	2	3	2	0
433	64	11	2	1	1	4	2	3	2	0
434	46	8	2	2	1	1	3	3	2	0
435	55	10	2	2	1	4	3	3	2	0
436	35	6	1	2	1	1	3	3	2	0
437	38	6	1	1	1	1	3	3	2	0
438	50	9	1	2	1	1	2	3	2	0
439	42	7	1	1	1	1	2	3	2	0
440	32	5	2	3	1	1	3	3	2	0
441	27	4	1	2	1	1	3	3	2	0
442	54	9	1	3	1	1	2	3	2	0
443	42	7	1	2	1	1	3	6	2	0
444	41	7	1	2	1	1	3	3	2	0
445	32	5	1	1	1	1	3	3	2	0
446	42	7	1	2	1	1	3	3	2	0
447	37	6	1	2	1	1	3	3	2	0
448	39	6	2	2	1	1	2	4	2	0
449	70	13	1	2	1	1	3	4	2	0
450	70	13	1	1	1	4	1	4	2	0
451	50	9	1	2	1	1	2	4	2	0
452	45	8	1	2	1	1	3	4	2	0
453	70	13	1	1	1	4	1	4	2	0
454	64	11	1	1	1	1	3	4	2	0
455	68	12	1	1	1	1	2	4	2	0
456	40	7	1	2	1	1	3	4	2	0
457	48	8	1	1	1	1	2	4	2	0
458	64	11	1	1	5	4	1	4	2	0
459	80	15	1	1	5	1	1	4	2	0
460	48	8	1	1	1	1	2	4	2	0

461	64	11	2	1	1	4	2	4	2	0
462	68	12	2	1	1	4	2	4	2	0
463	36	6	1	1	1	1	2	4	2	0
464	39	6	1	2	1	1	3	3	2	0
465	40	7	2	1	1	4	2	3	2	0
466	51	9	1	1	1	1	2	3	2	0
467	89	15	1	1	1	1	2	3	2	0
468	70	13	2	2	1	4	2	3	2	0
469	45	8	1	1	1	1	2	3	2	0
470	63	11	1	2	1	1	2	3	2	0
471	67	12	1	1	1	1	2	3	2	0
472	68	12	2	2	1	4	2	3	2	0
473	43	7	1	2	1	1	2	3	2	0
474	29	4	1	2	1	1	3	3	2	0
475	36	6	1	2	1	1	3	3	2	0
476	24	3	1	1	1	1	3	3	2	0
477	42	7	1	1	1	1	3	3	2	0
478	50	9	2	2	1	4	3	3	2	0
479	32	5	1	2	1	1	3	6	2	0
480	43	7	1	2	1	1	3	6	2	0
481	39	6	2	2	1	1	2	6	2	0
482	23	3	2	7	1	2	3	3	2	0
483	40	7	1	2	1	1	3	3	2	0
484	23	3	2	7	1	2	3	3	2	0
485	50	9	1	2	1	1	3	3	2	0
486	40	7	1	2	1	1	3	3	2	0
487	68	11	2	1	1	4	1	3	2	0
488	24	3	2	7	1	2	3	3	2	0
489	72	13	2	2	1	4	2	3	2	0
490	39	6	1	2	1	1	3	3	2	0
491	70	13	1	1	1	1	3	3	2	0

492	43	7	1	2	1	1	3	3	2	0
493	40	7	1	2	1	1	2	3	2	0
494	69	12	1	2	1	1	3	3	2	0
495	38	6	2	2	1	1	3	3	2	0
496	40	7	1	2	1	1	3	3	2	0
497	39	6	2	2	1	1	2	3	2	0
498	42	7	1	1	1	1	3	3	2	0
499	35	6	1	2	1	2	3	3	2	0
500	80	15	2	1	1	4	2	3	2	0
501	60	11	1	1	1	1	2	3	2	0
502	82	15	1	1	1	1	3	4	2	0
503	52	9	2	2	1	4	3	4	2	0
504	47	8	1	2	1	1	3	4	2	0
505	55	10	1	2	1	1	2	4	2	0
506	40	7	2	2	1	4	2	4	2	0
507	35	6	1	2	1	1	3	4	2	0
508	38	6	1	1	1	1	3	4	2	0
509	46	8	1	2	1	1	2	4	2	0
510	45	8	2	2	1	1	3	4	2	0
511	64	11	1	2	1	1	3	4	2	0
512	54	9	2	2	1	1	3	4	2	0
513	37	6	1	3	1	1	3	4	2	0
514	50	9	2	2	1	4	3	4	2	0
515	40	7	1	4	1	1	4	4	2	0
516	60	11	1	4	1	1	4	3	2	0
517	63	11	1	2	1	1	1	3	2	0
518	62	11	1	4	1	1	3	3	2	0
519	70	13	1	6	1	1	4	3	2	0
520	80	15	2	5	1	1	2	3	2	0
521	88	15	1	6	1	1	2	3	2	0
522	50	9	2	6	1	1	2	3	2	0

523	51	9	2	2	1	1	3	3	2	0
524	72	13	1	2	1	1	3	3	2	0
525	37	6	1	2	1	1	3	3	2	0
526	35	6	1	2	1	1	3	3	2	0
527	35	6	1	3	1	1	3	3	2	0
528	26	4	1	3	1	1	3	3	2	0
529	60	11	1	3	1	1	3	3	2	0
530	45	8	1	2	1	1	3	3	2	0
531	68	12	1	3	1	1	2	3	2	0
532	71	13	1	2	1	1	4	3	2	0
533	58	10	2	2	1	4	3	3	2	0
534	42	7	1	3	1	1	2	3	2	0
535	68	12	1	1	1	1	3	3	2	0
536	20	3	1	7	1	2	3	3	2	0
537	68	12	1	2	1	1	3	4	2	0
538	73	13	1	2	1	1	3	4	2	0
539	38	6	1	3	1	1	2	4	2	0
540	70	13	1	1	1	1	2	4	2	0
541	80	15	1	6	1	1	2	4	2	0
542	66	12	1	2	1	1	2	4	2	0
543	69	12	1	6	1	1	4	4	2	0
544	45	8	1	2	1	1	3	4	2	0
545	53	9	1	2	1	1	2	4	2	0
546	56	10	1	4	1	1	4	4	2	0
547	45	8	1	2	1	1	3	4	2	0
548	60	11	1	1	1	1	2	4	2	0
549	60	11	1	1	1	1	3	4	2	0
550	43	7	2	2	1	4	3	4	2	0
551	60	11	1	3	1	1	3	4	2	0
552	56	10	1	2	1	1	4	4	2	0
553	40	7	1	2	1	1	4	4	2	0

554	45	8	1	2	1	1	2	4	2	0
555	45	8	2	2	1	1	3	4	2	0
556	65	12	1	2	1	1	2	4	2	0
557	59	10	1	2	1	1	3	4	2	0
558	50	9	1	1	1	3	2	3	2	0
559	58	10	1	2	1	1	2	3	2	0
560	38	6	2	2	1	1	3	3	2	0
561	41	7	1	2	1	1	2	3	2	0
562	38	6	2	2	1	1	2	3	2	0
563	44	7	1	3	3	1	2	3	2	0
564	61	11	1	4	1	1	2	3	2	0
565	49	8	1	2	1	1	2	3	2	0
566	83	15	2	3	1	4	2	3	2	0
567	40	7	1	5	1	1	3	3	2	0
568	65	12	2	1	1	4	2	3	2	0
569	60	11	2	2	1	1	3	3	2	0
570	71	13	1	2	1	1	3	3	2	0
571	67	12	1	1	1	1	2	3	2	0
572	33	5	1	3	5	1	2	3	2	0
573	21	3	1	7	1	2	3	3	2	0
574	16	2	1	5	1	2	1	3	2	0
575	14	1	1	7	1	2	2	3	2	0
576	34	5	2	2	1	1	3	3	2	0
577	41	7	2	1	1	1	2	3	2	0
578	52	9	1	3	1	1	2	3	2	0
579	43	7	1	2	3	1	3	3	2	0
580	25	4	2	2	1	1	3	3	2	0
581	64	11	1	5	1	1	3	3	2	0
582	50	9	1	3	3	1	2	3	2	0
583	45	8	2	2	1	4	2	3	2	0
584	37	6	1	3	1	1	3	3	2	0

585	60	11	1	3	1	1	2	3	2	0
586	65	12	2	2	1	4	2	3	2	0
587	73	13	1	2	1	1	2	3	2	0
588	60	11	1	2	1	1	2	3	2	0
589	43	7	2	2	1	1	3	3	2	0
590	50	9	1	2	1	1	3	5	2	0
591	64	11	1	2	1	1	3	5	2	0
592	54	9	1	3	1	1	3	5	2	0
593	47	8	1	3	1	1	2	5	2	0
594	85	15	2	5	1	4	1	5	2	0
595	32	5	2	3	1	1	2	5	2	0
596	50	9	1	3	1	1	2	5	2	0
597	53	9	1	2	1	1	3	5	2	0
598	23	3	1	7	1	2	4	5	2	0
599	40	7	2	8	1	1	2	5	2	0
600	45	8	1	3	1	1	2	5	2	0
601	70	13	1	2	3	1	3	5	2	0
602	75	14	1	6	1	1	2	5	2	0
603	60	11	1	2	1	1	3	5	2	0
604	80	15	2	6	1	1	2	5	2	0
605	45	8	1	3	1	1	3	5	2	0
606	84	15	1	6	1	1	2	3	2	0
607	69	12	2	1	1	4	2	3	2	0
608	68	12	1	3	1	1	2	3	2	0
609	68	12	2	6	1	4	4	3	2	0
610	73	13	1	1	1	1	2	3	2	0
611	45	8	1	2	1	1	4	3	2	0
612	65	12	1	1	1	1	3	3	2	0
613	61	11	1	5	1	1	2	3	2	0
614	49	8	2	3	1	2	4	3	2	0
615	52	9	2	2	1	1	3	3	2	0

616	52	9	1	3	1	1	2	3	2	0
617	46	8	2	3	1	1	2	3	2	0
618	60	11	1	3	1	1	2	3	2	0
619	48	8	2	3	1	1	3	3	2	0
620	43	7	2	3	1	1	3	3	2	0
621	42	7	1	3	1	1	2	3	2	0
622	38	6	2	4	1	1	4	3	2	0
623	65	12	1	1	1	1	3	3	2	0
624	62	11	1	2	1	1	3	3	2	0
625	70	13	2	1	1	1	2	3	2	0
626	24	3	2	2	1	1	1	3	2	0
627	60	11	1	2	1	1	4	4	2	0
628	60	11	1	2	1	1	2	4	2	0
629	50	9	1	2	1	1	1	4	2	0
630	37	6	1	3	1	1	3	4	2	0
631	48	8	1	3	3	1	3	4	2	0
632	30	5	1	3	1	1	3	4	2	0
633	62	11	1	4	1	1	2	4	2	0
634	71	13	1	3	3	1	1	4	2	0
635	35	6	1	3	1	1	3	4	2	0
636	26	4	2	2	1	1	2	4	2	0
637	45	8	1	3	1	1	3	4	2	0
638	74	13	2	2	1	1	1	4	2	0
639	41	7	2	4	1	1	4	4	2	0
640	19	2	1	8	1	2	3	4	2	0
641	51	9	1	3	1	1	3	4	2	0
642	50	9	2	3	1	2	3	4	2	0
643	65	12	2	1	1	4	1	4	2	0
644	90	15	1	5	1	1	1	4	2	0
645	62	11	2	6	1	4	2	4	2	0
646	54	9	2	2	1	4	3	4	2	0

647	68	12	1	2	1	1	3	4	2	0
648	57	10	1	2	1	4	3	4	2	0
649	60	11	1	5	1	1	3	4	2	0
650	43	7	2	2	1	4	2	4	2	0
651	46	8	2	2	1	4	2	4	2	0
652	37	6	1	3	1	1	3	4	2	0
653	95	15	1	5	1	1	2	4	2	0
654	75	14	2	6	1	1	3	4	2	0
655	87	15	1	5	1	1	2	4	2	0
656	40	7	1	2	1	1	3	4	2	0
657	26	4	2	2	1	1	3	4	2	0
658	67	12	2	2	1	4	2	4	2	0
659	70	13	1	6	1	1	3	4	2	0
660	57	10	2	2	1	1	3	4	2	0
661	63	11	1	2	1	1	2	4	2	0
662	60	11	1	2	1	1	3	4	2	0
663	38	6	2	3	1	1	3	4	2	0
664	56	10	2	2	1	1	3	4	2	0
665	48	8	1	2	1	1	3	4	2	0
666	58	10	2	2	1	4	2	4	2	0
667	30	5	2	2	1	1	3	4	2	0
668	65	12	1	3	1	1	2	4	2	0
669	58	10	1	2	1	1	3	6	2	0
670	45	8	2	2	1	1	3	3	2	0
671	45	8	1	2	1	1	3	3	2	0
672	75	14	1	2	1	1	2	3	2	0
673	48	8	1	4	1	1	2	4	2	0
674	40	7	1	2	1	1	2	4	2	0
675	72	13	1	2	1	1	2	4	2	0
676	30	5	1	5	1	2	1	4	2	0
677	61	11	2	3	1	1	2	4	2	0

678	47	8	1	2	1	1	3	3	2	0
679	60	11	2	1	1	4	2	3	2	0
680	62	11	1	2	1	1	2	3	2	0
681	62	11	1	3	1	1	2	3	2	0
682	80	15	2	2	1	4	1	3	2	0
683	65	12	1	2	1	1	2	3	2	0
684	46	8	1	6	1	1	4	3	2	0
685	44	7	1	2	1	4	3	3	2	0
686	26	4	1	2	1	1	4	3	2	0
687	36	6	1	3	1	1	4	3	2	0
688	48	8	1	3	1	4	2	3	2	0
689	50	9	1	2	1	1	3	3	2	0
690	30	5	1	3	1	1	3	3	2	0
691	25	4	1	2	1	1	3	3	2	0
692	38	6	1	3	1	1	3	3	2	0
693	58	10	1	2	1	1	2	3	2	0
694	39	6	1	3	1	1	3	3	2	0
695	90	15	1	4	1	4	4	3	2	0
696	52	9	1	2	1	1	3	3	2	0
697	37	6	1	2	1	1	3	3	2	0
698	55	10	1	2	1	1	3	3	2	0
699	31	5	1	3	1	2	3	3	2	0
700	56	10	2	1	1	1	2	3	2	0
701	41	7	2	2	1	1	2	3	2	0
702	48	8	1	2	1	1	3	3	2	0
703	60	11	1	1	1	1	2	3	2	0
704	77	14	2	2	1	4	2	3	2	0
705	38	6	1	3	1	1	3	3	2	0
706	78	14	1	2	1	4	2	5	2	0
707	62	11	1	2	1	1	3	5	2	0
708	53	9	1	2	1	1	2	5	2	0

709	78	14	1	4	1	1	2	5	2	0
710	39	6	1	5	1	1	2	5	2	0
711	42	7	1	2	1	1	2	5	2	0
712	65	12	2	1	1	4	2	5	2	0
713	50	9	2	1	1	1	2	5	2	0
714	56	10	1	5	1	1	2	5	2	0
715	37	6	1	2	1	1	4	5	2	0
716	45	8	1	3	1	1	2	5	2	0
717	52	9	2	4	1	4	2	5	2	0
718	43	7	1	2	1	1	2	3	2	0
719	39	6	2	2	1	1	3	3	2	0
720	70	13	1	2	1	1	3	3	2	0
721	53	9	2	2	1	4	2	3	2	0
722	60	11	1	5	1	1	2	3	2	0
723	67	12	1	2	1	1	3	3	2	0
724	55	10	1	2	1	1	3	3	2	0
725	55	10	2	2	1	4	2	3	2	0
726	79	14	2	2	1	4	2	3	2	0
727	47	8	2	2	1	4	3	3	2	0
728	66	12	2	2	1	4	2	3	2	0
729	50	9	1	3	1	1	3	3	2	0
730	32	5	1	2	1	4	2	3	2	0
731	34	5	1	8	1	1	4	3	2	0
732	69	12	2	2	1	4	3	3	2	0
733	50	9	2	2	1	4	2	3	2	0
734	65	12	1	1	1	1	2	3	2	0
735	41	7	2	2	1	4	3	3	2	0
736	80	15	2	1	1	4	2	3	2	0
737	52	9	2	2	1	4	2	3	2	0
738	67	12	2	2	1	4	3	3	2	0
739	56	10	1	2	1	1	2	3	2	0

740	50	9	2	1	1	4	2	3	2	0
741	56	10	2	2	1	4	3	3	2	0
742	50	9	1	2	1	1	3	3	2	0
743	52	9	2	2	1	4	2	3	2	0
744	75	13	1	2	1	4	2	3	2	0
745	45	8	1	4	1	1	3	3	2	0
746	50	9	1	2	1	1	3	3	2	0
747	68	12	1	3	1	1	2	3	2	0
748	45	8	2	1	1	4	3	3	2	0
749	65	12	2	1	1	4	2	3	2	0
750	50	9	1	2	1	1	3	3	2	0
751	60	11	2	2	1	4	2	3	2	0
752	53	9	2	2	1	4	3	3	2	0
753	65	12	1	1	1	1	3	3	2	0
754	28	4	2	8	1	1	4	3	2	0
755	75	14	1	2	1	1	2	3	2	0
756	37	6	1	2	1	1	3	3	2	0
757	52	9	2	4	1	4	4	3	2	0
758	27	4	1	2	1	1	3	3	2	0
759	53	9	1	2	1	1	3	3	2	0
760	48	8	1	2	3	1	3	3	2	0
761	60	11	1	2	3	1	3	3	2	0
762	63	11	2	1	1	4	2	3	2	0
763	58	6	2	5	1	4	2	3	2	0
764	39	6	1	3	1	1	3	3	2	0
765	80	15	1	5	1	1	2	3	2	0
766	59	10	1	4	1	1	4	3	2	0
767	66	12	1	3	1	1	2	3	2	0
768	62	11	1	6	1	4	4	3	2	0
769	48	8	2	2	1	4	2	3	2	0
770	58	10	2	3	1	4	2	3	2	0

771	65	12	1	3	1	1	3	3	2	0
772	75	14	1	3	1	1	2	3	2	0
773	52	9	1	4	1	1	4	3	2	0
774	85	15	2	2	1	4	2	3	2	0
775	55	10	1	2	1	1	2	3	2	0
776	55	10	1	3	1	1	3	3	2	0
777	44	7	2	2	1	4	3	3	2	0
778	73	13	2	2	1	4	3	3	2	0
779	29	4	2	7	1	2	4	3	2	0
780	35	6	1	2	3	1	3	3	2	0
781	57	10	2	2	1	4	3	3	2	0
782	65	12	1	2	1	1	3	3	2	0
783	53	9	2	1	1	4	3	3	2	0
784	33	5	1	2	1	1	3	3	2	0
785	48	8	1	2	1	1	4	3	2	0
786	60	11	1	2	1	1	3	3	2	0
787	67	12	1	4	1	1	4	4	2	0
788	50	9	2	2	1	1	2	4	2	0
789	38	6	1	3	1	1	3	4	2	0
790	60	11	1	4	1	1	4	4	2	0
791	65	12	1	4	1	4	4	4	2	0
792	53	9	2	1	1	1	3	4	2	0
793	60	11	1	1	1	4	3	4	2	0
794	57	10	2	2	1	4	4	4	2	0
795	35	6	2	2	1	4	3	4	2	0
796	60	11	1	3	1	1	3	4	2	0
797	66	12	2	1	1	4	3	4	2	0
798	48	8	1	1	1	1	3	4	2	0
799	37	6	1	1	1	1	3	4	2	0
800	37	6	1	3	1	1	3	4	2	0
801	42	7	1	1	1	1	3	4	2	0

802	37	6	1	3	1	1	3	4	2	0
803	69	12	1	2	1	1	3	4	2	0
804	60	11	1	4	1	1	4	3	2	0
805	73	13	2	2	1	4	2	3	2	0
806	50	9	2	2	1	4	3	3	2	0
807	42	7	2	2	1	4	2	3	2	0
808	66	12	1	3	1	1	3	3	2	0
809	49	8	1	2	1	1	3	3	2	0
810	32	5	1	3	1	1	3	3	2	0
811	48	8	1	2	1	1	4	3	2	0
812	62	11	1	2	1	4	3	3	2	0
813	37	6	1	2	3	1	3	3	2	0
814	52	9	1	3	1	1	3	3	2	0
815	69	12	2	1	1	4	2	3	2	0
816	51	9	1	2	1	1	3	3	2	0
817	57	10	2	4	1	1	4	4	2	0
818	52	9	1	2	1	1	3	4	2	0
819	62	11	1	4	1	6	4	4	2	0
820	47	8	1	3	1	1	2	4	2	0
821	36	6	1	3	1	1	3	3	2	0
822	60	11	1	2	1	1	3	6	2	0
823	55	10	1	4	1	1	4	6	2	0
824	48	8	1	3	1	1	3	3	2	0
825	43	7	1	3	1	1	3	3	2	0
826	59	10	2	2	1	4	3	3	2	0
827	45	8	2	2	1	4	3	3	2	0
828	49	8	1	1	1	1	2	3	2	0
829	65	12	1	1	1	4	2	3	2	0
830	67	12	1	3	3	1	3	3	2	0
831	60	11	1	1	1	1	3	3	2	0
832	66	12	1	3	1	1	3	3	2	0

833	58	10	1	3	1	1	3	3	2	0
834	33	5	1	3	1	1	3	3	2	0
835	65	12	2	2	1	4	3	3	2	0
836	39	6	1	3	1	1	3	3	2	0
837	63	11	1	2	1	1	2	4	2	0
838	75	14	1	1	1	1	3	4	2	0
839	63	11	1	1	1	1	3	4	2	0
840	48	8	1	3	1	1	3	4	2	0
841	54	9	2	2	1	4	3	4	2	0
842	79	14	1	1	1	1	3	4	2	0
843	57	10	2	2	1	4	2	4	2	0
844	38	6	1	1	1	2	3	4	2	0
845	79	14	2	2	1	4	2	4	2	0
846	47	8	1	3	1	1	3	4	2	0
847	35	6	1	1	1	1	1	6	1	1
848	29	4	1	5	1	2	1	6	2	0
849	65	12	1	1	1	4	3	6	2	0
850	65	12	1	1	1	1	2	6	2	0
851	65	11	2	1	1	4	1	6	1	1
852	27	4	1	1	1	2	2	6	1	1
853	28	4	2	5	1	2	1	4	1	1
854	68	12	2	1	1	4	1	6	2	0
855	11	1	1	7	1	2	2	6	1	1
856	36	6	2	4	1	1	4	6	2	0
857	71	13	2	1	1	4	1	6	2	0
858	31	5	2	5	1	2	3	6	2	0
859	37	6	2	1	1	1	2	6	2	0
860	20	3	2	7	1	2	4	4	2	0
861	60	11	2	1	1	4	2	4	2	0
862	50	9	1	4	1	1	3	4	2	0
863	65	12	2	1	1	4	2	4	2	0

864	40	7	2	1	1	1	2	4	2	0
865	45	8	2	1	1	4	3	4	2	0
866	81	15	1	6	1	1	3	4	2	0
867	48	8	1	1	1	1	1	4	2	0
868	57	10	2	1	1	4	2	4	2	0
869	66	12	2	2	1	4	2	4	2	0
870	48	8	1	2	1	1	2	4	2	0
871	39	6	2	1	1	1	3	4	2	0
872	42	7	1	3	1	1	2	6	1	1
873	49	8	1	3	1	1	3	4	2	0
874	27	4	2	3	1	2	3	5	2	0
875	53	9	1	2	1	1	3	4	2	0
876	85	15	2	6	1	4	1	4	2	0
877	40	7	2	1	1	1	1	4	2	0
878	45	8	2	2	1	1	3	4	2	0
879	55	10	2	2	1	1	3	4	2	0
880	32	5	1	3	1	1	3	4	2	0
881	35	6	1	3	1	2	2	4	2	0
882	27	4	1	3	1	1	2	4	2	0
883	62	11	2	1	1	4	2	4	2	0
884	68	12	2	4	1	1	4	4	2	0
885	33	5	1	3	1	1	2	4	2	0
886	57	10	1	8	1	1	4	4	2	0
887	23	3	2	3	1	1	3	4	2	0
888	31	5	1	2	1	1	3	4	2	0
889	27	4	1	2	1	2	3	4	2	0
890	56	10	1	3	1	1	3	4	2	0
891	45	8	1	1	3	1	2	4	2	0
892	73	13	1	1	1	1	2	4	2	0
893	71	13	1	1	1	1	2	4	2	0
894	60	11	2	6	1	1	3	4	2	0

895	61	11	2	1	1	1	1	4	2	0
896	52	9	2	4	1	1	4	4	2	0
897	81	15	2	5	1	1	2	4	2	0
898	50	9	1	2	1	1	3	4	2	0
899	75	14	1	5	1	1	2	4	2	0
900	40	7	2	1	1	1	4	4	2	0
901	36	6	1	2	3	1	3	4	2	0
902	29	4	1	2	1	2	3	4	2	0
903	45	8	1	3	1	1	2	4	2	0
904	45	8	1	1	1	1	4	4	2	0
905	83	15	2	5	1	4	1	4	2	0
906	66	12	2	2	1	1	2	4	2	0
907	39	6	1	2	1	1	4	4	2	0
908	48	8	2	4	1	1	4	4	2	0
909	42	7	2	1	1	4	1	4	2	0
910	48	8	1	1	1	1	3	4	2	0
911	50	9	2	1	1	4	2	6	2	0
912	45	8	1	3	1	1	2	6	2	0
913	75	14	2	1	1	4	1	6	2	0
914	49	8	2	2	1	1	3	5	2	0
915	45	8	1	2	1	1	3	5	2	0
916	56	10	1	2	1	1	3	1	2	0
917	40	7	2	2	1	1	3	5	2	0
918	65	12	1	2	1	1	3	5	2	0
919	39	6	1	3	1	2	4	5	2	0
920	30	5	1	4	1	2	4	5	2	0
921	37	6	1	3	1	2	3	5	2	0
922	37	6	2	4	1	2	3	5	2	0
923	42	7	2	2	1	1	3	5	2	0
924	58	10	2	1	1	1	3	5	2	0
925	60	11	2	5	1	1	4	5	2	0

926	58	10	2	3	1	1	3	5	2	0
927	78	14	2	5	1	1	2	5	2	0
928	40	7	1	4	1	1	4	5	2	0
929	35	6	2	2	1	1	3	5	2	0
930	35	6	1	2	1	1	3	5	2	0
931	32	5	1	2	1	1	2	3	2	0
932	45	8	1	2	1	1	2	5	2	0
933	51	9	1	4	1	1	4	3	2	0
934	31	5	1	3	1	2	2	3	2	0
935	40	7	1	3	3	1	3	3	2	0
936	83	15	2	1	1	4	2	3	2	0
937	61	11	1	2	1	1	2	3	2	0
938	61	11	1	1	1	1	2	3	2	0
939	81	15	2	1	1	4	2	3	2	0
940	31	5	1	2	1	1	3	3	2	0
941	47	8	2	2	1	1	2	3	2	0
942	65	12	1	1	1	1	2	3	2	0
943	70	13	2	5	1	4	2	3	2	0
944	95	15	1	1	1	4	2	3	2	0
945	65	12	2	1	1	4	2	3	2	0
946	70	13	1	1	1	1	2	4	2	0
947	51	9	1	2	1	1	2	4	2	0
948	75	14	1	1	1	1	2	4	2	0
949	71	13	1	2	1	4	2	4	2	0
950	65	12	1	2	1	1	2	4	2	0
951	31	5	2	1	1	1	3	4	2	0
952	61	11	1	1	1	1	2	4	2	0
953	61	11	1	1	1	1	2	4	2	0
954	55	10	1	2	1	1	2	4	2	0
955	71	13	1	1	1	1	2	4	2	0
956	65	12	1	2	1	1	2	4	2	0

957	61	11	1	1	1	1	2	4	2	0
958	47	8	1	3	1	1	3	4	2	0
959	50	9	2	2	1	4	2	4	2	0
960	71	13	2	1	1	4	2	4	2	0
961	40	7	2	2	1	1	3	4	2	0
962	66	12	2	1	1	4	2	4	2	0
963	81	15	2	6	1	4	2	4	2	0
964	31	5	1	2	1	1	3	3	2	0
965	71	13	1	3	1	1	2	3	2	0
966	76	14	2	1	1	4	2	3	2	0
967	35	6	1	3	1	1	3	3	2	0
968	81	15	2	1	1	1	2	3	2	0
969	61	11	1	2	1	1	2	3	2	0
970	49	8	1	2	1	1	3	3	2	0
971	56	10	2	2	1	4	2	3	2	0
972	70	13	1	1	1	1	2	3	2	0
973	60	11	2	1	1	4	2	3	2	0
974	58	10	1	2	1	1	2	3	2	0
975	50	9	2	2	1	1	2	3	2	0
976	38	6	1	1	1	1	3	3	2	0
977	50	9	1	1	1	1	2	3	2	0
978	42	7	1	2	1	1	3	3	2	0
979	56	10	2	1	1	4	2	3	2	0
980	36	6	1	2	1	1	3	3	2	0
981	48	8	1	2	1	1	3	3	2	0
982	61	11	1	1	1	1	2	3	2	0
983	67	12	2	1	1	4	2	3	2	0
984	61	11	1	2	1	1	2	3	2	0
985	65	12	1	2	1	1	2	4	2	0
986	35	6	1	2	1	1	3	4	2	0
987	61	11	2	2	1	4	2	4	2	0

988	66	12	1	1	1	1	2	4	2	0
989	44	7	1	2	1	1	3	4	2	0
990	50	9	1	2	1	1	2	4	2	0
991	47	8	1	2	1	1	3	4	2	0
992	45	8	1	2	1	1	3	4	2	0
993	71	13	2	1	1	4	3	4	2	0
994	61	11	1	2	3	1	2	4	2	0
995	66	12	1	3	1	1	2	4	2	0
996	53	9	2	1	1	1	2	4	2	0
997	38	6	1	4	1	1	4	4	2	0
998	57	10	1	1	1	1	2	4	2	0
999	21	3	2	7	1	2	3	4	2	0
1000	46	8	2	2	1	4	3	4	2	0
1001	61	11	2	2	1	4	2	4	2	0
1002	51	9	2	2	1	1	3	4	2	0
1003	56	10	1	1	3	1	2	3	2	0
1004	36	6	1	2	5	1	2	3	2	0
1005	31	5	1	2	1	1	3	3	2	0
1006	61	11	1	1	3	1	2	3	2	0
1007	38	6	2	2	1	1	3	3	2	0
1008	37	6	1	2	3	1	3	3	2	0
1009	48	8	1	2	1	1	3	3	2	0
1010	59	10	1	2	1	1	3	3	2	0
1011	28	4	2	1	1	1	3	3	2	0
1012	48	8	1	2	1	1	3	3	2	0
1013	56	10	1	3	1	1	2	3	2	0
1014	73	13	2	1	1	4	2	3	2	0
1015	60	11	2	2	1	1	4	3	2	0
1016	43	7	1	4	1	1	4	3	2	0
1017	67	12	1	3	1	1	4	3	2	0
1018	70	13	1	2	1	1	4	3	2	0

1019	75	14	1	5	1	1	4	3	2	0
1020	59	10	1	2	1	1	4	3	2	0
1021	40	7	1	3	1	1	4	3	2	0
1022	80	15	1	5	1	1	4	3	2	0
1023	65	12	1	5	1	1	4	3	2	0
1024	51	9	2	1	1	4	4	3	2	0
1025	58	10	1	3	1	1	2	3	2	0
1026	27	4	1	3	1	2	3	3	2	0
1027	35	6	2	3	1	1	3	3	2	0
1028	43	7	1	3	1	1	3	3	2	0
1029	31	5	2	2	1	1	3	3	2	0
1030	60	11	2	2	1	4	3	3	2	0
1031	62	11	1	2	1	1	3	3	2	0
1032	20	3	1	3	1	2	3	3	2	0
1033	30	5	1	3	1	1	2	3	2	0
1034	52	9	2	2	1	4	2	3	2	0
1035	35	6	2	4	1	3	4	3	2	0
1036	38	6	1	3	1	1	3	3	2	0
1037	44	7	1	3	1	1	3	3	2	0
1038	78	14	2	5	1	4	1	3	2	0
1039	28	4	2	2	1	1	4	3	2	0
1040	38	6	1	2	1	1	4	3	2	0
1041	45	8	1	3	1	1	4	3	2	0
1042	68	12	1	3	1	1	4	3	2	0
1043	50	9	2	5	1	1	2	3	2	0
1044	40	7	1	3	1	1	3	6	2	0
1045	75	14	1	5	1	1	4	3	2	0
1046	73	13	2	2	1	1	3	1	2	0
1047	83	15	1	5	1	1	3	1	2	0
1048	45	8	2	2	1	1	2	1	2	0
1049	55	10	2	2	1	1	2	1	2	0

1050	55	10	1	2	1	1	3	1	2	0
1051	50	9	2	1	1	4	4	3	2	0
1052	35	6	1	2	1	1	3	3	2	0
1053	65	12	1	1	1	4	2	3	2	0
1054	47	8	1	3	1	1	3	3	2	0
1055	45	8	1	4	1	1	4	3	2	0
1056	55	10	1	3	1	1	3	3	2	0
1057	45	8	1	2	1	1	3	3	2	0
1058	78	14	1	5	1	1	4	3	2	0
1059	68	12	1	2	1	1	3	4	2	0
1060	57	10	2	5	1	1	3	4	2	0
1061	38	6	2	2	1	4	4	4	2	0
1062	78	14	1	5	1	4	4	4	2	0
1063	60	11	1	2	1	4	2	4	2	0
1064	80	15	2	5	1	4	3	4	2	0
1065	58	10	2	4	1	1	4	4	2	0
1066	51	9	1	1	1	1	2	4	2	0
1067	45	8	1	3	1	1	3	4	2	0
1068	70	13	1	1	1	1	2	4	2	0
1069	70	13	2	5	1	4	2	4	2	0
1070	70	13	2	5	1	1	3	4	2	0
1071	65	12	1	1	1	1	2	4	2	0
1072	53	9	2	2	1	4	2	4	2	0
1073	45	8	2	4	1	1	3	4	2	0
1074	75	14	1	5	1	4	2	4	2	0
1075	60	11	1	1	1	1	3	4	2	0
1076	42	7	2	2	1	4	3	4	2	0
1077	102	15	2	5	1	4	2	4	2	0
1078	60	11	2	2	1	1	3	4	2	0
1079	60	11	2	2	1	1	3	3	2	0
1080	70	13	2	2	1	4	2	3	2	0

1081	48	8	2	2	1	1	2	3	2	0
1082	72	13	1	1	1	1	2	3	2	0
1083	50	9	1	2	1	1	2	3	2	0
1084	54	9	1	2	1	1	4	3	2	0
1085	63	11	1	2	1	1	2	3	2	0
1086	40	7	2	2	1	4	3	3	2	0
1087	70	13	1	2	1	1	2	3	2	0
1088	65	12	1	1	1	1	2	3	2	0
1089	68	12	1	4	1	1	4	3	2	0
1090	55	10	1	4	1	1	4	3	2	0
1091	60	11	1	8	1	1	4	3	2	0
1092	35	6	2	2	1	4	4	3	2	0
1093	49	8	1	2	1	4	4	3	2	0
1094	21	3	1	5	1	2	1	3	2	0
1095	46	8	1	2	1	1	3	3	2	0
1096	45	8	1	3	1	1	3	3	2	0
1097	50	9	1	3	1	1	2	4	2	0
1098	69	12	1	2	1	1	3	4	2	0
1099	41	7	2	2	1	1	3	4	2	0
1100	65	12	2	2	1	1	2	4	2	0
1101	60	11	2	5	1	1	3	4	2	0
1102	48	8	2	4	1	1	3	4	2	0
1103	45	8	2	5	1	1	2	5	2	0
1104	56	10	1	2	1	1	2	5	2	0
1105	80	15	1	5	1	1	2	5	2	0
1106	64	11	2	5	1	1	2	5	2	0
1107	45	8	1	3	1	1	4	5	2	0
1108	14	1	1	7	1	2	3	6	2	0
1109	70	13	1	2	1	1	3	6	2	0
1110	32	5	1	3	1	1	3	6	2	0
1111	45	8	1	2	1	1	3	3	2	0

1112	75	14	2	5	1	1	3	3	2	0
1113	80	15	1	5	1	1	4	3	2	0
1114	31	5	1	3	1	1	2	3	2	0
1115	80	15	1	5	3	1	3	3	2	0
1116	30	5	2	2	1	1	3	3	2	0
1117	37	6	1	2	1	1	3	3	2	0
1118	40	7	1	3	1	1	3	3	2	0
1119	70	13	1	5	1	1	3	3	2	0
1120	80	15	2	1	1	4	1	3	2	0
1121	55	10	2	2	1	1	4	3	2	0
1122	40	7	1	3	1	1	2	4	2	0
1123	53	9	1	2	1	3	3	3	2	0
1124	35	6	2	2	1	1	3	3	2	0
1125	46	8	1	3	1	1	2	3	2	0
1126	61	11	1	1	3	1	2	3	2	0
1127	30	5	1	3	1	1	3	3	2	0
1128	61	11	1	1	1	1	2	3	2	0
1129	61	11	1	1	1	1	2	3	2	0
1130	43	7	2	2	1	4	3	3	2	0
1131	41	7	1	2	3	1	3	3	2	0
1132	51	9	1	2	1	1	3	3	2	0
1133	48	8	1	1	1	1	3	3	2	0
1134	61	11	1	2	1	1	3	3	2	0
1135	68	12	1	3	1	1	2	3	2	0
1136	62	11	1	2	1	1	4	3	2	0
1137	54	9	1	2	1	1	3	3	2	0
1138	58	10	2	2	1	1	2	3	2	0
1139	52	9	1	2	1	1	2	3	2	0
1140	46	8	1	2	1	1	2	3	2	0
1141	24	3	2	2	1	1	3	3	2	0
1142	58	10	1	2	1	1	3	3	2	0

1143	40	7	1	2	1	1	3	3	2	0
1144	38	6	2	2	1	1	3	3	2	0
1145	54	9	1	2	1	1	3	3	2	0
1146	65	12	1	6	1	1	4	4	2	0
1147	59	10	1	2	1	1	3	4	2	0
1148	65	12	2	2	1	4	3	4	2	0
1149	32	5	1	3	1	1	3	4	2	0
1150	35	6	2	2	1	4	3	4	2	0
1151	30	5	1	3	1	1	3	4	2	0
1152	68	12	2	2	1	4	2	4	2	0
1153	58	10	2	5	1	4	2	4	2	0
1154	38	6	1	2	1	1	3	4	2	0
1155	35	6	1	2	1	1	4	4	2	0
1156	75	14	2	6	1	4	4	4	2	0
1157	47	8	2	2	1	4	3	4	2	0
1158	65	12	2	5	1	5	2	4	2	0
1159	50	9	1	2	1	1	3	4	2	0
1160	32	5	1	2	1	1	3	4	2	0
1161	68	12	1	3	1	1	3	5	2	0
1162	17	2	1	7	1	2	3	3	2	0
1163	40	7	1	3	1	1	3	5	2	0
1164	90	15	1	5	1	1	1	5	2	0
1165	41	7	1	2	1	2	3	3	2	0
1166	101	15	2	5	3	4	1	5	2	0
1167	66	12	1	5	1	1	2	5	2	0
1168	80	15	1	3	3	1	1	5	2	0
1169	54	9	2	2	1	1	3	5	2	0
1170	74	13	2	5	1	1	2	3	2	0
1171	45	8	1	3	1	1	2	3	2	0
1172	65	12	2	5	1	1	3	3	2	0
1173	95	15	2	5	1	4	2	3	2	0

1174	20	3	1	7	1	2	3	3	2	0
1175	40	7	1	3	1	1	3	3	2	0
1176	85	15	1	3	1	1	1	3	2	0
1177	38	6	1	3	1	1	2	3	2	0
1178	46	8	1	3	1	1	3	4	2	0
1179	42	7	1	3	1	1	2	4	2	0
1180	57	10	1	2	1	1	3	4	2	0
1181	80	15	1	6	1	1	3	4	2	0
1182	60	11	2	2	1	1	2	4	2	0
1183	72	13	1	1	3	1	1	4	2	0
1184	46	8	1	3	1	1	3	4	2	0
1185	70	13	1	2	1	5	3	4	2	0
1186	48	8	1	3	1	1	3	4	2	0
1187	50	9	1	3	1	1	2	4	2	0
1188	60	11	2	5	1	4	2	4	2	0
1189	40	7	1	3	1	1	3	4	2	0
1190	52	9	1	2	1	1	3	4	2	0
1191	64	11	1	2	1	1	2	4	2	0
1192	79	14	1	5	1	1	2	4	2	0
1193	68	12	2	5	1	1	3	4	2	0
1194	44	7	1	3	1	1	3	4	2	0
1195	48	8	1	3	1	1	3	3	2	0
1196	70	13	2	5	1	4	2	5	2	0
1197	30	5	1	3	1	1	3	5	2	0
1198	39	6	1	3	1	1	3	5	2	0
1199	45	8	1	3	1	1	3	5	2	0
1200	70	13	1	3	1	1	3	5	2	0
1201	40	7	1	2	1	1	3	5	2	0
1202	58	10	2	5	1	1	2	5	2	0
1203	61	11	2	2	1	1	2	5	2	0
1204	72	13	1	2	1	1	2	5	2	0

1205	61	11	1	5	1	1	4	5	2	0
1206	50	9	1	2	1	1	3	5	2	0
1207	37	6	2	2	1	1	2	5	2	0
1208	43	7	1	2	3	1	3	5	2	0
1209	33	5	1	3	1	1	3	5	2	0
1210	32	5	1	3	1	1	3	5	2	0
1211	46	8	1	1	3	1	2	5	2	0
1212	68	12	1	3	1	1	3	5	2	0
1213	45	8	2	2	1	1	2	5	2	0
1214	45	8	1	2	1	1	3	5	2	0
1215	75	14	1	3	1	1	2	5	2	0
1216	32	5	1	3	1	1	4	5	2	0
1217	58	10	1	3	1	1	3	5	2	0
1218	75	14	1	5	1	1	3	5	2	0
1219	48	8	1	4	1	1	4	5	2	0
1220	46	8	1	3	3	1	3	5	2	0
1221	80	15	1	5	1	1	2	5	2	0
1222	60	11	1	2	1	1	4	5	2	0
1223	50	9	1	3	1	1	2	3	2	0
1224	70	13	1	5	1	1	3	3	2	0
1225	40	7	1	1	1	1	3	5	2	0
1226	78	14	2	1	1	4	2	5	2	0
1227	34	5	2	2	1	1	2	5	2	0
1228	47	8	1	3	1	1	2	5	2	0
1229	40	7	1	3	1	1	3	3	2	0
1230	62	11	1	2	1	4	3	3	2	0
1231	74	13	1	4	1	1	3	3	2	0
1232	35	6	1	3	1	1	3	3	2	0
1233	48	8	1	3	1	1	4	3	2	0
1234	75	14	1	2	3	1	2	3	2	0
1235	50	9	1	3	1	1	3	3	2	0

1236	38	6	1	2	1	1	3	3	2	0
1237	41	7	1	3	1	1	3	3	2	0
1238	54	9	1	3	1	1	3	3	2	0
1239	52	9	1	4	1	1	3	3	2	0
1240	56	10	1	3	1	1	3	3	2	0
1241	27	4	2	2	1	1	2	3	2	0
1242	41	7	1	3	1	1	3	3	2	0
1243	68	12	2	5	1	4	1	3	2	0
1244	64	11	1	3	1	1	1	3	2	0
1245	72	13	1	5	1	4	1	3	2	0
1246	44	7	2	4	1	4	3	3	2	0
1247	77	14	1	2	1	1	3	3	2	0
1248	47	8	1	2	5	1	2	3	2	0
1249	58	10	1	2	3	1	4	3	2	0
1250	62	11	2	2	1	4	2	3	2	0
1251	75	14	1	3	1	1	2	3	2	0
1252	66	12	1	1	3	1	3	3	2	0
1253	65	12	2	1	1	4	2	3	2	0
1254	37	6	2	2	1	1	3	3	2	0
1255	30	5	2	2	1	1	3	3	2	0
1256	36	6	2	2	1	1	3	3	2	0
1257	48	8	1	2	1	1	1	3	2	0
1258	60	11	2	5	1	4	1	3	2	0
1259	48	8	1	2	1	1	1	4	2	0
1260	58	10	2	4	1	3	4	4	2	0
1261	36	6	2	2	1	4	1	4	2	0
1262	48	8	2	2	1	2	2	4	2	0
1263	70	13	2	1	1	4	2	4	2	0
1264	54	9	1	3	1	1	2	4	2	0
1265	74	13	1	1	3	4	1	4	2	0
1266	46	8	1	3	1	1	2	4	2	0

1267	21	3	2	3	1	1	3	4	2	0
1268	55	10	2	4	1	1	4	4	2	0
1269	74	13	1	5	1	1	1	4	2	0
1270	70	13	1	6	1	1	3	4	2	0
1271	38	6	1	3	1	1	3	4	2	0
1272	45	8	2	2	1	1	4	4	2	0
1273	55	10	2	2	1	1	3	4	2	0
1274	45	8	1	2	1	1	3	4	2	0
1275	75	14	2	5	1	1	3	4	2	0
1276	35	6	1	3	1	1	3	3	2	0
1277	76	14	1	2	1	1	3	3	2	0
1278	27	4	1	3	1	2	3	5	2	0
1279	55	10	2	1	1	1	3	5	2	0
1280	40	7	1	2	1	1	3	5	2	0
1281	40	7	1	3	1	1	3	5	2	0
1282	21	3	1	7	1	2	3	5	2	0
1283	51	9	2	2	1	1	3	5	2	0
1284	44	7	2	2	1	1	4	5	2	0
1285	44	7	1	3	1	1	3	5	2	0
1286	45	8	1	3	3	1	3	5	2	0
1287	46	8	1	2	1	1	3	3	2	0
1288	39	6	1	2	1	1	3	3	2	0
1289	57	10	1	3	1	1	3	3	2	0
1290	47	8	1	3	1	1	3	3	2	0
1291	36	6	1	3	1	1	3	3	2	0
1292	50	9	1	3	1	1	3	5	2	0
1293	51	9	2	5	1	1	2	5	2	0
1294	35	6	2	2	1	1	3	5	2	0
1295	47	8	2	2	1	1	3	3	2	0
1296	87	15	1	3	1	1	2	3	2	0
1297	32	5	2	2	1	1	3	3	2	0

1298	36	6	1	3	1	1	3	3	2	0
1299	44	7	1	3	1	1	3	3	2	0
1300	66	12	2	2	1	1	2	3	2	0
1301	39	6	1	3	1	1	3	3	2	0
1302	70	13	1	3	1	4	2	3	2	0
1303	42	7	1	3	1	1	3	3	2	0
1304	58	10	1	3	1	1	2	3	2	0
1305	36	6	2	2	1	1	2	3	2	0
1306	45	8	1	1	1	1	3	3	2	0
1307	39	6	1	2	1	1	3	3	2	0
1308	49	8	1	3	1	1	3	3	2	0
1309	42	7	2	2	1	1	3	3	2	0
1310	63	11	1	2	1	4	2	3	2	0
1311	59	10	2	2	1	1	2	3	2	0
1312	76	14	1	2	1	1	2	3	2	0
1313	35	6	1	3	1	1	3	3	2	0
1314	40	7	2	2	1	4	2	3	2	0
1315	34	5	1	3	1	1	3	3	2	0
1316	35	6	1	3	1	1	3	3	2	0
1317	60	11	1	1	1	1	2	3	2	0
1318	42	7	1	2	1	1	3	3	2	0
1319	67	12	1	1	1	1	2	3	2	0
1320	43	7	2	2	1	3	2	3	2	0
1321	65	12	1	2	1	1	2	3	2	0
1322	30	5	2	2	1	1	3	3	2	0
1323	68	12	1	1	1	1	2	3	2	0
1324	26	4	1	2	1	2	3	3	2	0
1325	43	7	1	2	1	1	2	3	2	0
1326	75	14	1	2	1	1	1	3	2	0
1327	39	6	2	2	1	1	2	3	2	0
1328	60	11	1	1	1	1	2	3	2	0

1329	74	13	1	2	1	1	2	3	2	0
1330	60	11	2	2	1	1	2	3	2	0
1331	57	10	1	2	1	1	2	3	2	0
1332	49	8	2	2	1	1	3	3	2	0
1333	77	14	1	2	1	1	2	3	2	0
1334	43	7	1	2	1	1	3	3	2	0
1335	51	9	2	2	1	4	2	3	2	0
1336	85	15	1	3	1	1	2	3	2	0
1337	47	8	1	3	1	1	3	3	2	0
1338	79	14	1	3	1	1	2	3	2	0
1339	69	12	1	2	1	1	2	3	2	0
1340	62	11	1	3	1	4	2	3	2	0
1341	54	9	1	2	1	1	2	3	2	0
1342	47	8	1	2	1	4	3	3	2	0
1343	72	13	1	2	1	1	2	3	2	0
1344	66	12	1	2	1	1	2	3	2	0
1345	57	10	2	2	1	1	2	3	2	0
1346	86	15	1	1	1	4	2	3	2	0
1347	49	8	1	2	1	1	3	3	2	0
1348	35	6	1	2	1	1	3	3	2	0
1349	12	1	1	7	1	2	2	3	2	0
1350	58	10	1	3	1	1	2	3	2	0
1351	50	9	1	2	1	1	2	3	2	0
1352	65	12	1	3	1	1	2	3	2	0
1353	15	2	1	7	1	2	3	3	2	0
1354	58	10	2	3	1	1	3	6	2	0
1355	60	11	1	2	1	1	4	3	2	0
1356	66	12	1	2	1	1	2	3	2	0
1357	64	11	1	4	1	1	2	6	2	0
1358	48	8	1	2	1	1	3	3	2	0
1359	60	11	1	1	1	1	2	5	2	0

1360	32	5	2	2	1	1	3	3	2	0
1361	59	10	1	2	1	1	2	3	2	0
1362	49	8	1	2	1	1	2	3	2	0
1363	56	10	2	2	1	1	2	3	2	0
1364	59	10	1	2	1	1	3	3	2	0
1365	30	5	2	3	1	1	3	3	2	0
1366	51	9	2	2	1	1	2	3	2	0
1367	56	10	1	2	1	1	2	3	2	0
1368	54	9	1	2	1	1	3	3	2	0
1369	50	9	2	2	1	4	3	3	2	0
1370	41	7	1	2	1	1	3	3	2	0
1371	61	11	1	2	1	1	2	3	2	0
1372	36	6	1	2	1	1	3	3	2	0
1373	60	11	1	1	1	1	2	3	2	0
1374	60	11	1	1	1	1	3	3	2	0
1375	41	7	2	1	1	4	3	6	2	0
1376	45	8	2	2	1	1	2	3	2	0
1377	69	12	2	1	1	4	2	3	2	0
1378	30	5	2	3	1	4	3	3	2	0
1379	46	8	2	2	1	4	3	3	2	0
1380	36	6	1	2	1	1	3	3	2	0
1381	40	7	1	2	1	1	2	3	2	0
1382	38	6	1	2	1	1	3	3	2	0
1383	43	7	1	2	1	1	3	3	2	0
1384	72	13	1	2	1	1	2	3	2	0
1385	40	7	1	3	1	1	3	3	2	0
1386	69	12	1	2	1	1	3	3	2	0
1387	68	12	2	1	1	3	2	3	2	0
1388	70	13	2	2	1	4	2	3	2	0
1389	58	10	1	2	1	1	3	3	2	0
1390	39	6	1	2	1	4	3	3	2	0

1391	61	11	1	3	1	1	2	3	2	0
1392	58	10	2	2	1	1	2	3	2	0
1393	40	7	1	3	1	1	3	3	2	0
1394	47	8	1	2	1	1	3	3	2	0
1395	80	15	1	2	1	1	3	3	2	0
1396	48	8	2	1	1	4	2	3	2	0
1397	70	13	1	2	1	1	2	3	2	0
1398	30	5	1	2	1	1	3	3	2	0
1399	35	6	1	3	1	4	2	3	2	0
1400	50	9	2	2	1	1	3	3	2	0
1401	50	9	1	2	1	1	3	3	2	0
1402	65	12	1	3	1	1	2	3	2	0
1403	60	11	1	3	1	1	3	3	2	0
1404	52	9	2	3	1	1	3	3	2	0
1405	70	13	1	5	1	1	2	3	2	0
1406	35	6	1	2	1	1	3	3	2	0
1407	40	7	1	2	1	1	3	3	2	0
1408	70	13	1	2	1	1	3	3	2	0
1409	35	6	1	3	1	1	3	3	2	0
1410	65	12	1	3	1	1	3	3	2	0
1411	62	11	1	2	1	1	3	3	2	0
1412	68	12	1	2	1	1	3	3	2	0
1413	52	9	1	2	1	1	3	3	2	0
1414	78	14	2	5	1	4	1	3	2	0
1415	48	8	1	2	1	1	3	3	2	0
1416	80	15	2	5	1	4	2	3	2	0
1417	62	11	1	2	1	1	2	3	2	0
1418	66	12	1	2	1	1	2	3	2	0
1419	52	9	1	2	1	1	2	3	2	0
1420	55	10	1	1	1	1	2	3	2	0
1421	45	8	2	2	1	4	3	3	2	0

1422	67	12	1	2	1	4	2	3	2	0
1423	63	11	2	1	1	1	1	3	2	0
1424	39	6	1	2	1	2	2	3	2	0
1425	79	14	1	2	1	1	1	3	2	0
1426	62	11	1	1	1	1	1	3	2	0
1427	51	9	1	2	1	1	1	3	2	0
1428	31	5	1	2	1	1	2	3	2	0
1429	40	7	1	2	1	1	4	3	2	0
1430	47	8	2	2	1	4	1	3	2	0
1431	56	10	2	2	1	4	1	3	2	0
1432	36	6	2	2	1	4	3	3	2	0
1433	39	6	1	2	1	1	2	3	2	0
1434	59	10	1	1	1	4	3	3	2	0
1435	71	13	1	1	1	1	1	3	2	0
1436	65	12	1	2	1	1	2	3	2	0
1437	36	6	1	1	1	1	1	3	2	0
1438	41	7	1	1	1	1	1	3	2	0
1439	31	5	1	2	1	1	2	3	2	0
1440	45	8	1	2	1	1	3	3	2	0
1441	66	12	2	2	1	4	2	3	2	0
1442	36	6	1	2	1	2	2	3	2	0
1443	39	6	1	3	3	2	2	6	2	0
1444	30	5	1	2	1	1	4	6	2	0
1445	65	12	1	3	1	1	1	3	2	0
1446	51	9	1	2	1	1	2	3	2	0
1447	28	4	1	3	1	2	2	3	2	0
1448	40	7	1	2	1	1	3	3	2	0
1449	20	3	1	7	1	2	3	3	2	0
1450	48	8	2	2	1	1	1	3	2	0
1451	40	7	2	1	1	4	1	3	2	0
1452	61	11	2	5	1	4	1	3	2	0

1453	39	6	2	1	1	1	2	3	2	0
1454	61	11	1	1	1	4	3	3	2	0
1455	63	11	2	1	1	4	1	3	2	0
1456	41	7	1	2	1	1	2	3	2	0
1457	44	7	1	4	1	1	2	3	2	0
1458	36	6	1	2	1	1	2	3	2	0
1459	30	5	1	3	2	1	1	3	2	0
1460	50	9	2	1	1	1	1	3	2	0
1461	65	12	2	1	1	4	1	3	2	0
1462	60	11	1	2	1	1	3	3	2	0
1463	32	5	1	2	1	2	3	3	2	0
1464	45	8	1	2	1	1	2	3	2	0
1465	46	8	2	1	1	1	3	3	2	0
1466	56	10	1	1	3	1	1	3	2	0
1467	44	7	2	2	1	1	1	3	2	0
1468	65	12	1	1	1	1	1	3	2	0
1469	69	12	2	1	1	4	1	3	2	0
1470	70	13	2	1	1	4	1	3	2	0
1471	92	15	2	5	1	4	1	5	2	0
1472	51	9	1	1	1	1	1	5	2	0
1473	60	11	2	1	1	2	1	5	2	0
1474	59	10	2	2	1	1	2	5	2	0
1475	44	7	1	2	1	1	2	5	2	0
1476	56	10	1	1	1	1	1	5	2	0
1477	60	11	1	3	1	1	3	5	2	0
1478	49	8	2	1	1	1	1	5	2	0
1479	61	11	1	2	1	1	2	5	2	0
1480	49	8	2	2	1	1	2	5	2	0
1481	36	6	1	2	1	1	2	5	2	0
1482	43	7	2	2	1	1	1	5	2	0
1483	50	9	1	1	1	4	1	5	2	0

1484	61	11	1	2	1	1	2	5	2	0
1485	54	9	1	2	3	1	2	5	2	0
1486	50	9	1	1	1	1	2	5	2	0
1487	36	6	2	3	1	1	2	5	2	0
1488	40	7	1	2	1	1	2	5	2	0
1489	45	8	1	1	1	1	1	5	2	0
1490	30	5	1	3	1	1	2	3	2	0
1491	32	5	1	3	1	1	2	3	2	0
1492	74	13	1	3	1	1	3	3	2	0
1493	49	8	1	2	1	1	3	3	2	0
1494	78	14	1	1	1	1	2	3	2	0
1495	79	14	1	3	3	1	2	3	2	0
1496	58	10	1	2	1	1	3	3	2	0
1497	65	12	1	1	1	1	2	3	2	0
1498	68	12	1	3	1	1	2	3	2	0
1499	40	7	1	2	1	1	3	3	2	0
1500	62	11	1	3	1	1	2	3	2	0
1501	65	12	1	2	1	1	2	3	2	0
1502	76	14	1	1	1	1	2	3	2	0
1503	70	13	1	1	1	1	3	3	2	0
1504	56	10	1	2	1	1	3	3	2	0
1505	68	12	1	2	1	1	2	3	2	0
1506	80	15	1	1	1	1	2	3	2	0
1507	40	7	1	3	1	1	3	3	2	0
1508	35	6	1	3	1	1	2	3	2	0
1509	79	14	1	1	1	1	2	3	2	0
1510	39	6	1	2	1	1	3	3	2	0
1511	60	11	1	1	1	1	3	3	2	0
1512	64	11	1	6	1	1	2	3	2	0
1513	68	12	1	3	1	1	2	3	2	0
1514	46	8	1	2	1	1	3	3	2	0

1515	55	10	1	3	1	1	2	3	2	0
1516	40	7	1	8	1	1	3	3	2	0
1517	56	10	1	2	1	1	3	3	2	0
1518	79	14	1	6	1	1	2	3	2	0
1519	60	11	1	3	1	1	4	3	2	0
1520	68	12	1	8	1	1	3	3	2	0
1521	69	12	1	2	1	1	2	3	2	0
1522	56	10	1	3	1	1	2	3	2	0
1523	56	10	1	4	1	1	4	3	2	0
1524	56	10	1	2	1	1	3	3	2	0
1525	54	9	1	2	1	1	2	3	2	0
1526	55	10	1	4	1	1	4	3	2	0
1527	68	12	1	2	1	1	2	3	2	0
1528	70	13	1	6	1	1	4	3	2	0
1529	41	7	1	3	1	1	2	3	2	0
1530	57	10	1	2	1	1	2	3	2	0
1531	56	10	1	2	1	1	2	3	2	0
1532	52	9	2	2	1	1	3	3	2	0
1533	50	9	1	8	1	1	3	3	2	0
1534	69	12	1	8	3	1	2	3	2	0
1535	79	14	1	2	1	1	3	3	2	0
1536	40	7	2	2	1	1	4	3	2	0
1537	68	12	1	1	1	1	3	3	2	0
1538	40	7	1	2	1	1	3	3	2	0
1539	79	14	1	5	1	1	4	3	2	0
1540	39	6	1	2	1	1	2	3	2	0
1541	68	12	1	4	1	1	4	3	2	0
1542	35	6	2	2	1	1	2	3	2	0
1543	70	13	1	6	1	1	4	3	2	0
1544	80	15	1	6	1	1	4	3	2	0
1545	60	11	1	8	1	2	4	3	2	0

1546	70	13	1	3	1	1	3	3	2	0
1547	66	12	1	6	1	1	4	3	2	0
1548	70	13	1	4	1	1	4	3	2	0
1549	74	13	1	6	1	1	4	3	2	0
1550	39	6	2	3	1	1	3	3	2	0
1551	61	11	2	3	1	1	2	3	2	0
1552	42	7	2	2	1	1	2	3	2	0
1553	48	8	1	2	1	1	3	3	2	0
1554	38	6	1	8	1	1	4	3	2	0
1555	72	13	1	1	1	1	2	3	2	0
1556	71	13	1	3	1	1	2	3	2	0
1557	80	15	1	2	1	1	2	3	2	0
1558	40	7	2	2	1	1	3	3	2	0
1559	70	13	1	3	1	1	2	3	2	0
1560	28	4	2	3	1	2	3	5	2	0
1561	78	14	1	6	1	1	4	5	2	0
1562	70	13	2	6	1	1	3	5	2	0
1563	60	11	1	5	1	1	2	5	2	0
1564	80	15	2	1	1	1	2	5	2	0
1565	30	5	2	4	1	1	4	5	2	0
1566	80	15	2	3	1	1	3	5	2	0
1567	45	8	1	4	1	1	3	5	2	0
1568	56	10	1	3	1	1	3	5	2	0
1569	38	6	1	4	1	1	3	5	2	0
1570	80	15	1	6	1	1	3	5	2	0
1571	79	14	1	6	1	1	4	5	2	0
1572	70	13	1	5	1	1	3	5	2	0
1573	30	5	2	2	1	1	4	5	2	0
1574	38	6	2	1	1	1	4	5	2	0
1575	39	6	1	4	1	1	3	5	2	0
1576	49	8	2	2	1	4	2	5	2	0

1577	38	6	2	2	1	1	3	5	2	0
1578	65	12	2	2	1	4	2	5	2	0
1579	46	8	1	3	1	1	3	5	2	0
1580	42	7	1	3	1	1	3	5	2	0
1581	38	6	1	3	1	2	3	5	2	0
1582	42	7	2	2	1	4	3	5	2	0
1583	33	5	1	3	1	2	3	5	2	0
1584	39	6	1	3	1	1	2	5	2	0
1585	64	11	2	6	1	4	2	5	2	0
1586	45	8	1	2	1	1	3	5	2	0
1587	42	7	1	5	1	2	3	5	2	0
1588	58	10	1	2	1	1	3	5	2	0
1589	38	6	2	2	1	4	3	5	2	0
1590	50	9	2	1	1	1	3	5	2	0
1591	43	7	2	2	1	4	3	5	2	0
1592	48	8	2	1	1	4	3	5	2	0
1593	50	9	2	1	1	4	2	5	2	0
1594	49	8	2	2	1	4	2	5	2	0
1595	68	12	2	1	1	4	1	5	2	0
1596	32	5	2	3	1	1	3	5	2	0
1597	48	8	2	1	1	4	1	5	2	0
1598	35	6	1	4	1	1	4	5	2	0
1599	71	13	2	6	1	5	4	5	2	0
1600	39	6	1	3	1	1	3	5	2	0
1601	51	9	1	3	1	1	3	5	2	0
1602	46	8	2	1	1	4	3	5	2	0
1603	58	10	1	6	1	1	3	5	2	0
1604	64	11	1	2	1	1	2	6	2	0
1605	52	9	2	2	1	1	3	6	2	0
1606	40	7	1	2	4	5	3	5	2	0
1607	28	4	2	2	1	1	3	5	2	0

1608	32	5	2	8	1	1	4	5	2	0
1609	60	11	1	1	1	1	2	5	2	0
1610	29	4	2	8	1	1	3	5	2	0
1611	67	12	2	2	1	1	3	5	2	0
1612	78	14	2	6	1	4	4	5	2	0
1613	54	9	1	2	1	1	3	5	2	0
1614	45	8	1	2	1	1	3	5	2	0
1615	40	7	1	2	1	1	3	5	2	0
1616	45	8	1	2	1	1	2	5	2	0
1617	35	6	1	3	1	1	3	5	2	0
1618	50	9	2	2	1	1	3	5	2	0
1619	58	10	1	2	1	1	3	5	2	0
1620	50	9	1	3	1	1	2	5	2	0
1621	60	11	2	5	1	4	2	5	2	0
1622	55	10	2	4	1	2	4	5	2	0
1623	70	13	1	1	1	1	2	4	2	0
1624	88	15	1	6	1	4	4	5	2	0
1625	50	9	2	2	1	1	1	5	2	0
1626	43	7	2	2	1	1	3	5	2	0
1627	52	9	1	2	1	1	4	5	2	0
1628	56	10	1	3	1	1	2	5	2	0
1629	52	9	2	2	1	4	2	5	2	0
1630	40	7	1	3	1	1	2	5	2	0
1631	60	11	2	1	1	4	2	5	2	0
1632	50	9	1	3	1	1	4	5	2	0
1633	48	8	1	3	1	1	3	5	2	0
1634	50	9	1	3	1	1	3	5	2	0
1635	55	10	2	1	1	4	1	5	2	0
1636	70	13	1	6	1	1	4	5	2	0
1637	63	11	2	5	1	1	3	5	2	0
1638	53	9	1	3	1	1	3	5	2	0

1639	65	12	2	2	1	1	2	5	2	0
1640	65	12	2	1	1	4	2	5	2	0
1641	45	8	2	2	1	1	3	5	2	0
1642	35	6	2	3	1	1	3	5	2	0
1643	56	10	1	3	1	1	3	5	2	0
1644	42	7	2	2	1	1	3	5	2	0
1645	56	10	2	2	1	1	3	5	2	0
1646	60	11	2	1	1	4	1	5	2	0
1647	58	10	1	3	1	1	3	5	2	0
1648	74	13	1	1	1	4	2	5	2	0
1649	50	9	1	3	1	1	2	5	2	0
1650	42	7	1	2	1	1	3	5	2	0
1651	71	13	2	6	1	4	4	5	2	0
1652	39	6	1	2	1	1	3	5	2	0
1653	68	12	1	2	1	1	2	5	2	0
1654	67	12	1	3	1	1	2	5	2	0
1655	45	8	2	3	1	1	2	5	2	0
1656	72	13	1	2	1	4	2	5	2	0
1657	55	10	2	2	1	1	2	5	2	0
1658	40	7	1	4	1	1	4	5	2	0
1659	59	10	1	3	1	1	3	5	2	0
1660	59	10	1	2	1	1	2	5	2	0
1661	51	9	1	2	1	1	2	5	2	0
1662	44	7	2	2	1	1	3	5	2	0
1663	40	7	2	1	1	1	2	5	2	0
1664	53	9	1	3	1	1	3	5	2	0
1665	47	8	2	2	1	1	2	5	2	0
1666	70	13	2	5	1	4	2	5	2	0
1667	61	11	1	2	1	1	2	5	2	0
1668	75	14	2	5	1	4	1	5	2	0
1669	50	9	2	2	1	1	3	5	2	0

1670	47	8	1	2	3	5	2	5	2	0
1671	56	10	1	1	1	1	1	5	2	0
1672	78	14	1	5	1	1	1	5	2	0
1673	57	10	1	2	1	1	2	5	2	0
1674	82	15	1	2	1	1	1	5	2	0
1675	69	12	1	1	1	1	1	5	2	0
1676	74	13	2	5	1	1	2	5	2	0
1677	60	11	1	2	1	1	2	5	2	0
1678	76	14	1	2	1	1	1	5	2	0
1679	48	8	1	2	1	1	2	5	2	0
1680	86	15	1	5	1	1	1	5	2	0
1681	58	10	1	2	1	1	3	5	2	0
1682	68	12	1	5	1	1	1	5	2	0
1683	58	10	2	1	1	1	1	5	2	0
1684	28	4	1	8	1	2	3	5	2	0
1685	58	10	2	2	1	4	2	5	2	0
1686	50	9	1	2	1	1	2	5	2	0
1687	40	7	1	3	1	1	2	5	2	0
1688	35	6	1	3	1	1	2	5	2	0
1689	32	5	1	3	1	1	2	5	2	0
1690	30	5	1	2	1	1	3	5	2	0
1691	26	4	2	2	1	1	3	6	2	0
1692	33	5	1	3	1	1	2	6	2	0
1693	65	12	2	1	1	1	2	5	2	0
1694	40	7	2	2	1	1	3	5	2	0
1695	30	5	2	2	1	1	2	5	2	0
1696	70	13	1	5	1	1	3	5	2	0
1697	50	9	1	2	1	1	3	5	2	0
1698	40	7	1	3	1	1	3	5	2	0
1699	48	8	1	1	1	1	2	5	2	0
1700	54	9	2	1	1	1	1	5	2	0

1701	58	10	2	2	1	4	2	5	2	0
1702	68	12	1	3	1	4	2	5	2	0
1703	65	12	1	3	1	1	3	5	2	0
1704	20	3	1	7	1	2	3	5	2	0
1705	50	9	2	1	1	4	2	5	2	0
1706	45	8	1	3	1	1	3	5	2	0
1707	48	8	1	2	1	1	2	5	2	0
1708	77	14	1	1	1	4	2	5	2	0
1709	64	11	1	5	1	1	3	5	2	0
1710	65	12	2	2	1	4	2	5	2	0
1711	70	13	2	6	1	1	2	5	2	0
1712	50	9	2	2	1	1	3	5	2	0
1713	70	13	1	1	1	1	2	5	2	0
1714	75	14	1	5	1	1	2	5	2	0
1715	50	9	1	3	3	1	2	5	2	0
1716	66	12	2	2	1	1	3	5	2	0
1717	53	9	1	1	1	1	2	5	2	0
1718	83	15	2	5	1	4	1	5	2	0
1719	60	11	1	3	1	1	3	5	2	0
1720	74	13	2	6	1	4	2	5	2	0
1721	65	12	2	2	1	1	2	5	2	0
1722	80	15	2	1	1	4	1	5	2	0
1723	58	10	2	4	1	4	3	5	2	0
1724	70	13	2	5	1	4	3	5	2	0
1725	75	14	2	5	1	4	1	5	2	0
1726	72	13	1	3	1	1	1	5	2	0
1727	58	10	1	2	1	1	3	5	2	0
1728	60	11	1	2	1	1	2	5	2	0
1729	62	11	2	2	1	1	2	5	2	0
1730	40	7	2	2	1	1	3	5	2	0
1731	50	9	1	3	1	1	3	5	2	0

1732	20	3	2	7	1	2	3	5	2	0
1733	73	13	2	5	1	4	2	5	2	0
1734	78	14	1	5	1	1	2	5	2	0
1735	68	12	2	2	1	1	2	5	2	0
1736	57	10	2	2	1	1	1	5	2	0
1737	55	10	2	1	1	1	2	5	2	0
1738	45	8	1	3	1	1	2	5	2	0
1739	50	9	1	2	1	1	2	5	2	0
1740	52	9	2	2	1	1	3	5	2	0
1741	55	10	1	3	1	1	2	5	2	0
1742	59	10	2	5	1	3	2	5	2	0
1743	75	14	2	5	3	1	1	5	2	0
1744	55	10	1	3	1	1	1	5	2	0
1745	71	13	2	5	1	1	2	5	2	0
1746	59	10	1	2	1	1	2	5	2	0
1747	75	14	1	2	1	1	2	5	2	0
1748	32	5	2	4	1	1	3	5	2	0
1749	70	13	1	6	1	1	4	5	2	0
1750	75	14	2	1	1	4	2	5	2	0
1751	38	6	1	4	1	1	4	5	2	0
1752	65	12	2	3	1	1	2	5	2	0
1753	60	11	2	2	1	1	2	5	2	0
1754	72	13	1	1	1	1	2	5	2	0
1755	65	12	2	2	1	1	2	5	2	0
1756	74	13	1	2	1	1	3	5	2	0
1757	62	11	1	3	1	4	2	5	2	0
1758	48	8	1	2	1	1	3	5	2	0
1759	53	9	2	3	1	1	2	5	2	0
1760	65	12	1	2	1	1	3	5	2	0
1761	65	12	1	5	1	1	2	5	2	0
1762	38	6	2	2	1	4	2	5	2	0

1763	38	6	2	2	1	1	4	5	2	0
1764	48	8	1	1	1	1	2	5	2	0
1765	42	7	1	3	1	1	3	5	2	0
1766	24	3	2	7	1	2	3	5	2	0
1767	27	4	1	7	1	2	3	5	2	0
1768	30	5	2	2	1	2	3	5	2	0
1769	55	10	1	3	1	1	2	5	2	0
1770	54	9	1	2	1	1	3	5	2	0
1771	40	7	2	2	1	1	3	5	2	0
1772	54	9	2	2	1	1	3	5	2	0
1773	41	7	1	3	1	1	2	5	2	0
1774	72	13	1	2	1	1	3	5	2	0
1775	80	15	2	1	1	4	2	5	2	0
1776	74	13	1	3	1	1	3	5	2	0
1777	62	11	1	2	1	1	3	5	2	0
1778	42	7	2	1	1	1	2	5	2	0
1779	60	11	1	3	1	1	2	5	2	0
1780	35	6	2	2	1	1	3	5	2	0
1781	54	9	1	3	1	1	3	5	2	0
1782	56	10	2	2	1	1	2	5	2	0
1783	65	12	1	3	1	1	3	5	2	0
1784	60	11	1	3	3	1	2	5	2	0
1785	72	13	1	1	1	1	3	5	2	0
1786	85	15	1	3	1	1	2	5	2	0
1787	65	12	2	2	1	1	2	5	2	0
1788	49	8	2	2	1	1	2	5	2	0
1789	45	8	1	4	1	1	4	5	2	0
1790	49	8	1	2	1	1	2	5	2	0
1791	63	11	1	3	1	1	3	5	2	0
1792	62	11	1	3	1	1	2	5	2	0
1793	80	15	2	1	1	4	2	5	2	0

1794	45	8	2	2	1	1	3	5	2	0
1795	54	9	1	4	1	1	3	5	2	0
1796	49	8	1	3	1	1	3	5	2	0
1797	45	8	1	2	1	1	3	5	2	0
1798	67	12	2	1	1	1	2	5	2	0
1799	63	11	2	2	1	1	2	5	2	0
1800	35	6	1	4	1	2	3	5	2	0
1801	65	12	2	1	1	1	2	5	2	0
1802	60	11	1	5	1	1	2	5	2	0
1803	56	10	2	4	1	1	4	5	2	0
1804	52	9	1	2	1	1	2	5	2	0
1805	41	7	1	2	1	1	3	5	2	0
1806	37	6	1	2	1	1	4	5	2	0
1807	51	9	1	3	1	1	2	5	2	0
1808	73	13	2	2	1	4	1	5	2	0
1809	30	5	1	2	1	1	3	5	2	0
1810	62	11	2	2	1	1	3	5	2	0
1811	66	12	1	4	1	1	3	5	2	0
1812	48	8	1	4	1	1	3	5	2	0
1813	57	10	2	2	1	1	2	5	2	0
1814	58	10	2	2	1	1	3	5	2	0
1815	62	11	1	1	1	1	2	5	2	0
1816	55	10	2	4	1	1	2	5	2	0
1817	57	10	1	3	1	1	2	5	2	0
1818	34	5	1	1	1	1	3	5	2	0
1819	59	10	2	1	1	1	2	2	2	0
1820	59	10	2	2	1	1	2	2	2	0
1821	24	3	1	3	1	2	3	2	2	0
1822	34	5	1	3	1	1	2	4	2	0
1823	26	4	2	2	1	1	2	4	2	0
1824	62	11	2	1	1	1	2	2	2	0

1825	46	8	2	1	1	1	3	2	1	0
1826	62	11	2	5	1	1	2	2	2	0
1827	43	7	1	3	1	1	2	2	1	2
1828	45	8	1	2	1	1	2	2	2	0
1829	41	7	1	2	1	1	3	2	2	0
1830	35	6	2	1	1	1	3	2	1	0
1831	72	13	2	5	1	1	1	2	2	0
1832	70	13	2	1	1	1	2	2	2	0
1833	65	12	1	3	1	1	2	2	2	0
1834	40	7	1	2	1	1	3	2	2	0
1835	55	10	1	2	1	1	2	2	2	0
1836	33	5	1	3	1	1	2	2	2	0
1837	70	13	2	5	1	1	2	2	2	0
1838	66	12	1	1	1	1	1	2	2	0
1839	45	8	1	1	3	1	2	2	2	0
1840	63	11	2	1	1	1	2	2	2	0
1841	40	7	1	3	1	1	2	2	2	0
1842	36	6	1	2	1	1	3	2	2	0
1843	79	14	1	5	1	1	2	2	2	0
1844	50	9	1	3	1	1	2	2	2	0
1845	45	8	2	3	1	1	2	2	2	0
1846	36	6	1	1	1	1	1	2	2	0
1847	60	11	2	2	1	1	3	2	2	0
1848	71	13	1	3	1	1	2	2	2	0
1849	20	3	1	7	1	2	3	2	2	0
1850	45	8	1	3	1	1	3	2	2	0
1851	60	11	1	3	1	1	3	2	2	0
1852	69	12	2	1	1	1	2	2	2	0
1853	30	5	1	3	1	1	2	2	2	0
1854	46	8	1	3	1	1	3	2	2	0
1855	48	8	1	4	1	1	4	2	2	0

1856	89	15	1	6	1	1	2	2	2	0
1857	36	6	1	2	1	1	3	2	2	0
1858	56	10	2	4	1	1	3	2	2	0
1859	36	6	1	3	1	1	3	6	1	0
1860	25	4	2	3	1	1	3	6	1	0
1861	80	15	1	2	1	1	2	2	2	0
1862	50	9	2	2	1	1	2	2	2	0
1863	70	13	2	1	1	1	2	2	2	0
1864	45	8	1	3	1	1	2	2	2	0
1865	72	13	1	3	1	1	2	2	2	0
1866	82	15	1	5	1	4	2	2	2	0
1867	43	7	2	4	1	1	4	2	2	0
1868	60	11	2	5	1	1	2	2	2	0
1869	68	12	1	1	1	1	2	2	2	0
1870	47	8	1	3	1	1	3	2	2	0
1871	64	11	1	6	1	1	4	2	2	0
1872	38	6	1	2	1	1	3	2	2	0
1873	57	10	1	3	1	1	2	2	2	0
1874	67	12	2	1	1	1	2	2	2	0
1875	72	13	2	5	1	1	4	2	2	0
1876	77	14	2	5	1	4	3	2	2	0
1877	46	8	1	3	1	1	2	2	2	0
1878	26	4	1	7	1	2	4	2	2	0
1879	68	12	1	3	1	1	3	2	2	0
1880	40	7	1	3	1	1	3	2	2	0
1881	28	4	2	2	1	1	3	2	2	0
1882	78	14	1	2	1	1	2	2	2	0
1883	67	12	2	2	1	1	2	2	2	0
1884	41	7	1	3	1	1	3	2	1	1
1885	73	13	2	1	1	1	1	2	2	0
1886	25	4	1	2	1	1	3	2	2	0

1887	82	15	2	1	1	1	2	2	2	0
1888	39	6	1	2	1	1	3	2	2	0
1889	40	7	1	2	1	1	3	2	2	0
1890	35	6	2	2	1	1	3	2	2	0
1891	60	11	2	2	1	4	2	2	2	0
1892	65	12	2	5	3	1	3	2	2	0
1893	65	12	2	2	1	1	3	2	2	0
1894	65	12	1	2	1	1	2	2	2	0
1895	60	11	1	2	1	1	2	2	2	0
1896	48	8	2	1	1	1	2	2	2	0
1897	50	9	1	2	1	1	3	2	2	0
1898	50	9	2	1	1	1	3	2	2	0
1899	53	9	2	1	1	1	2	2	2	0
1900	60	11	2	2	1	1	3	2	2	0
1901	49	8	1	2	1	1	3	2	2	0
1902	33	5	2	2	1	1	3	2	2	0
1903	38	6	1	2	1	1	3	2	2	0
1904	27	4	2	2	1	1	2	2	2	0
1905	51	9	1	2	1	1	2	2	2	0
1906	85	15	1	6	1	4	4	2	2	0
1907	67	12	1	2	1	1	3	2	2	0
1908	23	3	1	2	1	2	2	2	2	0
1909	35	6	1	2	1	1	3	2	2	0
1910	55	10	2	1	1	1	2	2	2	0
1911	45	8	1	2	1	1	2	2	2	0
1912	40	7	2	1	1	1	2	2	2	0
1913	33	5	1	2	1	1	2	2	2	0
1914	54	9	1	2	1	1	2	2	2	0
1915	50	9	2	1	1	1	2	2	2	0
1916	61	11	1	2	1	1	3	2	2	0
1917	69	12	1	2	1	1	2	2	2	0

1918	81	15	1	6	1	1	4	2	2	0
1919	53	9	1	2	1	1	4	2	2	0
1920	50	9	2	2	1	1	2	2	2	0
1921	26	4	2	2	1	1	3	2	2	0
1922	58	10	1	2	1	1	2	4	2	0
1923	52	9	2	2	1	1	2	4	2	0
1924	50	9	1	1	1	1	2	2	2	0
1925	33	5	2	2	1	1	3	4	2	0
1926	42	7	1	2	1	1	2	4	2	0
1927	49	8	2	2	1	1	3	2	2	0
1928	70	13	1	6	1	1	4	2	2	0
1929	47	8	2	2	1	1	3	2	2	0
1930	62	11	1	2	1	1	2	2	2	0
1931	50	9	1	3	1	1	3	2	2	0
1932	49	8	1	3	1	1	3	2	2	0
1933	32	5	1	3	1	1	3	2	2	0
1934	49	8	2	2	1	1	2	2	2	0
1935	50	9	2	2	1	1	2	2	2	0
1936	64	11	1	1	1	1	2	2	2	0
1937	15	2	1	7	1	2	3	2	2	0
1938	70	13	2	1	1	1	3	2	2	0
1939	51	9	1	2	1	1	2	2	2	0
1940	52	9	1	2	1	1	3	2	2	0
1941	72	13	1	3	1	1	2	2	2	0
1942	46	8	2	3	1	1	3	2	2	0
1943	63	11	2	4	1	4	3	2	2	0
1944	45	8	2	2	1	1	2	2	2	0
1945	43	7	1	3	1	1	3	2	2	0
1946	30	5	2	1	1	1	3	2	2	0
1947	35	6	2	2	1	1	3	2	2	0
1948	36	6	2	4	1	1	4	2	2	0

1949	50	9	1	1	1	1	3	2	2	0
1950	49	8	1	3	1	1	3	2	2	0
1951	70	13	2	5	1	4	3	2	2	0
1952	44	7	2	2	1	1	3	2	2	0
1953	39	6	1	3	1	1	2	6	1	0
1954	55	10	1	2	1	4	2	2	2	0
1955	35	6	1	3	1	1	3	2	2	0
1956	32	5	2	2	1	1	3	6	2	0
1957	32	5	2	2	1	2	3	2	2	0
1958	50	9	2	2	1	1	3	2	2	0
1959	28	4	2	3	1	1	3	2	2	0
1960	45	8	1	2	1	1	2	2	2	0
1961	43	7	2	1	1	1	1	2	2	0
1962	67	12	2	1	1	1	2	2	2	0
1963	43	7	1	3	1	1	3	2	2	0
1964	57	10	2	2	1	4	2	2	2	0
1965	22	3	1	3	1	2	3	2	2	0
1966	30	5	2	2	1	1	3	2	2	0
1967	50	9	1	3	1	1	3	2	2	0
1968	43	7	1	3	1	1	3	2	2	0
1969	53	9	1	3	1	1	3	2	2	0
1970	42	7	2	3	1	1	2	2	2	0
1971	37	6	2	2	1	1	2	2	2	0
1972	35	6	1	2	1	1	3	2	2	0
1973	24	3	2	2	1	1	3	2	2	0
1974	60	11	1	3	1	1	3	2	2	0
1975	63	11	1	6	1	1	3	2	2	0
1976	82	15	1	5	1	4	2	2	2	0
1977	64	11	1	1	1	1	1	2	2	0
1978	65	12	2	1	1	1	2	2	2	0
1979	78	14	1	6	1	4	2	2	2	0

1980	60	11	2	3	1	1	2	2	2	0
1981	35	6	1	3	1	1	2	2	2	0
1982	23	3	2	2	1	1	2	2	2	0
1983	50	9	1	1	1	1	3	2	2	0
1984	28	4	1	2	1	2	3	2	2	0
1985	37	6	2	2	1	1	2	2	2	0
1986	49	8	1	3	1	1	2	2	2	0
1987	43	7	2	1	1	1	2	2	2	0
1988	72	13	1	1	1	1	2	2	2	0
1989	60	11	2	1	1	4	2	2	2	0
1990	65	12	2	4	1	1	3	2	2	0
1991	43	7	2	2	1	1	3	2	2	0
1992	33	5	2	2	1	4	3	2	2	0
1993	22	3	1	3	1	2	3	2	2	0
1994	67	12	1	3	3	1	2	2	2	0
1995	56	10	2	2	1	4	2	2	2	0
1996	71	13	1	1	1	1	2	2	2	0
1997	42	7	1	3	1	4	3	2	2	0
1998	46	8	1	2	1	1	2	2	2	0
1999	58	10	1	3	1	1	2	2	2	0
2000	52	9	2	1	1	4	2	2	2	0
2001	71	13	1	3	1	1	2	2	2	0
2002	62	11	2	1	1	1	2	2	2	0
2003	60	11	1	5	1	1	2	2	2	0
2004	67	12	1	6	1	1	4	2	2	0
2005	68	12	1	2	1	1	2	2	2	0
2006	42	7	1	2	1	1	3	2	2	0
2007	35	6	1	2	1	2	3	2	2	0
2008	22	3	2	1	1	1	2	2	2	0
2009	29	4	2	2	1	1	3	2	2	0
2010	29	4	1	2	1	1	3	2	2	0

2011	59	10	2	4	1	1	3	2	2	0
2012	26	4	1	3	1	1	3	2	2	0
2013	35	6	2	2	1	1	2	2	2	0
2014	30	5	2	2	1	1	2	2	2	0
2015	40	7	1	2	1	1	2	2	2	0
2016	27	4	2	2	1	1	3	2	2	0
2017	41	7	1	2	1	1	2	2	2	0
2018	45	8	2	2	1	1	3	2	2	0
2019	50	9	1	2	1	1	2	2	2	0
2020	28	4	2	1	1	1	2	2	2	0
2021	29	4	2	8	1	2	3	2	2	0
2022	39	6	2	8	1	1	3	6	2	0
2023	45	8	1	2	1	1	2	6	2	0
2024	58	10	1	2	1	1	3	2	2	0
2025	35	6	1	4	1	1	4	6	2	0
2026	23	3	1	3	1	2	3	2	2	0
2027	38	6	1	2	1	1	2	2	2	0
2028	38	6	1	3	1	2	3	2	2	0
2029	29	4	2	2	1	1	3	6	2	0
2030	77	14	2	5	1	4	2	2	2	0
2031	68	12	2	1	1	4	2	2	2	0
2032	76	14	1	5	1	4	2	2	2	0
2033	38	6	1	2	1	1	3	2	2	0
2034	80	15	2	2	1	4	2	2	2	0
2035	22	3	1	7	1	2	3	2	2	0
2036	65	12	1	2	1	1	3	2	2	0
2037	63	11	1	2	1	1	3	2	2	0
2038	57	10	1	3	1	4	2	2	2	0
2039	62	11	1	2	1	1	3	2	2	0
2040	54	9	1	5	1	1	2	2	2	0
2041	32	5	1	1	1	1	3	2	2	0

2042	61	11	1	2	1	1	2	2	2	0
2043	56	10	2	2	1	1	2	2	2	0
2044	33	5	1	2	1	2	4	2	2	0
2045	67	12	1	3	1	1	2	2	2	0
2046	42	7	1	1	1	1	2	2	2	0
2047	55	10	2	3	1	1	3	2	2	0
2048	35	6	2	2	1	1	3	2	2	0
2049	66	12	1	4	1	1	4	6	2	0
2050	61	11	2	2	1	1	3	6	2	0
2051	75	14	1	2	1	1	3	2	2	0
2052	59	10	2	2	1	1	3	2	2	0
2053	57	10	1	8	1	1	3	2	2	0
2054	47	8	2	2	1	1	3	2	2	0
2055	38	6	1	2	1	2	2	2	2	0
2056	44	7	1	2	1	1	3	2	2	0
2057	32	5	2	2	1	1	3	2	2	0
2058	63	11	1	2	1	1	2	2	2	0
2059	67	12	1	2	1	1	2	2	2	0
2060	38	6	1	3	1	4	3	2	2	0
2061	60	11	2	1	1	5	2	2	2	0
2062	65	12	1	3	1	1	3	2	2	0
2063	59	10	2	1	1	4	2	2	2	0
2064	58	10	1	2	1	1	3	2	2	0
2065	60	11	2	2	1	4	2	2	2	0
2066	51	9	1	2	1	1	3	2	2	0
2067	63	11	2	1	1	4	2	2	2	0
2068	72	13	1	1	1	1	3	2	2	0
2069	65	12	1	2	1	1	3	2	2	0
2070	41	7	1	2	1	1	3	2	2	0
2071	45	8	2	2	1	1	2	2	2	0
2072	58	10	1	1	1	1	2	2	2	0

2073	48	8	2	1	1	1	2	2	2	0
2074	56	10	2	2	1	1	3	2	2	0
2075	65	12	2	2	1	4	2	2	2	0
2076	36	6	1	1	1	1	3	2	2	0
2077	57	10	1	3	1	1	2	2	2	0
2078	35	6	2	2	1	1	3	2	2	0
2079	38	6	1	2	1	1	2	2	2	0
2080	37	6	1	3	1	1	3	2	2	0
2081	20	3	2	2	1	1	2	2	2	0
2082	38	6	1	2	1	1	3	2	2	0
2083	20	3	2	3	1	1	3	2	2	0
2084	24	3	1	7	1	2	3	2	2	0
2085	69	12	2	1	1	1	2	2	2	0
2086	52	9	2	2	1	1	3	2	2	0
2087	30	5	2	2	1	1	3	2	2	0
2088	34	5	2	2	1	1	2	2	2	0
2089	48	8	1	2	1	1	2	2	2	0
2090	93	15	2	5	1	4	1	2	2	0
2091	63	11	1	1	1	1	2	2	2	0
2092	55	10	2	2	1	4	2	2	2	0
2093	50	9	2	1	1	4	3	2	2	0
2094	66	12	2	5	1	4	2	2	2	0
2095	55	10	1	3	1	1	2	2	2	0
2096	78	14	1	1	1	1	2	2	2	0
2097	65	12	2	2	1	1	2	2	2	0
2098	30	5	1	2	1	1	3	2	2	0
2099	44	7	1	2	1	1	2	2	2	0
2100	62	11	1	2	1	1	2	2	2	0
2101	42	7	2	2	1	4	3	2	2	0
2102	22	3	2	2	1	1	3	2	2	0
2103	35	6	2	2	1	1	3	2	2	0

2104	30	5	1	7	1	2	4	2	2	0
2105	26	4	1	7	1	1	2	2	2	0
2106	40	7	2	2	1	2	3	2	2	0
2107	23	3	2	7	1	2	3	2	2	0
2108	78	14	1	1	1	1	2	2	2	0
2109	28	4	1	8	1	2	3	2	2	0
2110	68	12	1	2	1	1	2	2	2	0
2111	45	8	1	2	1	1	2	2	2	0
2112	49	8	2	2	1	1	2	2	2	0
2113	30	5	2	3	1	1	3	2	2	0
2114	48	8	1	1	1	1	2	2	2	0
2115	54	9	1	2	1	1	3	2	2	0
2116	40	7	1	3	1	1	2	2	2	0
2117	38	6	1	2	1	1	2	2	2	0
2118	17	2	2	7	1	2	3	2	2	0
2119	42	7	1	3	1	1	3	2	2	0
2120	38	6	2	2	1	1	3	2	2	0
2121	40	7	1	3	1	1	2	2	2	0
2122	10	1	1	7	1	2	2	2	2	0
2123	28	4	1	3	1	1	3	2	2	0
2124	45	8	1	3	1	1	3	2	2	0
2125	37	6	2	2	1	1	2	2	2	0
2126	16	2	1	7	1	2	3	2	2	0
2127	19	2	2	8	1	1	3	2	2	0
2128	45	8	1	3	1	1	2	2	2	0
2129	45	8	1	3	1	1	3	2	2	0
2130	27	4	2	2	1	1	2	2	2	0
2131	60	11	2	2	1	4	2	2	2	0
2132	45	8	1	3	1	1	2	2	2	0
2133	50	9	2	2	1	1	3	2	2	0
2134	41	7	1	2	1	1	3	2	2	0

2135	52	9	2	2	1	1	3	2	2	0
2136	10	1	1	7	1	2	2	2	2	0
2137	15	2	1	7	1	2	3	2	2	0
2138	68	12	2	1	1	2	2	2	2	0
2139	49	8	2	2	1	1	3	2	2	0
2140	21	3	1	2	1	2	3	2	2	0
2141	12	1	1	7	1	2	2	2	2	0
2142	56	10	1	1	1	1	2	2	2	0
2143	67	12	1	3	1	1	3	4	2	0
2144	58	10	2	2	1	1	2	4	2	0
2145	60	11	1	2	1	1	3	4	2	0
2146	52	9	2	2	1	1	3	4	2	0
2147	72	13	1	6	1	4	4	2	2	0
2148	76	14	1	2	1	1	3	2	2	0
2149	80	15	2	5	1	4	2	2	2	0
2150	63	11	1	5	1	4	2	2	2	0
2151	55	10	2	2	1	2	3	2	2	0
2152	42	7	2	2	1	2	3	2	2	0
2153	68	12	1	2	1	1	2	2	2	0
2154	34	5	2	2	1	1	2	6	2	0
2155	43	7	1	2	1	1	2	6	2	0
2156	26	4	2	2	1	1	2	2	2	0
2157	60	11	1	2	1	1	2	2	2	0
2158	57	10	1	6	1	4	3	2	2	0
2159	69	12	2	6	1	4	4	2	2	0
2160	55	10	2	2	1	1	3	2	2	0
2161	16	2	2	7	1	2	3	2	2	0
2162	70	13	2	6	1	4	4	2	2	0
2163	55	10	2	2	1	1	3	2	2	0
2164	65	12	1	2	1	4	2	2	2	0
2165	17	2	1	7	1	2	3	2	2	0

2166	81	15	1	6	1	4	2	2	2	0
2167	71	13	1	1	1	1	2	2	2	0
2168	67	12	1	2	1	1	2	2	2	0
2169	63	11	1	4	1	1	2	2	2	0
2170	73	13	1	6	1	1	2	2	2	0
2171	77	14	1	6	1	4	2	2	2	0
2172	55	10	1	2	1	1	2	2	2	0
2173	60	11	1	2	1	4	2	2	2	0
2174	77	14	1	2	1	1	2	2	2	0
2175	90	15	1	6	1	1	2	2	2	0
2176	70	13	2	5	1	4	2	2	2	0
2177	28	4	2	1	1	4	3	2	2	0
2178	70	13	1	3	1	1	2	2	2	0
2179	56	10	2	1	1	4	1	2	2	0
2180	40	7	2	1	1	4	2	2	2	0
2181	40	7	2	2	1	4	2	4	2	0
2182	60	11	1	1	1	1	3	4	2	0
2183	36	6	1	3	1	2	3	4	2	0
2184	53	9	1	3	3	1	2	2	2	0
2185	60	11	2	1	1	4	1	4	2	0
2186	57	10	1	2	1	1	3	4	2	0
2187	57	10	1	3	3	1	2	4	2	0
2188	42	7	1	2	1	2	3	4	2	0
2189	50	9	2	1	1	4	3	4	2	0
2190	70	13	2	5	1	4	1	4	2	0
2191	40	7	1	3	1	1	3	4	2	0
2192	52	9	1	3	1	1	2	4	2	0
2193	76	14	1	1	1	1	3	4	2	0
2194	45	8	1	1	1	1	2	4	2	0
2195	60	11	2	1	1	4	1	2	2	0
2196	57	10	2	1	1	4	3	2	2	0

2197	61	11	2	1	1	1	2	4	2	0
2198	52	9	2	4	1	1	3	4	2	0
2199	45	8	1	3	1	1	2	4	2	0
2200	50	9	1	4	1	1	4	4	2	0
2201	73	13	2	2	1	4	2	4	2	0
2202	30	5	1	3	1	1	2	4	2	0
2203	58	10	2	2	1	4	2	4	2	0
2204	75	14	1	1	1	1	2	4	2	0
2205	60	11	1	1	1	1	3	4	2	0
2206	45	8	2	1	1	4	3	4	2	0
2207	52	9	1	2	1	1	3	4	2	0
2208	45	8	1	1	1	1	2	4	2	0
2209	31	5	2	2	1	1	3	4	2	0
2210	53	9	1	1	1	1	1	4	2	0
2211	56	10	2	1	1	1	3	4	2	0
2212	36	6	1	1	1	1	2	4	2	0
2213	62	11	1	2	3	1	2	2	2	0
2214	60	11	1	1	5	1	2	4	2	0
2215	34	5	1	2	3	1	2	4	2	0
2216	52	9	2	1	1	4	2	4	2	0
2217	30	5	2	1	1	4	2	4	2	0
2218	70	13	2	1	1	4	2	4	2	0
2219	66	12	2	1	1	4	2	4	2	0
2220	62	11	2	2	1	4	3	4	2	0
2221	54	9	2	1	1	4	1	4	2	0
2222	50	9	2	1	1	4	1	4	2	0
2223	40	7	2	1	1	4	3	4	2	0
2224	82	15	1	1	1	1	2	4	2	0
2225	75	14	1	3	1	1	2	4	2	0
2226	70	13	1	3	1	1	2	4	2	0
2227	80	15	2	1	1	4	1	4	2	0

2228	60	11	2	1	1	4	2	4	2	0
2229	81	15	2	1	1	4	1	4	2	0
2230	62	11	1	1	1	1	1	4	2	0
2231	40	7	1	2	1	1	3	4	2	0
2232	75	14	2	1	1	1	3	4	2	0
2233	40	7	1	3	1	1	3	4	2	0
2234	76	14	1	3	1	1	1	4	2	0
2235	40	7	2	2	1	4	3	4	2	0
2236	48	8	2	2	1	1	3	4	2	0
2237	48	8	1	4	1	1	2	4	2	0
2238	46	8	1	2	1	1	3	4	2	0
2239	56	10	2	1	1	1	2	4	2	0
2240	23	3	2	2	1	2	3	4	2	0
2241	20	3	2	2	1	2	3	4	2	0
2242	30	5	1	3	2	1	2	4	2	0
2243	27	4	1	2	1	2	3	4	2	0
2244	32	5	1	2	1	1	2	4	1	2
2245	27	4	2	2	1	1	3	4	2	0
2246	82	15	1	6	1	1	2	4	2	0
2247	75	14	1	6	1	1	3	4	2	0
2248	70	13	1	1	1	1	2	4	2	0
2249	72	13	2	6	1	4	2	4	2	0
2250	38	6	2	1	1	4	2	4	2	0
2251	55	10	1	3	1	1	3	4	2	0
2252	40	7	2	2	1	4	2	4	2	0
2253	70	13	1	6	1	1	2	4	2	0
2254	19	2	1	2	1	1	2	4	2	0
2255	55	10	2	1	1	4	2	4	2	0
2256	35	6	1	1	1	1	2	4	2	0
2257	33	5	1	2	1	1	2	4	2	0
2258	50	9	1	2	1	1	2	2	2	0

2259	31	5	1	3	1	1	2	4	2	0
2260	54	9	1	8	3	1	2	4	2	0
2261	36	6	1	3	1	1	3	4	2	0
2262	24	3	2	3	1	1	2	4	2	0
2263	25	4	1	2	1	1	2	6	2	0
2264	22	3	2	1	1	1	2	6	2	0
2265	16	2	2	7	1	2	3	4	2	0
2266	23	3	1	1	1	2	2	4	2	0
2267	25	4	1	1	1	2	2	4	2	0
2268	42	7	2	1	1	1	2	4	2	0
2269	62	11	1	1	1	1	2	4	2	0
2270	20	3	2	7	1	2	3	4	2	0
2271	74	13	1	6	1	1	3	4	2	0
2272	37	6	2	1	1	1	2	4	2	0
2273	55	10	2	1	1	1	2	4	2	0
2274	42	7	2	1	1	1	3	4	2	0
2275	33	5	2	2	1	1	3	4	2	0
2276	47	8	1	2	3	1	2	4	2	0
2277	31	5	1	3	1	2	2	4	1	1
2278	52	9	2	4	1	3	3	4	1	1
2279	38	6	1	3	1	1	3	4	2	0
2280	28	4	2	2	1	1	3	4	2	0
2281	18	2	1	7	1	2	3	4	2	0
2282	13	1	2	7	1	2	3	4	2	0
2283	53	9	2	5	3	1	3	4	2	0
2284	32	5	2	3	1	1	2	4	2	0
2285	41	7	1	1	1	1	2	4	2	0
2286	10	1	2	7	1	2	2	6	2	0
2287	43	7	1	3	1	1	2	4	2	0
2288	31	5	2	2	1	1	2	4	2	0
2289	65	12	1	1	1	1	1	4	2	0

2290	46	8	1	1	1	1	3	4	2	0
2291	34	5	2	2	1	1	3	4	2	0
2292	25	4	1	8	1	2	3	4	2	0
2293	65	12	2	5	1	1	1	4	2	0
2294	36	6	1	3	1	1	2	4	2	0
2295	27	4	2	2	1	1	2	4	2	0
2296	51	9	2	2	3	1	2	4	2	0
2297	51	9	1	3	1	1	2	4	2	0
2298	64	11	1	1	1	1	2	4	2	0
2299	25	4	1	3	2	1	3	4	2	0
2300	35	6	1	4	1	1	3	4	2	0
2301	21	3	2	2	1	1	3	4	2	0
2302	28	4	2	2	1	1	2	4	2	0
2303	39	6	2	1	1	1	3	4	2	0
2304	52	9	2	2	1	4	2	4	2	0
2305	26	4	2	2	1	1	2	4	2	0
2306	23	3	1	1	1	1	2	4	2	0
2307	40	7	2	2	1	1	3	4	2	0
2308	31	5	2	2	1	1	3	4	2	0
2309	23	3	2	7	1	2	3	4	2	0
2310	59	10	2	1	1	1	3	4	2	0
2311	53	9	2	1	1	1	2	4	2	0
2312	40	7	1	2	1	1	3	4	2	0
2313	65	12	1	1	1	1	2	4	2	0
2314	22	3	1	8	1	2	3	4	2	0
2315	58	10	1	4	1	1	3	4	2	0
2316	34	5	1	2	1	1	2	4	2	0
2317	16	2	1	7	1	2	3	4	2	0
2318	34	5	2	1	1	1	3	4	2	0
2319	32	5	2	2	1	1	4	4	2	0
2320	53	9	1	2	1	1	3	4	2	0

2321	65	12	2	1	1	4	3	4	2	0
2322	42	7	1	2	1	1	2	4	2	0
2323	21	3	1	7	1	2	4	4	2	0
2324	65	12	1	1	1	1	2	4	2	0
2325	28	4	1	3	1	2	4	2	2	0
2326	23	3	1	8	1	2	3	4	2	0
2327	49	8	1	2	1	1	2	4	2	0
2328	43	7	1	2	1	1	3	4	2	0
2329	22	3	1	3	1	2	3	4	2	0
2330	63	11	1	6	1	1	2	4	2	0
2331	30	5	1	2	1	1	3	4	2	0
2332	34	5	1	3	1	1	3	4	2	0
2333	35	6	2	1	1	1	2	4	2	0
2334	65	12	2	5	1	1	2	4	2	0
2335	34	5	1	2	1	1	3	4	2	0
2336	13	1	2	7	1	2	3	2	2	0
2337	25	4	2	3	1	2	3	4	2	0
2338	43	7	2	4	1	1	3	4	2	0
2339	13	1	2	7	1	2	3	4	2	0
2340	52	9	2	1	1	4	2	4	2	0
2341	53	9	1	3	1	1	3	4	2	0
2342	41	7	1	3	1	1	3	4	2	0
2343	53	9	1	3	1	1	2	4	2	0
2344	23	3	1	8	1	2	3	4	2	0
2345	11	1	2	7	1	2	3	4	2	0
2346	34	5	1	5	1	2	1	4	2	0
2347	23	3	1	7	1	1	3	4	2	0
2348	32	5	1	2	1	1	2	4	2	0
2349	30	5	1	2	1	2	3	4	2	0
2350	60	11	1	3	1	4	1	4	2	0
2351	12	1	1	7	1	2	2	4	2	0

2352	48	8	1	3	1	1	3	4	2	0
2353	52	9	2	1	1	1	3	4	2	0
2354	57	10	2	2	1	1	2	6	2	0
2355	24	3	1	3	1	2	3	4	2	0
2356	38	6	1	1	1	1	3	4	2	0
2357	35	6	2	1	1	1	3	4	2	0
2358	64	11	1	2	1	1	2	4	2	0
2359	61	11	1	3	1	1	3	4	2	0
2360	27	4	2	1	1	1	3	4	2	0
2361	25	4	1	3	1	1	2	4	2	0
2362	46	8	1	2	1	1	2	4	2	0
2363	54	9	1	1	1	4	3	4	2	0
2364	45	8	1	2	1	1	3	4	2	0
2365	51	9	1	1	1	1	2	4	2	0
2366	59	10	1	1	1	1	2	4	2	0
2367	61	11	1	2	1	1	2	4	2	0
2368	43	7	1	8	3	1	2	4	2	0
2369	43	7	2	8	1	4	3	4	2	0
2370	41	7	1	1	1	1	3	4	2	0
2371	63	11	1	1	1	1	2	6	2	0
2372	52	9	1	2	1	1	3	6	2	0
2373	62	11	1	1	1	1	2	6	2	0
2374	60	11	2	1	1	4	2	6	2	0
2375	61	11	1	2	1	1	2	3	1	1
2376	60	11	1	3	1	1	2	6	2	0
2377	48	8	1	4	1	1	3	6	2	0
2378	32	5	1	3	1	1	3	6	2	0
2379	64	11	1	1	1	1	2	6	2	0
2380	60	11	1	1	1	1	2	6	2	0
2381	65	12	2	1	1	1	2	6	2	0
2382	58	10	2	1	1	1	2	6	2	0

2383	65	12	2	1	1	4	1	6	2	0
2384	32	5	2	3	1	1	3	6	2	0
2385	40	7	1	3	1	2	3	6	2	0
2386	38	6	1	1	1	2	3	6	2	0
2387	63	11	2	2	1	4	1	6	2	0
2388	40	7	1	3	1	1	3	6	2	0
2389	70	13	1	3	3	1	1	6	2	0
2390	71	13	1	1	1	1	3	6	2	0
2391	65	12	1	2	1	1	3	6	2	0
2392	45	8	1	2	1	1	2	6	2	0
2393	35	6	1	3	1	1	4	6	2	0
2394	34	5	1	3	1	1	3	6	2	0
2395	55	10	2	1	1	4	1	6	2	0
2396	60	11	2	1	1	4	1	6	2	0
2397	60	11	2	6	1	1	2	6	2	0
2398	30	5	1	1	3	1	2	6	2	0
2399	50	9	2	2	1	1	1	6	2	0
2400	25	4	2	4	1	2	4	6	2	0
2401	26	4	1	8	1	1	4	6	2	0
2402	70	13	1	6	1	1	2	6	2	0
2403	55	10	2	1	3	1	2	6	2	0
2404	60	11	2	6	1	4	1	6	2	0
2405	36	6	2	2	1	4	3	6	2	0
2406	70	13	2	1	1	4	2	6	2	0
2407	39	6	2	1	1	1	3	6	2	0
2408	67	12	2	1	1	4	2	6	2	0
2409	73	13	1	1	1	1	2	6	2	0
2410	59	10	2	1	1	4	3	6	2	0
2411	30	5	2	2	1	1	3	6	2	0
2412	83	15	1	2	1	1	3	6	2	0
2413	45	8	1	2	1	1	2	6	2	0

2414	86	15	2	1	1	1	2	6	2	0
2415	50	9	2	1	1	1	2	6	2	0
2416	69	12	1	1	1	4	2	6	2	0
2417	50	9	1	1	1	1	3	6	2	0
2418	45	8	2	1	1	4	2	6	2	0
2419	55	10	2	1	1	4	2	6	2	0
2420	72	13	2	5	1	4	2	6	2	0
2421	67	12	1	1	1	1	2	6	2	0
2422	50	9	2	1	1	4	2	6	2	0
2423	40	7	2	1	1	1	4	6	2	0
2424	59	10	2	1	1	4	1	6	2	0
2425	74	13	2	1	1	4	1	6	2	0
2426	37	6	2	1	1	1	3	6	2	0
2427	45	8	1	2	1	1	3	6	2	0
2428	50	9	1	3	3	1	2	6	2	0
2429	51	9	2	2	1	1	3	6	2	0
2430	25	4	2	2	1	1	3	6	2	0
2431	51	9	1	1	1	1	2	6	2	0
2432	54	9	2	3	1	1	2	6	2	0
2433	44	7	2	2	3	1	3	6	2	0
2434	34	5	1	3	3	2	3	6	2	0
2435	64	11	1	5	3	1	2	6	2	0
2436	40	7	1	8	1	1	3	6	2	0
2437	43	7	1	1	1	1	3	6	2	0
2438	34	5	1	2	1	1	4	4	2	0
2439	59	10	1	3	1	1	3	4	2	0
2440	46	8	1	3	1	1	3	4	1	2
2441	40	7	2	1	1	4	2	4	2	0
2442	47	8	2	1	1	4	3	6	2	0
2443	40	7	2	2	1	1	2	4	2	0
2444	51	9	2	2	1	4	2	6	2	0

2445	88	15	2	2	1	4	2	6	2	0
2446	76	14	1	5	1	4	3	6	2	0
2447	50	9	1	3	1	1	2	6	2	0
2448	45	8	2	1	1	1	3	6	1	0
2449	55	10	1	3	1	1	1	6	2	0
2450	60	11	2	1	1	1	2	6	2	0
2451	48	8	1	3	1	1	2	6	1	0
2452	32	5	2	3	1	1	3	6	1	1
2453	34	5	2	3	1	2	3	6	2	0
2454	37	6	2	3	1	1	2	6	2	0
2455	59	10	1	2	1	1	3	6	2	0
2456	69	12	2	1	1	1	3	6	2	0
2457	61	11	2	2	1	4	2	2	1	0
2458	71	13	2	6	1	4	4	2	2	0
2459	53	9	2	2	1	1	3	2	2	0
2460	35	6	1	3	1	1	3	2	2	0
2461	60	11	1	2	1	1	3	2	2	0
2462	45	8	1	3	1	1	3	6	2	0
2463	50	9	2	2	1	4	2	6	2	0
2464	66	12	2	6	1	4	4	6	1	0
2465	74	13	1	3	1	1	3	6	2	0
2466	63	11	2	1	1	4	2	6	2	0
2467	38	6	1	3	1	1	3	6	2	0
2468	51	9	2	1	1	4	2	6	2	0
2469	69	12	1	3	1	4	3	6	1	0
2470	62	11	1	8	1	1	2	6	2	0
2471	78	14	2	6	1	4	2	6	2	0
2472	45	8	1	3	1	1	3	6	2	0
2473	60	11	1	3	1	1	2	6	2	0
2474	60	11	1	1	1	1	1	6	2	0
2475	63	11	1	3	1	1	2	6	2	0

2476	50	9	1	1	1	1	2	6	2	0
2477	49	8	1	1	3	1	2	6	2	0
2478	59	10	1	3	1	1	2	6	2	0
2479	64	11	1	1	1	1	2	6	2	0
2480	62	11	2	1	1	1	2	4	2	0
2481	64	11	2	1	1	1	2	4	2	0
2482	42	7	2	1	1	1	3	4	2	0
2483	53	9	1	3	1	1	1	4	2	0
2484	60	11	2	1	1	1	2	4	2	0
2485	30	5	1	3	1	1	3	4	2	0
2486	49	8	2	1	1	1	2	4	2	0
2487	67	12	1	6	1	1	4	4	2	0
2488	77	14	1	5	3	1	1	4	2	0
2489	59	10	2	5	3	1	2	4	2	0
2490	68	12	2	1	3	1	1	4	2	0
2491	66	12	1	1	1	1	2	4	2	0
2492	50	9	1	2	1	1	3	4	2	0
2493	35	6	2	3	1	1	2	4	2	0
2494	68	12	1	3	1	1	2	4	2	0
2495	57	10	2	1	1	1	2	4	2	0
2496	68	12	2	1	1	1	3	4	2	0
2497	47	8	2	1	1	1	2	4	2	0
2498	39	6	1	1	1	1	2	4	2	0
2499	50	9	2	6	1	1	2	4	2	0
2500	34	5	1	2	1	1	3	4	2	0
2501	31	5	1	3	1	1	2	4	2	0
2502	70	13	1	6	1	1	4	4	2	0
2503	73	13	1	8	1	1	4	4	2	0
2504	57	10	1	3	1	1	3	4	2	0
2505	26	4	2	1	1	1	2	4	2	0
2506	71	13	1	3	1	1	2	4	2	0

2507	35	6	1	2	1	1	3	4	2	0
2508	56	10	1	3	1	1	3	4	2	0
2509	17	2	1	7	1	2	3	4	2	0
2510	67	12	2	1	1	4	2	4	2	0
2511	52	10	2	2	1	1	3	4	2	0
2512	65	12	2	1	1	1	2	4	2	0
2513	34	5	2	2	1	1	2	4	2	0
2514	40	7	2	1	3	2	2	4	1	1
2515	28	4	2	5	1	2	2	4	1	1
2516	74	13	1	2	1	1	2	4	2	0
2517	85	15	2	1	1	4	2	4	2	0
2518	62	11	2	1	1	1	2	4	2	0
2519	32	5	1	1	1	2	3	5	2	0
2520	86	15	1	1	3	4	1	5	2	0
2521	15	2	2	7	1	2	3	4	2	0
2522	32	5	2	1	1	1	2	4	2	0
2523	71	13	1	3	1	1	2	4	2	0
2524	59	10	1	3	1	1	2	4	2	0
2525	66	12	1	2	1	1	3	4	2	0
2526	55	10	1	4	1	1	3	4	2	0
2527	48	8	1	2	1	1	3	4	2	0
2528	57	10	1	2	1	1	3	4	2	0
2529	55	10	1	2	1	1	3	4	2	0
2530	68	12	2	2	1	2	2	4	2	0
2531	79	14	1	2	1	1	3	4	2	0
2532	31	5	2	8	1	2	4	4	2	0
2533	47	8	1	2	1	1	3	4	2	0
2534	39	6	1	2	1	2	3	4	2	0
2535	50	9	2	1	1	1	3	4	2	0
2536	35	6	2	2	1	1	3	4	2	0
2537	29	4	1	1	1	2	2	4	2	0

2538	77	14	1	3	1	1	3	4	2	0
2539	70	13	1	1	1	1	3	4	2	0
2540	54	9	2	3	1	1	3	4	2	0
2541	18	2	1	7	1	2	3	4	2	0
2542	32	5	2	2	1	1	3	2	2	0
2543	60	11	1	2	1	1	3	2	2	0
2544	50	9	1	1	1	1	2	2	2	0
2545	31	5	2	2	1	1	2	2	2	0
2546	15	2	2	7	1	2	3	2	2	0
2547	32	5	1	3	1	1	3	2	2	0
2548	30	5	2	1	1	1	3	2	2	0
2549	90	15	1	2	1	4	2	2	2	0
2550	52	9	2	1	1	1	3	2	2	0
2551	52	9	2	2	1	4	3	2	2	0
2552	40	7	2	2	4	4	3	2	2	0
2553	35	6	2	3	1	1	3	2	2	0
2554	83	15	2	5	1	4	1	2	2	0
2555	40	7	1	2	1	1	3	2	2	0
2556	46	8	2	2	1	4	3	2	2	0
2557	50	9	2	1	1	1	2	2	2	0
2558	80	15	1	1	1	1	2	2	2	0
2559	41	7	2	2	1	4	2	2	2	0
2560	73	13	2	2	1	4	3	2	2	0
2561	50	9	1	2	1	1	2	2	2	0
2562	61	11	2	2	1	1	2	2	2	0
2563	66	12	2	2	4	1	3	2	2	0
2564	68	12	2	1	1	1	3	2	2	0
2565	49	8	2	2	1	1	3	2	2	0
2566	70	13	2	2	1	1	2	2	2	0
2567	73	13	1	1	1	1	2	2	2	0
2568	94	15	2	5	1	4	1	2	2	0

2569	69	12	1	2	1	1	3	2	2	0
2570	65	12	1	1	1	1	3	2	2	0
2571	45	8	1	2	4	1	3	2	2	0
2572	94	15	2	2	1	4	2	2	2	0
2573	88	15	2	2	1	4	2	2	2	0
2574	68	12	1	2	1	1	3	2	2	0
2575	56	10	1	2	1	1	3	2	2	0
2576	20	3	2	7	1	2	3	2	2	0
2577	39	6	1	2	1	1	2	2	2	0
2578	41	7	1	2	1	1	3	2	2	0
2579	37	6	1	1	1	1	2	2	2	0
2580	47	8	1	2	1	1	3	2	2	0
2581	49	8	2	2	1	4	3	2	2	0
2582	59	10	2	5	1	4	3	2	2	0
2583	50	9	2	2	1	4	2	2	2	0
2584	47	8	1	2	1	2	3	2	2	0
2585	57	10	2	2	1	4	2	2	2	0
2586	65	12	2	5	1	4	3	2	2	0
2587	76	14	1	2	1	4	3	2	2	0
2588	59	10	2	2	1	4	3	2	2	0
2589	46	8	1	5	1	4	3	2	2	0
2590	88	15	1	2	1	1	2	2	2	0
2591	75	14	1	2	1	1	2	2	2	0
2592	59	10	2	6	1	4	3	2	2	0
2593	12	1	1	7	1	2	2	2	2	0
2594	23	3	2	7	1	2	3	2	2	0
2595	34	5	2	2	1	1	3	2	2	0
2596	21	3	2	7	1	2	4	2	2	0
2597	34	5	2	2	1	1	2	2	2	0
2598	31	5	2	1	1	1	2	2	2	0
2599	49	8	2	2	1	1	2	2	2	0

2600	12	1	2	7	1	2	2	2	2	0
2601	25	4	2	2	1	1	3	2	2	0
2602	24	3	1	8	1	2	3	2	2	0
2603	45	8	2	2	1	1	3	2	2	0
2604	40	7	2	2	1	1	3	2	2	0
2605	73	13	2	2	1	4	2	2	2	0
2606	54	9	2	2	1	1	2	2	2	0
2607	45	8	2	1	1	1	3	2	2	0
2608	45	8	1	2	1	1	3	2	2	0
2609	66	12	1	2	1	4	3	2	2	0
2610	47	8	2	2	1	1	2	4	2	0
2611	40	7	1	2	1	1	3	4	2	0
2612	22	3	1	3	1	2	3	4	2	0
2613	75	14	1	5	1	1	1	4	2	0
2614	80	15	2	5	1	4	2	4	2	0
2615	45	8	1	3	1	1	2	4	2	0
2616	21	3	1	7	1	2	3	4	2	0
2617	56	10	1	1	1	1	3	4	2	0
2618	24	3	2	3	1	1	3	4	2	0
2619	32	5	2	1	1	1	3	4	2	0
2620	41	7	1	2	1	1	3	4	2	0
2621	55	10	1	2	1	1	3	4	2	0
2622	37	6	2	2	1	1	3	4	2	0
2623	12	1	1	7	1	2	2	4	2	0
2624	44	7	1	2	1	1	3	4	2	0
2625	30	5	1	3	1	2	3	4	2	0
2626	30	5	1	1	1	1	3	4	2	0
2627	53	9	1	1	1	5	3	4	2	0
2628	23	3	1	7	1	2	3	4	2	0
2629	42	7	1	2	1	1	3	4	2	0
2630	21	3	2	7	1	2	3	4	2	0

2631	45	8	1	2	1	1	2	2	2	0
2632	44	7	2	5	1	1	2	2	2	0
2633	58	10	1	4	1	1	4	2	2	0
2634	47	8	1	2	1	1	3	2	2	0
2635	35	6	2	2	1	1	3	2	2	0
2636	52	9	1	4	1	1	4	2	2	0
2637	59	10	2	2	1	4	2	2	2	0
2638	81	15	1	6	1	1	3	2	2	0
2639	55	10	1	2	1	1	3	2	2	0
2640	37	6	1	2	1	1	3	2	2	0
2641	58	10	1	2	1	1	3	2	2	0
2642	12	1	2	7	1	2	2	2	2	0
2643	71	13	1	6	1	1	3	2	2	0
2644	45	8	2	2	1	1	3	2	2	0
2645	48	8	2	2	1	4	3	6	2	0
2646	14	1	2	7	1	2	3	4	2	0
2647	72	13	2	5	1	4	2	4	2	0
2648	36	6	2	2	1	1	3	4	2	0
2649	75	14	2	6	1	4	4	4	2	0
2650	62	11	1	2	1	1	2	4	2	0
2651	75	14	2	2	1	4	2	4	2	0
2652	50	9	1	2	1	1	2	4	2	0
2653	85	15	1	5	1	1	2	4	2	0
2654	46	8	2	2	1	4	2	6	2	0
2655	45	8	1	2	1	1	2	6	2	0
2656	65	12	1	4	1	1	4	2	2	0
2657	75	14	1	2	1	1	2	6	2	0
2658	46	8	2	4	1	4	4	2	2	0
2659	40	7	1	2	1	1	2	2	2	0
2660	54	9	2	2	1	1	2	2	2	0
2661	72	13	1	2	1	4	2	2	2	0

2662	48	8	1	2	1	1	2	2	2	0
2663	53	9	1	2	1	1	2	2	2	0
2664	72	13	1	5	1	1	2	2	2	0
2665	37	6	1	2	1	1	3	2	2	0
2666	39	6	1	2	1	1	3	2	2	0
2667	68	12	2	2	1	4	2	2	2	0
2668	23	3	2	3	1	1	3	2	2	0
2669	72	13	2	2	1	4	2	2	2	0
2670	15	2	2	7	1	2	3	2	2	0
2671	27	4	2	8	1	1	4	2	2	0
2672	29	4	1	8	1	2	4	2	2	0
2673	32	5	1	8	1	2	4	2	2	0
2674	29	4	2	8	1	2	4	2	2	0
2675	44	7	1	2	1	1	3	2	2	0
2676	64	11	1	2	1	1	2	2	2	0
2677	34	5	2	2	1	1	2	2	2	0
2678	36	6	1	2	1	1	3	2	2	0
2679	54	9	1	3	1	1	3	2	2	0
2680	49	8	1	2	1	1	3	2	2	0
2681	60	11	2	2	1	4	3	2	2	0
2682	52	9	1	3	1	1	2	2	2	0
2683	68	12	1	2	1	1	3	4	2	0
2684	35	6	2	3	1	2	3	4	2	0
2685	62	11	1	2	1	1	3	4	2	0
2686	32	5	1	3	1	2	3	4	2	0
2687	35	6	1	2	1	1	3	4	2	0
2688	40	7	1	2	1	1	3	4	2	0
2689	39	6	1	2	1	1	2	4	2	0
2690	17	2	2	7	1	2	3	4	2	0
2691	41	7	1	1	1	1	3	4	2	0
2692	34	5	2	2	1	1	3	4	2	0

2693	49	8	2	1	1	1	3	4	2	0
2694	62	11	2	2	1	4	3	4	2	0
2695	62	11	2	4	1	2	4	4	2	0
2696	36	6	1	2	1	1	3	4	2	0
2697	50	9	2	4	1	1	4	4	2	0
2698	20	3	2	2	1	2	3	4	2	0
2699	45	8	1	2	1	1	2	4	2	0
2700	42	7	1	2	1	1	2	4	2	0
2701	74	13	1	6	1	1	4	4	2	0
2702	52	9	1	4	1	1	4	4	2	0
2703	56	10	2	2	1	1	2	6	2	0
2704	65	12	1	3	3	1	2	2	2	0
2705	27	4	2	8	1	2	4	2	2	0
2706	26	4	1	7	1	2	4	2	2	0
2707	21	3	1	7	1	2	3	2	2	0
2708	46	8	1	4	3	1	3	2	2	0
2709	52	9	1	6	1	1	3	2	2	0
2710	13	1	1	7	1	2	2	2	2	0
2711	62	11	2	5	1	1	2	2	2	0
2712	32	5	1	2	1	1	3	2	2	0
2713	40	7	1	3	2	1	3	2	2	0
2714	41	7	1	2	1	1	2	2	2	0
2715	28	4	2	2	1	1	3	2	2	0
2716	24	3	1	2	1	2	3	2	2	0
2717	28	4	1	3	1	2	2	2	2	0
2718	40	7	1	3	1	1	3	2	2	0
2719	40	7	1	3	1	1	3	6	2	0
2720	55	10	2	4	1	1	4	2	2	0
2721	45	8	1	3	1	1	3	2	2	0
2722	46	8	1	2	1	1	2	2	2	0
2723	35	6	1	3	1	1	3	2	2	0

2724	49	8	2	2	1	1	3	6	2	0
2725	49	8	1	2	1	1	3	4	2	0
2726	50	9	1	2	1	1	2	4	2	0
2727	43	7	1	2	1	1	3	4	2	0
2728	58	10	1	2	1	1	2	4	2	0
2729	65	12	1	4	1	1	4	4	2	0
2730	45	8	2	4	1	4	3	4	2	0
2731	53	9	2	3	1	1	3	4	2	0
2732	48	8	1	3	1	1	3	4	2	0
2733	37	6	2	2	1	1	3	4	2	0
2734	43	7	2	2	1	4	2	4	2	0
2735	32	5	2	2	1	1	2	4	2	0
2736	44	7	2	2	1	1	3	4	2	0
2737	11	1	1	7	1	2	2	4	2	0
2738	48	8	1	2	1	1	2	4	2	0
2739	32	5	2	3	1	1	3	4	2	0
2740	38	6	2	1	1	1	2	4	2	0
2741	21	3	1	8	1	2	4	3	2	0
2742	40	7	1	3	1	1	2	4	2	0
2743	38	6	1	2	1	1	3	2	2	0
2744	57	10	1	2	1	1	2	2	2	0
2745	43	7	1	3	1	1	3	2	2	0
2746	57	10	1	3	1	1	2	2	2	0
2747	17	2	2	7	1	2	3	2	2	0
2748	53	9	1	2	1	1	2	2	2	0
2749	67	12	2	5	1	1	3	2	2	0
2750	44	7	1	2	1	1	3	2	2	0
2751	39	6	2	2	1	1	3	6	2	0
2752	41	7	1	4	1	6	3	6	2	0
2753	57	10	2	1	1	1	2	2	2	0
2754	39	6	1	3	1	1	3	2	2	0

2755	72	13	1	3	1	1	3	2	2	0
2756	34	5	1	3	1	1	3	2	2	0
2757	48	8	1	3	1	1	3	2	2	0
2758	73	13	1	3	1	1	2	2	2	0
2759	44	7	1	2	1	1	3	2	2	0
2760	47	8	1	3	1	1	3	2	2	0
2761	64	11	1	5	1	1	2	2	2	0
2762	41	7	1	3	1	1	3	2	2	0
2763	40	7	1	2	1	1	2	4	2	0
2764	45	8	1	5	1	1	2	4	2	0
2765	65	12	1	2	1	1	2	4	2	0
2766	52	9	2	2	1	4	2	4	2	0
2767	61	11	1	3	1	1	3	4	2	0
2768	14	1	2	7	1	2	3	4	2	0
2769	19	2	2	7	1	2	3	4	2	0
2770	57	10	1	2	1	1	2	4	2	0
2771	26	4	1	2	1	1	3	4	2	0
2772	31	5	1	2	1	1	3	4	2	0
2773	34	5	1	2	1	1	3	4	2	0
2774	33	5	1	3	1	1	2	4	2	0
2775	63	11	1	2	1	1	3	4	2	0
2776	52	9	1	3	1	2	3	4	2	0
2777	74	13	1	5	1	1	2	4	2	0
2778	52	9	1	1	1	1	3	4	2	0
2779	47	8	1	3	1	1	3	4	2	0
2780	61	11	1	3	1	1	3	4	2	0
2781	70	13	2	2	1	4	2	4	2	0
2782	65	12	2	2	1	4	2	4	2	0
2783	35	6	1	3	1	1	3	6	2	0
2784	43	7	1	3	1	1	2	4	2	0
2785	15	2	2	7	1	2	3	4	2	0

2786	12	1	2	7	1	2	3	4	2	0
2787	45	8	2	2	1	1	2	4	2	0
2788	64	11	1	2	1	1	2	4	2	0
2789	49	8	1	2	1	1	2	4	2	0
2790	46	8	2	2	1	1	3	4	2	0
2791	14	1	1	7	1	2	2	4	2	0
2792	19	2	2	7	1	2	3	4	2	0
2793	55	10	1	8	1	1	2	4	2	0
2794	57	10	1	3	1	1	3	4	2	0
2795	38	6	1	2	1	1	3	4	2	0
2796	63	11	2	3	1	1	3	4	2	0
2797	55	10	1	1	1	1	2	4	2	0
2798	69	12	1	2	1	1	3	4	2	0
2799	56	10	1	2	1	1	2	4	2	0
2800	15	2	2	7	1	2	3	4	2	0
2801	43	7	2	2	1	1	2	4	2	0
2802	66	12	1	1	1	1	3	4	2	0
2803	45	8	1	1	1	1	2	6	2	0
2804	64	11	1	4	1	1	2	2	2	0
2805	13	1	2	7	1	2	3	2	2	0
2806	35	6	1	2	1	1	4	2	2	0
2807	75	14	1	2	1	1	2	2	2	0
2808	25	4	2	7	1	2	4	2	2	0
2809	13	1	2	5	1	2	1	2	2	0
2810	25	4	1	2	1	2	2	2	2	0
2811	33	5	2	1	1	1	3	2	2	0
2812	54	9	1	3	1	1	1	2	2	0
2813	32	5	1	2	1	1	3	2	2	0
2814	11	1	2	7	1	2	2	2	2	0
2815	65	12	1	2	1	1	2	2	2	0
2816	63	11	1	1	1	1	2	2	2	0

2817	62	11	1	1	1	1	2	2	2	0
2818	33	5	1	3	1	1	3	2	2	0
2819	18	2	2	7	1	2	3	4	2	0
2820	12	1	1	7	1	2	2	4	2	0
2821	11	1	2	7	1	2	2	4	2	0
2822	16	2	2	7	1	2	3	4	2	0
2823	20	3	2	7	1	2	3	4	2	0
2824	24	3	2	2	1	1	3	4	2	0
2825	14	1	1	7	1	2	3	4	2	0
2826	23	3	1	5	1	2	1	4	2	0
2827	30	5	2	2	1	1	2	4	2	0
2828	35	6	1	3	1	1	3	4	2	0
2829	48	8	1	3	1	1	2	4	2	0
2830	43	7	1	2	1	1	2	4	2	0
2831	40	7	1	3	1	1	3	4	2	0
2832	55	10	1	2	1	1	2	4	2	0
2833	63	11	1	3	1	1	2	4	2	0
2834	13	1	2	7	1	2	3	4	2	0
2835	17	2	2	7	1	2	3	4	2	0
2836	21	3	2	7	1	2	3	4	2	0
2837	25	4	1	7	1	2	4	4	2	0
2838	77	14	1	2	1	1	2	4	2	0
2839	30	5	1	7	1	2	4	4	2	0
2840	32	5	1	2	1	2	3	4	2	0
2841	56	10	2	5	1	1	3	4	2	0
2842	56	10	1	3	1	1	3	4	2	0
2843	41	7	1	3	1	1	3	4	2	0
2844	56	10	2	2	1	1	3	4	2	0
2845	44	7	2	2	1	1	3	4	2	0
2846	45	8	1	4	1	1	3	4	2	0
2847	40	7	2	2	1	1	2	4	2	0

2848	38	6	2	2	1	1	3	4	2	0
2849	43	7	2	2	1	1	3	4	2	0
2850	46	8	2	2	1	1	3	4	2	0
2851	50	9	1	2	3	1	3	4	2	0
2852	53	9	1	4	1	1	4	4	2	0
2853	45	8	1	2	1	4	3	4	2	0
2854	82	15	2	5	1	4	2	4	2	0
2855	81	15	2	6	1	1	4	4	2	0
2856	60	11	2	1	1	1	2	4	2	0
2857	74	13	1	6	1	1	4	4	2	0
2858	32	5	1	3	1	1	3	4	2	0
2859	47	8	1	3	2	1	2	4	2	0
2860	24	3	1	7	1	2	3	4	2	0
2861	48	8	1	4	3	1	3	4	2	0
2862	40	7	1	3	1	1	2	4	2	0
2863	67	12	1	4	1	1	3	4	2	0
2864	78	14	1	2	1	1	3	4	2	0
2865	60	11	1	3	1	1	3	4	2	0
2866	61	11	1	4	1	1	4	4	2	0
2867	40	7	1	3	1	1	2	4	2	0
2868	89	15	1	1	1	1	1	4	2	0
2869	32	5	1	3	1	1	3	4	2	0
2870	30	5	1	3	1	1	3	4	2	0
2871	36	6	2	2	1	1	2	4	2	0
2872	37	6	2	1	1	1	1	4	2	0
2873	40	7	1	2	1	1	4	4	2	0
2874	81	15	2	1	1	1	2	4	2	0
2875	44	7	1	4	1	1	4	4	2	0
2876	86	15	1	5	1	1	2	4	2	0
2877	35	6	1	2	1	1	3	4	2	0
2878	60	11	2	2	1	1	2	4	2	0

2879	37	6	2	2	1	1	3	4	2	0
2880	33	5	2	3	1	1	2	4	2	0
2881	10	1	2	7	1	2	2	4	2	0
2882	20	3	1	7	1	2	3	4	2	0
2883	30	5	1	3	1	1	3	4	2	0
2884	21	3	2	7	1	2	3	4	2	0
2885	28	4	1	3	1	2	3	4	2	0
2886	25	4	1	3	1	2	3	4	2	0
2887	34	5	2	2	1	1	3	4	2	0
2888	61	11	1	2	1	1	2	4	2	0
2889	13	1	2	7	1	2	2	4	2	0
2890	85	15	1	5	1	1	2	4	2	0
2891	23	3	1	1	1	2	2	4	1	1
2892	92	15	1	3	1	1	3	4	2	0
2893	25	4	1	2	1	1	2	4	2	0
2894	80	15	2	1	1	1	2	4	2	0
2895	100	15	2	5	1	4	1	4	2	0
2896	50	9	2	1	1	1	3	4	2	0
2897	32	5	2	2	1	1	3	4	2	0
2898	44	7	1	3	1	1	2	4	2	0
2899	40	7	1	3	1	1	3	4	2	0
2900	40	7	2	1	1	1	2	4	2	0
2901	63	11	2	1	1	1	4	4	2	0
2902	36	6	1	2	1	1	3	4	2	0
2903	59	10	1	2	1	1	2	4	2	0
2904	70	13	1	3	1	1	3	4	2	0
2905	72	13	1	3	1	1	3	4	2	0
2906	18	2	2	7	1	2	3	4	2	0
2907	65	12	1	3	1	1	2	4	2	0
2908	59	10	2	1	1	1	2	4	2	0
2909	61	11	2	3	1	1	2	4	2	0

2910	28	4	1	8	1	1	3	4	2	0
2911	23	3	1	3	1	2	3	4	2	0
2912	53	9	2	3	1	1	3	4	2	0
2913	45	8	1	2	1	1	3	4	2	0
2914	60	11	1	3	1	1	2	4	2	0
2915	60	11	2	1	1	1	2	4	2	0
2916	65	12	1	1	1	1	2	4	2	0
2917	87	15	1	6	1	1	3	4	2	0
2918	75	14	1	1	1	1	2	4	2	0
2919	65	12	1	1	1	1	2	4	2	0
2920	52	9	1	4	1	1	3	4	2	0
2921	70	13	2	2	1	4	3	6	2	0
2922	65	12	1	3	1	1	2	1	2	0
2923	65	12	1	3	1	1	3	1	2	0
2924	80	15	1	3	1	1	2	1	2	0
2925	45	8	1	4	1	1	4	1	2	0
2926	62	11	1	4	1	1	4	1	2	0
2927	62	11	1	3	1	1	2	1	2	0
2928	47	8	1	2	1	1	3	1	2	0
2929	40	7	1	1	1	1	3	1	2	0
2930	45	8	1	3	1	1	3	1	2	0
2931	60	11	2	1	1	4	2	1	2	0
2932	63	11	1	3	1	1	2	1	2	0
2933	50	9	1	2	1	1	2	6	2	0
2934	45	8	1	3	1	1	3	6	2	0
2935	27	4	1	3	1	1	3	6	2	0
2936	22	3	2	7	1	2	4	1	2	0
2937	25	4	1	7	1	2	4	1	2	0
2938	31	5	2	8	1	2	4	1	2	0
2939	40	7	2	2	1	1	3	1	2	0
2940	65	12	2	2	1	1	2	1	2	0

2941	11	1	1	7	1	2	2	1	2	0
2942	26	4	1	7	1	2	4	1	2	0
2943	56	10	2	3	1	1	2	6	2	0
2944	61	11	1	2	1	1	2	6	2	0
2945	31	5	1	2	3	1	2	1	2	0
2946	50	9	2	3	1	1	2	6	2	0
2947	36	6	1	3	1	1	3	6	2	0
2948	60	11	1	3	1	1	3	6	2	0
2949	40	7	2	2	1	1	2	1	2	0
2950	28	4	2	7	1	2	3	1	2	0
2951	22	3	1	7	1	2	3	1	2	0
2952	40	7	1	3	1	1	3	6	2	0
2953	15	2	2	7	1	2	3	6	2	0
2954	53	9	2	2	1	1	3	1	2	0
2955	65	12	2	3	1	1	3	1	2	0
2956	60	11	1	1	1	1	2	1	2	0
2957	50	9	1	2	1	1	3	1	2	0
2958	35	6	2	3	1	1	3	1	2	0
2959	42	7	2	2	1	1	2	1	2	0
2960	23	3	2	7	1	2	3	1	2	0
2961	47	8	2	2	1	1	3	1	2	0
2962	55	10	1	2	1	1	2	1	2	0
2963	45	8	2	2	1	1	2	1	2	0
2964	60	11	2	2	1	1	2	1	2	0
2965	75	14	1	2	1	1	2	1	2	0
2966	67	12	1	2	1	4	3	1	2	0
2967	62	11	2	2	1	1	2	1	2	0
2968	24	3	1	7	1	2	3	1	2	0
2969	41	7	2	3	1	1	3	1	2	0
2970	53	9	1	3	1	4	2	1	2	0
2971	39	6	2	3	1	2	2	1	2	0

2972	29	4	1	7	1	2	4	1	2	0
2973	75	14	1	3	3	1	2	6	2	0
2974	47	8	2	2	1	1	3	6	2	0
2975	65	12	2	3	1	1	3	6	2	0
2976	41	7	2	4	1	1	3	1	2	0
2977	54	9	1	4	1	1	4	1	2	0
2978	10	1	1	7	1	2	2	1	2	0
2979	33	5	2	3	1	4	2	1	2	0
2980	54	9	1	3	3	1	2	1	2	0
2981	43	7	2	2	1	1	2	1	2	0
2982	98	15	1	5	1	1	2	1	2	0
2983	67	12	2	2	1	1	2	1	2	0
2984	13	1	1	7	1	2	2	1	2	0
2985	19	2	1	7	1	2	3	1	2	0
2986	53	9	1	8	1	1	3	1	2	0
2987	46	8	2	2	1	1	3	1	2	0
2988	13	1	2	7	1	2	2	1	2	0
2989	10	1	1	7	1	2	2	1	2	0
2990	16	2	1	7	1	2	3	1	2	0
2991	50	9	2	2	1	1	2	1	2	0
2992	68	12	2	2	1	4	2	1	2	0
2993	29	4	2	7	1	1	4	1	2	0
2994	81	15	1	2	3	1	3	1	2	0
2995	69	12	2	2	1	1	3	1	2	0
2996	63	11	2	1	3	1	3	1	2	0
2997	49	8	2	2	2	4	1	1	2	0
2998	55	10	1	3	2	1	2	1	2	0
2999	62	11	1	4	1	1	3	1	2	0
3000	52	9	1	4	1	1	4	1	2	0
3001	42	7	2	2	2	1	3	1	2	0
3002	41	7	1	3	1	1	3	1	2	0

3003	43	7	2	4	1	1	4	1	2	0
3004	31	5	2	2	1	1	3	1	2	0
3005	49	8	2	6	1	2	4	1	2	0
3006	54	9	1	8	2	1	3	1	2	0
3007	69	12	1	6	1	1	3	1	2	0
3008	30	5	2	2	1	1	3	1	2	0
3009	91	15	1	5	1	4	2	1	2	0
3010	41	7	2	2	3	1	3	1	2	0
3011	48	8	2	2	1	1	3	1	2	0
3012	60	11	2	2	1	4	2	1	2	0
3013	51	9	1	3	1	4	2	1	2	0
3014	35	6	1	3	1	1	3	1	2	0
3015	40	7	1	2	1	1	3	1	2	0
3016	50	9	1	3	3	1	3	1	2	0
3017	26	4	2	3	1	1	2	1	2	0
3018	85	15	1	5	3	1	2	1	2	0
3019	64	11	2	2	1	1	3	1	2	0
3020	48	8	2	8	1	1	3	1	2	0
3021	58	10	1	3	1	1	3	1	2	0
3022	33	5	1	2	1	1	3	1	2	0
3023	28	4	2	2	1	1	3	1	2	0
3024	41	7	1	4	1	1	4	1	2	0
3025	12	1	1	7	1	2	2	1	2	0
3026	40	7	1	2	1	1	3	1	2	0
3027	28	4	1	3	1	2	3	1	2	0
3028	72	13	1	5	1	1	2	3	2	0
3029	60	11	2	2	1	1	2	1	1	1
3030	62	11	2	2	1	1	3	1	2	0
3031	53	9	2	2	1	4	3	1	2	0
3032	22	3	2	7	1	2	3	1	2	0
3033	25	4	1	2	1	1	3	1	2	0

3034	47	8	2	4	1	1	4	6	2	0
3035	51	9	1	2	1	1	3	6	2	0
3036	60	11	1	2	1	1	3	6	2	0
3037	56	10	1	2	1	1	2	4	2	0
3038	40	7	1	2	1	1	3	6	2	0
3039	25	4	1	8	1	2	3	4	2	0
3040	64	11	2	2	1	4	3	4	2	0
3041	86	15	1	5	1	1	2	4	2	0
3042	71	13	2	6	1	1	3	4	2	0
3043	53	9	2	3	1	1	2	4	2	0
3044	42	7	2	2	1	4	3	4	2	0
3045	63	11	2	2	1	4	3	4	2	0
3046	24	3	1	7	1	2	4	4	2	0
3047	65	12	1	3	1	1	2	4	2	0
3048	23	3	2	7	1	2	3	4	2	0
3049	51	9	1	3	1	1	2	4	2	0
3050	14	1	2	7	1	2	2	4	2	0
3051	41	7	1	3	1	1	2	4	2	0
3052	62	11	2	2	1	4	2	4	2	0
3053	58	10	2	2	1	1	2	4	2	0
3054	35	6	1	3	1	1	2	4	2	0
3055	14	1	1	7	1	2	3	4	2	0
3056	64	11	1	2	1	1	2	4	2	0
3057	55	10	2	2	1	1	1	4	2	0
3058	22	3	1	7	1	2	4	4	2	0
3059	62	11	2	5	1	4	2	4	2	0
3060	33	5	1	3	1	1	2	4	2	0
3061	23	3	2	7	1	2	3	4	2	0
3062	11	1	2	7	1	2	2	4	2	0
3063	13	1	1	7	1	2	3	4	2	0
3064	14	1	2	7	1	2	3	4	2	0

3065	35	6	2	2	1	1	2	4	2	0
3066	35	6	2	2	1	4	3	4	2	0
3067	58	10	2	2	1	1	2	4	2	0
3068	43	7	1	2	1	1	2	4	2	0
3069	13	1	1	7	1	2	2	4	2	0
3070	80	15	1	5	1	1	2	4	2	0
3071	12	1	2	7	1	2	2	4	2	0
3072	43	7	2	2	1	1	3	4	2	0
3073	89	15	1	6	1	4	4	4	2	0
3074	63	11	1	3	1	1	2	4	2	0
3075	55	10	2	2	1	1	3	4	2	0
3076	53	9	1	5	3	1	2	4	2	0
3077	68	12	2	2	1	1	2	4	2	0
3078	62	11	1	3	1	1	2	4	2	0
3079	64	11	1	3	1	1	2	4	2	0
3080	70	13	1	2	1	1	2	4	2	0
3081	23	3	2	7	1	2	4	4	2	0
3082	53	9	2	5	1	1	2	4	2	0
3083	61	11	2	1	1	1	3	4	2	0
3084	33	5	1	3	1	1	2	4	2	0
3085	75	14	2	1	1	1	2	4	2	0
3086	24	3	1	7	1	2	3	6	2	0
3087	41	7	1	2	1	1	3	6	2	0
3088	37	6	2	2	1	1	3	6	2	0
3089	14	1	1	7	1	2	3	1	2	0
3090	60	11	2	1	1	1	3	1	2	0
3091	74	13	1	2	1	1	3	1	2	0
3092	60	11	1	2	1	1	3	1	2	0
3093	90	15	1	2	1	1	2	1	2	0
3094	40	7	1	2	1	1	2	1	2	0
3095	81	15	1	1	1	1	1	1	2	0

3096	25	4	2	8	1	2	3	1	2	0
3097	10	1	2	7	1	2	2	1	2	0
3098	50	9	1	8	1	1	3	3	2	0
3099	69	12	2	2	1	1	3	3	2	0
3100	50	9	2	2	1	1	2	3	2	0
3101	18	2	1	7	1	2	3	3	2	0
3102	45	8	2	2	1	1	3	3	2	0
3103	13	1	1	7	1	2	3	3	2	0
3104	15	2	2	7	1	2	3	3	2	0
3105	54	9	1	1	1	1	2	3	2	0
3106	36	6	2	1	1	1	2	3	2	0
3107	62	11	1	6	1	1	4	6	2	0
3108	49	8	2	2	1	1	3	6	2	0
3109	40	7	2	2	1	1	3	3	2	0
3110	35	6	1	3	1	1	3	3	2	0
3111	30	5	2	2	1	1	3	3	2	0
3112	14	1	2	7	1	2	3	3	2	0
3113	40	7	1	2	1	1	2	3	2	0
3114	13	1	1	7	1	2	2	3	2	0
3115	33	5	2	3	1	1	3	3	2	0
3116	59	10	1	2	1	1	3	3	2	0
3117	63	11	1	2	1	1	2	3	2	0
3118	19	2	1	7	1	2	3	3	2	0
3119	51	9	2	2	1	1	2	3	2	0
3120	15	2	1	7	1	2	3	3	2	0
3121	88	15	1	1	3	1	2	3	2	0
3122	11	1	1	7	1	2	2	3	2	0
3123	51	9	2	2	1	1	3	3	2	0
3124	68	12	1	3	1	1	2	3	2	0
3125	14	1	1	7	1	2	3	6	2	0
3126	45	8	1	3	1	1	3	6	2	0

3127	53	9	1	4	1	1	4	3	2	0
3128	40	7	1	3	1	1	3	3	2	0
3129	31	5	2	4	1	1	4	3	2	0
3130	18	2	1	7	1	2	3	3	2	0
3131	10	1	1	7	1	2	2	3	2	0
3132	26	4	2	2	1	4	3	3	2	0
3133	62	11	1	3	1	1	2	3	2	0
3134	68	12	1	3	1	1	2	3	2	0
3135	88	15	1	2	1	4	2	3	2	0
3136	43	7	1	3	1	1	2	3	2	0
3137	36	6	1	3	1	1	3	3	2	0
3138	42	7	2	3	1	4	3	3	2	0
3139	31	5	1	2	1	1	4	6	2	0
3140	40	7	1	4	1	1	4	6	2	0
3141	45	8	1	2	1	1	2	6	2	0
3142	50	9	1	3	1	1	2	3	2	0
3143	50	9	1	3	1	1	2	3	2	0
3144	70	13	1	3	1	1	2	3	2	0
3145	30	5	1	3	1	1	2	3	2	0
3146	80	15	1	1	1	1	2	3	2	0
3147	70	13	1	1	1	1	3	3	2	0
3148	67	12	1	3	1	1	2	3	2	0
3149	23	3	2	7	1	2	3	3	2	0
3150	69	12	1	2	1	1	3	3	2	0
3151	30	5	1	3	1	1	3	3	2	0
3152	57	10	1	3	1	1	2	3	2	0
3153	50	9	1	2	1	1	3	3	2	0
3154	20	3	2	7	1	2	3	3	2	0
3155	51	9	1	2	1	1	4	3	2	0
3156	73	13	1	2	1	1	2	3	2	0
3157	60	11	1	3	1	1	2	3	2	0

3158	13	1	1	7	1	2	3	3	2	0
3159	37	6	2	1	1	1	3	3	2	0
3160	76	14	1	2	1	1	3	3	2	0
3161	68	12	1	3	1	1	2	3	2	0
3162	56	10	2	2	1	1	2	6	2	0
3163	60	11	1	3	1	1	3	6	2	0
3164	15	2	2	7	1	2	3	3	2	0
3165	12	1	1	7	1	2	2	3	2	0
3166	20	3	2	7	1	2	3	3	2	0
3167	23	3	1	7	1	2	3	2	2	0
3168	30	5	2	2	1	1	2	3	2	0
3169	60	11	1	3	1	1	2	3	2	0
3170	80	15	1	2	1	1	2	3	2	0
3171	60	11	1	3	1	1	2	3	2	0
3172	40	7	2	3	1	1	3	3	2	0
3173	56	10	1	2	1	1	2	6	2	0
3174	26	4	1	3	1	1	3	6	2	0
3175	38	6	2	2	1	1	2	6	2	0
3176	36	6	2	2	1	1	2	6	2	0
3177	45	8	2	3	1	1	2	6	2	0
3178	50	9	2	2	1	4	1	6	2	0
3179	42	7	1	3	1	1	2	6	2	0
3180	31	5	2	2	1	1	3	6	2	0
3181	13	1	2	7	1	2	2	6	2	0
3182	38	6	2	2	1	1	2	6	2	0
3183	11	1	1	7	1	2	2	6	2	0
3184	70	13	1	3	1	1	2	3	2	0
3185	34	5	1	3	1	1	3	3	2	0
3186	15	2	1	7	1	2	3	6	2	0
3187	23	3	2	3	1	1	2	6	2	0
3188	55	10	1	3	1	1	2	6	2	0

3189	60	11	1	3	1	1	2	3	2	0
3190	36	6	1	3	1	1	2	3	2	0
3191	77	14	2	2	1	4	2	3	2	0
3192	72	13	1	5	3	4	2	3	2	0
3193	46	8	1	2	1	1	2	3	2	0
3194	40	7	1	2	1	4	2	3	2	0
3195	37	6	2	2	1	1	2	3	2	0
3196	42	7	1	4	1	1	3	3	2	0
3197	82	15	2	1	1	4	1	3	2	0
3198	66	12	1	3	1	1	2	3	2	0
3199	65	12	2	1	1	4	2	3	2	0
3200	46	8	2	2	1	4	3	3	2	0
3201	29	4	1	3	1	1	3	3	2	0
3202	79	14	1	3	1	1	3	3	2	0
3203	41	7	1	2	1	1	3	3	2	0
3204	31	5	1	2	1	2	3	3	2	0
3205	29	4	1	2	1	2	3	3	2	0
3206	61	11	1	5	1	1	2	3	2	0
3207	79	14	2	5	1	4	1	3	2	0
3208	28	4	1	3	1	2	3	3	2	0
3209	71	13	1	3	1	1	3	3	2	0
3210	33	5	1	3	1	1	3	3	2	0
3211	78	14	2	5	1	4	3	3	2	0
3212	34	5	2	2	1	1	3	3	2	0
3213	41	7	2	2	1	1	3	3	2	0
3214	48	8	2	2	1	1	3	3	2	0
3215	89	15	2	2	1	4	3	3	2	0
3216	70	13	1	3	1	1	3	3	2	0
3217	41	7	1	2	1	1	3	3	2	0
3218	17	2	1	7	1	2	3	3	2	0
3219	17	2	1	7	1	2	3	3	2	0

3220	57	10	2	3	1	1	3	3	2	0
3221	87	15	2	5	1	4	1	3	2	0
3222	46	8	2	2	1	1	2	3	2	0
3223	62	11	2	2	1	1	3	3	2	0
3224	17	2	2	7	1	2	3	3	2	0
3225	57	10	1	3	1	2	2	3	2	0
3226	39	6	1	2	1	1	2	4	2	0
3227	63	11	1	1	1	1	3	4	2	0
3228	82	15	2	6	1	1	3	4	2	0
3229	55	10	2	1	1	1	3	4	2	0
3230	51	9	1	4	1	1	4	4	2	0
3231	84	15	2	6	1	1	2	4	2	0
3232	36	6	1	2	1	1	3	4	2	0
3233	38	6	1	3	1	1	3	4	2	0
3234	68	12	1	3	1	1	3	4	2	0
3235	30	5	1	3	1	1	2	4	2	0
3236	74	13	1	2	1	1	3	4	2	0
3237	68	12	2	2	1	1	2	4	2	0
3238	78	14	1	1	1	1	2	4	2	0
3239	58	10	1	1	1	1	2	4	2	0
3240	38	6	1	1	1	1	3	4	2	0
3241	57	10	1	4	1	1	3	4	2	0
3242	60	11	1	8	1	1	4	4	2	0
3243	24	3	2	2	1	1	2	4	2	0
3244	30	5	2	2	1	1	2	4	2	0
3245	48	8	1	3	1	1	3	4	2	0
3246	67	12	1	3	1	1	2	4	2	0
3247	62	11	2	1	1	1	2	4	2	0
3248	73	13	1	3	1	1	2	4	2	0
3249	36	6	2	2	1	1	3	4	2	0
3250	45	8	2	4	1	1	4	4	2	0

3251	47	8	2	2	1	1	2	4	2	0
3252	34	5	1	3	1	1	3	4	2	0
3253	37	6	1	2	1	1	3	4	2	0
3254	30	5	2	2	1	1	3	4	2	0
3255	58	10	2	1	1	1	3	4	2	0
3256	65	12	1	4	1	1	2	4	2	0
3257	43	7	2	2	1	1	3	4	2	0
3258	40	7	1	2	1	1	3	4	2	0
3259	34	5	1	3	1	1	3	4	2	0
3260	35	6	1	3	1	1	3	4	2	0
3261	41	7	1	3	1	1	1	4	2	0
3262	45	8	1	3	1	1	3	4	2	0
3263	30	5	1	2	1	1	4	4	2	0
3264	35	6	1	3	1	1	3	4	2	0
3265	60	11	1	3	1	1	3	4	2	0
3266	81	15	1	4	1	1	3	4	2	0
3267	43	7	1	3	1	1	2	4	2	0
3268	70	13	2	1	1	1	2	4	2	0
3269	35	6	1	2	1	1	2	4	2	0
3270	37	6	1	2	1	1	2	4	2	0
3271	41	7	1	2	1	1	2	4	2	0
3272	40	7	1	3	1	1	3	4	2	0
3273	57	10	1	3	1	1	2	4	2	0
3274	45	8	1	8	1	1	3	4	2	0
3275	51	9	1	8	1	1	4	4	2	0
3276	35	6	1	3	1	1	2	4	2	0
3277	41	7	1	2	1	1	3	4	2	0
3278	75	14	1	2	1	1	2	4	2	0
3279	50	9	1	3	1	1	2	4	2	0
3280	53	9	1	3	1	1	2	4	2	0
3281	62	11	1	3	1	1	2	4	2	0

3282	45	8	1	3	1	1	3	4	2	0
3283	52	9	2	2	1	1	2	4	2	0
3284	31	5	2	4	1	1	4	4	2	0
3285	60	11	2	2	1	4	3	4	2	0
3286	58	10	1	3	1	1	3	4	2	0
3287	56	10	1	1	1	1	2	4	2	0
3288	56	10	2	1	1	4	2	4	2	0
3289	45	8	1	3	1	1	3	4	2	0
3290	14	1	1	7	1	2	3	4	2	0
3291	55	10	1	4	1	1	4	4	2	0
3292	58	10	1	6	1	1	4	4	2	0
3293	70	13	1	6	1	1	2	4	2	0
3294	53	9	1	2	1	1	3	4	2	0
3295	42	7	2	1	1	1	2	4	2	0
3296	65	12	1	3	1	1	2	4	2	0
3297	27	4	1	3	1	2	2	4	2	0
3298	72	13	1	5	1	1	2	4	2	0
3299	66	12	1	6	1	1	4	4	2	0
3300	56	10	1	4	1	1	3	4	2	0
3301	54	9	1	3	1	1	3	4	2	0
3302	63	11	1	2	1	1	3	4	2	0
3303	32	5	2	1	1	1	3	4	2	0
3304	22	3	2	7	1	2	3	4	2	0
3305	13	1	1	7	1	2	2	4	2	0
3306	35	6	1	2	1	1	3	4	2	0
3307	55	10	1	2	1	1	3	4	2	0
3308	53	9	1	3	1	3	3	4	2	0
3309	38	6	2	4	1	1	4	4	2	0
3310	86	15	1	3	1	1	3	4	2	0
3311	63	11	2	2	1	1	3	2	2	0
3312	35	6	2	3	1	1	2	2	2	0

3313	55	10	1	3	1	1	3	2	2	0
3314	65	12	2	2	1	1	3	2	2	0
3315	60	11	1	1	1	1	3	2	2	0
3316	45	8	1	3	1	1	2	2	2	0
3317	23	3	1	7	1	2	3	2	2	0
3318	34	5	2	2	1	1	3	2	2	0
3319	48	8	1	2	1	1	3	4	2	0
3320	18	2	1	7	1	2	3	4	2	0
3321	35	6	1	3	1	1	2	4	2	0
3322	10	1	2	7	1	2	2	2	2	0
3323	16	2	1	7	1	2	3	2	2	0
3324	68	12	1	1	1	1	1	2	2	0
3325	50	9	1	3	1	1	3	2	2	0
3326	62	11	2	1	1	4	1	2	2	0
3327	65	12	1	2	1	1	3	2	2	0
3328	65	12	1	4	1	1	4	2	2	0
3329	56	10	1	3	1	1	3	2	2	0
3330	54	9	2	1	1	1	2	2	2	0
3331	58	10	1	4	1	1	4	2	2	0
3332	50	9	1	2	1	1	2	2	2	0
3333	55	10	1	1	1	1	2	2	2	0
3334	57	10	1	4	1	1	4	2	2	0
3335	63	11	1	3	1	1	3	4	2	0
3336	55	10	1	2	1	1	2	4	2	0
3337	35	6	2	2	1	2	3	4	2	0
3338	52	9	1	3	1	1	2	4	2	0
3339	47	8	2	1	1	1	2	4	2	0
3340	35	6	2	4	1	2	4	4	2	0
3341	28	4	1	2	1	2	2	4	2	0
3342	20	3	2	7	1	2	3	4	2	0
3343	45	8	2	2	1	3	3	4	2	0

3344	34	5	1	2	1	1	2	6	2	0
3345	47	8	1	2	1	1	3	6	2	0
3346	33	5	2	2	1	1	2	6	2	0
3347	31	5	2	2	1	1	3	6	2	0
3348	30	5	1	4	1	1	3	2	2	0
3349	38	6	2	4	1	1	3	2	2	0
3350	80	15	2	5	1	4	3	2	2	0
3351	18	2	1	7	1	2	3	2	2	0
3352	11	1	2	7	1	2	2	2	2	0
3353	13	1	1	7	1	2	3	2	2	0
3354	19	2	2	7	1	2	3	2	2	0
3355	56	10	1	4	1	1	4	2	2	0
3356	58	10	2	1	1	4	2	2	2	0
3357	41	7	1	1	3	1	2	6	2	0
3358	39	6	2	1	3	1	1	6	2	0
3359	67	12	1	1	1	1	2	6	2	0
3360	30	5	1	2	1	1	3	6	2	0
3361	26	4	1	2	1	1	3	1	2	0
3362	53	9	1	2	1	1	3	6	2	0
3363	42	7	1	2	1	1	3	6	2	0
3364	29	4	1	2	1	1	3	1	2	0
3365	40	7	1	2	1	1	3	6	2	0
3366	41	7	1	2	1	1	3	6	2	0
3367	30	5	1	2	1	1	3	3	2	0
3368	49	8	1	1	3	1	2	6	2	0
3369	60	11	1	1	1	1	1	6	2	0
3370	37	6	1	2	1	1	2	6	2	0
3371	50	9	1	2	1	1	2	6	2	0
3372	60	11	1	2	1	1	2	6	2	0
3373	45	8	1	2	1	1	3	6	2	0
3374	29	4	1	2	1	1	3	6	2	0

3375	55	10	1	3	1	4	2	6	2	0
3376	50	9	2	3	1	1	1	6	2	0
3377	54	9	2	2	1	4	1	4	2	0
3378	39	6	1	2	1	1	2	4	2	0
3379	64	11	1	1	1	4	2	4	2	0
3380	57	10	2	1	1	1	2	4	2	0
3381	56	10	1	1	1	1	1	4	2	0
3382	38	6	1	2	1	1	3	4	2	0
3383	52	9	1	2	1	1	4	4	2	0
3384	40	7	1	4	1	1	3	4	2	0
3385	49	8	1	4	1	1	3	4	2	0
3386	52	9	2	3	1	1	2	4	2	0
3387	47	8	2	3	1	4	2	4	2	0
3388	47	8	1	2	1	1	3	4	2	0
3389	42	7	2	4	1	1	3	4	2	0
3390	38	6	1	4	1	1	4	4	2	0
3391	48	8	1	3	1	1	3	4	2	0
3392	42	7	2	2	1	1	3	4	2	0
3393	45	8	1	2	1	1	3	4	2	0
3394	38	6	2	2	1	1	3	4	2	0
3395	60	11	1	3	1	1	3	4	2	0
3396	50	9	1	2	1	1	3	4	2	0
3397	30	5	1	2	1	1	2	4	2	0
3398	60	11	1	6	1	1	4	4	2	0
3399	57	10	1	3	1	1	2	4	2	0
3400	28	4	2	3	1	1	2	4	2	0
3401	45	8	2	2	1	4	3	4	2	0
3402	28	4	2	3	1	1	2	4	2	0
3403	58	10	2	1	1	1	2	4	2	0
3404	30	5	1	3	1	2	2	4	2	0
3405	58	10	1	1	1	1	3	4	2	0

3406	30	5	2	2	1	1	3	4	2	0
3407	45	8	1	3	1	1	2	4	2	0
3408	38	6	2	3	1	3	3	4	2	0
3409	38	6	1	3	1	1	3	4	2	0
3410	20	3	1	3	1	2	3	4	2	0
3411	50	9	1	3	1	1	2	4	2	0
3412	51	9	2	2	1	4	1	4	2	0
3413	45	8	1	3	1	1	1	4	2	0
3414	55	10	1	2	1	1	1	4	2	0
3415	35	6	1	2	1	1	2	4	2	0
3416	50	9	1	2	1	2	2	4	2	0
3417	39	6	1	2	1	1	3	4	2	0
3418	48	8	2	3	1	4	2	2	2	0
3419	72	13	1	3	1	1	2	2	2	0
3420	53	9	2	2	1	1	3	2	2	0
3421	31	5	2	2	1	1	3	2	2	0
3422	57	10	1	4	1	1	3	2	2	0
3423	50	9	1	3	1	1	2	2	2	0
3424	56	10	2	5	3	1	3	2	2	0
3425	35	6	1	2	1	1	3	2	2	0
3426	55	10	1	3	1	1	3	2	2	0
3427	50	9	2	1	1	4	2	2	2	0
3428	15	2	1	7	1	2	3	2	2	0
3429	13	1	2	7	1	2	3	2	2	0
3430	61	11	1	1	3	1	1	2	2	0
3431	48	8	1	3	1	1	3	2	2	0
3432	57	10	1	1	1	1	2	2	2	0
3433	56	10	1	2	1	1	3	2	2	0
3434	21	3	1	3	1	2	3	2	2	0
3435	18	2	2	7	1	2	3	2	2	0
3436	68	12	1	1	1	1	2	2	2	0

3437	13	1	1	7	1	2	2	2	2	0
3438	65	12	1	3	1	3	3	2	2	0
3439	36	6	1	3	1	1	3	2	2	0
3440	58	10	2	4	1	4	3	2	2	0
3441	68	12	1	2	1	1	2	2	2	0
3442	48	8	1	3	1	1	3	2	2	0
3443	53	9	1	2	1	1	3	2	2	0
3444	40	7	1	2	1	1	2	2	2	0
3445	32	5	2	1	1	1	2	2	2	0
3446	50	9	2	2	1	2	2	2	2	0
3447	49	8	1	3	1	1	3	2	2	0
3448	40	7	2	1	1	4	3	2	2	0
3449	32	5	1	2	1	1	1	2	2	0
3450	35	6	2	1	1	1	2	2	2	0
3451	45	8	2	1	1	1	2	6	2	0
3452	52	9	1	2	1	1	2	6	2	0
3453	32	5	1	2	1	1	3	2	2	0
3454	45	8	1	2	1	1	2	2	2	0
3455	15	2	1	7	1	2	3	2	2	0
3456	13	1	1	7	1	2	3	2	2	0
3457	45	8	2	2	1	1	3	6	2	0
3458	45	8	1	2	1	1	2	2	2	0
3459	35	6	1	2	1	1	3	2	2	0
3460	48	8	1	2	1	1	3	2	2	0
3461	40	7	2	2	1	1	3	2	2	0
3462	65	12	1	3	1	1	3	2	2	0
3463	53	9	2	2	1	4	3	2	2	0
3464	57	10	1	3	1	1	3	2	2	0
3465	45	8	1	1	1	1	2	2	2	0
3466	45	8	1	2	1	1	1	2	2	0
3467	45	8	2	2	1	1	2	2	2	0

3468	55	10	1	2	1	1	2	6	2	0
3469	45	8	1	1	1	1	2	2	2	0
3470	36	6	2	2	1	1	3	2	2	0
3471	34	5	2	8	1	1	3	2	2	0
3472	32	5	1	2	1	2	3	2	2	0
3473	30	5	1	3	1	2	3	2	2	0
3474	12	1	2	7	1	2	2	1	2	0
3475	55	10	1	8	3	1	3	2	2	0
3476	53	9	1	3	1	1	3	2	2	0
3477	49	8	1	3	1	1	3	2	2	0
3478	69	12	2	8	1	1	3	2	2	0
3479	57	10	2	2	1	1	3	2	2	0
3480	50	9	2	4	1	1	4	2	2	0
3481	52	9	1	3	1	1	2	2	2	0
3482	19	2	2	7	1	2	3	2	2	0
3483	16	2	2	7	1	2	3	2	2	0
3484	40	7	1	1	1	1	3	2	2	0
3485	33	5	1	3	1	1	3	2	2	0
3486	39	6	2	2	1	4	3	2	2	0
3487	41	7	2	2	1	1	2	2	2	0
3488	10	1	2	7	1	2	2	6	2	0
3489	17	2	1	7	1	2	3	2	2	0
3490	16	2	1	7	1	2	3	2	2	0
3491	14	1	2	7	1	2	3	2	2	0
3492	11	1	1	7	1	2	2	2	2	0
3493	10	1	2	7	1	2	2	2	2	0
3494	19	2	2	7	1	2	3	2	2	0
3495	19	2	1	7	1	2	3	2	2	0
3496	17	2	1	7	1	2	3	2	2	0
3497	16	2	1	7	1	2	3	2	2	0
3498	13	1	2	7	1	2	3	2	2	0

3499	18	2	2	7	1	2	3	2	2	0
3500	17	2	1	7	1	2	3	2	2	0
3501	70	13	1	8	1	1	4	2	2	0
3502	43	7	1	2	1	1	2	4	2	0
3503	57	10	1	2	1	1	2	6	2	0
3504	15	2	2	7	1	2	3	4	2	0
3505	17	2	1	7	1	2	3	4	2	0
3506	15	2	1	7	1	2	3	4	2	0
3507	13	1	1	7	1	2	3	4	2	0
3508	18	2	2	7	1	2	3	4	2	0
3509	72	13	2	2	1	1	4	4	2	0
3510	50	9	1	2	1	1	3	4	2	0
3511	33	5	2	4	1	1	4	4	2	0
3512	37	6	1	2	1	1	4	4	2	0
3513	75	14	1	6	1	1	4	4	2	0
3514	55	10	2	2	1	1	3	6	2	0
3515	17	2	1	7	1	2	3	4	2	0
3516	23	3	1	3	1	2	3	4	2	0
3517	10	1	2	7	1	2	2	4	2	0
3518	12	1	1	3	1	2	2	4	2	0
3519	29	4	2	2	1	1	3	4	2	0
3520	37	6	2	2	1	1	3	4	2	0
3521	14	1	2	7	1	2	3	4	2	0
3522	18	2	2	7	1	2	3	4	2	0
3523	28	4	2	8	1	1	3	4	2	0
3524	15	2	1	7	1	2	3	4	2	0
3525	10	1	1	7	1	2	2	4	2	0
3526	19	2	2	8	1	2	3	4	2	0
3527	12	1	2	7	1	2	3	4	2	0
3528	17	2	1	7	1	2	3	4	2	0
3529	10	1	2	7	1	2	2	4	2	0

3530	36	6	2	2	1	1	3	4	2	0
3531	63	11	2	2	1	1	3	4	2	0
3532	76	14	2	5	1	1	3	4	2	0
3533	98	15	1	5	1	1	2	4	2	0
3534	62	11	2	4	1	1	4	4	2	0
3535	21	3	2	7	1	2	4	6	2	0
3536	11	1	1	7	1	2	2	1	2	0
3537	67	12	1	1	1	1	2	1	2	0
3538	82	15	1	1	1	1	2	1	2	0
3539	44	7	1	2	1	1	3	1	2	0
3540	47	8	1	3	1	1	3	3	2	0
3541	60	11	2	1	1	1	3	1	2	0
3542	66	12	1	1	1	1	2	1	2	0
3543	33	5	1	1	1	1	2	1	2	0
3544	96	15	1	5	1	1	2	1	2	0
3545	70	13	1	1	1	1	2	1	2	0
3546	62	11	1	1	1	1	3	1	2	0
3547	65	12	1	2	1	1	3	1	2	0
3548	54	9	1	1	1	1	4	1	2	0
3549	50	9	2	2	1	2	3	1	2	0
3550	58	10	1	3	1	1	3	1	2	0
3551	38	6	1	1	1	1	2	1	2	0
3552	36	6	1	2	1	2	4	4	2	0
3553	50	9	2	2	1	1	1	4	2	0
3554	65	12	1	3	1	1	3	4	2	0
3555	70	13	2	2	1	1	2	4	2	0
3556	80	15	1	1	1	1	2	4	2	0
3557	56	10	1	2	1	1	2	4	2	0
3558	34	5	1	2	1	1	3	4	2	0
3559	32	5	1	1	1	1	4	4	2	0
3560	34	5	1	2	1	1	3	4	2	0

3561	60	11	2	2	1	1	1	4	2	0
3562	28	4	1	2	1	1	3	4	2	0
3563	47	8	2	1	1	1	3	4	2	0
3564	12	1	1	7	1	2	3	1	2	0
3565	52	9	1	2	1	1	3	1	2	0
3566	56	10	1	4	1	1	4	1	2	0
3567	48	8	1	8	1	1	3	1	2	0
3568	57	10	1	1	1	1	2	1	2	0
3569	39	6	1	8	1	1	4	1	2	0
3570	66	12	1	2	1	1	2	1	2	0
3571	30	5	2	8	1	2	4	1	2	0
3572	72	13	2	6	1	1	4	1	2	0
3573	27	4	1	3	1	1	3	1	2	0
3574	28	4	1	2	1	1	3	1	2	0
3575	52	9	2	2	1	1	3	1	2	0
3576	63	11	1	3	1	1	2	1	2	0
3577	55	10	2	1	1	1	3	1	2	0
3578	39	6	1	2	1	1	4	1	2	0
3579	54	9	2	4	1	1	4	4	2	0
3580	65	12	1	6	1	1	3	4	2	0
3581	33	5	1	3	1	1	3	4	2	0
3582	52	9	1	3	1	1	3	4	2	0
3583	27	4	1	3	1	1	3	4	2	0
3584	22	3	1	8	1	2	3	4	2	0
3585	55	10	2	4	1	1	4	4	2	0
3586	20	3	1	7	1	2	3	4	2	0
3587	10	1	1	7	1	2	2	4	2	0
3588	70	13	2	1	1	1	2	4	2	0
3589	57	10	1	1	1	1	3	4	2	0
3590	40	7	2	2	1	1	3	4	2	0
3591	69	12	2	1	1	1	1	4	2	0

3592	22	3	1	7	1	2	3	4	2	0
3593	56	10	2	4	1	1	4	4	2	0
3594	60	11	1	8	1	1	4	4	2	0
3595	28	4	1	7	1	2	4	4	2	0
3596	24	3	2	7	1	2	4	4	2	0
3597	65	12	2	2	1	4	2	6	2	0
3598	60	11	2	2	1	1	3	1	2	0
3599	12	1	2	7	1	2	2	1	2	0
3600	67	12	2	2	1	1	3	2	2	0
3601	71	13	1	2	1	1	4	2	2	0
3602	18	2	1	7	1	2	3	4	2	0
3603	12	1	1	7	1	2	2	4	2	0
3604	50	9	1	4	1	1	4	4	2	0
3605	56	10	1	2	1	1	3	4	2	0
3606	56	10	1	4	1	1	4	4	2	0
3607	38	6	1	3	1	1	3	6	2	0
3608	32	5	2	2	1	1	3	6	2	0
3609	47	8	2	2	1	1	2	6	2	0
3610	50	9	1	3	1	1	3	6	2	0
3611	55	10	1	2	1	1	3	6	2	0
3612	46	8	2	2	1	1	4	6	2	0
3613	29	4	1	2	1	1	3	1	2	0
3614	33	5	1	8	1	2	4	1	2	0
3615	23	3	1	8	1	2	3	1	2	0
3616	56	10	1	8	1	1	3	1	2	0
3617	15	2	1	7	1	2	3	6	2	0
3618	13	1	1	7	1	2	3	6	2	0
3619	11	1	1	7	1	2	2	6	2	0
3620	40	7	1	3	1	1	3	4	2	0
3621	30	5	2	3	1	1	2	4	2	0
3622	27	4	1	2	1	2	3	4	2	0

3623	10	1	1	7	1	2	2	4	2	0
3624	15	2	1	7	1	2	3	4	2	0
3625	13	1	1	7	1	2	3	4	2	0
3626	12	1	1	7	1	2	2	4	2	0
3627	18	2	1	7	1	2	3	4	2	0
3628	11	1	2	7	1	2	2	4	2	0
3629	16	2	1	7	1	2	3	4	2	0
3630	54	9	2	3	1	1	3	4	2	0
3631	58	10	1	3	3	1	2	4	2	0
3632	60	11	1	3	3	1	2	4	2	0
3633	53	9	1	3	1	1	3	4	2	0
3634	36	6	1	3	1	1	3	4	2	0
3635	29	4	2	3	1	1	3	4	2	0
3636	60	11	1	3	3	1	2	4	2	0
3637	53	9	2	8	1	1	3	4	2	0
3638	26	4	1	8	1	2	3	4	2	0
3639	20	3	1	8	1	2	3	4	2	0
3640	17	2	1	7	1	2	3	4	2	0
3641	14	1	1	7	1	1	3	4	2	0
3642	11	1	1	7	1	2	2	4	2	0
3643	21	3	1	7	1	2	3	4	2	0
3644	60	11	2	2	1	4	2	4	2	0
3645	35	6	1	3	1	1	2	4	2	0
3646	18	2	1	7	1	2	3	4	2	0
3647	45	8	2	8	1	1	4	4	2	0
3648	52	9	1	8	1	1	4	4	2	0
3649	17	2	1	7	1	2	3	4	2	0
3650	11	1	1	7	1	2	2	4	2	0
3651	50	9	1	2	1	1	3	4	2	0
3652	15	2	1	7	1	2	3	4	2	0
3653	10	1	2	7	1	2	2	4	2	0

3654	58	10	1	3	1	1	2	4	2	0
3655	48	8	2	2	1	1	3	4	2	0
3656	16	2	1	7	1	2	3	4	2	0
3657	12	1	2	7	1	2	2	4	2	0
3658	10	1	2	7	1	2	2	4	2	0
3659	13	1	1	7	1	2	3	4	2	0
3660	62	11	1	2	1	1	3	4	2	0
3661	58	10	2	2	1	1	2	4	2	0
3662	29	4	2	4	1	1	4	4	2	0
3663	59	10	1	3	1	1	3	4	2	0
3664	56	10	2	2	1	1	2	4	2	0
3665	63	11	1	1	1	1	2	4	2	0
3666	43	7	2	2	1	1	3	4	2	0
3667	50	9	1	3	1	1	3	4	2	0
3668	46	8	2	2	1	4	2	4	2	0
3669	38	6	1	3	1	1	4	4	2	0
3670	38	6	2	2	1	1	3	4	2	0
3671	69	12	1	1	1	1	2	4	2	0
3672	36	6	1	3	1	2	3	4	2	0
3673	42	7	1	2	1	1	3	4	2	0
3674	31	5	2	2	1	1	3	4	2	0
3675	38	6	1	2	1	1	3	4	2	0
3676	39	6	2	1	1	1	3	4	2	0
3677	43	7	2	2	1	1	3	4	2	0
3678	46	8	1	2	1	1	3	4	2	0
3679	69	12	2	1	1	4	2	1	2	0
3680	38	6	1	2	1	1	3	1	2	0
3681	38	6	1	3	1	1	3	1	2	0
3682	62	11	1	1	1	1	2	1	2	0
3683	38	6	1	2	1	1	3	1	2	0
3684	29	4	1	3	1	2	3	1	2	0

3685	37	6	1	2	1	1	2	1	2	0
3686	35	6	1	2	1	2	1	1	2	0
3687	42	7	1	4	1	1	3	1	2	0
3688	49	8	2	2	1	4	2	1	2	0
3689	58	10	1	2	1	1	3	1	2	0
3690	27	4	1	7	1	2	4	1	2	0
3691	67	12	1	1	1	1	2	1	2	0
3692	25	4	1	8	1	2	2	1	2	0
3693	28	4	1	2	1	2	3	1	2	0
3694	38	6	1	2	1	1	3	1	2	0
3695	32	5	1	2	1	1	3	1	2	0
3696	72	13	1	1	1	1	3	1	2	0
3697	62	11	2	1	1	1	2	1	2	0
3698	42	7	2	2	1	4	2	1	2	0
3699	38	6	1	3	1	1	3	6	2	0
3700	32	5	2	2	1	1	2	6	2	0
3701	24	3	1	3	1	1	2	6	2	0
3702	29	4	2	2	1	1	3	1	2	0
3703	37	6	1	3	1	1	3	1	2	0
3704	45	8	1	3	1	1	2	1	2	0
3705	30	5	2	3	1	1	2	1	2	0
3706	35	6	2	3	1	1	2	6	2	0
3707	42	7	1	2	1	1	2	1	2	0
3708	45	8	1	3	1	1	3	1	2	0
3709	69	12	1	3	1	1	2	1	2	0
3710	65	12	2	5	1	1	1	1	2	0
3711	23	3	1	8	1	2	3	1	2	0
3712	20	3	1	7	1	2	3	1	2	0
3713	20	3	1	2	1	2	3	1	2	0
3714	30	5	1	2	1	1	3	1	2	0
3715	16	2	1	7	1	2	3	1	2	0

3716	24	3	1	7	1	2	4	1	2	0
3717	27	4	1	7	1	2	4	1	2	0
3718	25	4	1	2	1	2	3	1	2	0
3719	24	3	1	4	1	2	4	1	2	0
3720	27	4	1	2	1	1	3	1	2	0
3721	38	6	1	2	1	1	3	1	2	0
3722	61	11	1	2	1	1	3	6	2	0
3723	46	8	1	2	1	1	3	1	2	0
3724	53	9	2	2	1	1	3	6	2	0
3725	38	6	1	3	1	1	3	6	2	0
3726	28	4	2	2	1	1	3	6	2	0
3727	31	5	2	1	1	1	2	6	2	0
3728	48	8	1	2	1	1	3	6	2	0
3729	10	1	1	7	1	2	2	6	2	0
3730	43	7	1	4	1	1	3	1	2	0
3731	40	7	1	3	1	1	3	1	2	0
3732	38	6	1	2	1	1	3	1	2	0
3733	38	6	1	2	1	1	3	1	2	0
3734	38	6	1	2	1	1	2	1	2	0
3735	44	7	1	2	1	1	3	6	2	0
3736	38	6	2	2	1	1	3	6	2	0
3737	41	7	1	2	1	1	3	1	2	0
3738	38	6	2	1	1	1	3	1	2	0
3739	58	10	1	1	1	1	2	6	2	0
3740	37	6	1	2	1	1	3	1	2	0
3741	62	11	1	3	1	1	3	1	2	0
3742	37	6	2	2	1	1	3	1	2	0
3743	28	4	1	7	1	2	4	6	2	0
3744	38	6	2	1	1	1	3	1	2	0
3745	58	10	1	1	1	1	3	1	2	0
3746	55	10	1	1	1	1	2	1	2	0

3747	65	12	2	4	1	1	4	1	2	0
3748	45	8	2	2	1	1	2	1	2	0
3749	75	14	1	1	1	1	1	1	2	0
3750	30	5	1	2	1	1	3	1	2	0
3751	65	12	1	1	1	1	1	1	2	0
3752	50	9	1	1	1	1	2	1	2	0
3753	35	6	1	2	1	1	2	1	2	0
3754	50	9	2	2	1	1	2	1	2	0
3755	45	8	1	1	1	1	3	1	2	0
3756	11	1	1	7	1	2	2	6	2	0
3757	41	7	1	1	1	1	3	6	2	0
3758	38	6	2	2	1	1	3	6	2	0
3759	28	4	1	2	1	2	3	6	2	0
3760	18	2	1	7	1	2	3	6	2	0
3761	69	12	1	2	1	1	2	1	2	0
3762	10	1	1	7	1	2	2	6	2	0
3763	16	2	1	7	1	2	3	1	2	0
3764	16	2	1	2	1	2	3	1	2	0
3765	26	4	1	7	1	1	4	1	2	0
3766	12	1	1	7	1	2	3	1	2	0
3767	20	3	1	7	1	2	4	1	2	0
3768	12	1	1	7	1	2	2	1	2	0
3769	19	2	1	7	1	2	3	6	2	0
3770	30	5	1	4	1	2	3	4	2	0
3771	59	10	1	3	1	1	2	4	2	0
3772	63	11	1	1	1	1	2	4	2	0
3773	79	14	1	3	1	1	2	4	2	0
3774	14	1	2	7	1	2	3	4	2	0
3775	21	3	1	3	1	2	3	4	2	0
3776	52	9	2	3	1	1	3	4	2	0
3777	54	9	2	2	1	1	3	4	2	0

3778	22	3	2	7	1	2	3	4	2	0
3779	32	5	1	3	1	2	3	4	2	0
3780	47	8	1	5	3	2	1	4	2	0
3781	62	11	1	2	1	4	3	4	2	0
3782	40	7	1	2	1	1	3	4	2	0
3783	42	7	2	2	1	1	3	4	2	0
3784	45	8	2	2	1	1	3	4	2	0
3785	32	5	2	2	1	1	3	4	2	0
3786	60	11	2	5	1	1	2	4	2	0
3787	22	3	2	7	1	2	3	4	2	0
3788	13	1	1	7	1	2	2	4	2	0
3789	47	8	1	2	1	1	2	4	2	0
3790	33	5	2	2	1	1	3	4	2	0
3791	41	7	1	2	1	1	2	4	2	0
3792	61	11	2	2	1	1	2	4	2	0
3793	16	2	1	7	1	2	3	4	2	0
3794	17	2	2	7	1	2	3	4	2	0
3795	11	1	2	7	1	2	2	4	2	0
3796	13	1	1	7	1	2	2	4	2	0
3797	15	2	2	7	1	2	3	4	2	0
3798	14	1	1	7	1	2	3	3	2	0
3799	10	1	2	7	1	2	2	1	2	0
3800	17	2	1	7	1	2	3	1	2	0
3801	56	10	2	6	1	4	2	1	2	0
3802	43	7	2	2	1	4	2	1	2	0
3803	40	7	1	2	1	1	3	1	2	0
3804	34	5	2	2	1	1	3	1	2	0
3805	42	7	1	2	1	1	2	1	2	0
3806	44	7	1	2	1	1	3	1	2	0
3807	34	5	2	2	1	1	2	1	2	0
3808	30	5	1	2	1	2	3	1	2	0

3809	68	12	1	6	1	1	3	1	2	0
3810	23	3	2	8	1	1	3	1	2	0
3811	20	3	1	7	1	2	3	1	2	0
3812	32	5	2	2	1	1	3	1	2	0
3813	21	3	2	7	1	2	3	1	2	0
3814	53	9	2	2	1	1	3	1	2	0
3815	12	1	1	7	1	2	3	1	2	0
3816	16	2	2	7	1	2	3	1	2	0
3817	30	5	2	2	1	1	3	1	2	0
3818	60	11	2	2	1	1	3	1	2	0
3819	48	8	1	1	1	1	3	1	2	0
3820	29	4	1	7	1	2	4	1	2	0
3821	28	4	1	4	1	1	4	1	2	0
3822	37	6	1	2	1	1	2	1	2	0
3823	48	8	1	2	1	1	3	1	2	0
3824	32	5	1	2	5	1	3	1	2	0
3825	50	9	1	2	1	1	3	1	2	0
3826	48	8	1	3	1	1	3	1	2	0
3827	38	6	1	1	1	1	3	1	2	0
3828	37	6	2	2	1	1	3	1	2	0
3829	51	9	1	2	1	1	3	1	2	0
3830	38	6	1	2	1	1	3	6	2	0
3831	50	9	1	2	1	1	3	6	2	0
3832	51	9	2	2	1	1	3	4	2	0
3833	51	9	1	2	1	1	3	4	2	0
3834	51	9	1	2	1	1	3	4	2	0
3835	39	6	1	2	1	1	3	4	2	0
3836	51	9	1	2	1	1	3	4	2	0
3837	42	7	1	2	1	1	3	4	2	0
3838	61	11	1	1	1	1	3	4	2	0
3839	58	10	1	2	1	1	3	4	2	0

3840	41	7	1	1	1	1	2	4	2	0
3841	43	7	2	2	1	1	2	4	2	0
3842	48	8	1	2	1	1	1	4	2	0
3843	51	9	1	2	1	1	3	4	2	0
3844	56	10	1	3	1	1	2	4	2	0
3845	48	8	1	1	1	1	2	4	2	0
3846	43	7	1	2	1	1	2	4	2	0
3847	68	12	1	1	1	1	2	1	2	0
3848	44	7	1	4	1	1	4	1	2	0
3849	50	9	2	2	1	1	3	1	2	0
3850	35	6	1	4	1	1	4	1	2	0
3851	35	6	2	2	1	1	3	6	2	0
3852	65	12	2	1	1	1	2	1	2	0
3853	70	13	1	5	1	1	2	1	2	0
3854	42	7	1	2	1	1	2	6	2	0
3855	18	2	1	7	1	2	3	1	2	0
3856	18	2	2	7	1	2	3	1	2	0
3857	65	12	1	2	1	1	3	1	2	0
3858	28	4	2	2	1	1	3	1	2	0
3859	68	12	2	2	1	1	2	1	2	0
3860	74	13	1	2	1	1	3	1	2	0
3861	48	8	1	3	1	1	3	1	2	0
3862	30	5	2	2	1	1	2	1	2	0
3863	40	7	1	3	1	1	2	1	2	0
3864	70	13	1	3	1	1	2	1	2	0
3865	10	1	2	7	1	2	2	1	2	0
3866	42	7	1	2	1	1	3	1	2	0
3867	41	7	2	2	1	1	3	1	2	0
3868	48	8	2	1	1	1	3	1	2	0
3869	35	6	1	3	1	1	3	1	2	0
3870	48	8	1	2	1	1	3	1	2	0

3871	78	14	1	2	1	1	2	1	2	0
3872	45	8	1	3	1	1	2	4	2	0
3873	10	1	1	7	3	2	2	4	2	0
3874	16	2	1	7	1	2	3	4	2	0
3875	19	2	1	7	1	2	3	4	2	0
3876	16	2	1	7	1	2	3	4	2	0
3877	10	1	2	7	1	2	2	4	2	0
3878	18	2	1	7	1	2	3	4	2	0
3879	10	1	2	7	1	2	2	4	2	0
3880	60	11	2	1	1	1	3	4	2	0
3881	19	2	1	7	1	2	3	4	2	0
3882	35	6	2	1	1	1	3	4	2	0
3883	70	13	1	2	1	1	3	4	2	0
3884	53	9	2	2	1	1	2	4	2	0
3885	75	14	2	2	1	4	3	4	2	0
3886	56	10	2	1	1	1	2	4	2	0
3887	60	11	1	2	1	1	2	1	2	0
3888	58	10	2	1	1	1	3	1	2	0
3889	26	4	1	7	1	2	4	1	2	0
3890	45	8	2	1	1	1	3	1	2	0
3891	62	11	1	3	1	1	3	1	2	0
3892	53	9	1	3	1	6	3	1	2	0
3893	23	3	2	7	1	2	3	1	2	0
3894	38	6	1	2	1	1	3	1	2	0
3895	82	15	1	1	1	1	3	1	2	0
3896	21	3	1	7	1	2	4	1	2	0
3897	19	2	2	7	1	2	3	1	2	0
3898	33	5	2	2	1	1	3	1	2	0
3899	37	5	2	1	1	1	2	1	2	0
3900	74	13	1	3	1	1	3	1	2	0
3901	43	7	2	2	1	1	3	1	2	0

3902	67	12	2	1	1	1	2	1	2	0
3903	43	7	1	3	1	1	3	1	2	0
3904	30	5	2	2	1	1	2	1	2	0
3905	18	2	1	7	1	2	3	1	2	0
3906	64	11	2	2	1	4	3	1	2	0
3907	67	12	1	1	1	1	3	1	2	0
3908	68	12	1	2	1	1	3	1	2	0
3909	65	12	2	3	1	1	2	1	2	0
3910	11	1	2	7	1	2	2	1	2	0
3911	13	1	1	7	1	2	2	1	2	0
3912	19	2	2	2	1	2	3	1	2	0
3913	13	1	2	7	1	2	3	1	2	0
3914	13	1	2	7	1	2	3	1	2	0
3915	12	1	1	7	1	2	2	1	2	0
3916	14	1	1	7	1	2	3	1	2	0
3917	24	3	1	7	1	1	3	1	2	0
3918	32	5	1	4	1	2	4	1	2	0
3919	31	5	2	2	1	1	2	1	2	0
3920	56	10	2	2	1	1	2	1	2	0
3921	45	8	2	3	1	1	2	1	2	0
3922	54	9	1	2	1	1	3	1	2	0
3923	70	13	1	6	1	1	4	1	2	0
3924	48	8	1	2	1	1	3	1	2	0
3925	82	15	2	6	1	1	3	1	2	0
3926	52	9	1	2	1	1	3	1	2	0
3927	55	10	2	2	1	1	3	1	2	0
3928	42	7	2	2	1	1	3	1	2	0
3929	75	14	2	3	1	4	2	4	2	0
3930	35	6	1	3	1	1	3	4	2	0
3931	17	2	1	7	1	2	3	4	2	0
3932	12	1	2	7	1	2	3	4	2	0

3933	11	1	2	7	1	2	2	4	2	0
3934	78	14	2	1	1	4	2	4	2	0
3935	71	13	1	1	1	1	2	4	2	0
3936	19	2	1	7	1	2	3	4	2	0
3937	14	1	1	7	1	2	3	4	2	0
3938	17	2	2	7	1	2	3	4	2	0
3939	12	1	1	7	1	2	3	4	2	0
3940	28	4	2	2	1	1	3	4	2	0
3941	42	7	1	3	1	1	3	1	2	0
3942	31	5	2	2	5	1	3	1	2	0
3943	48	8	1	2	1	1	3	1	2	0
3944	42	7	2	2	1	1	2	1	2	0
3945	65	12	2	2	1	1	2	2	2	0
3946	30	5	2	5	1	2	1	1	2	0
3947	48	8	2	2	1	1	3	1	2	0
3948	60	11	1	2	1	1	3	1	2	0
3949	65	12	1	2	1	1	3	1	2	0
3950	50	9	2	5	1	1	2	1	2	0
3951	11	1	1	7	1	2	2	1	2	0
3952	12	1	1	7	1	2	2	1	2	0
3953	50	9	2	2	1	1	3	1	2	0
3954	55	10	2	2	1	1	3	1	2	0
3955	57	10	2	2	1	1	3	1	2	0
3956	51	9	2	3	1	1	2	1	2	0
3957	12	1	1	7	1	2	2	1	2	0
3958	19	2	1	7	1	2	3	1	2	0
3959	11	1	1	7	1	2	2	1	2	0
3960	10	1	1	7	1	2	2	1	2	0
3961	65	12	1	3	1	1	2	1	2	0
3962	46	8	2	1	1	1	4	1	2	0
3963	72	13	1	3	1	1	2	1	2	0

3964	40	7	1	3	1	1	3	1	2	0
3965	69	12	2	2	1	1	2	1	2	0
3966	39	6	1	2	1	1	3	1	2	0
3967	25	4	2	3	1	1	2	1	2	0
3968	43	7	2	2	1	4	2	1	2	0
3969	31	5	1	3	1	1	3	1	2	0
3970	40	7	1	3	1	4	2	1	2	0
3971	29	4	2	2	1	1	2	1	2	0
3972	93	15	1	5	3	1	2	1	2	0
3973	30	5	2	2	1	1	2	1	2	0
3974	81	15	2	5	1	1	1	1	2	0
3975	13	1	1	7	1	2	2	1	2	0
3976	11	1	2	7	1	2	2	1	2	0
3977	13	1	1	7	1	2	2	1	2	0
3978	19	2	1	7	1	2	3	1	2	0
3979	13	1	1	7	1	2	2	1	2	0
3980	33	5	1	3	1	1	2	1	2	0
3981	68	12	2	2	1	1	2	1	2	0
3982	23	3	1	7	1	2	3	1	2	0
3983	34	5	1	3	1	1	2	1	2	0
3984	65	12	1	3	1	1	2	1	2	0
3985	70	13	1	3	1	1	1	1	2	0
3986	48	8	1	3	1	1	3	1	2	0
3987	53	9	1	3	1	1	2	1	2	0
3988	66	12	1	2	1	1	3	1	2	0
3989	35	6	1	2	1	1	3	1	2	0
3990	57	10	1	1	1	1	3	1	2	0
3991	45	8	2	2	1	1	3	1	2	0
3992	17	2	1	7	1	2	3	1	2	0
3993	30	5	2	2	1	1	3	1	2	0
3994	17	2	1	7	1	2	3	1	2	0

3995	49	8	1	2	1	1	3	1	2	0
3996	42	7	1	1	1	1	2	1	2	0
3997	26	4	1	2	1	6	3	1	2	0
3998	65	12	1	2	1	1	3	1	2	0
3999	56	10	2	1	1	1	3	1	2	0
4000	64	11	1	3	1	1	2	1	2	0
4001	43	7	1	1	1	1	2	1	2	0
4002	45	8	1	2	1	1	3	1	2	0
4003	40	7	2	2	1	1	3	1	2	0
4004	70	13	1	2	1	1	3	1	2	0
4005	13	1	2	7	1	2	2	1	2	0
4006	48	8	1	2	1	1	2	1	2	0
4007	47	8	2	2	1	1	2	1	2	0
4008	77	14	1	2	1	1	2	1	2	0
4009	81	15	2	2	1	4	3	1	2	0
4010	17	2	2	7	1	2	3	1	2	0
4011	76	14	1	1	1	1	3	1	2	0
4012	68	12	1	6	1	1	2	1	2	0
4013	87	15	2	2	1	4	3	1	2	0
4014	66	12	2	2	1	1	2	1	2	0
4015	35	6	2	2	1	1	3	1	2	0
4016	57	10	2	2	1	1	2	1	2	0
4017	56	10	1	2	1	1	3	1	2	0
4018	30	5	1	3	1	1	3	6	2	0
4019	61	11	2	3	1	1	3	6	2	0
4020	68	12	1	3	1	1	2	6	2	0
4021	45	8	1	2	1	1	3	1	2	0
4022	49	8	1	2	1	1	3	1	2	0
4023	58	10	2	2	1	1	3	1	2	0
4024	40	7	1	2	1	1	4	6	2	0
4025	70	13	1	2	1	1	2	1	2	0

4026	41	7	1	4	1	1	4	1	2	0
4027	43	7	1	3	1	1	2	1	2	0
4028	76	14	1	2	1	1	2	1	2	0
4029	38	6	1	3	1	1	2	1	2	0
4030	41	7	1	3	1	1	2	1	2	0
4031	62	11	2	2	1	1	2	1	2	0
4032	50	9	2	1	1	1	2	3	2	0
4033	65	12	1	3	1	1	3	3	2	0
4034	10	1	1	7	1	2	2	1	2	0
4035	44	7	1	2	1	4	3	4	2	0
4036	66	12	2	1	1	1	3	4	2	0
4037	22	3	1	7	1	2	3	4	2	0
4038	10	1	1	7	1	2	2	4	2	0
4039	28	4	1	2	1	1	2	4	2	0
4040	31	5	1	1	1	1	2	4	2	0
4041	11	1	2	7	1	2	2	4	2	0
4042	19	2	2	7	1	2	3	4	2	0
4043	17	2	1	7	1	2	3	4	2	0
4044	33	5	2	2	1	2	2	4	2	0
4045	58	10	1	3	1	1	2	4	2	0
4046	60	11	2	2	1	1	2	4	2	0
4047	34	5	2	2	1	1	2	4	2	0
4048	68	12	1	1	1	1	2	4	2	0
4049	61	11	2	1	1	1	2	4	2	0
4050	23	3	1	7	1	1	3	4	2	0
4051	30	5	1	3	1	1	2	4	2	0
4052	16	2	1	7	1	2	3	4	2	0
4053	12	1	2	7	1	2	2	4	2	0
4054	37	6	2	2	1	1	2	4	2	0
4055	55	10	1	1	1	1	3	4	2	0
4056	19	2	1	7	1	2	3	4	2	0

4057	55	10	2	1	1	1	3	4	2	0
4058	15	2	2	7	1	2	3	4	2	0
4059	55	10	1	3	1	1	1	4	2	0
4060	65	12	1	1	1	1	2	4	2	0
4061	69	12	1	1	1	1	3	4	2	0
4062	43	7	1	3	1	1	3	4	2	0
4063	64	11	1	1	1	1	2	4	2	0
4064	74	13	2	1	1	4	4	4	2	0
4065	61	11	2	2	1	1	2	4	2	0
4066	47	8	2	3	1	1	2	4	2	0
4067	54	9	2	1	1	1	3	4	2	0
4068	68	12	1	1	1	1	2	4	2	0
4069	34	5	1	2	1	1	3	4	2	0
4070	47	8	1	1	1	1	2	4	2	0
4071	70	13	1	1	1	1	3	4	2	0
4072	16	2	1	7	1	2	3	4	2	0
4073	10	1	2	7	1	2	2	4	2	0
4074	16	2	2	7	1	2	3	4	2	0
4075	14	1	1	7	1	2	2	4	2	0
4076	13	1	1	7	1	2	2	4	2	0
4077	65	12	2	2	1	4	3	4	2	0
4078	13	1	2	7	1	2	3	4	2	0
4079	19	2	1	7	1	2	3	4	2	0
4080	16	2	2	7	1	2	3	4	2	0
4081	87	15	1	2	1	1	3	4	2	0
4082	96	15	1	1	3	1	1	4	2	0
4083	85	15	1	1	1	1	1	4	2	0
4084	72	13	1	3	1	1	4	4	2	0
4085	54	9	1	3	1	1	3	4	2	0
4086	90	15	1	1	3	1	1	4	2	0
4087	37	6	1	4	1	1	4	4	2	0

4088	57	10	1	3	1	1	3	4	2	0
4089	72	13	1	2	1	1	2	4	2	0
4090	32	5	1	4	1	1	4	4	2	0
4091	62	11	1	4	1	1	4	4	2	0
4092	60	11	1	4	1	1	4	4	2	0
4093	60	11	1	4	1	1	4	4	2	0
4094	49	8	1	4	1	1	4	4	2	0
4095	81	15	1	1	1	1	2	4	2	0
4096	62	11	1	1	1	1	4	1	2	0
4097	28	4	2	4	1	1	3	1	2	0
4098	40	7	1	1	1	1	2	1	2	0
4099	50	9	1	1	1	1	2	1	2	0
4100	30	5	2	2	1	1	4	1	2	0
4101	52	9	1	2	1	1	3	1	2	0
4102	30	5	2	8	1	1	4	1	2	0
4103	78	14	1	1	1	1	2	1	2	0
4104	50	9	1	2	1	1	3	1	2	0
4105	39	6	2	2	1	1	3	1	2	0
4106	50	9	1	1	1	1	2	1	2	0
4107	49	8	1	1	3	1	3	1	2	0
4108	40	7	2	2	1	1	2	1	2	0
4109	46	8	2	4	1	1	3	1	2	0
4110	52	9	1	3	1	1	2	1	2	0
4111	56	10	1	1	1	1	2	1	2	0
4112	65	12	1	1	1	1	2	1	2	0
4113	38	6	1	3	1	1	3	1	2	0
4114	50	9	1	1	1	1	3	1	2	0
4115	40	7	2	2	1	1	4	1	2	0
4116	30	5	2	2	1	2	4	1	2	0
4117	55	10	1	1	1	1	3	1	2	0
4118	60	11	2	2	1	1	4	1	2	0

4119	61	11	1	1	1	1	4	1	2	0
4120	50	9	1	1	1	1	3	1	2	0
4121	30	5	1	1	1	1	4	1	2	0
4122	40	7	2	2	1	1	3	1	2	0
4123	45	8	1	2	1	1	4	1	2	0
4124	39	6	2	2	1	1	4	1	2	0
4125	11	1	2	7	1	2	2	1	2	0
4126	18	2	1	7	1	2	3	1	2	0
4127	11	1	1	7	1	2	2	1	2	0
4128	13	1	2	7	1	2	2	1	2	0
4129	14	1	1	7	1	2	3	4	2	0
4130	18	2	1	7	1	2	3	4	2	0
4131	18	2	2	1	1	2	3	4	2	0
4132	31	5	2	2	1	1	2	4	2	0
4133	17	2	2	7	1	2	3	4	2	0
4134	18	2	2	7	1	2	3	4	2	0
4135	28	4	2	2	1	1	3	4	2	0
4136	36	6	2	2	1	1	4	4	2	0
4137	67	12	2	2	1	1	3	4	2	0
4138	62	11	2	2	1	1	4	4	2	0
4139	14	1	1	7	1	2	3	4	2	0
4140	21	3	1	8	1	1	2	4	2	0
4141	42	7	1	2	1	1	3	4	2	0
4142	12	1	2	7	1	2	2	4	2	0
4143	24	3	2	7	1	1	4	4	2	0
4144	41	7	2	2	1	1	3	4	2	0
4145	26	4	2	4	1	1	3	4	2	0
4146	67	12	2	2	1	1	3	4	2	0
4147	13	1	1	7	1	2	3	1	2	0
4148	12	1	2	7	1	2	2	1	2	0
4149	30	5	2	2	1	1	3	1	2	0

4150	66	12	2	8	1	1	3	1	2	0
4151	45	8	2	2	1	1	3	1	2	0
4152	58	10	2	8	1	1	2	1	2	0
4153	28	4	2	3	1	1	3	1	2	0
4154	14	1	2	7	1	2	3	1	2	0
4155	18	2	2	7	1	2	3	1	2	0
4156	11	1	1	7	1	2	2	1	2	0
4157	22	3	2	2	1	1	3	1	2	0
4158	18	2	2	7	1	2	3	1	2	0
4159	28	4	2	1	1	1	2	1	2	0
4160	61	11	1	3	1	1	3	1	2	0
4161	56	10	1	3	1	1	2	1	2	0
4162	37	6	1	3	1	2	4	1	2	0
4163	18	2	1	3	1	2	3	1	2	0
4164	42	7	1	3	1	1	2	1	2	0
4165	47	8	1	2	1	1	3	1	2	0
4166	41	7	1	2	1	1	2	1	2	0
4167	48	8	1	2	1	1	3	1	2	0
4168	41	7	2	2	1	1	2	1	2	0
4169	32	5	2	1	1	1	2	1	2	0
4170	60	11	1	3	1	1	3	1	2	0
4171	50	9	1	2	1	1	3	1	2	0
4172	51	9	1	2	1	1	3	1	2	0
4173	50	9	1	3	1	1	3	1	2	0
4174	47	8	2	2	1	1	3	1	2	0
4175	64	11	2	1	1	1	2	1	2	0
4176	34	5	2	2	1	1	3	1	2	0
4177	31	5	2	2	1	1	3	1	2	0
4178	10	1	2	7	1	2	2	1	2	0
4179	46	8	1	2	1	5	3	1	2	0
4180	18	2	1	7	1	2	3	1	2	0

4181	16	2	2	7	1	2	3	1	2	0
4182	11	1	2	7	1	2	2	1	2	0
4183	21	3	2	7	1	2	4	6	2	0
4184	14	1	1	7	1	2	3	1	2	0
4185	46	8	2	5	1	1	2	1	2	0
4186	45	8	1	2	1	1	3	1	2	0
4187	49	8	1	2	1	1	3	1	2	0
4188	40	7	2	3	1	1	3	1	2	0
4189	19	2	1	7	1	2	3	1	2	0
4190	63	11	1	2	1	4	2	1	2	0
4191	51	9	2	3	1	1	3	1	2	0
4192	10	1	2	7	1	2	2	1	2	0
4193	20	3	2	7	1	2	3	1	2	0
4194	38	6	1	2	1	1	3	1	2	0
4195	51	9	2	2	1	4	3	6	2	0
4196	30	5	1	3	1	1	3	6	2	0
4197	23	3	2	2	1	1	2	6	2	0
4198	14	1	2	7	1	2	3	1	2	0
4199	15	2	1	7	1	2	3	1	2	0
4200	17	2	1	8	1	2	3	1	2	0
4201	21	3	1	2	1	2	3	6	2	0
4202	11	1	2	7	1	2	2	1	2	0
4203	13	1	1	7	1	2	3	1	2	0
4204	31	5	1	5	1	2	1	1	2	0
4205	26	4	2	2	1	2	3	1	2	0
4206	60	10	1	4	1	1	3	1	2	0
4207	45	8	2	8	1	1	3	1	2	0
4208	21	3	2	7	1	2	4	1	2	0
4209	17	2	1	7	1	2	3	1	2	0
4210	13	1	1	7	1	2	3	1	2	0
4211	17	2	2	2	1	2	3	1	2	0

4212	30	5	2	2	1	1	3	1	2	0
4213	13	1	2	7	1	2	2	1	2	0
4214	11	1	1	7	1	2	2	6	2	0
4215	27	4	1	4	1	2	3	6	2	0
4216	34	5	2	1	1	1	3	1	2	0
4217	31	5	2	2	1	1	3	4	2	0
4218	15	2	1	7	1	2	3	6	2	0
4219	13	1	2	7	1	2	3	6	2	0
4220	10	1	2	7	1	2	2	6	2	0
4221	14	1	1	7	1	2	3	6	2	0
4222	16	2	2	7	1	2	3	6	2	0
4223	26	4	2	2	1	1	3	6	2	0
4224	12	1	1	7	1	2	2	6	2	0
4225	10	1	1	7	1	2	2	6	2	0
4226	28	4	2	2	1	1	3	1	2	0
4227	15	2	2	7	1	2	3	1	2	0
4228	13	1	2	7	1	2	3	1	2	0
4229	10	1	1	7	1	2	2	1	2	0
4230	45	8	1	2	1	1	3	5	2	0
4231	65	12	2	2	1	1	3	5	2	0
4232	38	7	1	2	1	1	3	5	2	0
4233	33	5	1	2	1	1	3	5	2	0
4234	61	11	1	1	1	1	3	5	2	0
4235	48	8	1	2	1	1	2	5	2	0
4236	65	12	2	2	1	1	2	5	2	0
4237	43	7	1	2	1	1	3	5	2	0
4238	60	11	1	2	1	1	3	5	2	0
4239	49	8	2	2	1	1	3	5	2	0
4240	54	9	1	2	3	1	3	5	2	0
4241	80	15	1	2	1	1	3	5	2	0
4242	70	13	2	5	1	1	2	5	2	0

4243	32	5	1	2	1	1	3	5	2	0
4244	14	1	1	7	1	2	3	5	2	0
4245	82	15	1	5	1	1	2	5	2	0
4246	51	9	1	2	1	1	3	5	2	0
4247	54	9	1	2	1	1	3	5	2	0
4248	52	9	1	4	1	1	4	5	2	0
4249	48	8	2	4	1	1	4	5	2	0
4250	30	5	1	4	1	2	4	5	2	0
4251	26	4	1	7	1	2	4	5	2	0
4252	42	7	1	4	1	1	4	5	2	0
4253	28	4	2	4	1	2	4	5	2	0
4254	26	4	1	2	1	2	3	5	2	0
4255	24	3	1	4	1	2	4	5	2	0
4256	34	5	2	4	1	1	4	5	2	0
4257	20	3	1	7	1	2	4	5	2	0
4258	16	2	1	7	1	2	3	5	2	0
4259	42	7	1	4	1	1	4	1	2	0
4260	19	2	1	7	1	2	4	5	2	0
4261	15	2	2	7	1	2	3	5	2	0
4262	13	1	1	7	1	2	3	5	2	0
4263	45	8	1	4	1	1	4	5	2	0
4264	60	11	2	5	1	1	2	5	2	0
4265	35	6	1	2	1	1	3	5	2	0
4266	60	11	1	2	1	1	3	5	2	0
4267	73	13	1	2	1	1	2	5	2	0
4268	82	15	1	5	1	1	2	5	2	0
4269	72	13	2	2	1	1	2	5	2	0
4270	72	13	1	5	1	1	2	5	2	0
4271	68	12	2	2	1	1	3	5	2	0
4272	28	4	1	3	1	1	3	5	2	0
4273	26	4	2	2	1	1	3	5	2	0

4274	68	12	1	1	1	1	2	5	2	0
4275	60	11	2	1	1	1	1	5	2	0
4276	70	13	1	2	1	1	2	5	2	0
4277	52	9	2	1	1	1	2	5	2	0
4278	52	9	1	1	1	1	2	5	2	0
4279	63	11	1	2	1	1	3	5	2	0
4280	76	14	1	5	1	1	3	5	2	0
4281	65	12	1	1	1	1	2	5	1	1
4282	54	9	2	1	1	1	2	5	2	0
4283	59	10	2	1	1	1	2	5	2	0
4284	70	13	2	1	1	4	2	5	2	0
4285	74	13	2	1	1	4	1	5	2	0
4286	76	14	2	2	1	4	2	5	2	0
4287	87	15	2	1	1	4	2	5	2	0
4288	63	11	1	2	1	1	3	5	2	0
4289	51	9	2	2	1	1	3	5	2	0
4290	36	6	1	2	1	1	3	5	2	0
4291	76	14	1	2	1	1	2	5	2	0
4292	68	12	2	5	1	1	3	5	2	0
4293	46	8	1	2	1	1	3	5	2	0
4294	65	12	2	1	1	1	3	5	2	0
4295	66	12	2	5	1	1	3	5	2	0
4296	96	15	1	5	1	1	3	5	2	0
4297	76	14	1	1	1	1	2	5	2	0
4298	75	14	2	1	1	1	3	5	2	0
4299	78	14	1	2	1	1	3	5	2	0
4300	65	12	2	2	1	1	3	5	2	0
4301	40	7	1	2	1	1	3	5	2	0
4302	46	8	1	2	1	1	3	5	2	0
4303	42	7	2	2	1	1	3	5	2	0
4304	18	2	2	7	1	2	3	5	2	0

4305	16	2	2	7	1	2	3	5	2	0
4306	12	1	2	7	1	2	3	5	2	0
4307	49	8	1	2	1	1	3	5	2	0
4308	43	7	2	4	1	1	4	5	2	0
4309	16	2	1	7	1	1	3	5	2	0
4310	13	1	2	7	1	2	3	5	2	0
4311	10	1	1	7	1	2	3	5	2	0
4312	58	10	1	5	1	1	3	5	2	0
4313	67	12	1	3	1	1	3	5	2	0
4314	75	14	2	5	1	4	3	5	2	0
4315	55	10	2	1	1	1	3	5	2	0
4316	66	12	2	5	1	1	3	5	2	0
4317	65	12	1	1	1	1	3	5	2	0
4318	57	10	1	4	1	1	3	5	2	0
4319	45	8	1	2	3	1	3	5	2	0
4320	40	7	1	2	1	1	3	5	2	0
4321	68	12	1	2	1	1	3	5	2	0
4322	36	6	1	2	1	1	3	4	2	0
4323	44	7	1	2	1	1	3	5	2	0
4324	46	8	1	3	1	2	3	5	2	0
4325	33	5	1	3	1	1	3	5	2	0
4326	45	8	1	2	1	1	3	5	2	0
4327	67	12	1	3	1	1	3	5	2	0
4328	63	11	2	1	1	4	3	5	2	0
4329	44	7	2	4	1	1	4	5	2	0
4330	56	10	1	2	1	1	3	5	2	0
4331	68	12	1	6	1	1	4	5	2	0
4332	53	9	2	4	1	1	4	5	2	0
4333	72	13	1	3	1	1	2	5	2	0
4334	38	6	1	2	1	1	3	5	2	0
4335	48	8	2	2	1	1	3	5	2	0

4336	54	9	1	2	1	1	3	5	2	0
4337	42	7	1	3	1	1	3	5	2	0
4338	75	14	1	5	1	1	3	5	2	0
4339	52	9	1	3	1	1	3	5	2	0
4340	51	9	1	2	1	4	2	5	2	0
4341	45	8	2	1	1	4	3	5	2	0
4342	83	15	2	5	1	4	1	5	2	0
4343	56	10	2	2	1	1	2	5	2	0
4344	62	11	1	1	1	1	2	5	2	0
4345	60	11	2	2	1	4	2	5	2	0
4346	65	12	2	1	1	4	2	5	2	0
4347	84	15	2	5	1	4	1	5	2	0
4348	75	14	2	2	1	4	1	5	2	0
4349	50	9	2	2	1	4	2	5	2	0
4350	41	7	1	3	1	1	2	5	2	0
4351	62	11	1	3	1	1	3	5	2	0
4352	90	15	2	5	1	4	1	5	2	0
4353	45	8	2	2	1	4	3	5	2	0
4354	58	10	1	2	1	1	2	5	2	0
4355	61	11	1	3	1	1	2	5	2	0
4356	42	7	1	3	1	1	2	5	2	0
4357	57	10	1	3	1	1	2	5	2	0
4358	50	9	1	4	1	1	2	5	2	0
4359	72	13	1	1	1	1	2	5	2	0
4360	35	6	2	1	1	1	2	5	2	0
4361	52	9	1	1	1	1	2	5	2	0
4362	12	1	1	7	1	2	2	5	2	0
4363	73	13	1	1	1	4	2	5	2	0
4364	14	1	1	7	1	2	2	5	2	0
4365	56	10	2	2	1	1	2	5	2	0
4366	62	11	1	1	1	1	2	5	2	0

4367	57	10	1	2	1	1	2	5	2	0
4368	10	1	1	7	1	2	2	5	2	0
4369	60	11	2	2	1	4	2	5	2	0
4370	50	9	2	1	1	2	2	5	2	0
4371	58	10	2	2	1	1	2	5	2	0
4372	12	1	1	7	1	2	2	5	2	0
4373	40	7	2	2	1	1	2	5	2	0
4374	58	10	1	2	1	1	2	5	2	0
4375	78	14	2	1	1	1	2	5	2	0
4376	50	9	2	1	1	1	1	5	2	0
4377	54	9	2	1	1	1	1	5	2	0
4378	81	15	1	2	1	1	1	5	2	0
4379	32	5	1	2	1	1	2	5	2	0
4380	72	13	2	1	1	1	1	5	2	0
4381	74	13	2	2	1	1	1	5	2	0
4382	78	14	2	2	1	1	1	5	2	0
4383	51	9	1	2	1	1	1	5	2	0
4384	74	13	2	1	1	4	1	5	2	0
4385	47	8	1	1	1	1	2	5	2	0
4386	50	9	1	2	1	1	1	5	2	0
4387	70	13	2	1	1	1	1	5	2	0
4388	58	10	1	2	1	1	2	5	2	0
4389	60	11	1	2	1	1	2	5	2	0
4390	54	9	1	2	1	1	2	5	2	0
4391	58	10	1	2	1	1	3	5	2	0
4392	77	14	2	1	1	1	1	5	2	0
4393	48	8	1	4	1	2	3	5	2	0
4394	67	12	1	1	1	1	1	5	2	0
4395	60	11	1	2	1	1	1	5	2	0
4396	56	10	1	2	1	1	1	5	2	0
4397	73	13	1	1	3	1	1	5	2	0

4398	80	15	1	1	3	1	1	5	2	0
4399	61	11	1	2	1	1	4	5	2	0
4400	56	10	2	2	1	1	1	5	2	0
4401	48	8	2	2	1	1	1	5	2	0
4402	36	6	1	3	1	1	2	5	2	0
4403	35	6	1	2	1	1	3	5	2	0
4404	40	7	2	2	1	1	3	5	2	0
4405	40	7	1	2	1	1	1	5	2	0
4406	52	9	1	2	1	1	2	5	2	0
4407	51	9	1	3	1	1	2	5	2	0
4408	47	8	1	3	1	1	2	5	2	0
4409	47	8	1	3	1	1	3	5	2	0
4410	45	8	1	3	1	1	2	5	2	0
4411	42	7	1	3	1	1	2	5	2	0
4412	32	5	1	3	1	1	1	5	2	0
4413	47	8	1	2	1	1	2	5	2	0
4414	60	11	1	2	1	1	1	5	2	0
4415	52	9	1	3	1	1	2	5	2	0
4416	65	12	1	3	1	1	2	5	2	0
4417	63	11	1	3	1	1	2	5	2	0
4418	54	9	2	1	1	4	1	5	2	0
4419	70	13	1	5	1	1	1	5	2	0
4420	32	5	1	3	1	2	2	5	2	0
4421	52	9	1	3	1	1	4	5	2	0
4422	60	11	2	3	1	4	2	5	2	0
4423	50	9	1	3	1	1	2	5	2	0
4424	45	8	1	3	1	1	3	5	2	0
4425	63	11	2	5	1	4	1	5	2	0
4426	52	9	1	3	1	1	2	5	2	0
4427	68	12	2	1	1	4	1	5	2	0
4428	58	10	1	3	1	1	2	5	2	0

4429	55	10	1	3	1	1	2	5	2	0
4430	61	11	2	1	1	4	1	5	2	0
4431	65	12	1	3	1	1	1	5	2	0
4432	37	6	1	3	1	1	2	5	2	0
4433	55	10	2	3	1	1	3	5	2	0
4434	36	6	1	3	1	1	3	5	2	0
4435	48	8	2	1	1	4	1	5	2	0
4436	47	8	1	3	1	1	3	5	2	0
4437	29	4	2	3	1	1	3	5	2	0
4438	48	8	1	4	1	1	3	5	2	0
4439	35	6	1	3	1	1	2	5	2	0
4440	45	8	1	3	1	1	3	5	2	0
4441	69	12	1	2	1	1	3	5	2	0
4442	42	7	1	2	1	1	3	5	2	0
4443	65	12	2	1	1	1	2	5	2	0
4444	83	15	2	5	1	1	1	5	2	0
4445	68	12	2	5	1	1	3	5	2	0
4446	44	7	1	2	1	1	3	5	2	0
4447	45	7	1	2	1	1	3	5	2	0
4448	70	13	1	2	1	1	2	5	2	0
4449	58	10	1	2	1	1	3	5	2	0
4450	66	12	2	5	1	1	3	5	2	0
4451	35	6	1	3	1	1	3	5	2	0
4452	46	8	1	2	1	1	3	5	2	0
4453	60	11	2	2	1	1	3	5	2	0
4454	76	14	1	5	1	1	3	5	2	0
4455	48	8	1	2	1	1	3	5	2	0
4456	38	6	1	3	1	1	3	5	2	0
4457	56	10	2	1	1	1	3	5	2	0
4458	76	14	2	5	1	1	2	5	2	0
4459	54	9	1	3	1	1	3	5	2	0

4460	46	8	1	3	1	1	3	5	2	0
4461	48	8	2	4	1	1	4	5	2	0
4462	48	8	1	2	1	1	3	5	2	0
4463	38	6	1	2	1	1	3	5	2	0
4464	52	9	1	2	1	1	3	5	2	0
4465	54	9	1	2	1	1	3	5	2	0
4466	66	12	1	2	1	1	4	5	2	0
4467	46	8	1	2	1	1	4	5	2	0
4468	60	11	2	2	1	1	3	5	2	0
4469	56	10	1	3	1	1	3	5	2	0
4470	68	12	2	1	1	1	3	5	2	0
4471	48	8	1	2	1	1	4	5	2	0
4472	49	8	1	2	1	1	4	5	2	0
4473	52	9	1	2	1	1	3	5	2	0
4474	48	8	1	1	1	1	3	5	2	0
4475	52	9	1	3	1	1	2	5	2	0
4476	62	11	1	2	1	1	3	5	2	0
4477	52	9	1	2	1	1	3	5	2	0
4478	49	8	1	3	1	1	3	5	2	0
4479	63	11	2	1	1	4	2	5	2	0
4480	75	14	2	5	1	4	2	5	2	0
4481	60	11	1	3	1	1	3	5	2	0
4482	67	12	2	1	1	4	1	5	2	0
4483	81	15	1	5	1	4	2	5	2	0
4484	50	9	2	5	1	4	1	5	2	0
4485	60	11	1	3	1	1	2	5	2	0
4486	85	15	2	5	1	4	1	5	2	0
4487	56	10	1	2	1	1	2	5	2	0
4488	73	13	2	2	1	4	2	5	2	0
4489	61	11	1	1	1	1	2	5	2	0
4490	55	10	2	3	1	1	2	5	2	0

4491	65	12	1	4	1	1	2	5	2	0
4492	78	14	1	3	1	1	2	5	2	0
4493	63	11	2	1	1	4	3	5	2	0
4494	31	5	1	3	1	1	1	5	2	0
4495	40	7	1	3	1	1	3	5	2	0
4496	42	7	1	3	1	1	3	5	2	0
4497	52	9	1	3	1	1	2	5	2	0
4498	73	13	1	5	1	1	2	5	2	0
4499	68	12	2	1	1	4	2	5	2	0
4500	60	11	1	2	1	4	3	5	2	0
4501	45	8	2	2	1	1	3	5	2	0
4502	87	15	2	1	1	4	1	5	2	0
4503	73	13	1	3	1	1	2	5	2	0
4504	80	15	2	1	1	4	1	5	2	0
4505	74	13	1	1	1	1	2	5	2	0
4506	65	12	2	2	1	1	3	5	2	0
4507	65	12	2	5	1	1	2	5	2	0
4508	65	12	1	3	1	1	3	5	2	0
4509	70	13	1	3	1	1	2	5	2	0
4510	70	13	1	1	1	1	2	5	2	0
4511	35	6	1	3	1	1	3	5	2	0
4512	72	13	2	2	1	1	2	5	2	0
4513	70	13	1	1	1	1	2	5	2	0
4514	56	10	2	2	1	1	2	5	2	0
4515	48	8	2	2	1	1	3	5	2	0
4516	55	10	2	2	1	1	3	5	2	0
4517	78	14	1	2	1	4	2	5	2	0
4518	55	10	2	2	1	1	3	5	2	0
4519	65	12	2	1	1	1	2	5	2	0
4520	50	9	2	2	1	1	3	5	2	0
4521	72	13	1	1	1	1	1	5	2	0

4522	68	12	1	3	1	1	2	5	2	0
4523	60	11	1	2	1	1	2	5	2	0
4524	62	11	2	2	1	4	2	5	2	0
4525	45	8	1	2	1	1	3	5	2	0
4526	55	10	2	2	1	1	3	5	2	0
4527	60	11	2	5	1	1	3	5	2	0
4528	56	11	2	1	1	4	2	5	2	0
4529	55	10	1	2	1	1	3	5	2	0
4530	50	9	2	4	1	1	3	5	2	0
4531	57	10	2	4	1	1	4	5	2	0
4532	45	8	1	3	1	1	2	5	2	0
4533	50	9	1	4	1	1	2	5	2	0
4534	45	8	1	3	1	1	3	5	2	0
4535	40	7	1	2	1	1	3	5	2	0
4536	58	10	1	2	1	1	3	5	2	0
4537	40	7	1	3	1	1	3	5	2	0
4538	60	11	2	2	1	1	2	5	2	0
4539	71	13	2	2	1	4	2	5	2	0
4540	45	8	1	2	1	1	3	5	2	0
4541	66	12	1	3	1	4	3	5	2	0
4542	33	5	1	2	1	1	1	5	2	0
4543	56	10	2	2	1	1	2	5	2	0
4544	55	10	1	2	1	1	3	5	2	0
4545	51	9	1	2	1	1	3	5	2	0
4546	83	15	1	6	1	1	2	5	2	0
4547	50	9	1	3	1	1	2	5	2	0
4548	32	5	1	2	1	1	3	5	2	0
4549	30	5	1	2	1	2	4	5	2	0
4550	47	8	1	2	1	1	3	5	2	0
4551	69	12	1	6	1	1	4	5	2	0
4552	56	10	1	2	1	1	3	5	2	0

4553	64	11	1	2	1	1	2	5	2	0
4554	70	13	2	1	1	4	2	5	2	0
4555	90	15	1	2	1	1	3	5	2	0
4556	60	11	2	1	1	1	2	5	2	0
4557	81	15	2	1	1	4	2	5	2	0
4558	68	12	2	1	1	4	2	5	2	0
4559	60	11	1	2	1	1	2	5	2	0
4560	55	10	2	2	1	1	3	5	2	0
4561	50	9	2	2	1	1	3	5	2	0
4562	40	7	2	2	1	1	3	5	2	0
4563	58	10	1	1	1	1	2	5	2	0
4564	69	12	1	1	1	1	3	5	2	0
4565	66	12	1	2	1	1	2	5	2	0
4566	42	7	1	3	1	1	3	5	2	0
4567	65	12	2	1	1	1	2	5	2	0
4568	56	10	2	1	1	1	2	5	2	0
4569	40	7	1	4	1	1	3	5	2	0
4570	70	13	1	2	1	1	3	5	2	0
4571	42	7	1	3	1	1	3	3	2	0
4572	25	4	1	2	1	2	2	5	2	0
4573	53	9	2	4	1	2	4	5	2	0
4574	68	12	1	5	1	1	3	5	2	0
4575	43	7	1	2	1	1	3	3	2	0
4576	37	6	1	3	1	1	3	5	2	0
4577	60	11	1	2	1	1	2	5	2	0
4578	45	8	1	2	1	1	2	5	2	0
4579	67	12	1	8	1	1	2	5	2	0
4580	38	6	1	4	1	1	3	5	2	0
4581	47	8	1	4	1	1	4	5	2	0
4582	35	6	1	2	1	2	3	5	2	0
4583	52	9	1	2	1	1	2	5	2	0

4584	67	12	2	5	1	4	2	5	2	0
4585	72	13	1	5	1	1	1	5	2	0
4586	40	7	2	3	1	1	2	5	2	0
4587	45	8	1	2	1	1	4	5	2	0
4588	34	5	1	2	1	1	3	5	2	0
4589	53	9	1	3	1	1	3	5	2	0
4590	74	13	1	5	1	1	1	5	2	0
4591	41	7	1	2	3	1	3	5	2	0
4592	36	6	2	2	1	1	3	5	2	0
4593	70	13	1	6	1	1	4	5	2	0
4594	92	15	1	5	1	1	2	5	2	0
4595	40	7	1	1	1	1	2	5	2	0
4596	85	15	1	1	1	1	2	5	2	0
4597	65	12	1	1	1	1	2	5	2	0
4598	62	11	1	2	1	1	2	5	2	0
4599	35	6	1	3	1	1	3	5	2	0
4600	30	5	2	3	1	1	3	5	2	0
4601	37	6	1	3	1	1	3	5	2	0
4602	28	4	1	2	3	2	3	5	2	0
4603	42	7	1	2	1	1	4	5	2	0
4604	30	5	1	3	1	2	2	5	2	0
4605	37	6	1	2	1	1	2	5	2	0
4606	40	7	1	2	1	2	4	5	2	0
4607	56	10	1	2	1	1	2	5	2	0
4608	52	9	1	2	1	1	3	5	2	0
4609	37	6	1	2	1	1	4	5	2	0
4610	37	6	1	2	1	1	3	5	2	0
4611	39	6	2	8	1	2	4	5	2	0
4612	38	6	1	2	1	1	3	5	2	0
4613	80	15	2	5	1	4	1	5	2	0
4614	48	8	1	2	1	1	2	5	2	0

4615	32	5	2	2	1	1	3	5	2	0
4616	72	13	1	5	1	1	2	5	2	0
4617	24	3	1	7	3	2	4	5	2	0
4618	30	5	1	2	1	1	2	5	2	0
4619	70	13	2	2	1	4	3	5	2	0
4620	92	15	1	8	3	1	2	5	2	0
4621	73	13	1	3	3	1	1	6	2	0
4622	28	4	1	3	1	1	3	5	2	0
4623	67	12	2	2	1	1	2	6	2	0
4624	50	9	1	2	1	1	2	5	2	0
4625	49	8	1	2	1	1	3	6	2	0
4626	52	9	1	2	1	1	3	6	2	0
4627	48	8	1	3	1	1	2	5	2	0
4628	56	10	2	2	1	4	2	5	2	0
4629	37	6	1	3	1	1	3	1	2	0
4630	45	8	1	3	1	1	2	6	2	0
4631	37	6	1	2	1	1	3	5	1	1
4632	47	8	2	2	1	1	3	6	2	0
4633	65	12	1	2	1	1	3	5	2	0
4634	55	10	2	4	1	4	4	5	2	0
4635	65	12	1	3	1	1	3	5	2	0
4636	66	12	1	1	3	1	2	5	2	0
4637	73	13	1	6	1	1	4	5	2	0
4638	74	13	2	6	1	4	4	5	2	0
4639	23	3	2	7	1	2	4	5	2	0
4640	27	4	1	4	1	1	4	5	2	0
4641	79	14	1	6	1	1	3	5	2	0
4642	70	13	1	3	1	1	3	5	2	0
4643	65	12	1	3	1	1	2	5	2	0
4644	28	4	1	2	1	1	3	5	2	0
4645	80	15	1	5	1	4	4	5	2	0

4646	65	12	2	2	1	4	2	5	2	0
4647	68	12	1	3	1	1	2	5	2	0
4648	53	9	2	5	1	4	3	5	2	0
4649	63	11	1	8	3	1	2	5	2	0
4650	46	8	1	4	1	1	4	5	2	0
4651	43	7	1	4	1	1	4	5	2	0
4652	40	7	1	3	1	1	3	5	2	0
4653	49	8	1	2	1	1	2	5	2	0
4654	50	9	1	2	1	1	3	6	2	0
4655	35	6	1	2	1	1	2	5	2	0
4656	32	5	1	4	1	1	4	5	2	0
4657	47	8	1	2	1	1	2	5	2	0
4658	38	6	2	3	1	1	3	5	2	0
4659	54	9	2	2	1	4	2	5	2	0
4660	60	11	1	2	1	1	3	5	2	0
4661	60	11	1	4	1	1	4	5	2	0
4662	87	15	1	1	1	1	1	5	2	0
4663	65	12	1	1	1	1	3	5	2	0
4664	75	14	1	6	1	3	4	5	2	0
4665	60	11	1	8	1	2	4	5	2	0
4666	48	8	2	3	1	1	3	5	2	0
4667	43	9	1	2	1	1	2	5	2	0
4668	50	9	1	2	1	1	2	5	2	0
4669	69	12	1	1	1	1	2	5	2	0
4670	70	13	2	1	1	4	2	5	2	0
4671	40	7	1	8	1	1	4	5	2	0
4672	75	14	2	5	1	4	2	5	2	0
4673	63	11	1	3	1	1	2	5	2	0
4674	34	5	1	3	1	1	2	5	2	0
4675	70	13	1	3	1	1	3	5	2	0
4676	50	9	1	3	1	1	3	5	2	0

4677	55	10	1	1	1	1	3	5	2	0
4678	52	9	2	2	1	1	3	5	2	0
4679	59	10	2	2	1	1	2	5	2	0
4680	49	8	1	2	1	1	3	5	2	0
4681	51	9	1	2	1	1	3	5	2	0
4682	59	10	1	2	1	1	3	5	2	0
4683	89	15	2	5	1	4	2	5	2	0
4684	81	15	2	5	1	4	2	5	2	0
4685	38	6	1	2	1	1	3	5	2	0
4686	55	10	2	2	1	1	3	5	2	0
4687	32	5	1	2	3	1	3	5	2	0
4688	72	13	1	2	1	1	3	5	2	0
4689	33	5	1	4	1	1	3	5	2	0
4690	35	6	2	2	1	1	3	5	2	0
4691	42	7	1	1	1	1	3	5	2	0
4692	65	12	1	2	1	1	2	5	2	0
4693	83	15	2	5	1	4	1	5	2	0
4694	73	13	2	1	1	1	1	5	2	0
4695	63	11	1	2	3	1	2	5	2	0
4696	42	7	1	2	1	2	2	5	2	0
4697	40	7	1	2	1	1	2	5	2	0
4698	48	8	1	1	1	1	2	5	2	0
4699	74	13	1	2	1	1	3	5	2	0
4700	70	13	1	1	1	1	3	5	2	0
4701	62	11	2	1	1	1	1	5	2	0
4702	43	7	2	1	1	1	2	5	2	0
4703	54	9	2	1	5	1	1	5	2	0
4704	56	10	1	2	1	1	3	5	2	0
4705	72	13	2	1	1	4	2	5	2	0
4706	52	9	2	2	1	1	3	5	2	0
4707	43	7	1	2	1	1	3	5	2	0

4708	39	13	1	3	1	1	2	5	2	0
4709	73	13	2	2	1	1	1	5	2	0
4710	50	9	1	3	1	1	2	5	2	0
4711	75	14	1	2	1	1	3	5	2	0
4712	58	10	2	1	1	1	3	5	2	0
4713	62	11	2	2	1	1	2	5	2	0
4714	60	11	1	2	1	1	3	5	2	0
4715	47	8	2	1	1	4	3	5	1	1
4716	60	11	2	1	1	1	2	5	2	0
4717	13	1	1	7	1	2	2	5	1	1
4718	33	5	1	5	1	2	1	5	2	0
4719	39	6	1	2	1	2	2	5	2	0
4720	41	7	1	3	1	1	3	5	2	0
4721	52	9	2	2	1	1	2	5	2	0
4722	32	5	1	3	1	1	2	5	2	0
4723	56	10	2	5	1	4	3	5	1	0
4724	23	11	2	5	1	2	2	5	1	1
4725	75	14	1	5	1	1	3	5	1	0
4726	15	2	2	7	1	2	3	5	2	0
4727	42	7	2	2	1	1	3	5	2	0
4728	16	2	1	5	1	2	2	5	1	0
4729	26	4	1	3	1	2	2	5	1	1
4730	14	1	1	5	1	2	2	5	1	0
4731	36	6	1	3	1	2	3	4	1	1
4732	21	3	1	7	1	2	4	5	2	0
4733	20	3	2	5	1	2	2	5	1	1
4734	16	2	1	7	1	2	3	5	2	0
4735	13	1	2	5	1	2	3	5	2	0
4736	25	4	2	5	1	2	3	5	2	0
4737	38	6	1	3	1	2	3	5	2	0
4738	25	4	1	5	1	2	1	5	1	0

4739	50	9	2	2	1	4	2	5	1	0
4740	48	8	2	2	1	2	3	5	2	0
4741	40	7	1	2	1	1	2	5	2	0
4742	52	9	1	1	1	2	2	2	1	1
4743	38	6	1	2	1	1	2	2	1	0
4744	33	5	2	2	1	1	3	2	1	0
4745	35	6	2	3	1	2	2	2	2	1
4746	10	1	2	5	1	2	2	2	2	0
4747	50	9	1	3	1	1	2	2	2	0
4748	48	8	1	3	1	5	2	2	1	1
4749	11	1	1	5	1	2	2	2	2	0
4750	39	6	2	4	1	1	3	2	2	0
4751	28	4	2	2	1	1	3	2	2	0
4752	10	1	2	7	1	2	2	4	2	0
4753	11	1	1	7	1	2	2	4	2	0
4754	19	2	1	7	1	2	3	4	2	0
4755	27	4	2	4	1	1	4	4	2	0
4756	46	8	2	2	1	1	2	4	2	0
4757	25	4	2	2	1	1	3	4	2	0
4758	22	3	2	2	1	1	3	4	2	0
4759	19	2	1	7	1	2	3	4	2	0
4760	14	1	2	7	1	2	3	4	2	0
4761	25	4	1	3	1	2	2	4	2	0
4762	12	1	1	7	1	2	2	4	2	0
4763	11	1	1	7	1	2	2	4	2	0
4764	16	2	1	7	1	2	3	4	2	0
4765	19	2	2	7	1	2	3	4	2	0
4766	13	1	2	7	1	2	2	4	2	0
4767	20	3	1	3	1	2	3	4	2	0
4768	31	5	1	3	1	2	3	4	2	0
4769	17	2	1	7	1	2	3	4	2	0

4770	44	7	2	3	1	2	2	4	1	1
4771	10	1	2	7	1	2	3	2	1	0
4772	46	8	1	3	1	2	3	2	1	0
4773	27	4	2	3	1	2	2	2	2	1
4774	38	6	1	1	1	2	3	2	1	0
4775	45	8	2	1	1	1	2	2	1	0
4776	13	1	1	7	1	2	2	2	1	1
4777	55	10	2	3	1	1	2	2	1	0
4778	45	8	2	1	1	1	3	2	1	0
4779	37	6	1	3	1	2	2	2	1	1
4780	20	3	2	7	1	2	3	4	2	1
4781	34	5	1	3	1	2	3	2	1	0
4782	28	4	2	5	1	2	3	2	1	0
4783	63	11	2	1	1	4	2	2	2	0
4784	20	3	1	8	1	2	2	4	1	1
4785	20	3	1	3	1	2	2	4	1	0
4786	22	3	2	7	1	2	3	4	2	0
4787	11	1	1	5	1	2	2	4	2	0
4788	41	7	2	4	1	1	4	4	2	0
4789	28	4	2	3	1	1	3	4	2	0
4790	25	4	2	2	1	1	3	4	2	0
4791	14	1	1	7	1	2	3	4	2	0
4792	17	2	1	7	1	2	3	4	2	0
4793	50	9	1	2	1	1	2	4	1	0
4794	12	1	2	7	1	2	2	4	2	1
4795	10	1	1	7	1	2	2	4	1	0
4796	48	8	1	5	1	2	1	4	2	1
4797	21	3	1	5	1	2	2	4	1	0
4798	14	1	1	5	1	2	2	4	1	0
4799	23	3	1	3	1	2	2	4	2	1
4800	17	2	1	7	1	2	3	4	1	0

4801	10	1	1	5	1	2	2	4	1	0
4802	50	9	1	1	1	1	2	4	1	0
4803	11	1	2	7	1	2	2	4	1	0
4804	21	3	2	5	1	2	2	4	1	0
4805	34	5	2	3	1	2	2	4	1	0
4806	33	5	2	8	1	2	4	4	1	1
4807	29	4	1	5	1	2	1	4	2	0
4808	39	6	1	3	1	2	2	4	1	1
4809	33	5	1	2	1	3	3	4	2	0
4810	45	8	2	5	1	2	1	4	2	0
4811	35	6	2	3	1	1	3	4	2	0
4812	38	6	2	1	1	1	2	4	2	0
4813	44	7	1	2	1	1	2	4	2	0
4814	64	11	2	1	1	4	1	4	2	0
4815	23	3	2	2	1	1	3	4	2	0
4816	17	2	1	7	1	2	3	4	2	0
4817	15	2	1	7	1	2	2	4	2	0
4818	11	1	1	7	1	2	2	6	2	0
4819	27	4	2	3	1	2	3	6	2	0
4820	22	3	1	1	1	2	2	6	2	0
4821	26	4	2	2	1	1	3	6	2	0
4822	25	4	2	3	1	2	3	6	2	0
4823	10	1	1	7	1	2	2	6	2	0
4824	22	3	2	7	1	2	3	6	2	0
4825	11	1	1	7	1	2	2	6	2	0
4826	50	9	1	3	1	5	3	6	2	0
4827	32	5	1	5	1	2	1	6	2	0
4828	50	9	1	2	1	1	3	6	2	0
4829	24	3	1	7	1	2	4	6	2	0
4830	20	3	1	2	1	2	3	6	2	0
4831	19	2	1	5	1	2	3	6	2	0

4832	19	2	1	5	1	2	3	6	2	0
4833	41	7	2	1	1	1	2	6	2	0
4834	20	3	2	5	1	2	2	6	2	0
4835	11	1	2	5	1	2	2	6	2	0
4836	18	2	1	7	1	2	2	6	2	0
4837	39	6	1	3	1	2	2	6	2	0
4838	63	11	2	1	1	1	2	6	2	0
4839	15	2	1	7	1	2	3	6	2	0
4840	11	1	1	7	1	2	2	6	2	0
4841	32	5	2	1	1	1	2	6	2	0
4842	15	2	2	5	1	2	3	6	2	0
4843	13	1	2	5	1	2	3	6	2	0
4844	31	5	2	1	1	2	2	6	2	0
4845	23	3	1	3	1	2	3	6	2	0
4846	33	5	2	2	1	2	3	6	2	0
4847	19	2	2	2	1	2	3	6	2	0
4848	29	4	1	2	1	2	3	6	2	0
4849	38	6	2	2	1	1	3	6	2	0
4850	19	2	2	7	1	2	2	6	2	0
4851	10	1	2	7	1	2	2	6	2	0
4852	17	2	2	7	1	2	2	6	2	0
4853	40	7	2	1	1	1	2	6	2	0
4854	15	2	1	7	1	2	3	6	2	0
4855	50	9	2	1	1	1	1	6	2	0
4856	44	7	1	3	1	2	3	6	2	0
4857	28	4	2	1	1	1	2	6	2	0
4858	35	6	2	2	1	1	2	6	2	0
4859	38	6	2	2	1	2	4	6	2	0
4860	44	7	1	2	1	1	3	6	2	0
4861	11	1	2	7	1	2	2	6	2	0
4862	48	8	1	2	1	1	2	6	2	0

4863	38	6	2	2	1	1	2	6	2	0
4864	12	1	1	7	1	2	2	6	2	0
4865	13	1	1	7	1	2	3	6	2	0
4866	45	8	2	4	1	3	4	6	2	0
4867	12	1	2	7	1	2	2	6	2	0
4868	38	6	1	3	1	2	4	6	2	0
4869	32	5	1	7	1	2	4	6	2	0
4870	28	4	2	7	1	2	4	6	2	0
4871	12	1	2	7	1	2	2	6	2	0
4872	60	11	2	5	1	1	2	6	2	0
4873	45	8	2	2	1	1	2	6	2	0
4874	40	7	1	3	1	1	2	6	2	0
4875	32	5	2	7	1	1	4	6	2	0
4876	35	6	2	2	1	1	2	6	2	0
4877	30	5	2	2	1	3	2	6	2	0
4878	22	3	2	7	1	2	3	6	2	0
4879	28	4	2	3	1	1	3	6	2	0
4880	30	5	1	3	1	1	3	6	2	0
4881	28	4	2	2	1	1	3	6	2	0
4882	60	11	2	1	1	1	2	6	2	0
4883	23	3	1	7	1	1	3	6	2	0
4884	10	1	2	7	1	2	2	6	2	0
4885	25	4	2	1	1	1	2	6	2	0
4886	59	10	2	2	1	1	2	6	2	0
4887	41	7	2	2	1	1	3	6	2	0
4888	31	5	2	3	1	1	3	6	2	0
4889	38	6	2	4	1	2	3	6	2	0
4890	13	1	2	7	1	2	3	6	2	0
4891	27	4	2	2	1	1	3	6	2	0
4892	35	6	1	4	1	1	2	6	2	0
4893	14	1	2	7	1	2	2	6	2	0

4894	48	8	1	2	1	1	2	6	2	0
4895	12	1	2	7	1	2	2	6	2	0
4896	40	7	2	2	1	1	3	6	2	0
4897	12	1	1	7	1	2	2	6	2	0
4898	14	1	2	7	1	2	3	6	2	0
4899	16	2	1	7	1	2	3	6	2	0
4900	18	2	1	7	1	2	3	6	2	0
4901	30	5	2	2	1	1	3	6	2	0
4902	17	2	1	7	1	2	3	6	2	0
4903	15	2	2	7	1	2	3	6	2	0
4904	10	1	2	7	1	2	2	6	2	0
4905	40	7	2	2	1	1	3	6	2	0
4906	35	6	2	4	1	1	4	6	2	0
4907	27	4	2	5	1	1	3	6	2	0
4908	38	6	2	5	1	2	3	6	2	0
4909	17	2	2	7	1	2	3	6	2	0
4910	35	6	2	2	1	4	3	6	2	0
4911	30	5	2	3	1	1	2	6	2	0
4912	28	4	2	2	1	1	3	6	2	0
4913	20	3	2	7	1	2	3	6	2	0
4914	17	2	1	7	1	2	3	6	2	0
4915	10	1	1	7	1	2	2	6	2	0
4916	28	4	2	7	1	2	4	6	2	0
4917	38	6	1	2	1	2	4	6	2	0
4918	45	8	1	2	1	1	4	6	2	0
4919	47	8	1	2	1	1	4	6	2	0
4920	11	1	2	7	1	2	2	6	2	0
4921	16	2	2	7	1	2	3	6	2	0
4922	30	5	1	3	1	1	3	6	2	0
4923	60	11	2	1	1	1	3	6	2	0
4924	16	2	2	7	1	2	3	6	2	0

4925	18	2	2	7	1	2	3	6	2	0
4926	20	3	1	3	1	2	3	6	2	0
4927	22	3	1	2	1	2	3	6	2	0
4928	15	2	2	7	1	2	3	6	2	0
4929	10	1	1	7	1	2	2	6	2	0
4930	34	5	2	2	1	1	3	6	2	0
4931	12	1	2	7	1	2	2	6	2	0
4932	32	5	2	7	1	2	3	6	2	0
4933	19	2	1	7	1	2	3	6	2	0
4934	23	3	1	1	1	2	3	6	2	0
4935	34	5	1	3	1	2	3	6	2	0
4936	24	3	1	3	1	2	3	6	2	0
4937	32	5	2	2	1	2	3	6	2	0
4938	25	3	1	2	1	2	3	6	2	0
4939	22	3	2	7	1	2	3	6	2	0
4940	15	2	1	7	1	2	3	6	2	0
4941	41	7	1	7	1	2	3	6	2	0
4942	38	6	1	3	1	2	3	6	2	0
4943	34	5	2	2	1	1	3	6	2	0
4944	38	6	1	2	1	1	3	6	2	0
4945	21	3	2	7	1	1	3	6	2	0
4946	11	1	2	7	1	2	3	6	2	0
4947	20	3	2	1	1	1	2	6	2	0
4948	23	3	2	3	1	1	3	6	2	0
4949	24	3	2	3	1	1	3	4	2	0
4950	12	1	2	7	1	1	3	4	2	0
4951	36	6	2	1	1	1	2	4	2	0
4952	32	5	2	2	1	1	2	4	2	0
4953	40	7	2	2	1	1	3	4	2	0
4954	65	12	2	2	1	1	3	4	2	0
4955	50	9	2	1	1	1	2	4	2	0

4956	33	5	1	5	1	2	3	4	2	0
4957	30	5	1	4	1	2	4	4	2	0
4958	27	4	1	3	1	2	3	4	2	0
4959	48	8	1	4	1	1	3	4	2	0
4960	27	4	1	3	1	2	3	4	2	0
4961	33	5	2	2	1	1	3	4	2	0
4962	22	3	1	7	1	2	4	4	2	0
4963	21	3	2	2	1	1	3	4	2	0
4964	53	9	2	1	1	1	3	4	2	0
4965	25	3	2	2	1	1	3	4	2	0
4966	13	1	2	7	1	2	2	4	2	0
4967	11	1	1	7	1	2	2	4	2	0
4968	31	5	2	2	1	1	2	4	2	0
4969	14	1	1	5	1	2	2	4	2	0
4970	38	6	2	8	1	1	4	4	2	0
4971	55	10	2	1	1	1	2	4	2	0
4972	70	13	2	1	1	1	1	4	2	0
4973	47	8	1	2	1	1	4	4	2	0
4974	29	4	2	2	1	1	3	4	2	0
4975	10	1	2	7	1	2	2	4	2	0
4976	36	6	2	2	1	1	3	4	2	0
4977	80	15	1	1	3	1	2	4	2	0
4978	62	11	2	1	1	1	2	4	2	0
4979	40	7	2	1	1	1	2	4	2	0
4980	50	9	2	1	1	1	2	4	2	0
4981	11	1	1	5	1	2	2	4	2	0
4982	15	2	1	5	1	2	2	4	2	0
4983	22	3	1	3	1	1	2	4	2	0
4984	11	1	2	7	1	2	2	4	2	0
4985	13	1	2	5	1	2	3	4	2	0
4986	32	5	2	1	1	1	3	4	2	0

4987	32	5	2	1	1	1	3	4	1	0
4988	27	4	2	2	1	1	3	4	2	0
4989	16	2	1	7	1	2	3	4	1	0
4990	19	2	2	7	1	2	3	4	2	0
4991	32	5	2	1	1	1	3	4	2	0
4992	16	2	2	7	1	2	3	4	2	0
4993	11	1	2	7	1	2	2	4	2	0
4994	11	1	2	7	1	2	2	4	2	0
4995	27	4	2	2	1	2	3	4	2	0
4996	27	4	2	2	1	2	3	4	2	0
4997	15	2	1	8	1	2	2	4	2	0
4998	10	1	1	7	1	2	2	4	2	0
4999	65	12	2	6	1	1	2	4	2	0
5000	63	11	1	4	1	1	3	4	2	0
5001	14	1	2	7	1	2	2	4	2	0
5002	13	1	2	7	1	2	2	4	2	0
5003	13	1	2	5	1	2	2	4	2	0
5004	40	7	2	2	1	2	2	4	2	0
5005	14	1	2	5	1	2	2	4	2	0
5006	12	1	2	5	1	2	2	4	2	0
5007	11	1	1	5	1	2	2	4	2	0
5008	22	3	1	7	1	2	3	4	2	0
5009	28	4	2	2	1	2	3	4	2	0
5010	35	6	2	2	1	1	2	4	2	0
5011	14	1	2	5	1	2	2	4	2	0
5012	10	1	1	5	1	2	2	4	2	0
5013	18	2	2	3	1	2	3	4	2	0
5014	18	2	2	7	1	2	3	4	2	0
5015	35	6	2	1	1	1	2	4	2	0
5016	70	13	2	1	1	1	1	4	2	0
5017	10	1	2	7	1	2	3	2	2	0

5018	29	4	2	5	1	1	3	4	2	0
5019	70	13	2	1	1	1	2	4	2	0
5020	52	9	1	1	3	1	2	4	2	0
5021	39	6	1	4	1	2	4	4	2	0
5022	75	14	2	1	1	1	2	4	2	0
5023	25	4	2	1	1	1	3	4	2	0
5024	14	1	1	7	1	2	3	4	2	0
5025	19	2	1	7	1	2	2	4	2	0
5026	10	1	1	7	1	2	2	4	2	0
5027	25	4	2	1	1	2	3	4	2	0
5028	50	9	1	4	1	1	3	4	2	0
5029	12	1	1	7	1	2	2	4	2	0
5030	30	5	2	1	1	1	3	4	2	0
5031	12	1	1	7	1	2	2	4	2	0
5032	10	1	1	7	1	2	2	4	2	0
5033	33	5	1	2	1	1	3	4	2	0
5034	62	11	2	1	1	1	2	4	2	0
5035	13	1	2	7	1	2	2	4	2	0
5036	20	3	2	5	1	1	3	4	2	0
5037	60	11	2	1	1	1	1	4	2	0
5038	30	5	2	5	1	2	4	4	2	0
5039	70	13	2	1	1	1	2	4	2	0
5040	35	6	2	2	1	1	3	4	2	0
5041	19	2	1	8	1	2	3	4	2	0
5042	25	4	1	2	1	2	3	4	2	0
5043	27	4	1	2	1	2	3	4	2	0
5044	22	3	2	7	1	2	4	4	2	0
5045	34	5	2	2	1	1	3	4	2	0
5046	16	2	2	5	1	2	3	4	2	0
5047	15	2	1	7	1	2	3	4	1	0
5048	17	2	2	5	1	2	2	4	2	0

5049	31	5	2	3	1	1	3	4	2	0
5050	22	3	2	2	1	1	3	4	2	0
5051	28	4	1	2	1	1	4	4	2	0
5052	26	4	2	3	1	1	3	4	2	0
5053	32	5	1	3	1	1	3	4	2	0
5054	25	4	2	1	1	1	3	4	2	0
5055	31	5	2	1	1	1	3	4	2	0
5056	30	5	2	2	1	1	3	4	2	0
5057	10	1	1	5	1	2	2	4	2	0
5058	23	3	2	1	1	1	3	4	2	0
5059	12	1	1	7	1	2	2	4	2	0
5060	10	1	1	7	1	2	2	4	2	0
5061	27	4	2	2	1	1	3	4	2	0
5062	23	3	2	2	1	1	2	4	2	0
5063	11	1	2	7	1	2	2	4	2	0
5064	12	1	2	7	1	2	2	4	2	0
5065	40	7	2	8	3	1	2	4	2	0
5066	14	1	2	7	1	2	3	4	1	0
5067	25	4	2	3	1	1	3	4	2	0
5068	25	4	2	1	1	1	3	4	2	0
5069	12	1	2	7	1	2	2	4	2	0
5070	36	6	2	3	1	1	3	4	2	0
5071	60	11	2	1	1	4	1	4	2	0
5072	48	8	1	2	1	1	4	4	2	0
5073	19	2	2	2	1	2	3	4	2	0
5074	32	5	1	3	1	2	4	4	2	0
5075	29	4	2	1	1	2	3	4	2	0
5076	18	2	1	7	1	2	3	4	2	0
5077	24	3	1	2	1	2	3	4	2	0
5078	23	3	2	2	1	1	3	4	2	0
5079	15	2	1	7	1	2	3	4	2	0

5080	11	1	1	7	1	2	3	4	2	0
5081	10	1	1	7	1	2	2	4	2	0
5082	11	1	1	7	1	2	2	4	2	0
5083	43	7	2	1	1	1	2	4	2	0
5084	10	1	1	7	1	2	2	4	2	0
5085	16	2	2	7	1	2	3	4	2	0
5086	34	5	2	4	1	1	4	4	2	0
5087	29	4	2	2	1	1	3	4	2	0
5088	21	3	2	2	1	1	4	4	2	0
5089	20	3	1	7	1	2	4	4	2	0
5090	24	3	2	2	1	1	3	4	2	0
5091	21	3	2	7	1	2	3	4	2	0
5092	15	2	2	7	1	2	2	4	1	0
5093	46	8	2	2	1	1	3	4	2	0
5094	19	2	2	7	1	2	3	4	2	0
5095	17	2	2	7	1	2	2	4	2	0
5096	12	1	1	7	1	2	2	4	2	0
5097	15	2	1	7	1	2	2	4	2	0
5098	28	4	2	7	1	2	4	4	2	0
5099	13	1	2	7	1	2	2	2	1	0
5100	22	3	1	7	1	2	3	4	2	0
5101	20	3	1	7	1	2	3	4	2	0
5102	17	2	1	7	1	2	2	4	2	0
5103	12	1	2	7	1	2	2	4	2	0
5104	15	2	1	7	1	2	3	4	2	0
5105	42	7	1	4	1	1	4	4	2	0
5106	35	6	1	3	1	1	1	4	2	0
5107	38	6	1	3	1	1	2	4	2	0
5108	21	3	2	2	1	1	3	4	2	0
5109	50	9	1	3	1	1	2	4	2	0
5110	45	8	2	3	1	1	2	4	2	0

5111	12	2	2	7	1	2	3	4	2	0
5112	26	4	2	2	1	1	3	4	2	0
5113	22	3	1	3	1	2	3	4	2	0
5114	35	6	2	3	1	1	1	4	2	0
5115	31	5	1	2	1	2	3	4	2	0
5116	14	1	2	7	1	2	2	4	2	0
5117	29	4	1	2	1	1	2	4	2	0
5118	62	11	2	1	1	1	3	4	2	0
5119	35	6	2	2	1	1	3	4	2	0
5120	47	8	1	2	1	1	2	4	2	0
5121	37	6	1	2	1	1	3	4	2	0
5122	30	5	1	2	1	1	3	4	2	0
5123	12	1	2	7	1	2	3	4	2	0
5124	13	1	2	7	1	2	2	4	2	0
5125	43	7	2	2	1	1	3	4	2	0
5126	49	8	2	1	1	1	2	4	2	0
5127	30	5	1	2	1	2	3	4	2	0
5128	18	2	2	7	1	2	3	4	2	0
5129	25	4	1	2	1	1	3	4	2	0
5130	22	3	1	2	1	2	2	4	2	0
5131	21	3	2	7	1	2	4	4	2	0
5132	10	1	2	7	1	2	2	4	2	0
5133	21	3	2	2	1	1	3	4	2	0
5134	54	9	1	2	1	1	2	4	2	0
5135	28	4	2	2	1	4	3	4	2	0
5136	72	13	2	1	1	4	2	4	2	0
5137	30	5	2	1	1	1	3	4	2	0
5138	10	1	2	7	1	2	2	4	2	0
5139	47	8	2	1	1	1	2	4	2	0
5140	38	6	2	5	1	1	3	4	2	0
5141	12	1	2	7	1	2	3	4	2	0

5142	14	1	2	7	1	2	3	4	2	0
5143	37	6	2	3	1	1	3	4	2	0
5144	12	1	2	7	1	2	3	4	2	0
5145	26	4	2	2	1	2	2	4	2	0
5146	25	4	2	2	1	2	3	6	2	0
5147	60	11	2	2	1	4	2	6	2	0
5148	32	5	1	3	1	1	3	6	2	0
5149	24	3	1	7	1	2	4	4	2	0
5150	36	6	1	3	1	2	3	4	2	0
5151	10	1	2	7	1	2	2	6	2	0
5152	35	6	2	1	1	1	2	4	2	0
5153	22	3	2	1	1	1	3	6	2	0
5154	13	1	2	7	1	2	3	4	2	0
5155	19	2	2	7	1	2	3	4	2	0
5156	17	2	1	7	1	2	3	4	2	0
5157	42	7	2	1	1	1	3	4	2	0
5158	34	5	1	3	1	1	2	4	2	0
5159	14	1	1	7	1	2	2	4	2	0
5160	27	4	2	1	1	1	3	4	2	0
5161	50	9	2	2	1	1	2	4	2	0
5162	12	1	2	7	1	2	2	4	2	0
5163	14	1	2	7	1	2	2	4	2	0
5164	27	4	1	8	1	2	4	4	2	0
5165	26	4	2	2	1	1	2	4	2	0
5166	45	8	2	2	1	1	2	4	2	0
5167	12	1	2	7	1	2	2	4	2	0
5168	22	4	2	7	1	1	4	4	2	0
5169	18	2	2	7	1	2	2	4	2	0
5170	11	1	1	7	1	2	2	4	2	0
5171	24	3	2	3	1	1	3	4	2	0
5172	38	6	2	3	1	1	3	4	2	0

5173	31	5	2	2	1	1	3	4	2	0
5174	34	5	2	1	1	1	3	4	2	0
5175	22	3	1	1	1	3	3	4	2	0
5176	12	1	2	7	1	2	3	4	2	0
5177	16	2	1	7	1	2	3	4	2	0
5178	11	1	1	5	1	2	1	4	2	0
5179	17	2	1	7	1	2	2	4	2	0
5180	17	2	1	7	1	2	3	4	2	0
5181	18	2	1	7	1	2	3	4	2	0
5182	14	1	1	7	1	2	3	4	2	0
5183	10	1	1	5	1	2	1	4	2	0
5184	30	5	2	1	1	1	2	4	2	0
5185	20	2	1	7	1	2	3	4	2	0
5186	22	3	1	4	1	2	3	4	2	0
5187	35	6	2	2	1	1	2	4	2	0
5188	15	2	2	7	1	2	2	4	2	0
5189	19	2	2	7	1	2	2	4	2	0
5190	13	1	2	7	1	2	2	4	2	0
5191	23	3	1	7	1	2	3	4	2	0
5192	21	3	1	7	1	2	3	4	2	0
5193	19	1	2	2	1	2	3	4	2	0
5194	26	2	1	2	1	2	3	4	2	0
5195	22	3	2	7	1	2	3	4	2	0
5196	79	14	1	2	1	1	2	4	2	0
5197	29	4	2	2	1	1	2	4	2	0
5198	85	15	2	1	1	4	1	4	2	0
5199	28	4	2	2	1	1	3	4	2	0
5200	100	15	2	5	1	4	1	4	2	0
5201	11	1	2	7	1	2	2	4	2	0
5202	11	1	2	7	1	2	2	4	2	0
5203	11	1	2	7	1	2	2	4	2	0

5204	14	1	1	7	1	2	3	4	2	0
5205	14	1	2	7	1	2	3	4	2	0
5206	39	6	1	4	1	1	3	4	2	0
5207	40	7	2	1	1	1	2	4	2	0
5208	35	6	2	3	1	1	3	4	2	0
5209	40	7	2	2	1	1	3	4	2	0
5210	31	5	2	2	1	1	4	4	2	0
5211	19	2	2	7	1	2	3	4	2	0
5212	13	1	1	7	1	2	3	4	2	0
5213	17	2	1	7	1	2	3	4	2	0
5214	24	3	2	7	1	2	4	4	2	0
5215	30	5	2	2	1	2	3	4	2	0
5216	27	4	2	7	1	2	3	4	2	0
5217	28	4	2	4	1	2	4	4	2	0
5218	25	4	2	7	1	2	4	4	2	0
5219	21	3	2	7	1	2	3	4	2	0
5220	34	5	1	2	1	2	4	4	2	0
5221	18	2	2	7	1	2	3	4	2	0
5222	35	6	2	1	1	1	3	4	2	0
5223	17	2	1	2	1	2	2	4	2	0
5224	15	2	2	7	1	2	2	4	2	0
5225	12	1	2	7	1	2	2	4	2	0
5226	12	1	1	5	1	2	2	4	2	0
5227	18	2	1	5	1	2	2	4	2	0
5228	23	3	2	5	1	2	3	4	2	0
5229	22	3	1	8	1	2	2	4	2	0
5230	45	8	2	1	1	1	3	4	2	0
5231	35	6	2	1	1	1	2	4	2	0
5232	57	10	2	1	1	1	3	4	2	0
5233	27	4	1	2	1	2	3	4	2	0
5234	23	3	1	2	1	2	3	4	2	0

5235	20	3	2	7	1	2	3	4	2	0
5236	40	7	2	1	1	1	3	4	2	0
5237	22	3	1	2	1	2	3	4	2	0
5238	27	4	1	2	1	2	3	4	2	0
5239	28	4	1	7	1	2	3	4	2	0
5240	14	1	2	7	1	2	2	4	2	0
5241	18	2	2	7	1	2	3	4	2	0
5242	20	3	2	4	1	1	3	4	2	0
5243	22	3	1	3	1	2	3	4	2	0
5244	20	3	1	2	1	2	2	4	2	0
5245	18	2	1	7	1	2	3	4	2	0
5246	48	8	2	1	1	1	3	4	2	0
5247	19	2	1	7	1	2	3	4	1	0
5248	50	9	2	1	1	1	3	4	2	0
5249	37	6	2	1	1	1	2	4	2	0
5250	60	11	2	1	1	1	1	4	2	0
5251	35	6	2	2	1	1	2	4	2	0
5252	11	1	2	7	1	2	1	4	2	0
5253	17	2	2	7	1	2	3	4	2	0
5254	15	2	2	7	1	2	2	4	2	0
5255	13	1	1	7	1	2	2	4	2	0
5256	11	1	1	7	1	2	2	4	2	0
5257	25	4	2	1	1	1	2	4	2	0
5258	22	3	1	2	1	2	2	4	2	0
5259	20	3	1	1	1	2	2	4	2	0
5260	18	2	2	5	1	2	2	4	2	0
5261	15	2	2	7	1	2	2	4	2	0
5262	12	1	2	7	1	2	2	4	2	0
5263	20	3	1	2	1	2	3	4	2	0
5264	20	3	1	7	1	2	3	4	2	0
5265	15	2	2	7	1	2	2	4	2	0

5266	14	1	2	7	1	2	2	4	2	0
5267	13	1	2	7	1	2	2	4	2	0
5268	65	12	1	1	1	1	2	4	2	0
5269	42	7	2	1	1	1	2	4	2	0
5270	19	2	2	7	1	2	3	4	2	0
5271	16	2	2	7	1	2	3	4	2	0
5272	14	1	2	7	1	2	3	4	2	0
5273	11	1	1	7	1	2	2	4	2	0
5274	42	7	2	2	1	1	3	4	2	0
5275	43	7	1	2	1	1	3	4	2	0
5276	12	1	2	7	1	2	2	4	2	0
5277	50	9	1	1	1	1	3	6	2	0
5278	45	8	2	1	1	1	3	6	2	0
5279	99	15	2	1	1	4	1	4	2	0
5280	22	3	1	3	1	2	3	4	2	0
5281	16	2	2	7	1	2	3	4	2	0
5282	19	2	2	7	1	2	3	4	2	0
5283	19	2	1	7	1	2	3	4	2	0
5284	11	1	1	7	1	2	2	4	2	0
5285	20	3	1	7	1	2	3	4	2	0
5286	57	10	2	1	1	1	2	4	2	0
5287	35	6	1	2	1	1	3	4	2	0
5288	21	3	2	2	1	1	3	4	2	0
5289	35	6	2	2	1	1	3	4	2	0
5290	21	3	2	8	1	1	3	4	2	0
5291	19	2	1	7	1	2	3	4	2	0
5292	17	2	2	7	1	2	2	4	2	0
5293	14	1	1	7	1	2	2	4	2	0
5294	12	1	1	7	1	2	2	4	2	0
5295	45	8	2	3	1	1	3	4	2	0
5296	23	3	1	2	1	2	3	4	2	0

5297	25	3	2	7	1	2	2	4	2	0
5298	46	8	2	2	1	1	2	4	2	0
5299	24	3	2	3	1	2	3	4	2	0
5300	17	2	2	7	1	2	2	4	2	0
5301	22	3	2	7	1	2	3	4	2	0
5302	25	3	1	7	1	2	4	4	2	0
5303	30	5	1	2	1	2	2	4	2	0
5304	53	9	2	1	1	1	2	4	2	0
5305	10	1	2	7	1	2	2	4	2	0
5306	19	2	1	7	1	2	3	4	2	0
5307	16	2	2	7	1	2	2	4	2	0
5308	34	5	2	2	1	1	3	4	2	0
5309	10	1	1	7	1	2	2	4	2	0
5310	13	1	2	7	1	2	2	4	2	0
5311	42	7	2	2	1	1	2	4	2	0
5312	21	3	2	1	1	1	2	4	2	0
5313	24	3	2	8	1	1	2	4	2	0
5314	31	5	2	1	1	1	2	4	2	0
5315	19	2	2	3	1	2	2	4	2	0
5316	10	1	2	7	1	2	2	4	2	0
5317	35	6	1	1	1	1	3	4	2	0
5318	25	3	2	2	1	1	3	4	2	0
5319	25	3	2	2	1	1	3	4	2	0
5320	11	1	1	7	1	2	2	4	2	0
5321	23	3	2	1	1	1	2	4	2	0
5322	18	2	1	7	1	2	2	4	2	0
5323	15	2	1	7	1	2	2	4	2	0
5324	10	1	1	7	1	2	2	4	2	0
5325	60	11	2	1	1	1	1	4	2	0
5326	57	10	2	1	1	1	2	4	2	0
5327	15	2	2	7	1	2	2	4	2	0

5328	15	2	2	7	1	2	2	4	2	0
5329	11	1	1	7	1	2	2	4	2	0
5330	15	2	2	7	1	2	2	4	2	0
5331	24	3	2	2	1	1	3	4	2	0
5332	11	1	2	7	1	2	2	4	2	0
5333	78	14	2	1	1	1	1	4	2	0
5334	36	6	2	1	1	1	2	4	2	0
5335	13	1	1	5	1	2	2	4	2	0
5336	12	1	2	7	1	2	3	4	2	0
5337	12	1	2	7	1	2	3	4	2	0
5338	11	1	1	7	1	2	2	4	2	0
5339	30	5	2	2	1	1	3	4	2	0
5340	13	1	1	7	1	2	2	4	2	0
5341	35	6	2	1	1	1	2	4	2	0
5342	20	3	1	7	1	2	3	4	2	0
5343	65	12	1	2	1	1	2	4	2	0
5344	24	3	2	3	1	2	3	4	2	0
5345	23	3	2	2	1	1	4	4	2	0
5346	31	5	1	2	1	1	3	6	2	0
5347	66	12	2	5	1	4	2	4	2	0
5348	36	6	2	2	1	1	3	4	2	0
5349	18	2	2	7	1	2	3	4	2	0
5350	19	2	2	7	1	2	2	4	2	0
5351	32	5	2	1	1	4	2	4	2	0
5352	12	1	2	7	1	2	2	4	2	0
5353	10	1	2	7	1	2	2	4	2	0
5354	14	1	1	7	1	2	3	4	2	0
5355	17	2	1	7	1	2	3	4	2	0
5356	10	1	1	7	1	2	2	4	2	0
5357	32	5	2	3	1	1	3	4	2	0
5358	32	5	1	1	1	1	2	4	2	0

5359	71	13	1	2	1	1	2	3	2	0
5360	33	5	2	5	1	2	3	3	2	0
5361	68	12	1	2	1	1	3	3	2	0
5362	12	1	1	7	1	2	2	3	2	0
5363	67	12	2	6	1	4	4	3	2	0
5364	45	8	1	2	1	3	4	3	2	0
5365	19	2	1	1	1	2	3	3	2	0
5366	46	8	1	2	1	1	4	3	2	0
5367	30	5	2	4	1	1	3	3	2	0
5368	37	6	1	2	1	1	3	3	2	0
5369	34	5	2	5	1	2	2	3	2	0
5370	75	14	1	6	1	1	2	3	2	0
5371	27	4	2	5	1	2	1	3	2	0
5372	61	11	2	3	1	4	3	3	2	0
5373	37	6	1	3	1	2	2	3	2	0
5374	65	12	2	1	1	1	2	3	2	0
5375	26	4	1	3	1	2	3	3	2	0
5376	29	4	2	4	1	1	3	1	1	1
5377	14	1	2	7	1	2	3	1	1	1
5378	37	6	1	2	1	1	2	1	2	0
5379	27	4	1	5	1	2	2	1	2	0
5380	29	4	1	3	1	2	3	1	1	1
5381	20	3	1	8	1	2	2	1	2	0
5382	40	7	1	1	1	2	2	1	1	1
5383	22	3	2	7	1	2	3	1	2	0
5384	21	3	1	5	1	2	2	1	2	0
5385	26	4	1	3	1	2	2	1	1	0
5386	16	2	1	5	1	2	3	1	2	0
5387	34	5	2	3	1	2	2	1	2	0
5388	20	3	2	5	1	2	2	1	2	0
5389	40	7	2	3	1	2	2	1	1	2

5390	21	3	2	8	1	2	2	1	1	0
5391	31	5	2	3	1	2	2	4	1	1
5392	55	10	1	3	1	1	2	1	2	0
5393	14	1	1	7	1	2	3	1	2	0
5394	28	4	1	2	1	2	3	1	2	0
5395	30	5	1	3	1	1	3	1	2	0
5396	13	1	1	5	1	2	1	1	1	0
5397	33	5	2	3	1	2	3	1	2	0
5398	17	2	1	7	1	2	2	1	1	0
5399	15	2	1	7	1	2	2	1	1	0
5400	23	3	1	8	1	2	3	1	1	0
5401	32	5	2	2	1	4	2	1	1	0
5402	56	10	1	1	1	1	3	1	2	0
5403	17	2	1	7	1	2	3	1	1	0
5404	62	11	2	5	1	2	1	1	2	0
5405	60	11	1	2	1	1	3	1	2	0
5406	15	2	1	7	1	2	2	1	1	0
5407	65	12	1	2	1	1	2	1	1	0
5408	27	4	1	3	1	2	3	1	2	0
5409	47	8	2	2	1	1	2	1	1	0
5410	36	6	2	3	1	2	3	1	1	0
5411	22	3	1	3	1	2	2	1	2	0
5412	72	13	1	5	1	1	2	1	1	0
5413	34	5	1	3	1	2	3	1	2	0
5414	14	1	2	7	1	2	3	1	1	0
5415	29	4	1	3	1	2	2	1	2	0
5416	21	3	1	8	1	2	3	1	1	0
5417	19	2	1	7	1	2	3	1	2	0
5418	65	12	1	2	1	1	2	1	2	0
5419	12	1	1	7	1	2	2	1	2	0
5420	32	5	2	2	1	1	2	1	2	0

5421	22	3	1	5	1	2	2	1	1	0
5422	27	4	1	5	1	2	2	1	2	0
5423	63	11	1	3	1	1	2	5	1	0
5424	30	5	2	3	1	1	2	5	1	0
5425	60	11	1	2	1	1	3	5	1	0
5426	18	2	1	5	1	2	2	5	1	0
5427	56	10	1	1	1	1	2	5	1	0
5428	13	1	2	7	1	2	3	5	1	0
5429	10	1	1	7	1	2	2	5	1	0
5430	75	14	1	2	1	1	1	5	2	0
5431	56	10	1	2	1	2	2	1	1	1
5432	14	1	2	7	1	2	3	5	1	0
5433	70	13	1	2	1	1	2	5	1	0
5434	13	1	2	7	1	2	3	5	2	0
5435	12	1	2	7	1	2	2	5	2	0
5436	50	9	2	6	1	1	4	5	2	0
5437	33	5	1	5	1	2	4	5	2	0
5438	20	3	1	7	1	2	3	5	2	0
5439	18	2	1	7	1	2	3	5	2	0
5440	16	2	2	7	1	2	3	5	2	0
5441	80	15	2	1	1	2	1	5	2	0
5442	14	1	2	7	1	2	3	5	2	0
5443	45	8	1	2	1	1	2	5	2	0
5444	50	9	1	3	1	1	2	5	2	0
5445	35	6	1	2	1	1	3	5	2	0
5446	45	8	1	2	1	1	2	5	2	0
5447	42	7	1	2	1	1	3	5	2	0
5448	45	8	1	3	1	1	3	5	2	0
5449	25	4	1	3	1	1	3	5	2	0
5450	70	13	1	2	1	1	3	5	2	0
5451	32	5	1	4	1	2	3	5	2	0

5452	50	9	2	2	1	1	3	5	2	0
5453	50	9	1	2	1	1	2	5	2	0
5454	37	6	2	2	1	2	4	5	2	0
5455	30	5	2	2	1	1	3	5	2	0
5456	40	7	2	2	1	1	3	5	2	0
5457	15	2	1	7	1	2	2	5	1	0
5458	35	6	1	2	1	1	3	5	2	0
5459	45	8	1	1	1	1	2	5	2	0
5460	38	6	2	2	1	1	2	5	2	0
5461	35	6	2	1	1	1	2	5	2	0
5462	38	6	1	3	1	1	3	5	2	0
5463	26	4	1	3	1	1	3	5	2	0
5464	35	6	1	2	1	1	2	5	2	0
5465	25	4	2	1	1	4	2	5	2	0
5466	35	6	2	3	1	1	2	5	2	0
5467	36	6	2	1	1	1	3	5	2	0
5468	42	7	2	1	1	1	2	5	2	0
5469	27	4	2	2	1	1	3	5	2	0
5470	16	2	1	7	1	2	3	5	2	0
5471	18	2	1	7	1	2	3	5	2	0
5472	12	1	1	7	1	2	3	5	2	0
5473	45	8	2	1	1	1	2	5	2	0
5474	27	4	1	3	1	1	3	5	2	0
5475	20	3	2	7	1	2	3	5	2	0
5476	22	3	2	4	1	1	3	5	2	0
5477	23	3	2	2	1	1	3	5	2	0
5478	28	4	2	5	1	1	3	5	2	0
5479	10	1	1	7	1	2	2	5	2	0
5480	16	2	2	7	1	2	3	5	2	0
5481	50	9	2	1	1	1	3	5	2	0
5482	15	2	2	7	1	2	3	5	2	0

5483	36	6	1	3	1	1	4	5	2	0
5484	23	3	1	3	1	2	3	5	2	0
5485	27	4	2	8	1	1	3	5	2	0
5486	19	2	1	7	1	2	2	5	2	0
5487	55	10	2	2	1	1	2	5	2	0
5488	20	3	2	7	1	2	4	5	2	0
5489	28	4	1	7	1	2	2	5	2	0
5490	26	4	1	3	1	2	2	5	2	0
5491	53	9	2	1	1	1	2	5	2	0
5492	30	5	2	2	1	1	3	5	2	0
5493	32	5	2	1	1	1	3	5	2	0
5494	44	7	1	2	1	3	3	5	2	0
5495	37	6	2	5	1	2	4	5	2	0
5496	56	10	2	1	1	1	3	5	2	0
5497	16	2	1	7	1	2	3	5	2	0
5498	21	3	2	5	1	1	3	5	2	0
5499	34	5	1	3	1	1	3	5	2	0
5500	32	5	1	3	1	2	2	5	2	0
5501	24	3	2	7	1	2	4	5	2	0
5502	58	10	2	5	1	1	3	5	2	0
5503	30	5	1	3	1	2	3	5	2	0
5504	25	4	2	7	1	2	3	5	2	0
5505	31	5	2	4	1	1	4	5	2	0
5506	34	5	2	4	1	1	4	5	2	0
5507	11	1	1	5	1	2	1	5	2	0
5508	14	1	1	7	1	2	3	5	2	0
5509	18	2	1	7	1	2	2	5	2	0
5510	38	6	2	2	1	1	2	5	2	0
5511	16	2	1	7	1	2	3	5	2	0
5512	56	10	1	2	1	1	2	5	2	0
5513	33	5	1	2	1	2	4	5	2	0

5514	10	1	2	7	1	2	2	5	1	0
5515	19	2	1	7	1	2	3	3	2	0
5516	19	2	1	7	1	2	3	3	2	0
5517	38	6	2	4	1	2	3	3	2	0
5518	30	5	2	2	1	2	3	3	2	0
5519	29	4	1	7	1	2	3	3	2	0
5520	18	2	1	7	1	2	3	3	2	0
5521	39	6	2	2	1	1	4	3	2	0
5522	28	4	1	5	1	2	4	3	2	0
5523	53	9	2	4	1	1	3	3	2	0
5524	30	5	2	5	1	2	2	3	2	0
5525	16	2	2	7	1	2	3	3	1	0
5526	24	3	2	7	1	2	3	3	1	0
5527	33	5	2	3	1	2	3	3	1	0
5528	53	9	1	3	1	1	3	3	2	0
5529	43	7	2	2	1	2	2	3	2	0
5530	48	8	1	5	1	2	3	3	2	0
5531	36	6	1	3	1	2	2	3	1	0
5532	18	2	1	8	1	2	2	3	1	0
5533	37	6	1	3	1	2	2	3	2	0
5534	27	4	2	3	1	2	1	3	2	0
5535	28	4	2	5	1	2	2	3	2	0
5536	11	1	2	7	1	2	2	3	1	0
5537	17	2	1	7	1	2	3	3	2	0
5538	32	5	2	3	1	2	1	3	1	0
5539	18	2	1	7	1	2	3	3	2	0
5540	25	4	2	8	1	2	2	3	2	0
5541	19	2	2	7	1	2	3	3	2	0
5542	10	1	2	7	1	2	2	3	2	0
5543	17	2	1	7	1	2	3	3	1	0
5544	13	1	1	7	1	2	2	3	2	0

5545	34	5	2	3	1	2	3	3	2	0
5546	30	5	1	3	1	2	3	3	2	0
5547	14	1	1	5	1	2	1	3	2	0
5548	20	3	1	3	1	2	2	3	2	0
5549	59	10	1	2	1	1	2	4	1	0
5550	13	1	2	7	1	2	2	4	2	0
5551	42	7	1	3	1	3	3	4	2	0
5552	45	8	2	2	1	1	2	4	2	0
5553	31	5	1	3	1	2	2	4	2	0
5554	33	5	2	8	1	1	4	3	2	0
5555	39	6	1	4	1	1	4	3	2	0
5556	53	9	1	2	1	1	2	3	2	0
5557	48	8	1	2	1	1	3	6	2	0
5558	28	4	2	3	1	2	3	4	2	0
5559	59	10	1	2	1	1	2	6	2	0
5560	69	12	2	2	1	1	3	6	2	0
5561	37	6	1	3	1	1	3	3	2	0
5562	27	4	2	2	1	2	3	3	2	0
5563	21	3	2	8	1	2	3	3	2	0
5564	50	9	1	2	1	1	3	3	2	0
5565	61	11	1	2	1	1	3	3	2	0
5566	16	2	2	7	1	2	3	3	2	0
5567	43	7	2	2	1	1	3	3	2	0
5568	36	6	1	2	1	1	3	3	2	0
5569	11	1	2	7	1	2	2	3	2	0
5570	32	5	1	3	1	1	3	3	2	0
5571	28	4	2	3	1	1	3	3	2	0
5572	16	2	1	7	1	2	3	3	2	0
5573	17	2	1	7	1	2	3	3	2	0
5574	18	2	1	7	1	2	3	3	2	0
5575	25	4	2	8	1	2	3	3	2	0

5576	19	2	2	7	1	2	3	3	2	0
5577	10	1	2	7	1	2	2	3	2	0
5578	17	2	1	7	1	2	3	3	2	0
5579	18	2	2	7	1	2	3	3	2	0
5580	15	2	1	7	1	2	3	3	2	0
5581	18	2	2	7	1	2	3	3	2	0
5582	13	1	1	7	1	2	3	3	2	0
5583	34	5	2	3	1	1	4	3	2	0
5584	30	5	1	3	1	1	3	3	2	0
5585	43	7	1	2	1	1	4	3	2	0
5586	20	2	1	3	1	2	3	3	2	0
5587	40	7	1	3	1	1	3	3	2	0
5588	14	1	1	7	1	2	3	3	2	0
5589	17	2	2	7	1	2	3	4	2	0
5590	35	6	1	3	1	2	4	6	2	0
5591	65	12	2	6	1	4	4	6	2	0
5592	28	4	1	4	1	2	4	6	2	0
5593	27	4	1	2	1	1	2	6	2	0
5594	40	7	1	4	1	1	4	6	2	0
5595	11	1	1	7	1	2	2	6	2	0
5596	34	5	2	4	1	1	3	6	2	0
5597	18	2	2	7	1	2	3	6	2	0
5598	30	5	2	4	1	1	3	6	2	0
5599	32	5	2	4	1	1	3	6	2	0
5600	42	7	1	4	1	2	4	6	2	0
5601	31	5	2	2	1	1	3	3	2	0
5602	38	6	1	4	1	2	3	3	2	0
5603	19	2	2	7	1	2	4	3	2	0
5604	42	7	1	2	1	1	3	6	2	0
5605	46	8	1	2	1	1	3	6	2	0
5606	40	7	2	2	1	1	3	3	2	0

5607	19	2	1	7	1	2	3	3	2	0
5608	20	3	1	7	1	2	4	3	2	0
5609	30	5	2	8	1	1	3	3	2	0
5610	12	1	1	7	1	2	2	3	2	0
5611	34	5	2	4	1	1	4	3	2	0
5612	42	7	2	4	1	1	3	6	2	0
5613	40	7	2	2	1	1	3	6	2	0
5614	30	5	2	4	1	1	3	3	2	0
5615	45	8	2	2	1	1	3	6	2	0
5616	60	11	2	2	1	1	2	6	2	0
5617	30	5	2	4	1	2	4	3	2	0
5618	41	7	2	4	1	2	4	3	2	0
5619	29	4	2	2	1	1	3	3	2	0
5620	45	8	1	2	1	1	3	3	2	0
5621	48	8	1	3	1	1	3	6	2	0
5622	27	4	2	3	1	1	3	3	2	0
5623	19	2	1	7	1	2	3	3	2	0
5624	35	6	1	2	1	1	3	3	2	0
5625	12	1	1	7	1	2	2	3	2	0
5626	37	6	1	2	1	1	2	3	2	0
5627	13	1	1	7	1	2	3	3	2	0
5628	10	1	1	7	1	2	2	3	2	0
5629	32	5	2	8	1	2	4	6	2	0
5630	15	2	2	7	1	2	3	3	2	0
5631	42	7	1	3	1	2	3	1	1	1
5632	38	6	2	4	1	2	4	1	2	0
5633	12	1	1	7	1	2	2	1	1	1
5634	16	2	1	5	1	2	2	1	2	0
5635	11	1	1	7	1	2	2	1	2	0
5636	17	2	2	5	1	2	2	4	1	1
5637	67	12	1	1	1	1	3	3	2	0

5638	61	11	2	2	1	4	1	5	2	0
5639	56	10	1	3	1	1	3	4	2	0
5640	68	12	1	2	1	1	2	4	2	0
5641	70	13	1	1	1	1	3	4	2	0
5642	64	11	2	5	1	4	2	4	2	0
5643	65	12	1	1	1	1	2	4	2	0
5644	16	2	1	3	1	2	3	4	2	0
5645	18	2	1	8	1	2	2	4	2	0
5646	52	9	1	2	1	1	3	4	2	0
5647	14	1	1	7	1	2	3	4	2	0
5648	12	1	1	7	1	2	3	4	2	0
5649	17	2	1	7	1	2	3	4	2	0
5650	15	2	2	7	1	2	3	4	2	0
5651	10	1	2	7	1	2	3	4	2	0
5652	46	8	2	2	1	1	3	4	2	0
5653	42	7	1	4	1	1	4	6	2	0
5654	40	7	2	2	1	1	3	6	2	0
5655	15	2	1	7	1	2	3	6	2	0
5656	12	1	2	7	1	2	2	6	2	0
5657	10	1	1	7	1	2	2	4	2	0
5658	46	8	1	4	1	1	4	6	2	0
5659	40	7	2	4	1	1	4	6	2	0
5660	10	1	2	7	1	2	2	6	2	0
5661	47	8	1	4	1	1	4	4	2	0
5662	35	6	2	4	1	1	4	4	2	0
5663	19	2	2	5	1	2	3	6	2	0
5664	84	15	1	5	1	1	3	4	2	0
5665	63	11	2	2	1	1	1	4	2	0
5666	50	9	1	4	1	1	4	4	2	0
5667	42	7	2	4	1	1	4	4	2	0
5668	14	1	2	7	1	2	3	4	2	0

5669	12	1	1	7	1	2	2	4	2	0
5670	10	1	1	7	1	2	2	4	2	0
5671	45	8	2	4	1	2	3	4	2	0
5672	36	6	1	2	1	1	3	6	2	0
5673	30	5	2	2	1	1	3	6	2	0
5674	12	1	2	7	1	2	2	6	2	0
5675	11	1	1	7	1	2	2	4	2	0
5676	38	6	1	1	1	1	3	4	2	0
5677	30	5	2	2	1	1	3	4	2	0
5678	14	1	1	7	1	2	3	4	2	0
5679	12	1	1	7	1	2	3	4	2	0
5680	10	1	2	7	1	2	2	4	2	0
5681	42	7	1	1	1	1	3	4	2	0
5682	40	7	2	2	1	1	3	3	2	0
5683	62	11	1	2	1	4	3	3	2	0
5684	24	3	2	7	1	5	4	3	2	0
5685	20	3	1	7	1	2	4	3	2	0
5686	56	10	1	2	1	1	4	3	2	0
5687	44	7	2	2	1	1	3	3	2	0
5688	20	3	2	7	1	2	4	3	2	0
5689	18	2	2	7	1	2	3	3	2	0
5690	16	2	1	7	1	2	3	3	2	0
5691	14	1	2	7	1	2	3	3	2	0
5692	12	1	2	7	1	2	2	3	2	0
5693	11	1	1	7	1	2	2	3	2	0
5694	92	15	1	5	3	1	1	3	2	0
5695	74	13	2	2	1	1	2	3	2	0
5696	48	8	1	2	1	1	3	3	2	0
5697	36	6	2	2	1	1	3	3	2	0
5698	20	3	1	7	1	2	3	3	2	0
5699	19	2	1	7	1	2	3	3	2	0

5700	16	2	1	7	1	2	3	3	2	0
5701	44	7	1	2	1	1	3	3	2	0
5702	33	5	2	2	1	1	3	3	2	0
5703	17	2	2	7	1	2	3	3	2	0
5704	15	2	2	7	1	2	3	3	2	0
5705	12	1	2	7	1	2	2	3	2	0
5706	10	1	1	7	1	2	2	3	2	0
5707	38	6	2	2	1	2	3	3	2	0
5708	40	7	1	2	1	1	3	3	2	0
5709	30	5	2	2	1	1	3	3	2	0
5710	10	1	1	7	1	2	2	3	2	0
5711	36	6	1	2	1	2	3	3	2	0
5712	86	15	1	5	1	1	2	4	2	0
5713	70	13	2	2	1	1	3	4	2	0
5714	46	8	1	2	1	1	4	4	2	0
5715	30	5	2	2	1	1	3	4	2	0
5716	44	7	1	1	1	1	3	4	2	0
5717	32	5	2	2	1	1	3	4	2	0
5718	20	3	2	7	1	2	4	4	2	0
5719	18	2	1	7	1	2	3	4	2	0
5720	15	2	1	7	1	2	3	4	2	0
5721	16	2	2	7	1	2	3	4	2	0
5722	14	1	2	7	1	2	2	4	2	0
5723	12	1	2	7	1	2	2	4	2	0
5724	11	1	1	7	1	2	2	4	2	0
5725	11	1	2	7	1	2	2	4	2	0
5726	13	1	1	7	1	2	3	4	2	0
5727	66	12	1	6	1	1	4	4	2	0
5728	52	9	2	4	1	1	4	4	2	0
5729	19	2	1	7	1	2	4	4	2	0
5730	17	2	2	7	1	2	4	4	2	0

5731	15	2	1	7	1	2	3	4	2	0
5732	11	1	1	7	1	2	2	4	2	0
5733	83	15	1	1	3	1	1	4	2	0
5734	66	12	2	2	1	1	2	4	2	0
5735	40	7	1	2	1	1	3	4	2	0
5736	32	5	2	2	1	1	3	4	2	0
5737	12	1	1	7	1	2	2	4	2	0
5738	10	1	1	7	1	2	2	4	2	0
5739	80	15	1	6	1	4	4	4	2	0
5740	46	8	1	4	1	1	4	4	2	0
5741	38	6	2	4	1	1	4	4	2	0
5742	14	1	1	7	1	2	3	4	2	0
5743	12	1	1	7	1	2	3	4	2	0
5744	10	1	1	7	1	2	2	4	2	0
5745	84	15	2	5	1	4	3	4	2	0
5746	37	6	1	2	1	1	3	4	2	0
5747	35	6	2	2	1	1	3	4	2	0
5748	78	14	1	1	3	1	1	4	2	0
5749	70	13	2	2	1	1	2	4	2	0
5750	50	9	2	2	1	1	3	4	2	0
5751	56	10	1	2	1	1	3	4	2	0
5752	43	7	1	3	3	1	2	4	2	0
5753	32	5	2	2	1	1	2	4	2	0
5754	43	7	1	3	1	1	2	4	2	0
5755	40	7	2	2	1	1	3	4	2	0
5756	40	7	1	2	1	1	3	4	2	0
5757	28	4	2	2	1	1	3	4	2	0
5758	20	3	1	7	1	2	4	4	2	0
5759	17	2	1	7	1	2	3	4	2	0
5760	74	13	1	6	1	1	4	4	2	0
5761	68	12	2	6	1	1	4	4	2	0

5762	40	7	1	4	1	1	4	4	2	0
5763	32	5	2	2	1	1	4	4	2	0
5764	36	6	2	4	1	2	4	4	2	0
5765	34	5	1	4	1	1	4	4	2	0
5766	27	4	2	4	1	1	4	4	2	0
5767	25	4	1	7	1	2	4	4	2	0
5768	20	3	1	7	1	2	4	4	2	0
5769	16	2	1	7	1	2	3	4	2	0
5770	58	10	1	2	1	1	3	1	2	0
5771	34	5	2	4	1	1	4	1	2	0
5772	14	1	1	7	1	2	3	4	2	0
5773	12	1	1	7	1	2	3	1	2	0
5774	10	1	2	7	1	2	2	1	2	0
5775	66	12	2	2	1	4	3	1	2	0
5776	40	7	1	2	1	1	3	1	2	0
5777	30	5	2	2	1	1	3	1	2	0
5778	36	6	1	3	1	1	3	1	2	0
5779	29	4	2	2	1	1	3	1	2	0
5780	29	4	1	2	1	1	3	1	2	0
5781	26	4	2	2	1	1	3	1	2	0
5782	16	2	1	7	1	2	3	1	2	0
5783	14	1	1	7	1	2	3	1	2	0
5784	12	1	2	7	1	2	2	1	2	0
5785	10	1	2	7	1	2	2	1	2	0
5786	15	2	2	7	1	2	3	1	2	0
5787	13	1	2	7	1	2	3	1	2	0
5788	11	1	2	7	1	2	2	1	2	0
5789	10	1	2	7	1	2	2	1	2	0
5790	12	1	1	7	1	2	2	1	2	0
5791	51	9	1	3	1	1	3	6	2	0
5792	44	7	2	2	1	1	3	6	2	0

5793	18	2	1	7	1	2	3	6	2	0
5794	15	2	1	7	1	2	3	6	2	0
5795	13	1	1	7	1	2	3	6	2	0
5796	10	1	1	7	1	2	2	6	2	0
5797	58	10	1	1	1	1	3	1	2	0
5798	50	9	2	1	1	1	3	1	2	0
5799	27	4	1	2	1	2	3	1	2	0
5800	21	3	1	3	1	2	3	1	2	0
5801	22	3	2	7	1	2	4	1	2	0
5802	16	2	1	7	1	2	3	1	2	0
5803	12	1	1	7	1	2	2	1	2	0
5804	74	13	1	6	1	1	3	1	2	0
5805	60	11	2	4	1	1	3	1	2	0
5806	35	6	1	2	1	1	3	1	2	0
5807	28	4	2	2	1	1	3	1	2	0
5808	33	5	1	3	1	1	3	1	2	0
5809	30	5	2	2	1	1	3	1	2	0
5810	31	5	1	2	1	2	3	1	2	0
5811	29	4	2	7	1	2	4	1	2	0
5812	10	1	1	7	1	2	2	1	2	0
5813	69	12	2	2	1	4	3	1	2	0
5814	40	7	1	2	1	1	3	1	2	0
5815	29	4	2	2	1	1	3	1	2	0
5816	35	6	1	2	1	2	3	1	2	0
5817	11	1	1	7	1	2	2	1	2	0
5818	76	14	1	6	1	1	4	1	2	0
5819	72	13	2	6	1	1	3	1	2	0
5820	34	6	1	4	1	1	4	1	2	0
5821	29	4	2	4	1	1	4	1	2	0
5822	51	9	1	3	1	1	2	6	2	0
5823	46	8	2	2	1	1	2	6	2	0

5824	20	3	1	3	1	2	3	6	2	0
5825	13	1	2	7	1	2	2	6	2	0
5826	16	2	1	7	1	2	3	6	2	0
5827	10	1	1	7	1	2	2	6	2	0
5828	69	12	1	1	1	4	2	6	2	0
5829	30	5	1	2	1	2	3	6	2	0
5830	26	4	1	3	1	2	3	6	2	0
5831	23	3	1	2	1	2	3	6	2	0
5832	18	2	2	7	1	2	3	6	2	0
5833	75	14	2	2	1	4	3	6	2	0
5834	65	12	1	2	1	1	2	1	2	0
5835	33	5	1	2	1	1	3	1	2	0
5836	36	6	2	4	1	1	4	1	2	0
5837	40	7	1	2	1	1	3	1	2	0
5838	36	6	2	2	1	1	3	1	2	0
5839	17	2	2	7	1	2	3	1	2	0
5840	37	6	1	2	1	1	3	1	2	0
5841	29	4	2	2	1	1	3	1	2	0
5842	14	1	2	7	1	2	3	1	2	0
5843	82	15	1	1	3	1	1	5	2	0
5844	67	12	2	2	3	1	3	5	2	0
5845	34	5	2	1	1	2	2	5	2	0
5846	42	7	1	2	1	1	3	5	2	0
5847	32	5	2	2	1	1	3	5	2	0
5848	36	6	2	4	1	2	4	5	2	0
5849	32	5	2	2	1	2	3	5	2	0
5850	30	5	1	2	1	1	3	5	2	0
5851	23	3	2	2	1	1	3	5	2	0
5852	16	2	1	7	1	2	3	5	2	0
5853	15	2	1	7	1	2	3	5	2	0
5854	56	10	2	2	1	4	2	3	2	0

5855	33	5	1	2	3	1	1	3	2	0
5856	24	3	2	2	1	1	3	3	2	0
5857	68	12	1	3	1	1	3	3	2	0
5858	60	11	2	2	1	1	3	3	2	0
5859	42	7	1	2	1	1	3	3	2	0
5860	38	6	2	2	1	1	3	3	2	0
5861	39	6	1	2	1	1	3	3	2	0
5862	18	2	1	7	1	2	4	3	2	0
5863	15	2	1	7	1	2	3	3	2	0
5864	11	1	1	7	1	2	2	3	2	0
5865	49	8	1	2	1	1	3	3	2	0
5866	40	7	2	2	1	1	2	3	2	0
5867	12	1	1	7	1	2	2	3	2	0
5868	71	13	2	2	1	1	2	3	2	0
5869	78	14	1	2	1	1	1	3	2	0
5870	18	2	2	7	1	2	3	3	2	0
5871	60	11	2	2	1	1	2	3	2	0
5872	40	7	1	3	1	1	3	3	2	0
5873	32	5	2	2	1	1	3	3	2	0
5874	36	6	1	2	1	1	3	3	2	0
5875	29	4	1	3	1	2	3	3	2	0
5876	24	3	1	7	1	2	4	3	2	0
5877	18	2	2	7	1	2	3	3	2	0
5878	80	15	1	2	1	1	3	3	2	0
5879	40	7	1	2	1	1	3	6	2	0
5880	28	4	2	2	1	1	3	6	2	0
5881	10	1	2	7	1	2	2	6	2	0
5882	25	4	1	8	1	2	3	6	2	0
5883	22	3	1	8	1	2	3	6	2	0
5884	45	8	1	3	1	1	3	3	2	0
5885	36	6	2	2	1	1	3	3	2	0

5886	11	1	1	7	1	2	2	3	2	0
5887	53	9	1	2	1	1	3	3	2	0
5888	40	7	2	4	1	1	3	3	2	0
5889	16	2	1	7	1	2	3	3	2	0
5890	14	1	1	7	1	2	3	3	2	0
5891	11	1	2	7	1	2	2	3	2	0
5892	60	11	2	4	1	4	4	3	2	0
5893	32	5	1	4	1	2	4	3	2	0
5894	20	3	2	7	1	2	4	3	2	0
5895	14	1	2	7	1	2	3	3	2	0
5896	68	12	1	3	3	1	2	3	2	0
5897	50	9	2	1	3	1	2	3	2	0
5898	36	6	1	2	1	1	3	3	2	0
5899	33	5	1	2	1	1	3	3	2	0
5900	28	4	2	8	1	2	4	3	2	0
5901	22	3	2	7	1	2	4	3	2	0
5902	62	11	1	2	1	1	2	3	2	0
5903	48	8	2	2	1	1	3	3	2	0
5904	25	4	1	2	1	2	3	3	2	0
5905	23	3	2	7	1	2	4	3	2	0
5906	20	3	1	7	1	2	3	3	2	0
5907	45	8	1	2	1	1	3	3	2	0
5908	43	7	2	2	1	1	3	3	2	0
5909	17	2	2	7	1	2	3	3	2	0
5910	14	1	2	7	1	2	3	3	2	0
5911	12	1	1	7	1	2	2	3	2	0
5912	60	11	2	1	1	4	2	3	2	0
5913	44	7	1	2	1	1	3	3	2	0
5914	30	5	2	2	1	1	3	3	2	0
5915	38	6	1	1	1	1	3	3	2	0
5916	27	4	2	2	1	1	3	3	2	0

5917	35	6	1	2	1	1	3	3	2	0
5918	34	5	2	4	1	1	3	3	2	0
5919	30	5	2	2	1	2	3	3	2	0
5920	26	4	1	3	1	2	2	1	2	0
5921	20	3	2	7	1	2	4	1	2	0
5922	17	2	1	7	1	2	3	1	2	0
5923	13	1	1	7	1	2	2	1	2	0
5924	13	1	2	7	1	2	2	1	2	0
5925	12	1	2	7	1	2	2	1	2	0
5926	11	1	2	7	1	2	2	1	2	0
5927	48	8	2	2	1	3	3	1	2	0
5928	15	2	2	7	1	2	3	1	2	0
5929	13	1	2	7	1	2	3	1	2	0
5930	40	7	2	4	1	1	4	1	2	0
5931	55	10	1	3	1	1	2	1	2	0
5932	61	11	1	1	3	1	3	1	2	0
5933	73	13	2	2	1	4	2	1	2	0
5934	51	9	2	2	1	1	3	1	2	0
5935	30	5	2	2	1	2	3	1	2	0
5936	27	4	2	7	1	2	4	1	2	0
5937	25	4	1	7	1	2	4	1	2	0
5938	20	3	1	7	1	2	4	1	2	0
5939	24	3	1	3	1	2	3	1	2	0
5940	12	1	1	7	1	2	2	5	2	0
5941	17	2	1	7	1	2	3	5	2	0
5942	52	9	2	2	1	1	3	5	2	0
5943	23	3	1	3	1	2	3	5	2	0
5944	15	2	2	7	1	2	3	5	2	0
5945	13	1	1	7	1	2	3	5	2	0
5946	17	2	1	7	1	2	3	5	2	0
5947	21	3	1	7	1	2	3	5	2	0

5948	13	1	2	7	1	2	3	5	2	0
5949	73	13	1	1	1	1	2	5	2	0
5950	54	9	2	2	1	1	3	5	2	0
5951	32	5	2	2	1	1	3	5	2	0
5952	43	7	2	2	1	1	2	5	2	0
5953	90	15	1	5	3	1	2	5	2	0
5954	78	14	2	1	1	1	3	5	2	0
5955	45	8	1	2	1	1	3	5	2	0
5956	56	10	1	2	1	1	3	5	2	0
5957	59	10	1	1	1	1	3	5	2	0
5958	50	9	2	1	1	1	3	5	2	0
5959	42	7	1	2	1	1	3	5	2	0
5960	38	6	1	3	1	1	2	5	2	0
5961	38	6	2	2	1	1	3	5	2	0
5962	28	4	2	2	1	1	3	5	2	0
5963	22	3	1	7	1	2	4	5	2	0
5964	32	5	2	4	1	2	4	5	2	0
5965	16	2	2	7	1	2	3	5	2	0
5966	13	1	1	7	1	2	2	5	2	0
5967	25	4	1	7	1	2	3	5	2	0
5968	67	12	1	1	1	4	1	5	2	0
5969	18	2	1	7	1	2	3	6	2	0
5970	20	3	2	7	1	2	3	5	2	0
5971	84	15	1	5	1	1	1	5	2	0
5972	56	10	2	2	1	1	2	5	2	0
5973	30	5	1	3	1	1	3	5	2	0
5974	25	4	2	2	1	1	3	5	2	0
5975	28	4	1	2	1	2	3	5	2	0
5976	26	4	2	7	1	2	3	5	2	0
5977	24	3	1	3	1	2	3	5	2	0
5978	62	11	1	1	3	1	2	5	2	0

5979	50	9	2	1	1	1	3	5	2	0
5980	30	5	2	2	1	2	3	5	2	0
5981	28	4	2	2	1	2	3	5	2	0
5982	24	3	1	3	1	2	3	5	2	0
5983	57	10	2	2	1	4	2	5	2	0
5984	33	5	1	2	1	2	3	5	2	0
5985	30	5	1	2	1	1	3	5	2	0
5986	23	3	2	2	1	1	3	5	2	0
5987	27	4	2	7	1	2	3	5	2	0
5988	83	15	1	6	1	4	4	5	2	0
5989	22	3	2	8	1	2	3	5	2	0
5990	51	9	1	2	1	1	2	3	2	0
5991	37	6	2	2	1	1	3	3	2	0
5992	16	2	1	7	1	2	3	3	2	0
5993	14	1	1	7	1	2	3	3	2	0
5994	12	1	2	7	1	2	2	3	2	0
5995	10	1	1	7	1	2	2	3	2	0
5996	53	9	1	3	1	1	3	3	2	0
5997	43	7	2	2	1	1	3	3	2	0
5998	17	2	2	7	1	2	3	3	2	0
5999	20	3	1	7	1	2	3	3	2	0
6000	15	2	1	7	1	2	3	3	2	0
6001	13	1	1	7	1	2	3	3	2	0
6002	59	10	1	1	1	1	2	3	2	0
6003	41	7	2	2	1	1	2	3	2	0
6004	17	2	1	7	1	2	3	3	2	0
6005	15	2	2	7	1	2	3	3	2	0
6006	13	1	1	7	1	2	3	3	2	0
6007	11	1	2	7	1	2	2	3	2	0
6008	63	11	2	2	1	4	2	3	2	0
6009	36	6	1	2	1	1	2	3	2	0

6010	28	4	2	2	1	1	3	3	2	0
6011	28	4	1	2	1	2	3	3	2	0
6012	32	5	1	2	1	1	3	3	2	0
6013	30	5	1	2	1	1	3	3	2	0
6014	13	1	1	7	1	2	3	3	2	0
6015	20	3	1	7	1	2	3	3	2	0
6016	11	1	1	7	1	2	3	3	2	0
6017	10	1	2	7	1	2	3	3	2	0
6018	68	12	2	2	1	4	2	3	2	0
6019	42	7	1	2	1	1	3	3	2	0
6020	38	6	1	2	1	1	3	3	2	0
6021	35	6	1	2	1	1	3	3	2	0
6022	32	5	1	2	1	1	3	3	2	0
6023	28	4	2	2	1	1	3	3	2	0
6024	23	3	2	2	1	1	3	3	2	0
6025	22	3	2	7	1	1	3	3	2	0
6026	17	2	2	7	1	2	3	3	2	0
6027	16	2	2	7	1	2	3	3	2	0
6028	14	1	1	7	1	2	3	3	2	0
6029	12	1	1	7	1	2	3	3	2	0
6030	10	1	1	7	1	2	2	3	2	0
6031	11	1	1	7	1	2	2	3	2	0
6032	41	7	1	1	1	1	2	6	2	0
6033	28	4	2	1	1	1	2	6	2	0
6034	59	10	1	2	1	1	3	3	2	0
6035	30	5	2	2	1	1	3	3	2	0
6036	12	1	2	7	1	2	2	3	2	0
6037	44	7	1	3	1	1	3	6	2	0
6038	26	4	2	2	1	1	3	6	2	0
6039	32	5	2	2	1	1	3	3	2	0
6040	12	1	1	7	1	2	3	3	2	0

6041	10	1	2	7	1	2	2	3	2	0
6042	38	6	1	2	1	1	2	3	2	0
6043	58	10	1	2	1	1	3	3	2	0
6044	42	7	2	2	1	1	3	5	2	0
6045	19	2	1	7	1	2	4	5	2	0
6046	16	2	1	7	1	2	3	5	2	0
6047	14	1	1	7	1	2	3	5	2	0
6048	12	1	1	7	1	2	2	5	2	0
6049	70	13	1	1	1	1	2	5	2	0
6050	57	10	2	2	1	1	3	5	2	0
6051	23	3	1	7	1	2	4	5	2	0
6052	31	5	1	2	1	1	3	5	2	0
6053	27	4	1	3	1	2	3	5	2	0
6054	18	2	2	7	1	2	3	5	2	0
6055	22	3	2	2	1	1	3	5	2	0
6056	55	10	1	2	1	1	3	5	2	0
6057	48	8	2	2	1	1	3	5	2	0
6058	23	3	1	3	1	1	2	5	2	0
6059	20	3	1	2	1	2	3	5	2	0
6060	18	2	2	7	1	2	3	5	2	0
6061	16	2	2	7	1	2	3	5	2	0
6062	14	1	2	7	1	2	3	5	2	0
6063	12	1	2	7	1	2	2	5	2	0
6064	21	3	2	2	1	1	2	5	2	0
6065	68	12	1	1	1	4	2	5	2	0
6066	30	5	1	3	1	1	2	5	2	0
6067	38	6	1	1	1	1	2	5	2	0
6068	35	6	1	2	1	1	2	5	2	0
6069	26	4	2	2	1	2	3	5	2	0
6070	23	3	1	3	1	1	2	5	2	0
6071	22	3	2	2	1	1	3	5	2	0

6072	17	2	2	7	1	2	3	5	2	0
6073	18	2	1	7	1	2	3	5	2	0
6074	13	1	1	7	1	2	3	5	2	0
6075	12	1	1	7	1	2	3	5	2	0
6076	11	1	1	7	1	2	3	5	2	0
6077	53	9	1	3	1	1	2	5	2	0
6078	51	9	2	1	1	1	2	5	2	0
6079	29	4	1	2	1	1	2	5	2	0
6080	27	4	2	2	1	1	2	5	2	0
6081	25	4	1	3	1	1	2	5	2	0
6082	23	3	1	2	1	1	3	5	2	0
6083	20	3	2	2	1	1	3	5	2	0
6084	18	2	2	8	1	1	2	5	2	0
6085	16	2	2	7	1	2	3	5	2	0
6086	13	1	2	7	1	2	3	5	2	0
6087	11	1	1	7	1	2	3	5	2	0
6088	67	12	2	2	1	4	3	5	2	0
6089	40	7	1	2	1	1	3	5	2	0
6090	32	5	2	2	1	1	3	5	2	0
6091	38	6	1	2	1	1	3	3	2	0
6092	25	4	2	2	1	1	3	3	2	0
6093	35	6	2	2	1	2	3	3	2	0
6094	30	5	1	2	1	2	3	3	2	0
6095	15	2	1	7	1	2	3	3	2	0
6096	13	1	1	7	1	2	3	3	2	0
6097	10	1	1	7	1	2	2	3	2	0
6098	46	8	1	3	1	1	3	6	2	0
6099	38	6	2	2	1	1	3	6	2	0
6100	19	2	2	7	1	2	3	6	2	0
6101	17	2	2	7	1	2	3	6	2	0
6102	14	1	1	7	1	2	3	6	2	0

6103	12	1	1	7	1	2	2	6	2	0
6104	65	12	1	2	1	1	1	3	2	0
6105	53	9	2	2	1	1	2	3	2	0
6106	32	5	2	2	1	2	3	3	2	0
6107	29	4	1	3	1	1	3	3	2	0
6108	27	4	2	3	1	2	3	3	2	0
6109	24	3	1	2	1	2	3	3	2	0
6110	22	3	1	2	1	2	3	3	2	0
6111	20	3	2	7	1	2	3	3	2	0
6112	18	2	1	7	1	2	3	3	2	0
6113	12	1	2	7	1	2	2	3	2	0
6114	22	3	2	2	1	1	3	3	2	0
6115	60	11	1	1	1	1	2	4	2	0
6116	54	9	2	1	1	1	2	4	2	0
6117	33	5	1	2	1	1	2	4	2	0
6118	31	5	1	1	1	2	3	4	2	0
6119	29	4	1	2	1	2	3	4	2	0
6120	24	3	2	2	1	2	3	4	2	0
6121	26	4	1	3	1	2	3	4	2	0
6122	24	3	1	3	1	1	3	4	2	0
6123	22	3	2	2	1	2	3	4	2	0
6124	10	1	1	7	1	2	2	4	2	0
6125	88	15	2	5	1	4	1	4	2	0
6126	60	11	1	2	1	1	3	4	2	0
6127	58	10	1	4	1	1	4	4	2	0
6128	56	10	1	2	1	1	3	4	2	0
6129	54	9	1	2	1	1	3	4	2	0
6130	52	9	1	3	1	1	3	4	2	0
6131	50	9	1	3	1	1	3	4	2	0
6132	48	8	2	2	1	1	3	4	2	0
6133	45	8	2	4	1	1	3	4	2	0

6134	45	8	2	2	1	1	3	4	2	0
6135	39	6	2	2	1	1	3	4	2	0
6136	47	8	2	2	1	1	3	4	2	0
6137	20	3	1	7	1	2	4	4	2	0
6138	18	2	1	7	1	2	3	4	2	0
6139	16	2	2	7	1	2	3	4	2	0
6140	14	1	1	7	1	2	3	4	2	0
6141	22	3	2	7	1	2	3	4	2	0
6142	20	3	1	7	1	2	3	4	2	0
6143	18	2	1	7	1	2	3	4	2	0
6144	18	2	2	7	1	2	3	4	2	0
6145	15	2	1	7	1	2	3	4	2	0
6146	77	14	1	2	1	1	2	4	2	0
6147	65	12	2	2	1	1	2	4	2	0
6148	43	7	1	2	1	1	3	4	2	0
6149	38	6	1	2	1	1	3	4	2	0
6150	32	5	1	3	1	2	3	4	2	0
6151	30	5	2	2	1	2	3	4	2	0
6152	20	3	2	7	1	2	4	4	2	0
6153	18	2	2	7	1	2	3	4	2	0
6154	16	2	1	7	1	2	3	4	2	0
6155	13	1	1	7	1	2	3	4	2	0
6156	10	1	2	7	1	2	3	4	2	0
6157	37	6	2	3	1	1	3	4	2	0
6158	12	1	2	7	1	2	3	4	2	0
6159	10	1	2	7	1	2	3	4	2	0
6160	28	4	2	2	1	1	3	4	2	0
6161	78	14	1	5	1	1	2	4	2	0
6162	74	13	2	1	1	1	3	4	2	0
6163	56	10	1	3	1	1	3	4	2	0
6164	52	9	1	2	1	1	3	4	2	0

6165	48	8	1	3	1	1	3	4	2	0
6166	45	8	1	2	1	1	3	4	2	0
6167	48	8	2	2	1	2	3	4	2	0
6168	18	2	1	7	1	2	3	4	2	0
6169	37	6	2	2	1	1	3	4	2	0
6170	25	4	1	3	1	2	3	4	2	0
6171	46	8	2	2	1	1	3	4	2	0
6172	32	5	1	2	1	2	3	4	2	0
6173	33	5	1	2	1	1	3	4	2	0
6174	40	7	2	2	1	1	3	4	2	0
6175	10	1	1	7	1	2	2	4	2	0
6176	13	1	1	7	1	2	2	4	2	0
6177	16	2	1	7	1	2	3	4	2	0
6178	19	2	2	7	1	2	3	4	2	0
6179	11	1	1	7	1	2	2	4	2	0
6180	30	5	1	2	1	1	2	6	2	0
6181	23	3	2	2	1	1	3	6	2	0
6182	63	11	1	1	1	1	2	3	2	0
6183	54	9	2	2	1	1	3	3	2	0
6184	31	5	1	3	1	2	3	3	2	0
6185	28	4	2	3	1	2	3	3	2	0
6186	25	4	1	2	1	2	3	3	2	0
6187	21	3	2	7	1	2	4	3	2	0
6188	60	11	1	1	3	1	1	3	2	0
6189	58	10	2	1	1	1	3	3	2	0
6190	31	5	2	7	1	2	4	3	2	0
6191	20	3	1	7	1	2	3	3	2	0
6192	18	2	1	7	1	2	3	3	2	0
6193	26	4	2	7	1	2	4	3	2	0
6194	28	4	2	7	1	2	4	3	2	0
6195	12	1	2	7	1	2	2	3	2	0

6196	71	13	1	2	1	1	2	3	2	0
6197	20	3	1	2	1	2	3	3	2	0
6198	55	10	2	2	1	1	3	3	2	0
6199	24	3	1	3	1	1	3	3	2	0
6200	33	5	1	2	1	1	3	3	2	0
6201	77	14	1	6	1	1	4	3	2	0
6202	71	13	2	6	1	1	4	3	2	0
6203	15	2	2	7	1	2	3	3	2	0
6204	28	4	1	1	1	1	2	6	2	0
6205	22	3	2	1	1	1	2	6	2	0
6206	56	10	1	3	3	1	2	3	2	0
6207	54	9	2	2	1	1	3	3	2	0
6208	20	3	2	7	1	2	3	3	2	0
6209	17	2	2	7	1	2	3	3	2	0
6210	15	2	1	7	1	2	3	3	2	0
6211	13	1	1	7	1	2	2	3	2	0
6212	36	6	1	4	1	2	3	3	2	0
6213	54	9	1	2	1	1	2	6	2	0
6214	39	6	2	2	1	1	3	6	2	0
6215	20	3	1	3	1	2	2	6	2	0
6216	18	2	2	7	1	2	3	6	2	0
6217	17	2	2	7	1	2	3	6	2	0
6218	15	2	1	8	1	2	2	6	2	0
6219	92	15	2	5	3	4	2	3	2	0
6220	70	13	1	2	1	1	3	3	2	0
6221	61	11	2	2	1	1	3	3	2	0
6222	66	12	1	3	1	1	3	3	2	0
6223	54	9	1	2	1	1	2	3	2	0
6224	56	10	1	1	1	1	3	3	2	0
6225	63	11	1	1	1	1	3	3	2	0
6226	58	10	1	2	1	1	2	3	2	0

6227	60	11	2	2	1	1	3	3	2	0
6228	52	9	2	2	1	1	3	3	2	0
6229	52	9	2	2	1	1	3	3	2	0
6230	50	9	2	1	1	1	3	3	2	0
6231	48	8	2	2	1	1	3	3	2	0
6232	20	3	2	2	1	2	3	3	2	0
6233	44	7	1	2	1	1	2	3	2	0
6234	27	4	2	3	1	2	3	3	2	0
6235	39	6	1	3	1	1	3	3	2	0
6236	40	7	1	1	1	1	3	3	2	0
6237	35	6	1	2	1	1	3	3	2	0
6238	28	4	2	3	1	2	3	3	2	0
6239	20	3	2	7	1	2	3	3	2	0
6240	16	2	1	7	1	2	3	3	2	0
6241	18	2	1	8	1	2	3	3	2	0
6242	16	2	2	7	1	2	3	3	2	0
6243	14	1	2	7	1	2	3	3	2	0
6244	12	1	2	7	1	2	2	3	2	0
6245	11	1	1	7	1	2	2	3	2	0
6246	39	6	2	3	1	1	3	3	2	0
6247	30	5	2	2	1	1	3	3	2	0
6248	32	5	2	4	1	1	3	3	2	0
6249	28	4	2	2	1	1	3	3	2	0
6250	52	9	1	3	1	1	2	3	2	0
6251	47	8	2	1	1	1	3	3	2	0
6252	21	3	1	7	1	2	3	3	2	0
6253	19	2	1	7	1	2	3	3	2	0
6254	17	2	1	7	1	2	3	3	2	0
6255	15	2	2	7	1	2	3	3	2	0
6256	13	1	1	7	1	2	2	3	2	0
6257	42	7	1	2	1	1	3	3	2	0

6258	39	6	2	2	1	1	3	3	2	0
6259	17	2	1	7	1	2	3	3	2	0
6260	15	2	1	7	1	2	3	3	2	0
6261	13	1	1	7	1	2	3	3	2	0
6262	10	1	1	7	1	2	3	3	2	0
6263	50	9	1	1	1	1	2	3	2	0
6264	30	5	2	2	1	1	3	3	2	0
6265	10	1	1	7	1	2	2	3	2	0
6266	39	6	1	3	1	1	3	6	2	0
6267	30	5	2	3	1	1	3	6	2	0
6268	12	1	2	7	1	2	2	6	2	0
6269	66	12	2	2	1	4	3	4	2	0
6270	65	12	2	2	1	1	3	4	2	0
6271	41	7	2	2	1	1	3	4	2	0
6272	44	7	1	2	1	1	3	4	2	0
6273	20	3	1	3	1	2	2	4	2	0
6274	19	2	1	8	1	2	3	4	2	0
6275	38	6	1	2	1	1	2	4	2	0
6276	33	5	2	3	1	1	3	4	2	0
6277	55	10	1	2	1	1	2	4	2	0
6278	50	9	2	1	1	1	3	4	2	0
6279	66	12	2	1	1	4	3	4	2	0
6280	35	6	1	2	1	1	3	4	2	0
6281	28	4	2	8	1	1	3	4	2	0
6282	77	14	1	1	1	1	2	4	2	0
6283	54	9	2	2	1	1	2	4	2	0
6284	70	13	2	6	1	4	3	4	2	0
6285	32	5	2	2	1	1	3	4	2	0
6286	45	8	2	2	1	1	2	4	2	0
6287	10	1	2	7	1	2	2	4	2	0
6288	12	1	2	7	1	2	2	4	2	0

6289	30	5	2	8	1	2	4	4	2	0
6290	30	5	2	3	1	2	3	4	2	0
6291	28	4	1	7	1	2	4	4	2	0
6292	28	4	1	7	1	2	4	4	2	0
6293	26	4	1	7	1	2	4	4	2	0
6294	40	7	1	2	1	1	3	4	2	0
6295	32	5	2	2	1	1	3	4	2	0
6296	18	2	1	7	1	2	3	4	2	0
6297	16	2	1	7	1	2	3	4	2	0
6298	13	1	1	7	1	2	3	4	2	0
6299	28	4	2	3	1	2	3	4	2	0
6300	26	4	1	2	1	2	2	4	2	0
6301	21	3	1	2	1	2	3	4	2	0
6302	18	2	1	7	1	2	3	4	2	0
6303	49	8	1	2	1	1	2	4	2	0
6304	38	6	2	2	1	1	3	4	2	0
6305	45	8	1	2	1	1	3	4	2	0
6306	40	7	2	2	1	1	3	4	2	0
6307	40	7	1	3	1	1	3	4	2	0
6308	29	4	2	2	1	1	3	4	2	0
6309	11	1	2	7	1	2	2	4	2	0
6310	17	2	2	7	1	2	3	4	2	0
6311	13	1	2	7	1	2	3	4	2	0
6312	11	1	2	7	1	2	3	4	2	0
6313	12	1	1	7	1	2	2	4	2	0
6314	42	7	2	2	1	1	3	4	2	0
6315	46	8	1	2	1	1	2	4	2	0
6316	63	11	1	2	1	1	2	4	2	0
6317	30	5	2	2	1	1	3	4	2	0
6318	60	11	2	2	1	1	2	4	2	0
6319	40	7	2	3	1	1	3	4	2	0

6320	32	5	1	7	1	2	4	4	2	0
6321	86	15	1	5	1	1	2	4	2	0
6322	78	14	2	2	1	1	3	4	2	0
6323	29	4	2	3	1	1	3	4	2	0
6324	68	12	1	2	1	1	2	4	2	0
6325	45	8	2	2	1	1	3	4	2	0
6326	29	4	1	3	1	2	3	4	2	0
6327	38	6	1	2	1	1	2	4	2	0
6328	20	3	1	3	1	1	2	4	2	0
6329	81	15	2	5	1	4	1	4	2	0
6330	42	7	1	2	1	1	3	4	2	0
6331	40	7	2	2	1	1	3	4	2	0
6332	28	4	2	3	1	1	3	4	2	0
6333	47	8	1	1	1	1	2	6	2	0
6334	30	5	2	1	1	1	2	6	2	0
6335	14	1	1	7	1	2	3	6	2	0
6336	13	1	1	7	1	2	3	6	2	0
6337	10	1	1	7	1	2	2	6	2	0
6338	75	14	1	2	1	1	2	4	2	0
6339	66	12	2	2	1	1	3	4	2	0
6340	45	8	1	3	1	1	2	4	2	0
6341	41	7	2	2	1	1	3	4	2	0
6342	27	4	1	3	1	2	3	4	2	0
6343	47	8	1	2	1	1	3	4	2	0
6344	26	4	2	3	1	1	3	4	2	0
6345	77	14	2	2	1	4	3	4	2	0
6346	50	9	1	2	1	1	3	4	2	0
6347	42	7	2	2	1	5	3	4	2	0
6348	57	10	1	2	1	1	3	4	2	0
6349	48	8	2	2	1	1	3	4	2	0
6350	88	15	1	6	1	1	3	4	2	0

6351	67	12	2	2	1	1	3	4	2	0
6352	33	5	2	2	1	2	3	4	2	0
6353	59	10	2	2	1	2	3	4	2	0
6354	49	8	2	2	1	1	3	4	2	0
6355	60	11	1	2	1	1	3	4	2	0
6356	33	5	1	3	1	2	3	4	2	0
6357	32	5	2	2	1	2	3	4	2	0
6358	56	10	1	1	1	1	2	4	2	0
6359	68	12	1	2	1	1	2	4	2	0
6360	56	10	2	2	1	1	2	4	2	0
6361	60	11	1	2	1	1	3	4	2	0
6362	78	14	1	2	1	1	2	4	2	0
6363	70	13	2	2	1	4	3	4	2	0
6364	79	14	1	5	1	1	2	4	2	0
6365	56	10	2	2	1	1	3	4	2	0
6366	53	9	2	1	1	1	2	4	2	0
6367	62	11	1	2	1	1	4	4	2	0
6368	28	4	1	2	1	2	3	4	2	0
6369	25	4	1	2	1	2	3	4	2	0
6370	21	3	2	7	1	2	4	4	2	0
6371	23	3	1	7	1	2	4	4	2	0
6372	87	15	1	5	1	1	2	4	2	0
6373	65	12	2	2	1	1	3	4	2	0
6374	37	6	1	3	1	1	3	4	2	0
6375	26	4	2	2	1	1	3	4	2	0
6376	22	3	2	7	1	2	3	4	2	0
6377	18	2	1	7	1	2	3	4	2	0
6378	66	12	2	1	1	1	2	4	2	0
6379	81	15	1	2	1	1	2	4	2	0
6380	16	2	1	7	1	2	3	4	2	0
6381	15	2	1	7	1	2	3	4	2	0

6382	72	13	1	1	1	1	2	4	2	0
6383	67	12	2	2	1	1	3	4	2	0
6384	21	3	1	8	1	2	2	4	2	0
6385	41	7	1	2	1	1	3	4	2	0
6386	28	4	2	2	1	1	3	4	2	0
6387	65	12	1	2	1	1	3	4	2	0
6388	55	10	2	2	1	1	2	4	2	0
6389	94	15	1	5	1	1	1	4	2	0
6390	73	13	2	2	1	1	2	4	2	0
6391	60	11	1	2	1	1	2	4	2	0
6392	46	8	2	2	1	1	3	4	2	0
6393	48	8	1	3	1	1	2	4	2	0
6394	45	8	2	2	1	2	3	4	2	0
6395	42	7	1	2	1	1	3	4	2	0
6396	40	7	1	2	1	1	3	4	2	0
6397	41	7	2	2	1	1	3	4	2	0
6398	34	5	2	4	1	1	3	4	2	0
6399	29	4	2	3	1	1	3	4	2	0
6400	50	9	1	2	1	1	3	4	2	0
6401	46	8	2	2	1	1	3	4	2	0
6402	77	14	1	2	1	1	2	4	2	0
6403	55	10	2	2	1	1	2	4	2	0
6404	70	13	1	3	1	1	3	4	2	0
6405	67	12	2	2	1	1	3	4	2	0
6406	28	4	2	3	1	2	3	4	2	0
6407	24	3	1	2	1	2	2	4	2	0
6408	20	3	1	7	1	2	3	4	2	0
6409	18	2	2	7	1	2	3	4	2	0
6410	16	2	2	7	1	2	3	4	2	0
6411	57	10	2	1	1	4	2	4	2	0
6412	31	5	2	3	1	2	3	4	2	0

6413	29	4	1	2	1	2	3	4	2	0
6414	26	4	2	7	1	2	4	4	2	0
6415	22	3	1	2	1	2	2	4	2	0
6416	41	7	2	2	1	1	3	4	2	0
6417	66	12	1	2	1	1	3	4	2	0
6418	48	8	2	2	1	1	3	4	2	0
6419	40	7	2	2	1	1	3	4	2	0
6420	15	1	1	7	1	2	3	4	2	0
6421	21	3	1	2	1	2	3	4	2	0
6422	82	15	1	5	1	1	2	4	2	0
6423	77	14	2	2	1	1	3	4	2	0
6424	60	11	2	2	1	1	3	4	2	0
6425	36	6	1	3	3	2	3	4	2	0
6426	43	7	1	2	1	1	3	4	2	0
6427	17	2	1	8	1	2	2	4	2	0
6428	29	4	1	2	1	2	3	4	2	0
6429	40	7	2	2	1	1	3	4	2	0
6430	80	15	1	2	1	1	2	4	2	0
6431	40	7	1	3	1	1	3	4	2	0
6432	28	4	2	3	1	1	3	4	2	0
6433	60	11	2	1	1	1	3	4	2	0
6434	72	13	2	2	1	1	2	4	2	0
6435	47	8	2	2	1	1	3	4	2	0
6436	66	12	1	2	1	1	2	4	2	0
6437	33	5	1	4	1	2	3	4	2	0
6438	56	10	2	2	1	1	3	4	2	0
6439	50	9	2	2	1	1	2	4	2	0
6440	70	13	2	1	1	1	2	4	2	0
6441	33	5	2	3	1	2	3	4	2	0
6442	43	7	2	2	1	1	3	4	2	0
6443	32	5	2	2	1	1	3	4	2	0

6444	47	8	2	3	1	1	3	4	2	0
6445	41	7	1	2	1	1	3	4	2	0
6446	64	11	1	1	1	1	3	4	2	0
6447	26	4	1	2	1	1	2	4	2	0
6448	54	9	2	2	1	1	3	4	2	0
6449	30	5	1	2	1	1	2	4	2	0
6450	16	2	1	7	1	2	3	4	2	0
6451	61	11	1	1	1	1	2	4	2	0
6452	18	2	1	3	1	2	2	4	2	0
6453	57	10	1	3	1	1	2	4	2	0
6454	52	9	2	2	1	1	3	4	2	0
6455	15	2	1	7	1	2	3	4	2	0
6456	12	1	1	7	1	2	2	4	2	0
6457	70	13	1	2	1	1	2	4	2	0
6458	50	9	2	2	1	1	3	4	2	0
6459	42	7	2	2	1	4	3	4	2	0
6460	32	5	2	3	1	2	3	4	2	0
6461	26	4	1	2	1	2	3	4	2	0
6462	60	11	1	3	1	1	3	4	2	0
6463	41	7	2	2	1	1	3	4	2	0
6464	47	8	1	2	1	1	2	4	2	0
6465	15	2	1	7	1	2	3	4	2	0
6466	32	5	2	2	1	1	3	4	2	0
6467	36	6	2	2	1	1	3	4	2	0
6468	35	6	1	2	1	1	2	4	2	0
6469	30	5	2	2	1	1	3	4	2	0
6470	10	1	2	7	1	2	2	4	2	0
6471	64	11	1	1	1	1	3	4	2	0
6472	45	8	2	1	1	1	3	4	2	0
6473	19	2	1	7	1	2	3	4	2	0
6474	13	1	1	7	1	2	3	4	2	0

6475	40	7	1	2	1	1	2	6	2	0
6476	28	4	2	2	1	1	3	6	2	0
6477	12	1	2	7	1	2	2	6	2	0
6478	39	6	2	2	1	1	3	4	2	0
6479	56	10	1	1	1	1	3	4	2	0
6480	50	9	2	1	1	2	3	4	2	0
6481	82	15	2	1	1	4	2	4	2	0
6482	45	8	1	2	1	1	3	4	2	0
6483	49	8	1	2	1	1	3	4	2	0
6484	60	11	2	2	1	1	3	4	2	0
6485	39	6	2	2	1	1	3	4	2	0
6486	50	9	1	1	1	1	3	4	2	0
6487	13	1	1	7	1	2	2	4	2	0
6488	28	4	2	4	1	2	3	4	2	0
6489	33	5	1	3	1	1	2	4	2	0
6490	30	5	2	3	1	1	3	4	2	0
6491	42	7	2	4	1	1	3	4	2	0
6492	35	6	1	2	1	1	3	4	2	0
6493	19	2	1	7	1	2	3	4	2	0
6494	18	2	1	7	1	2	3	4	2	0
6495	26	4	2	2	1	2	3	4	2	0
6496	48	8	1	1	1	1	2	4	2	0
6497	38	6	2	2	1	1	2	4	2	0
6498	15	2	1	7	1	2	3	4	2	0
6499	12	1	1	7	1	2	2	4	2	0
6500	10	1	1	7	1	2	2	4	2	0
6501	58	10	1	3	1	1	2	4	2	0
6502	55	10	2	2	1	1	3	4	2	0
6503	46	8	1	1	1	1	2	4	2	0
6504	38	6	2	2	1	1	3	4	2	0
6505	40	7	1	2	1	1	2	4	2	0

6506	26	4	2	3	1	1	3	4	2	0
6507	10	1	1	7	1	2	2	4	2	0
6508	15	2	2	7	1	2	2	4	2	0
6509	10	1	1	7	1	2	2	4	2	0
6510	18	2	2	7	1	2	3	4	2	0
6511	22	3	1	3	1	2	2	4	2	0
6512	11	1	1	7	1	2	2	4	2	0
6513	72	13	1	2	1	1	2	4	2	0
6514	58	10	2	2	1	1	3	4	2	0
6515	30	5	1	2	1	2	3	4	2	0
6516	47	8	1	2	1	1	3	4	2	0
6517	60	11	2	1	1	4	3	4	2	0
6518	65	12	1	2	1	1	3	4	2	0
6519	58	10	2	2	1	1	3	4	2	0
6520	36	6	2	2	1	1	3	4	2	0
6521	42	7	1	1	1	1	2	4	2	0
6522	27	4	2	2	1	1	3	4	2	0
6523	36	6	1	3	1	2	3	4	2	0
6524	20	3	1	7	1	2	4	4	2	0
6525	16	2	1	7	1	2	3	4	2	0
6526	13	1	1	7	1	2	2	4	2	0
6527	19	2	2	7	1	2	3	4	2	0
6528	17	2	1	7	1	2	3	4	2	0
6529	18	2	2	7	1	2	3	4	2	0
6530	31	5	1	3	1	2	3	4	2	0
6531	26	4	1	3	1	2	3	4	2	0
6532	12	1	1	7	1	2	2	4	2	0
6533	19	2	2	7	1	2	3	4	2	0
6534	14	1	1	7	1	2	2	4	2	0
6535	37	6	1	2	1	1	3	6	2	0
6536	26	4	2	2	1	1	2	6	2	0

6537	56	10	1	2	1	1	2	4	2	0
6538	49	8	2	2	1	1	3	4	2	0
6539	70	13	2	1	1	4	2	4	2	0
6540	48	8	1	2	1	1	3	4	2	0
6541	30	5	1	3	1	1	3	4	2	0
6542	79	14	1	1	1	1	2	4	2	0
6543	67	12	2	2	1	1	2	4	2	0
6544	53	9	2	2	1	1	3	4	2	0
6545	11	1	1	7	1	2	2	4	2	0
6546	13	1	2	7	1	2	3	4	2	0
6547	13	1	1	7	1	2	3	4	2	0
6548	20	3	1	7	1	2	3	4	2	0
6549	37	6	2	2	1	2	3	4	2	0
6550	42	7	1	3	1	1	2	4	2	0
6551	39	6	1	2	1	1	2	4	2	0
6552	20	3	2	7	1	2	3	4	2	0
6553	66	12	1	1	1	1	2	4	2	0
6554	50	9	2	1	1	1	2	4	2	0
6555	57	10	1	4	1	1	3	4	2	0
6556	14	1	1	7	1	2	3	4	2	0
6557	17	2	1	7	1	2	3	4	2	0
6558	45	8	2	2	1	1	3	4	2	0
6559	24	3	2	7	1	2	4	4	2	0
6560	60	11	1	3	1	1	2	6	2	0
6561	23	3	1	3	1	2	4	6	2	0
6562	56	10	2	2	1	1	3	6	2	0
6563	45	8	2	3	1	5	3	4	2	0
6564	29	4	1	3	1	2	3	4	2	0
6565	37	6	2	2	1	1	3	4	2	0
6566	43	7	2	4	1	1	4	4	2	0
6567	77	14	1	5	1	1	2	4	2	0

6568	51	9	2	2	1	1	3	4	2	0
6569	81	15	2	5	1	4	2	4	2	0
6570	50	9	1	4	1	1	3	4	2	0
6571	62	11	1	1	1	1	1	4	2	0
6572	28	4	1	3	1	2	3	4	2	0
6573	24	3	1	2	1	2	2	4	2	0
6574	19	2	1	3	1	2	3	4	2	0
6575	17	2	2	7	1	2	3	4	2	0
6576	14	1	2	7	1	2	3	4	2	0
6577	15	2	1	7	1	2	3	4	2	0
6578	17	2	1	7	1	2	3	4	2	0
6579	14	1	1	7	1	2	3	4	2	0
6580	12	1	2	7	1	2	2	4	2	0
6581	58	10	2	2	1	1	3	4	2	0
6582	36	6	2	2	1	1	3	4	2	0
6583	93	15	1	5	3	1	2	4	2	0
6584	71	13	2	2	1	1	2	4	2	0
6585	53	9	1	1	1	1	3	4	2	0
6586	12	1	1	7	1	2	3	4	2	0
6587	19	2	2	7	1	2	3	4	2	0
6588	45	8	2	2	1	1	3	4	2	0
6589	36	6	1	3	1	1	2	4	2	0
6590	10	1	2	7	1	2	2	4	2	0
6591	27	4	1	2	1	2	3	4	2	0
6592	18	2	1	3	1	2	2	4	2	0
6593	30	5	2	2	1	1	3	4	2	0
6594	23	3	2	3	1	2	3	4	2	0
6595	13	1	2	7	1	2	2	4	2	0
6596	33	5	1	3	1	1	2	4	2	0
6597	48	8	1	2	1	1	3	4	2	0
6598	34	5	2	2	1	1	3	4	2	0

6599	28	4	1	3	1	2	2	4	2	0
6600	35	6	1	3	1	2	3	4	2	0
6601	59	10	1	2	1	1	3	4	2	0
6602	55	10	2	2	1	1	3	4	2	0
6603	40	7	2	1	1	2	2	4	2	0
6604	86	15	1	6	1	1	3	4	2	0
6605	64	11	2	2	1	1	3	4	2	0
6606	25	4	1	3	1	2	3	4	2	0
6607	40	7	1	3	1	1	2	4	2	0
6608	30	5	2	2	1	1	3	4	2	0
6609	39	6	1	1	1	1	3	4	2	0
6610	48	8	1	1	1	1	2	4	2	0
6611	43	7	2	2	1	1	3	4	2	0
6612	27	4	2	3	1	1	3	4	2	0
6613	67	12	2	2	1	4	2	4	2	0
6614	47	8	1	2	1	1	3	4	2	0
6615	42	7	2	2	1	2	3	4	2	0
6616	31	5	1	3	1	2	3	4	2	0
6617	45	8	1	1	1	1	3	4	2	0
6618	40	7	2	2	1	1	3	4	2	0
6619	25	4	1	7	1	2	4	4	2	0
6620	53	9	1	4	1	1	3	4	2	0
6621	48	8	2	4	1	1	3	4	2	0
6622	16	2	1	7	1	2	3	4	2	0
6623	14	1	1	7	1	2	3	4	2	0
6624	12	1	2	7	1	2	3	4	2	0
6625	60	11	1	1	1	1	2	4	2	0
6626	20	3	2	7	1	2	4	4	2	0
6627	48	8	2	2	1	1	3	4	2	0
6628	16	2	2	7	1	2	3	4	2	0
6629	36	6	1	3	1	1	3	6	2	0

6630	11	1	2	7	1	2	2	4	2	0
6631	30	5	1	3	1	2	2	4	1	0
6632	66	12	1	2	1	1	2	4	2	0
6633	50	9	2	2	1	1	2	4	2	0
6634	21	3	2	7	1	2	3	4	2	0
6635	13	1	1	7	1	2	2	4	2	0
6636	17	2	2	7	1	2	3	4	2	0
6637	36	6	2	3	1	1	3	4	2	0
6638	56	10	1	1	1	1	3	4	2	0
6639	47	8	2	3	1	1	3	4	2	0
6640	79	14	1	1	1	1	2	4	2	0
6641	38	6	1	1	1	2	2	4	2	0
6642	61	11	2	2	1	1	3	4	2	0
6643	41	7	1	2	1	1	2	4	2	0
6644	38	6	2	2	1	1	3	4	2	0
6645	35	6	1	3	1	1	2	4	2	0
6646	33	5	2	2	1	1	3	4	2	0
6647	19	2	1	7	1	2	3	4	2	0
6648	13	1	2	7	1	2	3	4	2	0
6649	17	2	1	7	1	2	3	4	2	0
6650	10	1	2	7	1	2	2	4	2	0
6651	92	15	2	5	1	4	1	4	2	0
6652	38	6	1	2	1	1	3	4	2	0
6653	33	5	2	2	1	1	4	4	2	0
6654	43	7	1	2	1	1	3	4	2	0
6655	60	11	1	2	1	1	2	4	2	0
6656	59	10	2	3	1	1	2	4	2	0
6657	55	10	2	2	1	1	3	4	2	0
6658	63	11	1	2	1	1	2	4	2	0
6659	17	2	1	7	1	2	3	4	2	0
6660	37	6	2	3	1	1	3	4	2	0

6661	36	6	1	2	1	2	3	4	2	0
6662	39	6	1	3	1	1	3	4	2	0
6663	16	2	1	7	1	2	3	4	2	0
6664	13	1	2	7	1	2	2	4	2	0
6665	18	2	2	7	1	2	3	4	2	0
6666	15	2	1	7	1	2	3	4	2	0
6667	25	4	2	2	1	1	3	4	2	0
6668	23	3	1	7	1	2	4	4	2	0
6669	68	12	1	3	1	1	2	4	2	0
6670	66	12	2	2	1	1	2	4	2	0
6671	40	7	1	1	1	1	2	4	2	0
6672	31	5	2	2	1	1	3	4	2	0
6673	37	6	2	2	1	2	3	4	2	0
6674	29	4	2	3	1	1	2	4	2	0
6675	22	3	2	7	1	2	3	4	2	0
6676	49	8	1	1	1	1	2	4	2	0
6677	35	6	1	2	1	1	3	4	2	0
6678	27	4	2	2	1	1	3	4	2	0
6679	39	6	1	2	1	1	2	4	2	0
6680	26	4	1	3	1	2	3	4	2	0
6681	34	5	2	1	1	1	3	4	2	0
6682	25	4	2	3	1	1	3	4	2	0
6683	74	13	2	5	1	4	2	4	2	0
6684	55	10	1	2	1	1	3	4	2	0
6685	23	3	1	3	1	2	3	4	2	0
6686	30	5	1	2	1	2	3	4	2	0
6687	22	3	2	2	1	1	3	4	2	0
6688	35	6	1	2	1	1	2	4	2	0
6689	21	3	1	3	1	2	2	4	2	0
6690	63	11	2	2	1	1	3	4	2	0
6691	47	8	1	1	1	1	2	4	2	0

6692	26	4	2	1	1	1	2	4	2	0
6693	19	2	1	3	1	2	2	4	2	0
6694	62	11	1	4	1	1	3	4	2	0
6695	19	2	2	7	1	2	3	4	2	0
6696	17	2	1	7	1	2	3	4	2	0
6697	52	9	2	2	1	1	3	4	2	0
6698	16	2	2	7	1	2	3	4	2	0
6699	13	8	1	7	1	2	2	4	2	0
6700	41	7	1	2	1	1	3	4	2	0
6701	35	6	2	2	1	1	3	4	2	0
6702	65	12	1	1	1	1	2	4	2	0
6703	24	3	1	3	1	2	2	4	2	0
6704	50	9	2	2	1	1	3	4	2	0
6705	13	1	2	7	1	2	3	4	2	0
6706	69	12	1	1	1	4	3	4	2	0
6707	32	5	1	2	1	1	3	4	2	0
6708	34	5	2	3	1	2	3	4	2	0
6709	31	5	1	2	1	2	3	4	2	0
6710	22	3	2	7	1	2	3	4	2	0
6711	29	4	2	3	1	1	3	4	2	0
6712	66	12	1	2	1	1	2	4	2	0
6713	47	8	2	2	1	1	2	4	2	0
6714	26	4	2	8	1	2	2	4	2	0
6715	55	10	1	2	1	1	2	4	2	0
6716	67	12	1	1	1	1	2	4	2	0
6717	12	1	2	7	1	2	2	4	2	0
6718	40	7	2	2	1	3	3	4	2	0
6719	17	2	1	7	1	2	3	4	2	0
6720	20	3	1	7	1	2	4	4	2	0
6721	37	6	1	2	1	2	3	4	2	0
6722	14	1	1	7	1	2	3	4	2	0

6723	27	4	1	2	1	2	3	4	2	0
6724	20	3	1	3	1	2	3	4	2	0
6725	14	1	1	7	1	2	3	4	2	0
6726	11	1	2	7	1	2	2	4	2	0
6727	16	2	2	7	1	2	3	4	2	0
6728	18	2	2	7	1	2	3	4	2	0
6729	48	8	2	2	1	1	3	4	2	0
6730	15	2	2	7	1	2	3	4	2	0
6731	19	2	2	7	1	2	3	4	2	0
6732	17	2	2	7	1	2	3	4	2	0
6733	12	1	2	7	1	2	2	4	2	0
6734	17	2	2	7	1	2	3	4	2	0
6735	10	1	1	7	1	2	2	4	2	0
6736	15	2	2	7	1	2	3	4	2	0
6737	16	2	1	7	1	2	3	4	2	0
6738	56	10	2	3	1	1	2	1	2	0
6739	14	1	1	7	1	2	3	1	2	0
6740	15	2	1	7	1	2	3	1	2	0
6741	12	1	2	7	1	2	2	1	2	0
6742	19	2	1	8	1	2	3	1	2	0
6743	67	12	1	3	1	1	2	1	2	0
6744	23	3	1	2	1	2	2	1	2	0
6745	27	4	1	2	1	2	2	1	2	0
6746	60	11	2	2	1	4	3	1	2	0
6747	15	2	1	7	1	2	3	1	2	0
6748	12	1	2	7	1	2	2	1	2	0
6749	17	2	1	7	1	2	3	1	2	0
6750	56	10	2	1	1	4	2	1	2	0
6751	34	5	1	2	1	2	3	1	2	0
6752	20	3	2	7	1	2	3	1	2	0
6753	10	1	2	7	1	2	2	1	2	0

6754	14	1	2	7	1	2	2	1	2	0
6755	71	13	1	2	1	1	2	1	2	0
6756	19	2	1	7	1	2	3	1	2	0
6757	11	1	1	7	1	2	2	1	2	0
6758	53	9	2	2	1	1	3	1	2	0
6759	23	3	1	7	1	2	4	1	2	0
6760	33	5	1	2	1	2	3	1	2	0
6761	45	8	1	2	1	1	3	1	2	0
6762	51	9	2	4	1	1	3	1	2	0
6763	54	9	1	2	1	1	3	1	2	0
6764	19	2	1	7	1	2	3	1	2	0
6765	37	6	1	2	1	1	3	1	2	0
6766	26	4	2	3	1	1	3	1	2	0
6767	30	5	2	2	1	2	3	1	2	0
6768	21	3	1	3	1	2	2	1	2	0
6769	13	1	2	7	1	2	2	1	2	0
6770	65	12	2	2	1	4	2	1	2	0
6771	42	7	1	2	1	1	3	1	2	0
6772	37	6	2	2	1	1	3	1	2	0
6773	11	1	1	7	1	2	2	1	2	0
6774	39	6	1	3	1	1	2	1	2	0
6775	17	2	1	7	1	2	3	1	2	0
6776	19	2	1	7	1	2	3	1	2	0
6777	88	15	2	5	3	4	2	1	2	0
6778	70	13	1	2	1	1	3	1	2	0
6779	68	12	1	2	1	1	3	1	2	0
6780	54	9	1	2	1	1	3	1	2	0
6781	35	6	1	3	1	2	3	1	2	0
6782	18	2	1	7	1	2	3	1	2	0
6783	57	10	2	2	1	1	3	1	2	0
6784	48	8	2	2	1	1	3	1	2	0

6785	37	6	2	4	1	1	3	1	2	0
6786	16	2	2	7	1	2	3	1	2	0
6787	15	2	1	7	1	2	3	1	2	0
6788	21	3	2	2	1	1	3	1	2	0
6789	28	4	1	2	1	1	2	1	2	0
6790	11	1	2	7	1	2	2	1	2	0
6791	17	2	2	7	1	2	2	1	2	0
6792	10	1	2	7	1	2	2	1	2	0
6793	82	15	1	1	1	1	1	1	2	0
6794	69	12	2	2	1	1	2	1	2	0
6795	26	4	1	2	1	2	3	1	2	0
6796	29	4	2	3	1	2	3	1	2	0
6797	27	4	1	3	1	2	3	1	2	0
6798	38	6	1	2	1	1	2	1	2	0
6799	29	4	2	2	1	1	3	1	2	0
6800	52	9	2	2	1	4	3	1	2	0
6801	26	4	2	7	1	2	4	1	2	0
6802	91	15	1	5	3	1	1	1	2	0
6803	39	6	1	3	1	1	3	1	2	0
6804	69	12	1	2	1	1	3	1	2	0
6805	64	11	1	2	1	1	3	1	2	0
6806	54	9	1	4	1	1	3	1	2	0
6807	82	15	2	2	1	1	2	1	2	0
6808	26	4	1	2	1	2	2	1	2	0
6809	20	3	2	7	1	2	4	1	2	0
6810	30	5	2	3	1	1	2	1	2	0
6811	60	11	2	2	1	1	2	1	2	0
6812	48	8	1	3	1	1	3	1	2	0
6813	40	7	2	2	1	1	3	1	2	0
6814	36	6	1	3	1	1	3	1	2	0
6815	26	4	2	3	1	1	3	1	2	0

6816	44	7	1	2	1	1	3	1	2	0
6817	46	8	2	4	1	1	3	1	2	0
6818	65	12	1	2	1	1	2	1	2	0
6819	16	2	1	7	1	2	3	1	2	0
6820	80	15	1	2	1	4	1	1	2	0
6821	21	3	2	7	1	2	4	1	2	0
6822	63	11	2	2	1	1	2	1	2	0
6823	69	12	2	2	1	1	2	1	2	0
6824	47	8	1	1	1	1	3	1	2	0
6825	72	13	2	2	1	1	1	1	2	0
6826	17	2	1	7	1	2	3	1	2	0
6827	31	5	2	2	1	1	3	1	2	0
6828	52	9	1	2	1	1	2	1	2	0
6829	41	7	1	2	1	1	2	1	2	0
6830	48	8	1	4	1	1	2	1	2	0
6831	73	13	1	2	1	1	2	1	2	0
6832	46	8	1	2	1	1	3	1	2	0
6833	61	11	2	2	1	1	2	1	2	0
6834	57	10	2	1	1	1	3	1	2	0
6835	33	5	1	2	1	2	3	1	2	0
6836	66	12	1	3	1	1	2	1	2	0
6837	83	15	1	6	1	1	4	1	2	0
6838	59	10	1	2	1	4	2	1	2	0
6839	46	8	2	2	1	1	3	1	2	0
6840	76	14	2	6	1	1	4	1	2	0
6841	50	9	2	2	1	1	3	1	2	0
6842	40	7	2	2	1	2	3	1	2	0
6843	33	5	1	3	1	1	3	6	2	0
6844	24	3	2	2	1	1	3	6	2	0
6845	66	12	1	2	1	1	3	1	2	0
6846	57	10	2	2	1	1	3	1	2	0

6847	48	8	1	1	1	1	3	6	2	0
6848	35	6	2	3	1	1	3	6	2	0
6849	39	6	1	2	1	1	3	1	2	0
6850	45	8	1	1	1	1	2	1	2	0
6851	81	15	1	1	1	1	2	1	2	0
6852	68	12	2	1	1	1	2	1	2	0
6853	37	6	2	2	1	1	3	1	2	0
6854	40	7	1	2	1	1	3	1	2	0
6855	31	5	2	2	1	1	3	1	2	0
6856	50	9	1	1	1	1	2	1	2	0
6857	34	5	2	2	1	1	3	1	2	0
6858	78	14	1	5	1	1	2	1	2	0
6859	51	9	1	2	1	1	3	1	2	0
6860	65	12	2	2	1	1	3	1	2	0
6861	33	5	2	2	1	1	3	1	2	0
6862	44	7	1	2	1	1	3	1	2	0
6863	36	6	1	2	1	2	3	1	2	0
6864	40	7	2	2	1	1	3	1	2	0
6865	14	1	2	7	1	2	3	1	2	0
6866	10	1	2	7	1	2	2	1	2	0
6867	14	1	2	7	1	2	3	1	2	0
6868	46	8	1	2	1	1	3	1	2	0
6869	49	8	1	1	1	1	3	1	2	0
6870	38	6	2	2	1	1	3	1	2	0
6871	12	1	1	7	1	2	2	1	2	0
6872	25	4	2	7	1	2	3	1	2	0
6873	27	4	1	2	1	2	3	1	2	0
6874	66	12	1	2	1	1	3	1	2	0
6875	13	1	1	7	1	2	2	1	2	0
6876	79	14	2	2	1	4	2	1	2	0
6877	26	4	1	3	1	2	3	1	2	0

6878	23	3	1	7	1	2	3	1	2	0
6879	30	5	2	2	1	1	3	1	2	0
6880	39	6	1	2	1	1	3	1	2	0
6881	21	3	1	8	1	2	3	1	2	0
6882	37	6	2	2	1	2	3	1	2	0
6883	20	3	2	7	1	2	3	1	2	0
6884	10	1	1	7	1	2	2	1	2	0
6885	13	1	1	7	1	2	3	1	2	0
6886	47	8	1	3	1	2	3	1	2	0
6887	18	2	1	7	1	2	3	1	2	0
6888	15	2	1	7	1	2	3	1	2	0
6889	11	1	1	7	1	2	2	1	2	0
6890	60	11	1	2	1	1	3	1	2	0
6891	17	2	2	7	1	2	3	1	2	0
6892	19	2	1	7	1	2	3	1	2	0
6893	10	1	1	7	1	2	2	1	2	0
6894	15	2	1	7	1	2	3	1	2	0
6895	28	4	1	2	1	2	3	1	2	0
6896	31	5	2	3	1	2	3	1	2	0
6897	30	5	2	2	1	1	3	1	2	0
6898	37	6	1	2	1	1	3	1	2	0
6899	17	2	1	7	1	2	3	1	2	0
6900	12	1	1	7	1	2	2	1	2	0
6901	20	3	2	7	1	2	3	1	2	0
6902	18	2	2	7	1	2	3	1	2	0
6903	75	14	1	2	1	1	2	1	2	0
6904	57	10	1	2	1	1	3	1	2	0
6905	10	1	2	7	1	2	2	1	2	0
6906	70	13	2	2	1	1	3	1	2	0
6907	30	5	1	3	1	2	3	1	2	0
6908	38	6	2	2	1	2	3	1	2	0

6909	43	7	1	2	1	1	3	1	2	0
6910	35	6	2	2	1	1	3	1	2	0
6911	54	9	2	2	1	1	3	1	2	0
6912	43	7	1	3	1	1	3	1	2	0
6913	70	13	1	1	1	1	3	1	2	0
6914	42	7	1	3	1	1	3	1	2	0
6915	68	12	2	2	1	1	2	1	2	0
6916	34	5	1	2	1	2	3	1	2	0
6917	40	7	1	2	1	1	3	1	2	0
6918	36	6	2	2	1	1	3	1	2	0
6919	17	2	1	7	1	2	3	1	2	0
6920	38	6	1	2	1	1	3	1	2	0
6921	25	4	2	3	1	1	3	1	2	0
6922	60	11	1	3	1	1	3	1	2	0
6923	34	5	2	2	1	1	3	1	2	0
6924	46	8	2	1	1	1	2	1	2	0
6925	56	10	2	2	1	1	3	1	2	0
6926	47	8	1	3	1	1	3	1	2	0
6927	60	11	1	1	1	1	2	1	2	0
6928	41	7	1	2	1	1	3	1	2	0
6929	58	10	1	3	1	1	2	1	2	0
6930	31	5	1	2	1	1	3	1	2	0
6931	27	4	2	3	1	2	3	1	2	0
6932	37	6	1	2	1	1	3	1	2	0
6933	35	6	1	2	1	1	2	1	2	0
6934	15	2	1	7	1	2	3	1	2	0
6935	11	1	1	7	1	2	2	1	2	0
6936	16	2	2	7	1	2	3	1	2	0
6937	45	8	2	3	1	1	3	1	2	0
6938	50	9	2	2	1	4	2	1	2	0
6939	18	2	1	7	1	2	3	1	2	0

6940	43	7	1	3	1	1	2	1	2	0
6941	40	7	2	3	1	2	3	1	2	0
6942	62	11	2	2	1	4	3	6	2	0
6943	26	4	1	2	1	1	2	6	2	0
6944	23	3	2	2	1	1	3	6	2	0
6945	30	5	1	2	1	2	3	6	2	0
6946	28	4	2	7	1	2	4	6	2	0
6947	40	7	2	2	1	5	3	1	2	0
6948	12	1	2	7	1	2	2	1	2	0
6949	48	8	1	2	1	1	2	6	2	0
6950	36	6	2	2	1	1	2	6	2	0
6951	13	1	1	7	1	2	2	6	2	0
6952	11	1	1	7	1	2	2	6	2	0
6953	15	2	2	8	1	2	2	6	2	0
6954	72	13	2	2	1	4	3	1	2	0
6955	35	6	1	3	1	1	3	1	2	0
6956	28	4	2	2	1	1	3	1	2	0
6957	46	8	1	3	1	1	3	1	2	0
6958	31	5	2	3	1	1	3	1	2	0
6959	42	7	1	2	1	1	3	1	2	0
6960	34	5	2	2	1	1	3	1	2	0
6961	65	12	1	2	1	1	2	1	2	0
6962	51	9	2	3	1	1	2	1	2	0
6963	30	5	2	3	1	2	3	1	2	0
6964	23	3	1	3	1	2	3	1	2	0
6965	26	4	2	2	1	2	3	1	2	0
6966	77	14	1	2	1	1	2	1	2	0
6967	72	13	2	2	1	1	3	1	2	0
6968	47	8	2	2	1	2	3	1	2	0
6969	41	7	1	2	1	1	3	1	2	0
6970	29	4	2	3	1	1	3	1	2	0

6971	38	6	1	2	1	1	3	1	2	0
6972	37	6	2	2	1	1	3	1	2	0
6973	44	8	1	2	1	1	3	1	2	0
6974	82	15	2	2	1	4	2	1	2	0
6975	66	12	1	3	1	1	2	1	2	0
6976	65	12	2	2	1	1	3	1	2	0
6977	46	8	1	2	1	1	3	1	2	0
6978	39	6	2	2	1	1	3	1	2	0
6979	79	14	1	2	1	1	3	1	2	0
6980	42	7	1	2	1	1	3	1	2	0
6981	31	5	2	2	1	1	3	1	2	0
6982	29	4	2	7	1	2	4	1	2	0
6983	68	12	2	2	1	1	3	1	2	0
6984	58	10	2	2	1	1	3	1	2	0
6985	28	4	1	3	1	2	3	1	2	0
6986	71	13	1	2	1	1	3	1	2	0
6987	35	6	2	3	1	2	3	1	2	0
6988	40	7	2	2	1	1	3	1	2	0
6989	45	8	1	3	1	1	2	1	2	0
6990	66	12	2	2	1	1	3	1	2	0
6991	56	10	1	4	1	1	3	1	2	0
6992	39	6	2	2	1	2	3	1	2	0
6993	65	12	1	1	1	1	3	1	2	0
6994	62	11	2	2	1	1	2	1	2	0
6995	55	10	2	1	1	1	3	1	2	0
6996	29	4	1	2	1	1	3	1	2	0
6997	20	3	2	3	1	1	2	1	2	0
6998	34	5	2	1	1	1	2	1	2	0
6999	42	7	1	1	1	1	2	1	2	0
7000	67	12	1	3	1	1	2	1	2	0
7001	57	9	2	2	1	1	3	1	2	0

7002	40	7	2	2	1	1	3	1	2	0
7003	48	8	1	1	1	1	3	1	2	0
7004	39	6	2	3	1	1	3	1	2	0
7005	46	8	1	2	1	1	3	1	2	0
7006	83	15	1	2	1	1	2	1	2	0
7007	53	9	2	2	1	1	3	1	2	0
7008	68	12	2	2	1	1	2	1	2	0
7009	41	7	1	3	1	1	3	1	2	0
7010	38	6	1	2	1	1	3	1	2	0
7011	32	5	1	2	1	2	3	1	2	0
7012	27	4	2	3	1	2	3	1	2	0
7013	10	1	1	7	1	2	2	1	2	0
7014	17	2	1	7	1	2	3	1	2	0
7015	19	2	2	7	1	2	3	1	2	0
7016	66	12	1	2	1	1	3	1	2	0
7017	50	9	2	2	1	1	3	1	2	0
7018	77	14	1	1	1	1	2	1	2	0
7019	69	12	2	2	1	1	3	1	2	0
7020	18	2	1	7	1	2	3	1	2	0
7021	13	1	1	7	1	2	3	1	2	0
7022	41	7	1	2	1	1	3	1	2	0
7023	38	6	2	3	1	1	3	1	2	0
7024	14	2	2	7	1	2	2	1	2	0
7025	65	12	1	4	1	1	3	1	2	0
7026	12	1	2	7	1	2	2	1	2	0
7027	58	10	2	1	1	1	3	1	2	0
7028	78	14	2	5	1	4	2	1	2	0
7029	53	9	1	2	1	1	3	1	2	0
7030	44	7	2	2	1	1	3	1	2	0
7031	92	15	1	5	1	4	2	1	2	0
7032	57	10	1	2	1	1	3	1	2	0

7033	51	9	2	3	1	1	3	1	2	0
7034	64	11	1	2	1	1	2	1	2	0
7035	40	7	2	4	1	2	3	1	2	0
7036	68	12	1	2	1	1	2	1	2	0
7037	72	13	1	1	1	1	2	1	2	0
7038	50	9	2	3	1	1	3	1	2	0
7039	54	9	2	2	1	1	3	1	2	0
7040	59	10	2	4	1	1	3	1	2	0
7041	50	9	1	2	1	1	3	1	2	0
7042	85	15	2	1	1	4	1	1	2	0
7043	62	11	1	3	1	1	3	1	2	0
7044	17	2	1	7	1	2	3	1	2	0
7045	19	2	1	7	1	2	3	1	2	0
7046	15	2	2	7	1	2	3	1	2	0
7047	23	3	2	7	1	2	3	1	2	0
7048	12	1	1	7	1	2	2	1	2	0
7049	24	3	2	8	1	2	2	1	2	0
7050	68	12	1	2	1	1	3	1	2	0
7051	20	3	2	7	1	2	3	1	2	0
7052	23	3	2	8	1	2	3	1	2	0
7053	49	8	2	3	1	1	3	1	2	0
7054	18	2	1	7	1	2	3	1	2	0
7055	70	13	1	2	1	1	2	1	2	0
7056	90	15	1	5	3	1	1	1	2	0
7057	12	1	1	7	1	2	2	1	2	0
7058	14	1	1	7	1	2	3	1	2	0
7059	25	4	1	7	1	2	3	1	2	0
7060	16	2	1	7	1	2	3	1	2	0
7061	21	3	1	3	1	2	2	1	2	0
7062	77	14	2	2	1	1	2	1	2	0
7063	65	12	2	2	1	1	3	1	2	0

7064	17	2	2	7	1	2	3	1	2	0
7065	20	3	1	7	1	2	3	1	2	0
7066	59	10	1	3	1	1	3	1	2	0
7067	13	1	2	7	1	2	2	1	2	0
7068	61	11	1	2	1	1	3	1	2	0
7069	18	2	1	7	1	2	3	1	2	0
7070	54	9	2	2	1	1	3	1	2	0
7071	40	7	2	2	1	1	3	1	2	0
7072	56	10	1	3	1	1	3	1	2	0
7073	29	4	2	3	1	2	3	1	2	0
7074	46	8	2	2	1	1	3	1	2	0
7075	28	4	1	3	1	2	3	1	2	0
7076	22	3	1	8	1	2	2	1	2	0
7077	81	15	1	1	1	1	2	1	2	0
7078	17	2	1	7	1	2	3	1	2	0
7079	79	14	2	2	1	1	2	1	2	0
7080	26	4	2	7	1	2	3	1	2	0
7081	75	14	2	5	1	4	1	1	2	0
7082	21	3	2	7	1	2	3	1	2	0
7083	27	4	2	2	1	1	3	1	2	0
7084	19	2	1	7	1	2	3	1	2	0
7085	64	11	2	3	1	1	3	1	2	0
7086	16	2	1	7	1	2	3	1	2	0
7087	40	7	1	2	1	1	3	1	2	0
7088	60	11	1	2	1	1	3	1	2	0
7089	47	8	2	2	1	1	3	1	2	0
7090	23	3	2	7	1	2	4	1	2	0
7091	37	6	1	3	1	2	2	1	2	0
7092	62	11	2	2	1	1	3	1	2	0
7093	22	3	2	7	1	2	3	1	2	0
7094	74	13	1	2	1	1	2	1	2	0

7095	16	2	2	7	1	2	3	1	2	0
7096	15	2	2	7	1	2	3	1	2	0
7097	60	11	2	2	1	1	3	1	2	0
7098	72	13	1	3	1	1	2	1	2	0
7099	46	8	2	3	1	1	3	1	2	0
7100	18	2	2	7	1	2	3	1	2	0
7101	41	7	2	3	1	2	3	1	2	0
7102	49	8	1	2	1	1	3	1	2	0
7103	80	15	1	2	1	1	2	1	2	0
7104	66	12	2	2	1	1	3	1	2	0
7105	70	13	1	2	1	1	3	1	2	0
7106	21	3	2	7	1	2	3	1	2	0
7107	27	4	1	8	1	2	3	1	2	0
7108	13	1	2	7	1	2	2	1	2	0
7109	47	8	2	2	1	1	3	1	2	0
7110	53	9	1	1	1	1	2	1	2	0
7111	11	1	1	7	1	2	2	1	2	0
7112	43	7	2	2	1	1	3	1	2	0
7113	38	6	2	2	1	1	3	1	2	0
7114	16	2	2	7	1	2	3	1	2	0
7115	72	13	1	2	1	1	2	1	2	0
7116	65	12	2	2	1	1	3	1	2	0
7117	60	11	1	1	1	1	3	1	2	0
7118	57	10	2	2	1	1	3	1	2	0
7119	43	7	1	3	1	1	3	1	2	0
7120	61	11	1	2	1	1	2	1	2	0
7121	75	14	1	2	1	4	2	1	2	0
7122	23	3	1	7	1	2	3	1	2	0
7123	12	2	1	7	1	2	2	1	2	0
7124	28	4	2	2	1	1	3	1	2	0
7125	42	7	2	2	1	1	3	1	2	0

7126	17	2	2	7	1	2	3	1	2	0
7127	31	5	2	4	1	1	3	1	2	0
7128	14	1	2	7	1	2	3	1	2	0
7129	68	12	1	1	1	1	2	1	2	0
7130	59	10	2	2	1	1	3	1	2	0
7131	11	1	2	7	1	2	3	1	2	0
7132	17	2	2	7	1	2	3	1	2	0
7133	32	5	2	3	1	2	3	1	2	0
7134	39	6	1	2	1	1	3	1	2	0
7135	45	8	1	3	1	1	3	1	2	0
7136	84	15	2	5	1	4	2	1	2	0
7137	17	2	2	8	1	2	2	1	2	0
7138	30	5	1	1	1	2	3	1	2	0
7139	37	6	1	2	1	1	2	1	2	0
7140	34	5	2	2	1	1	3	1	2	0
7141	16	2	2	7	1	2	3	1	2	0
7142	11	1	2	7	1	2	3	1	2	0
7143	13	1	2	7	1	2	3	1	2	0
7144	20	3	1	7	1	2	3	1	2	0
7145	15	2	2	7	1	2	3	1	2	0
7146	39	6	2	2	1	1	3	1	2	0
7147	45	8	1	2	1	1	3	1	2	0
7148	24	3	2	3	1	2	3	1	2	0
7149	62	11	2	2	1	4	3	1	2	0
7150	15	2	2	7	1	2	3	1	2	0
7151	12	1	1	7	1	2	2	1	2	0
7152	23	3	2	7	1	2	3	1	2	0
7153	43	7	1	2	1	1	3	1	2	0
7154	41	7	1	1	1	1	3	1	2	0
7155	33	5	2	2	1	1	3	1	2	0
7156	25	4	2	2	1	1	3	1	2	0

7157	14	1	2	7	1	2	3	1	2	0
7158	73	13	2	2	1	4	2	1	2	0
7159	49	8	1	2	1	1	3	1	2	0
7160	20	3	2	7	1	2	3	1	2	0
7161	44	7	1	2	1	1	2	1	2	0
7162	35	6	2	2	1	1	3	1	2	0
7163	29	4	2	2	1	1	3	1	2	0
7164	18	2	2	7	1	2	3	1	2	0
7165	16	2	1	7	1	2	3	1	2	0
7166	13	1	2	7	1	2	3	1	2	0
7167	21	3	2	7	1	2	4	1	2	0
7168	46	8	1	2	1	1	3	1	2	0
7169	56	10	2	2	1	1	3	1	2	0
7170	13	1	2	7	1	2	3	1	2	0
7171	68	12	1	3	1	1	2	1	2	0
7172	24	3	1	3	1	2	3	1	2	0
7173	52	9	1	2	1	1	3	1	2	0
7174	10	1	2	7	1	2	2	1	2	0
7175	17	2	1	7	1	2	3	1	2	0
7176	14	1	1	7	1	2	3	1	2	0
7177	12	1	1	7	1	2	2	1	2	0
7178	19	2	2	7	1	2	3	1	2	0
7179	40	7	2	2	1	1	3	1	2	0
7180	22	3	1	7	1	2	3	1	2	0
7181	50	9	1	2	1	1	3	1	2	0
7182	41	7	2	3	1	1	3	1	2	0
7183	69	12	1	2	1	1	3	1	2	0
7184	76	14	1	2	1	1	2	1	2	0
7185	63	11	2	2	1	1	2	1	2	0
7186	17	2	2	7	1	2	3	1	2	0
7187	14	1	2	7	1	2	3	1	2	0

7188	11	1	2	7	1	2	2	1	2	0
7189	16	2	1	7	1	2	3	1	2	0
7190	47	8	1	3	1	1	2	1	2	0
7191	49	8	1	2	1	1	3	1	2	0
7192	87	15	2	5	3	4	1	1	2	0
7193	34	5	2	2	1	1	2	1	2	0
7194	18	2	2	7	1	2	3	1	2	0
7195	42	7	2	2	1	1	3	1	2	0
7196	37	6	2	3	1	2	3	1	2	0
7197	72	13	2	2	1	1	1	1	2	0
7198	10	1	2	7	1	2	2	1	2	0
7199	33	5	2	2	1	1	3	1	2	0
7200	20	3	2	7	1	2	3	1	2	0
7201	14	1	2	7	1	2	3	1	2	0
7202	12	1	2	7	1	2	2	1	2	0
7203	70	13	1	6	1	1	3	1	2	0
7204	62	11	2	4	1	1	3	1	2	0
7205	16	2	1	7	1	2	3	1	2	0
7206	34	5	1	2	1	2	3	1	2	0
7207	41	7	1	2	1	2	3	1	2	0
7208	45	8	1	1	1	1	3	1	2	0
7209	56	10	1	1	1	1	3	1	2	0
7210	13	1	1	7	1	2	3	1	2	0
7211	15	2	2	7	1	2	3	1	2	0
7212	71	13	2	1	1	3	2	1	2	0
7213	19	2	2	7	1	2	3	1	2	0
7214	22	3	2	3	1	2	3	1	2	0
7215	28	4	2	1	1	2	4	1	2	0
7216	63	11	2	2	1	1	2	1	2	0
7217	68	12	1	2	1	1	2	1	2	0
7218	16	2	2	7	1	2	3	1	2	0

7219	18	2	2	7	1	2	3	1	2	0
7220	26	4	1	7	1	2	4	1	2	0
7221	21	3	2	7	1	2	4	1	2	0
7222	38	6	1	2	1	1	3	1	2	0
7223	43	7	1	2	1	1	3	1	2	0
7224	58	10	1	3	1	1	2	1	2	0
7225	15	2	2	7	1	2	3	1	2	0
7226	22	3	1	8	1	2	3	1	2	0
7227	65	12	1	2	1	1	2	1	2	0
7228	52	9	2	2	1	1	3	1	2	0
7229	60	11	2	1	1	1	2	1	2	0
7230	16	2	2	7	1	2	3	1	2	0
7231	28	4	2	8	1	2	3	1	2	0
7232	44	7	1	2	1	1	2	1	2	0
7233	92	15	2	5	1	4	2	1	2	0
7234	17	2	2	7	1	2	3	1	2	0
7235	15	2	2	7	1	2	3	1	2	0
7236	23	3	2	7	1	2	4	1	2	0
7237	50	9	1	4	1	1	3	1	2	0
7238	27	4	1	3	1	1	3	6	2	0
7239	19	2	1	7	1	2	3	1	2	0
7240	14	1	2	7	1	2	2	1	2	0
7241	20	3	2	3	1	1	3	6	2	0
7242	56	10	2	2	1	1	3	4	2	0
7243	83	15	1	5	1	1	2	4	2	0
7244	16	2	2	7	1	2	3	4	2	0
7245	70	13	2	1	1	1	2	4	2	0
7246	12	1	2	7	1	2	3	4	2	0
7247	26	4	1	7	1	2	4	4	2	0
7248	17	2	2	7	1	2	3	4	2	0
7249	45	8	1	2	1	1	2	4	2	0

7250	13	1	2	7	1	2	2	4	2	0
7251	11	1	2	7	1	2	2	4	2	0
7252	30	5	2	3	1	2	3	4	2	0
7253	10	1	2	7	1	2	2	4	2	0
7254	92	15	1	5	1	4	1	4	2	0
7255	16	2	2	7	1	2	3	4	2	0
7256	15	2	1	7	1	2	3	4	2	0
7257	62	11	1	2	1	1	3	4	2	0
7258	57	10	2	2	1	1	3	4	2	0
7259	23	3	2	7	1	2	3	4	2	0
7260	18	2	2	7	1	2	3	4	2	0
7261	43	7	1	2	1	1	3	4	2	0
7262	48	8	1	2	1	1	3	4	2	0
7263	19	2	1	7	1	2	3	4	2	0
7264	29	4	1	2	1	2	3	4	2	0
7265	37	6	1	2	1	1	3	4	2	0
7266	78	14	2	5	1	1	2	4	2	0
7267	53	9	1	2	1	1	3	4	2	0
7268	16	2	2	7	1	2	3	4	2	0
7269	88	15	1	5	1	1	1	4	2	0
7270	11	1	1	7	1	2	2	4	2	0
7271	16	2	1	7	1	2	3	4	2	0
7272	18	2	2	7	1	2	3	4	2	0
7273	32	5	1	2	1	2	3	4	2	0
7274	49	8	2	2	1	1	2	4	2	0
7275	22	3	2	7	1	2	3	4	2	0
7276	35	6	2	3	1	2	3	4	2	0
7277	60	11	2	2	1	1	2	4	2	0
7278	21	3	2	7	1	2	3	4	2	0
7279	29	4	2	2	1	2	3	4	2	0
7280	70	13	2	2	1	1	2	4	2	0

7281	68	12	2	2	1	1	2	4	2	0
7282	74	13	2	2	1	1	3	4	2	0
7283	20	3	2	7	1	2	3	4	2	0
7284	42	7	2	2	1	1	3	4	2	0
7285	51	9	1	2	1	1	3	4	2	0
7286	69	12	2	1	1	1	3	4	2	0
7287	14	1	1	7	1	2	3	4	2	0
7288	19	2	1	7	1	2	3	4	2	0
7289	73	13	1	2	1	1	1	4	2	0
7290	63	11	2	2	1	1	3	4	2	0
7291	36	6	1	2	1	2	3	4	2	0
7292	27	4	2	7	1	2	4	4	2	0
7293	58	10	1	2	1	1	3	4	2	0
7294	63	11	1	2	1	1	3	4	2	0
7295	15	2	2	7	1	2	3	4	2	0
7296	17	2	2	7	1	2	3	4	2	0
7297	13	1	1	7	1	2	2	4	2	0
7298	21	3	1	7	1	2	3	4	2	0
7299	79	14	2	1	1	1	2	4	2	0
7300	12	1	2	7	1	2	2	4	2	0
7301	84	15	2	5	1	4	1	4	2	0
7302	52	9	2	2	1	1	3	4	2	0
7303	47	8	2	1	1	1	3	4	2	0
7304	56	10	1	1	1	1	3	4	2	0
7305	15	2	2	7	1	2	3	4	2	0
7306	17	2	1	7	1	2	3	4	2	0
7307	73	13	1	2	1	1	2	4	2	0
7308	69	12	2	2	1	1	2	4	2	0
7309	50	9	2	2	1	2	3	4	2	0
7310	72	13	1	1	3	1	2	4	2	0
7311	55	10	2	2	1	1	3	4	2	0

7312	16	2	2	7	1	2	3	4	2	0
7313	23	3	2	8	1	2	3	4	2	0
7314	39	6	2	2	1	1	3	4	2	0
7315	56	10	1	2	1	1	2	4	2	0
7316	61	11	2	1	1	1	3	4	2	0
7317	38	6	1	2	1	2	3	4	2	0
7318	10	1	2	7	1	2	2	4	2	0
7319	14	1	2	7	1	2	3	4	2	0
7320	19	2	1	7	1	2	3	4	2	0
7321	53	9	2	2	1	1	2	4	2	0
7322	31	5	1	3	1	2	3	4	2	0
7323	48	8	1	2	1	1	3	4	2	0
7324	20	3	2	7	1	2	3	4	2	0
7325	13	1	2	7	1	2	3	4	2	0
7326	34	5	2	3	1	2	3	4	2	0
7327	49	8	2	2	1	1	3	4	2	0
7328	12	1	2	7	1	1	3	4	2	0
7329	66	12	1	2	1	1	3	4	2	0
7330	55	10	2	2	1	1	2	4	2	0
7331	15	2	2	7	1	2	3	4	2	0
7332	36	6	2	3	1	2	2	4	2	0
7333	61	11	2	2	1	4	3	4	2	0
7334	42	7	2	7	1	2	4	4	2	0
7335	40	7	1	2	1	1	2	4	2	0
7336	38	6	1	2	1	1	2	4	2	0
7337	34	5	1	3	1	1	2	4	2	0
7338	30	5	1	3	1	2	2	4	2	0
7339	27	4	2	3	1	2	3	4	2	0
7340	22	3	2	3	1	2	3	4	2	0
7341	82	15	1	2	1	1	2	4	2	0
7342	49	8	1	3	1	1	3	4	2	0

7343	11	1	1	7	1	2	2	4	2	0
7344	19	2	2	7	1	2	3	4	2	0
7345	74	13	2	6	1	1	3	4	2	0
7346	10	1	1	7	1	2	2	4	2	0
7347	28	4	2	7	1	2	3	4	2	0
7348	34	5	2	8	1	2	3	4	2	0
7349	55	10	1	2	1	1	4	4	2	0
7350	14	1	2	7	1	2	3	4	2	0
7351	42	7	2	4	1	2	3	4	2	0
7352	18	2	1	7	1	2	3	4	2	0
7353	23	3	2	7	1	2	4	4	2	0
7354	88	15	2	6	1	4	3	4	2	0
7355	70	13	1	2	1	1	3	4	2	0
7356	15	2	2	7	1	2	3	4	2	0
7357	18	2	2	7	1	2	3	4	2	0
7358	20	3	2	7	1	2	3	4	2	0
7359	13	1	2	7	1	2	3	4	2	0
7360	15	2	2	7	1	2	3	4	2	0
7361	66	12	2	6	1	2	3	4	2	0
7362	17	2	2	7	1	2	3	4	2	0
7363	25	4	2	7	1	2	3	4	2	0
7364	57	10	1	2	1	1	3	4	2	0
7365	74	13	1	1	1	1	2	4	2	0
7366	70	13	2	2	1	4	3	4	2	0
7367	16	2	2	7	1	2	3	4	2	0
7368	46	8	1	2	1	1	3	4	2	0
7369	54	9	1	2	1	1	3	4	2	0
7370	15	2	1	7	1	2	3	4	2	0
7371	36	6	1	4	1	2	4	4	2	0
7372	24	3	2	7	1	2	3	4	2	0
7373	60	11	1	3	1	1	2	4	2	0

7374	16	2	2	7	1	2	3	4	2	0
7375	61	11	1	8	1	2	4	4	2	0
7376	13	1	1	7	1	2	3	4	2	0
7377	27	4	1	3	1	2	3	4	2	0
7378	30	5	2	3	1	2	3	4	2	0
7379	75	14	1	6	1	1	4	4	2	0
7380	67	12	2	6	1	1	4	4	2	0
7381	21	3	2	7	1	2	3	4	2	0
7382	38	6	1	2	1	1	2	4	2	0
7383	12	1	2	7	1	2	2	4	2	0
7384	73	13	1	2	1	1	2	4	2	0
7385	11	1	2	7	1	2	2	4	2	0
7386	68	12	2	2	1	1	3	4	2	0
7387	41	7	1	1	1	1	2	4	2	0
7388	16	2	1	7	1	2	3	4	2	0
7389	79	14	1	5	1	1	2	4	2	0
7390	10	1	2	7	1	2	2	4	2	0
7391	17	2	2	7	1	2	3	4	2	0
7392	88	15	2	5	1	4	2	4	2	0
7393	97	15	1	5	3	4	1	4	2	0
7394	18	2	2	7	1	2	3	4	2	0
7395	26	4	2	7	1	2	3	4	2	0
7396	70	13	1	2	1	1	2	4	2	0
7397	15	2	2	7	1	2	3	4	2	0
7398	13	1	2	7	1	2	2	4	2	0
7399	18	2	2	7	1	2	3	4	2	0
7400	25	4	1	3	1	2	2	4	2	0
7401	60	11	2	2	1	1	2	4	2	0
7402	53	9	1	2	1	1	3	4	2	0
7403	73	13	1	1	1	1	1	4	2	0
7404	13	1	2	7	1	2	2	4	2	0

7405	11	1	1	7	1	2	2	4	2	0
7406	17	2	2	7	1	2	3	4	2	0
7407	15	2	2	7	1	2	3	4	2	0
7408	21	3	2	7	1	2	4	4	2	0
7409	57	10	2	2	1	1	3	4	2	0
7410	63	11	1	2	1	1	2	4	2	0
7411	67	12	2	2	1	1	2	4	2	0
7412	16	2	2	7	1	2	3	4	2	0
7413	14	1	2	7	1	2	3	4	2	0
7414	11	1	2	7	1	2	2	4	2	0
7415	34	5	1	2	1	2	2	4	2	0
7416	51	9	2	2	1	1	2	4	2	0
7417	20	3	2	7	1	2	3	4	2	0
7418	52	9	2	1	1	1	3	4	2	0
7419	35	6	1	3	1	2	3	4	2	0
7420	64	11	2	2	1	1	3	4	2	0
7421	70	13	1	5	1	1	3	4	2	0
7422	58	10	2	2	1	1	3	4	2	0
7423	86	15	1	5	1	1	1	4	2	0
7424	66	12	2	2	1	1	2	4	2	0
7425	51	9	2	2	1	1	3	4	2	0
7426	65	12	1	2	1	1	2	4	2	0
7427	58	10	2	2	1	4	2	4	2	0
7428	58	10	2	2	1	1	3	4	2	0
7429	60	11	1	3	1	1	3	4	2	0
7430	79	14	1	1	1	1	2	4	2	0
7431	70	13	1	6	1	4	4	4	2	0
7432	82	15	2	1	1	4	1	4	2	0
7433	65	12	1	2	1	1	3	4	2	0
7434	68	12	1	2	1	1	2	4	2	0
7435	42	7	2	3	1	1	2	4	2	0

7436	59	10	2	2	1	1	2	4	2	0
7437	86	15	2	5	1	4	2	4	2	0
7438	79	14	1	2	1	1	3	4	2	0
7439	60	11	1	4	1	1	3	6	2	0
7440	47	8	1	1	1	1	2	2	2	0
7441	72	13	1	1	1	1	3	2	2	0
7442	57	10	2	2	1	1	3	6	2	0
7443	95	15	2	5	1	4	1	4	2	0
7444	16	2	1	7	1	2	3	4	2	0
7445	29	4	2	7	1	2	3	4	2	0
7446	20	3	2	3	1	1	3	4	2	0
7447	47	8	1	2	1	1	2	4	2	0
7448	76	14	1	2	1	1	2	4	2	0
7449	53	9	2	2	1	1	3	4	2	0
7450	67	12	1	1	1	1	1	4	2	0
7451	61	11	1	2	1	1	3	4	2	0
7452	35	6	2	2	1	2	3	4	2	0
7453	76	14	1	6	1	1	2	4	2	0
7454	64	11	2	2	1	1	3	4	2	0
7455	11	1	2	7	1	2	2	4	2	0
7456	77	14	2	2	1	4	2	4	2	0
7457	45	8	1	3	1	1	2	4	2	0
7458	36	6	2	2	1	1	3	4	2	0
7459	56	10	1	1	1	1	3	4	2	0
7460	69	12	1	2	1	1	3	4	2	0
7461	40	7	2	2	1	1	3	4	2	0
7462	46	8	1	2	1	1	3	4	2	0
7463	23	3	2	7	1	2	3	4	2	0
7464	29	4	2	2	1	1	3	4	2	0
7465	63	11	1	5	1	1	3	4	2	0
7466	16	2	2	7	1	2	3	4	2	0

7467	19	2	2	7	1	2	3	4	2	0
7468	28	4	1	3	1	2	2	4	2	0
7469	17	2	2	7	1	2	3	4	2	0
7470	12	1	2	7	1	2	2	4	2	0
7471	22	3	2	7	1	2	3	4	2	0
7472	26	4	2	7	1	2	3	4	2	0
7473	53	9	2	2	1	1	2	4	2	0
7474	68	12	1	2	1	1	2	4	2	0
7475	14	1	2	7	1	2	2	4	2	0
7476	17	2	1	7	1	2	3	4	2	0
7477	19	2	1	7	1	2	3	4	2	0
7478	21	3	2	7	1	2	3	4	2	0
7479	73	13	1	2	1	1	2	4	2	0
7480	10	1	2	7	1	2	2	4	2	0
7481	60	11	2	2	1	1	1	4	2	0
7482	53	9	1	2	1	1	3	4	2	0
7483	81	15	1	5	1	1	1	4	2	0
7484	24	3	2	7	1	2	3	4	2	0
7485	45	8	2	3	1	1	3	4	2	0
7486	16	2	1	7	1	2	3	4	2	0
7487	11	1	2	7	1	2	2	4	2	0
7488	55	10	2	2	1	1	2	4	2	0
7489	15	2	2	7	1	2	3	4	2	0
7490	20	3	2	7	1	2	3	4	2	0
7491	64	11	1	3	1	1	3	4	2	0
7492	85	15	2	5	1	4	1	4	2	0
7493	14	1	2	7	1	2	2	4	2	0
7494	13	1	2	7	1	2	2	4	2	0
7495	18	2	2	7	1	2	3	4	2	0
7496	72	13	1	2	1	1	2	4	2	0
7497	65	12	2	2	1	1	3	4	2	0

7498	19	2	1	3	1	2	2	4	2	0
7499	15	2	2	7	1	2	3	4	2	0
7500	12	1	2	7	1	2	2	4	2	0
7501	33	5	1	3	1	1	2	4	2	0
7502	21	3	2	7	1	2	3	4	2	0
7503	42	7	1	2	1	1	3	4	2	0
7504	68	12	2	1	1	4	2	4	2	0
7505	46	8	1	2	1	1	3	2	2	0
7506	56	10	2	2	1	1	2	2	2	0
7507	18	2	2	7	1	2	3	2	2	0
7508	11	1	2	7	1	2	2	2	2	0
7509	13	1	2	7	1	2	2	2	2	0
7510	15	2	1	7	1	2	3	4	2	0
7511	10	1	2	7	1	2	2	4	2	0
7512	31	5	2	2	1	1	3	4	2	0
7513	18	2	2	7	1	2	3	4	2	0
7514	69	12	1	2	1	1	3	2	2	0
7515	30	5	1	3	1	2	3	2	2	0
7516	56	10	2	2	1	1	3	2	2	0
7517	12	1	1	7	1	2	2	2	2	0
7518	30	5	2	2	1	1	3	2	2	0
7519	45	8	1	2	1	1	3	2	2	0
7520	29	4	2	7	1	2	4	2	2	0
7521	17	2	2	7	1	2	3	2	2	0
7522	22	3	2	7	1	2	3	2	2	0
7523	62	11	1	3	1	1	2	2	2	0
7524	19	2	1	7	1	2	3	2	2	0
7525	11	1	1	7	1	2	2	2	2	0
7526	28	4	1	8	1	2	3	2	2	0
7527	34	5	1	3	1	2	3	2	2	0
7528	27	4	2	7	1	2	4	2	2	0

7529	80	15	1	5	1	1	1	2	2	0
7530	68	12	2	2	1	1	2	2	2	0
7531	15	2	2	7	1	2	3	2	2	0
7532	16	2	2	7	1	2	3	2	2	0
7533	13	1	2	7	1	2	3	2	2	0
7534	55	10	2	2	1	1	2	2	2	0
7535	59	10	1	2	1	1	2	6	2	0
7536	46	8	2	8	1	1	3	6	2	0
7537	12	1	1	7	1	2	2	6	2	0
7538	14	1	1	7	1	2	2	6	2	0
7539	25	4	1	3	1	2	2	4	2	0
7540	62	11	1	2	1	1	2	4	2	0
7541	18	2	2	7	1	2	3	2	2	0
7542	56	10	2	2	1	1	3	2	2	0
7543	20	3	2	7	1	2	3	2	2	0
7544	16	2	2	7	1	2	3	2	2	0
7545	11	1	2	7	1	2	2	4	2	0
7546	32	5	1	3	1	2	3	2	2	0
7547	10	1	2	7	1	2	2	2	2	0
7548	18	2	1	7	1	2	3	2	2	0
7549	16	2	2	7	1	2	3	2	2	0
7550	86	15	1	5	1	1	2	2	2	0
7551	20	3	2	7	1	2	3	2	2	0
7552	15	2	2	7	1	2	3	2	2	0
7553	76	14	2	2	1	1	2	2	2	0
7554	18	2	2	7	1	2	3	6	2	0
7555	15	2	1	7	1	2	3	6	2	0
7556	11	1	2	7	1	2	2	6	2	0
7557	57	10	1	2	1	1	3	2	2	0
7558	17	2	1	7	1	2	3	6	2	0
7559	14	1	1	7	1	2	3	6	2	0

7560	23	3	2	3	1	2	3	6	2	0
7561	13	1	2	7	1	2	2	2	2	0
7562	17	2	2	8	1	2	2	2	2	0
7563	41	7	1	1	1	1	2	2	2	0
7564	70	13	1	1	1	1	2	2	2	0
7565	23	3	2	7	1	2	3	2	2	0
7566	12	1	2	7	1	2	2	2	2	0
7567	36	6	2	2	1	1	3	2	2	0
7568	63	11	2	2	1	1	2	2	2	0
7569	43	7	1	3	1	4	3	2	2	0
7570	17	2	1	7	1	2	3	2	2	0
7571	69	12	1	1	1	1	2	2	2	0
7572	50	9	2	2	1	1	3	2	2	0
7573	16	2	2	7	1	2	3	2	2	0
7574	13	1	2	7	1	2	3	2	2	0
7575	20	3	2	7	1	2	4	2	2	0
7576	27	4	1	3	1	2	3	2	2	0
7577	15	2	2	7	1	2	3	2	2	0
7578	19	2	1	7	1	2	3	2	2	0
7579	74	13	1	2	1	1	2	2	2	0
7580	68	12	2	6	1	1	3	2	2	0
7581	14	1	1	7	1	2	3	2	2	0
7582	15	2	1	7	1	2	3	2	2	0
7583	56	10	2	2	1	1	2	2	2	0
7584	17	2	2	7	1	2	3	2	2	0
7585	13	1	1	7	1	2	2	2	2	0
7586	15	2	2	7	1	2	3	2	2	0
7587	23	3	1	3	1	2	3	2	2	0
7588	18	2	2	7	1	2	3	2	2	0
7589	23	3	2	7	1	2	3	2	2	0
7590	62	11	1	2	1	1	3	2	2	0

7591	17	2	2	7	1	2	3	2	2	0
7592	25	4	2	3	1	1	2	6	2	0
7593	59	10	2	1	1	1	2	2	2	0
7594	35	6	1	2	1	1	2	6	2	0
7595	12	1	2	7	1	2	2	2	2	0
7596	71	13	1	1	1	1	2	2	2	0
7597	14	1	2	7	1	2	3	2	2	0
7598	11	1	2	7	1	2	2	2	2	0
7599	16	2	2	7	1	2	3	2	2	0
7600	23	3	2	7	1	2	3	2	2	0
7601	67	12	2	2	1	1	2	2	2	0
7602	32	5	2	2	1	1	2	2	2	0
7603	56	10	2	1	1	1	3	2	2	0
7604	10	1	2	7	1	2	2	2	2	0
7605	15	2	2	7	1	2	3	2	2	0
7606	24	3	1	7	1	2	4	2	2	0
7607	40	7	1	3	1	1	3	2	2	0
7608	62	11	2	5	1	1	3	2	2	0
7609	38	6	1	2	1	2	3	2	2	0
7610	71	13	2	1	3	4	1	2	2	0
7611	17	2	2	7	1	2	3	2	2	0
7612	14	1	2	7	1	2	3	2	2	0
7613	22	3	1	7	1	2	3	2	2	0
7614	45	8	2	2	1	2	3	2	2	0
7615	48	8	1	2	1	1	3	2	2	0
7616	36	6	2	2	1	1	3	2	2	0
7617	10	1	1	7	1	2	2	2	2	0
7618	51	9	1	2	1	1	2	2	2	0
7619	13	1	2	7	1	2	2	2	2	0
7620	17	2	1	7	1	2	3	2	2	0
7621	29	4	1	2	1	2	3	2	2	0

7622	27	4	1	1	1	2	3	2	2	0
7623	28	4	1	2	1	2	3	2	2	0
7624	10	1	2	7	1	2	2	2	2	0
7625	60	11	2	2	1	1	2	2	2	0
7626	45	8	2	2	1	1	3	2	2	0
7627	11	1	2	7	1	2	2	2	2	0
7628	28	4	1	7	1	2	3	2	2	0
7629	19	2	2	7	1	2	3	2	2	0
7630	35	6	2	3	1	1	3	2	2	0
7631	20	3	2	7	1	2	3	2	2	0
7632	58	10	1	1	1	1	3	2	2	0
7633	37	6	2	2	1	1	3	2	2	0
7634	17	2	1	7	1	2	3	2	2	0
7635	24	3	1	7	1	2	3	2	2	0
7636	55	10	1	3	1	1	3	2	2	0
7637	38	6	2	2	1	1	3	2	2	0
7638	14	1	2	7	1	2	3	2	2	0
7639	19	2	2	7	1	2	3	2	2	0
7640	15	2	2	7	1	2	3	2	2	0
7641	53	9	2	2	1	1	3	2	2	0
7642	76	14	2	2	1	1	2	2	2	0
7643	26	4	2	7	1	2	4	2	2	0
7644	16	2	2	7	1	2	3	2	2	0
7645	12	1	1	7	1	2	2	2	2	0
7646	57	10	2	2	1	1	2	2	2	0
7647	13	1	2	7	1	2	3	2	2	0
7648	52	9	2	2	1	1	3	2	2	0
7649	48	8	2	2	1	1	3	2	2	0
7650	13	1	1	7	1	2	2	2	2	0
7651	19	2	1	7	1	2	3	2	2	0
7652	71	13	1	1	1	1	2	2	2	0

7653	28	4	1	3	1	1	3	2	2	0
7654	12	1	1	7	1	2	2	2	2	0
7655	17	2	2	7	1	2	3	2	2	0
7656	65	12	2	2	1	1	2	2	2	0
7657	18	2	1	8	1	2	2	2	2	0
7658	60	11	2	3	1	1	2	2	2	0
7659	50	9	1	2	1	1	3	2	2	0
7660	20	3	1	7	1	2	3	2	2	0
7661	59	10	2	4	1	1	4	2	2	0
7662	61	11	1	2	1	1	2	2	2	0
7663	35	6	2	3	1	1	3	2	2	0
7664	20	3	2	7	1	2	3	2	2	0
7665	11	1	1	7	1	2	2	2	2	0
7666	44	7	1	3	1	2	2	2	2	0
7667	80	15	2	1	3	4	1	2	2	0
7668	20	3	2	3	1	2	3	2	2	0
7669	55	10	2	2	1	1	3	2	2	0
7670	32	5	1	2	1	2	4	2	2	0
7671	13	1	2	7	1	2	3	2	2	0
7672	21	3	2	7	1	2	3	2	2	0
7673	28	4	1	4	1	2	4	2	2	0
7674	19	2	2	7	1	2	3	2	2	0
7675	30	5	2	3	1	2	3	2	2	0
7676	89	15	2	5	1	4	1	2	2	0
7677	27	4	1	2	1	2	4	2	2	0
7678	30	5	2	2	1	1	3	6	2	0
7679	43	7	1	2	1	1	3	6	2	0
7680	15	2	1	7	1	2	3	2	2	0
7681	12	1	1	7	1	2	2	2	2	0
7682	50	9	1	2	1	1	2	2	2	0
7683	24	3	2	2	1	2	3	2	2	0

7684	11	1	2	7	1	2	2	2	2	0
7685	21	3	2	7	1	2	4	2	2	0
7686	17	2	2	7	1	2	3	2	2	0
7687	47	8	2	4	1	1	3	5	2	0
7688	19	2	2	7	1	2	3	5	2	0
7689	39	6	1	2	1	1	3	5	2	0
7690	60	11	1	4	1	1	3	5	2	0
7691	56	10	2	2	1	1	3	5	2	0
7692	28	4	2	7	1	2	4	5	2	0
7693	37	6	1	3	1	1	3	5	2	0
7694	26	4	2	3	1	1	3	5	2	0
7695	24	3	2	7	1	2	3	5	2	0
7696	18	2	2	7	1	2	3	5	2	0
7697	98	15	2	5	1	4	1	5	2	0
7698	26	4	2	4	1	2	4	5	2	0
7699	46	8	2	2	1	1	3	5	2	0
7700	17	2	2	7	1	2	3	5	2	0
7701	15	2	2	7	1	2	3	5	2	0
7702	15	2	2	7	1	2	3	5	2	0
7703	19	2	2	7	1	2	3	5	2	0
7704	13	1	2	7	1	2	3	5	2	0
7705	15	2	1	7	1	2	3	5	2	0
7706	19	2	2	7	1	2	3	5	2	0
7707	18	2	2	7	1	2	3	5	2	0
7708	14	1	2	7	1	2	3	5	2	0
7709	32	5	2	3	1	2	3	5	2	0
7710	55	10	1	2	1	1	2	5	2	0
7711	60	11	1	3	1	1	2	5	2	0
7712	70	13	1	6	1	1	2	5	2	0
7713	10	1	1	7	1	2	2	5	2	0
7714	19	2	1	7	1	2	3	5	2	0

7715	33	5	1	2	1	1	2	5	2	0
7716	16	2	2	7	1	2	3	5	2	0
7717	24	3	1	7	1	2	3	5	2	0
7718	41	7	1	2	1	1	2	5	2	0
7719	69	12	1	1	1	1	3	5	2	0
7720	52	9	2	2	1	1	3	5	2	0
7721	16	2	1	7	1	2	3	5	2	0
7722	75	14	2	5	1	4	2	5	2	0
7723	17	2	2	7	1	2	3	5	2	0
7724	48	8	1	2	1	1	3	5	2	0
7725	27	4	2	3	1	2	3	5	2	0
7726	95	15	1	5	1	4	2	5	2	0
7727	18	2	2	7	1	2	3	5	2	0
7728	15	2	1	7	1	2	3	5	2	0
7729	19	2	2	7	1	2	3	5	2	0
7730	25	4	2	7	1	2	4	5	2	0
7731	60	11	1	2	1	1	3	5	2	0
7732	60	11	2	2	1	1	3	5	2	0
7733	13	1	2	7	1	2	3	5	2	0
7734	15	2	2	7	1	2	3	5	2	0
7735	17	2	1	7	1	2	3	5	2	0
7736	47	8	1	3	1	1	3	5	2	0
7737	30	5	2	2	1	1	3	5	2	0
7738	57	10	1	1	1	1	3	5	2	0
7739	54	9	2	3	1	1	3	5	2	0
7740	12	1	2	7	1	2	2	5	2	0
7741	16	2	1	8	1	2	2	5	2	0
7742	70	13	1	2	3	1	2	5	2	0
7743	65	12	2	2	1	1	3	5	2	0
7744	11	1	2	7	1	2	2	5	2	0
7745	13	1	1	7	1	2	3	5	2	0

7746	50	9	2	2	1	1	3	5	2	0
7747	10	1	1	7	1	2	2	5	2	0
7748	22	3	2	7	1	2	3	5	2	0
7749	58	10	1	2	1	1	3	5	2	0
7750	16	2	1	7	1	2	3	5	2	0
7751	88	15	2	5	1	4	1	3	2	0
7752	59	10	1	2	1	1	3	3	2	0
7753	64	11	1	2	1	1	3	3	2	0
7754	68	12	1	2	1	1	2	3	2	0
7755	56	10	2	2	1	1	3	3	2	0
7756	21	3	2	7	1	2	4	3	2	0
7757	25	4	1	7	1	2	4	3	2	0
7758	17	2	2	7	1	2	3	3	2	0
7759	13	1	2	7	1	2	2	3	2	0
7760	15	2	1	7	1	2	3	3	2	0
7761	18	2	2	7	1	2	3	3	2	0
7762	27	4	2	7	1	2	4	3	2	0
7763	66	12	1	2	1	1	3	3	2	0
7764	58	10	2	3	1	1	3	3	2	0
7765	60	11	1	2	1	1	3	3	2	0
7766	10	1	2	7	1	2	2	3	2	0
7767	14	1	2	7	1	2	3	3	2	0
7768	13	1	2	7	1	2	2	3	2	0
7769	17	2	2	7	1	2	3	3	2	0
7770	37	6	2	3	1	2	3	3	2	0
7771	22	3	2	8	1	2	3	3	2	0
7772	50	9	2	2	1	1	3	3	2	0
7773	45	8	2	2	1	2	3	3	2	0
7774	13	1	2	7	1	2	3	3	2	0
7775	15	2	1	7	1	2	3	3	2	0
7776	17	2	1	7	1	2	3	3	2	0

7777	11	1	1	7	1	2	2	3	2	0
7778	13	1	2	7	1	2	2	3	2	0
7779	12	1	2	7	1	2	2	3	2	0
7780	15	2	2	7	1	2	3	3	2	0
7781	47	8	2	2	1	5	3	3	2	0
7782	52	9	2	2	1	1	3	3	2	0
7783	62	11	2	3	1	1	2	3	2	0
7784	16	2	2	7	1	2	3	3	2	0
7785	13	1	2	7	1	2	3	3	2	0
7786	11	1	2	7	1	2	3	3	2	0
7787	15	2	1	7	1	2	3	3	2	0
7788	66	12	1	2	1	1	3	3	2	0
7789	13	1	2	7	1	2	2	3	2	0
7790	53	9	2	2	1	1	2	3	2	0
7791	59	10	2	2	1	1	2	3	2	0
7792	91	15	2	5	1	4	2	3	2	0
7793	65	12	1	6	1	1	4	3	2	0
7794	58	10	2	4	1	1	4	3	2	0
7795	13	1	2	7	1	2	3	3	2	0
7796	16	2	2	7	1	2	3	3	2	0
7797	21	3	2	7	1	2	4	3	2	0
7798	17	2	2	7	1	2	3	3	2	0
7799	12	1	1	7	1	2	2	3	2	0
7800	43	7	1	5	1	2	1	3	2	0
7801	47	8	1	2	1	1	3	6	2	0
7802	14	1	1	7	1	2	3	6	2	0
7803	16	2	2	7	1	2	3	3	2	0
7804	12	1	1	7	1	2	2	3	2	0
7805	17	2	2	7	1	2	3	3	2	0
7806	38	6	2	2	1	1	3	6	2	0
7807	25	4	1	3	1	2	2	5	2	0

7808	22	3	1	7	1	2	3	5	2	0
7809	11	1	2	7	1	2	2	5	2	0
7810	70	13	2	2	1	4	2	5	2	0
7811	59	10	1	2	1	1	2	5	2	0
7812	13	1	2	7	1	2	3	5	2	0
7813	15	2	2	7	1	2	3	5	2	0
7814	24	3	2	7	1	2	3	5	2	0
7815	35	6	1	3	1	2	3	5	2	0
7816	42	7	2	2	1	2	3	5	2	0
7817	19	2	1	7	1	2	3	5	2	0
7818	10	1	1	7	1	2	2	5	2	0
7819	61	11	2	2	1	1	3	5	2	0
7820	21	3	2	7	1	2	3	5	2	0
7821	23	3	1	7	1	2	3	5	2	0
7822	66	12	1	2	1	1	2	5	2	0
7823	11	1	1	7	1	2	2	5	2	0
7824	12	1	1	7	1	2	2	5	2	0
7825	15	2	2	7	1	2	3	5	2	0
7826	33	5	1	3	1	1	2	5	2	0
7827	45	8	2	2	1	1	3	5	2	0
7828	52	9	1	2	1	1	2	5	2	0
7829	13	1	2	7	1	2	2	5	2	0
7830	10	1	2	7	1	2	2	5	2	0
7831	20	3	1	7	1	2	3	5	2	0
7832	15	2	1	7	1	2	3	5	2	0
7833	31	5	2	3	1	1	3	5	2	0
7834	55	10	1	2	1	1	3	5	2	0
7835	13	1	2	7	1	2	3	5	2	0
7836	18	2	1	7	1	2	3	5	2	0
7837	17	2	2	7	1	2	3	5	2	0
7838	19	2	2	8	1	2	3	5	2	0

7839	16	2	1	7	1	2	3	5	2	0
7840	40	7	1	3	1	1	3	5	2	0
7841	18	2	1	7	1	2	3	5	2	0
7842	22	3	1	8	1	2	3	5	2	0
7843	53	9	2	2	1	1	3	5	2	0
7844	37	6	1	2	1	2	3	5	2	0
7845	12	1	2	7	1	2	2	5	2	0
7846	30	5	2	3	1	1	3	5	2	0
7847	18	2	2	7	1	2	3	5	2	0
7848	27	4	2	7	1	2	4	5	2	0
7849	33	5	1	2	1	1	3	5	2	0
7850	37	6	2	2	1	1	3	5	2	0
7851	16	2	2	7	1	2	3	5	2	0
7852	12	1	1	7	1	2	2	5	2	0
7853	14	1	1	7	1	2	3	5	2	0
7854	50	9	1	2	1	1	2	5	2	0
7855	48	8	2	2	1	1	3	5	2	0
7856	29	4	2	3	1	1	3	5	2	0
7857	78	14	1	2	1	1	2	5	2	0
7858	60	11	2	2	1	1	3	5	2	0
7859	26	4	2	7	1	2	4	5	2	0
7860	24	3	1	7	1	2	3	5	2	0
7861	16	2	2	7	1	2	3	5	2	0
7862	15	2	1	7	1	2	3	5	2	0
7863	68	12	2	2	1	4	3	5	2	0
7864	13	1	1	7	1	2	3	5	2	0
7865	72	13	1	2	1	1	2	5	2	0
7866	20	3	1	7	1	2	3	5	2	0
7867	35	6	2	2	1	1	3	5	2	0
7868	63	11	2	2	1	1	3	5	2	0
7869	21	3	2	7	1	2	3	5	2	0

7870	10	1	2	7	1	2	2	5	2	0
7871	19	2	2	7	1	2	3	5	2	0
7872	12	1	1	7	1	2	2	5	2	0
7873	38	6	2	1	1	1	3	5	2	0
7874	11	1	2	7	1	2	2	5	2	0
7875	15	2	1	7	1	2	3	5	2	0
7876	49	8	1	2	1	1	3	6	2	0
7877	37	6	2	2	1	1	3	6	2	0
7878	89	15	2	5	1	4	2	3	2	0
7879	19	2	1	8	1	2	2	3	2	0
7880	10	1	2	7	1	2	2	3	2	0
7881	52	9	1	2	1	1	3	3	2	0
7882	23	3	2	7	1	2	3	3	2	0
7883	45	8	2	2	1	1	3	3	2	0
7884	61	11	1	1	1	1	3	3	2	0
7885	50	9	2	2	1	1	3	3	2	0
7886	32	5	1	2	1	2	3	3	2	0
7887	20	3	2	7	1	2	3	3	2	0
7888	18	2	2	7	1	2	3	3	2	0
7889	36	6	1	3	1	1	3	3	2	0
7890	17	2	2	7	1	2	3	3	2	0
7891	29	4	1	3	1	2	3	3	2	0
7892	52	9	2	2	1	1	3	3	2	0
7893	68	12	1	2	1	1	2	3	2	0
7894	30	5	2	3	1	2	3	3	2	0
7895	15	2	2	7	1	2	3	3	2	0
7896	34	5	2	3	1	1	3	3	2	0
7897	12	1	1	7	1	2	2	3	2	0
7898	28	4	2	7	1	2	3	3	2	0
7899	49	8	1	2	1	1	3	3	2	0
7900	27	4	2	7	1	2	3	3	2	0

7901	65	12	2	1	1	1	2	3	2	0
7902	83	15	1	5	1	1	1	3	2	0
7903	27	4	2	3	1	1	2	3	2	0
7904	13	1	2	7	1	2	3	3	2	0
7905	10	1	1	7	1	2	2	3	2	0
7906	18	2	2	7	1	2	3	3	2	0
7907	12	1	1	7	1	2	2	3	2	0
7908	13	1	2	7	1	2	2	3	2	0
7909	21	3	1	7	1	2	3	3	2	0
7910	35	6	2	4	1	1	3	3	2	0
7911	52	9	1	2	1	1	2	3	2	0
7912	19	2	2	7	1	2	3	3	2	0
7913	13	1	2	7	1	2	2	3	2	0
7914	43	7	2	2	1	1	2	3	2	0
7915	40	7	2	3	1	2	3	3	2	0
7916	45	8	1	2	1	1	2	3	2	0
7917	11	1	1	7	1	2	2	3	2	0
7918	18	2	2	7	1	2	3	3	2	0
7919	17	2	1	7	1	2	3	3	2	0
7920	67	12	1	1	1	1	3	3	2	0
7921	37	6	1	2	1	2	3	3	2	0
7922	33	5	1	3	1	2	3	3	2	0
7923	60	11	2	2	1	1	2	5	2	0
7924	63	11	1	1	1	1	3	5	2	0
7925	20	3	1	3	1	2	2	5	2	0
7926	22	3	2	3	1	2	2	5	2	0
7927	29	4	2	2	1	2	3	5	2	0
7928	50	9	2	2	1	1	2	5	2	0
7929	79	14	1	2	1	4	2	5	2	0
7930	13	1	2	7	1	2	2	5	2	0
7931	14	1	2	7	1	2	3	5	2	0

7932	10	1	1	7	1	2	2	5	2	0
7933	33	5	2	2	1	1	3	5	2	0
7934	66	12	2	6	1	1	3	5	2	0
7935	27	4	1	7	1	2	4	5	2	0
7936	11	1	2	7	1	2	2	5	2	0
7937	31	5	2	3	1	1	3	5	2	0
7938	26	4	1	8	1	2	3	5	2	0
7939	68	12	1	1	1	1	3	5	2	0
7940	57	10	2	1	1	1	3	3	2	0
7941	77	14	1	2	1	4	2	3	2	0
7942	25	4	1	2	1	2	3	3	2	0
7943	50	9	1	3	1	1	3	3	2	0
7944	30	5	1	3	1	1	2	3	2	0
7945	49	8	2	2	1	2	3	3	2	0
7946	22	3	2	7	1	2	3	3	2	0
7947	16	1	1	7	1	2	3	3	2	0
7948	11	2	2	7	1	2	2	3	2	0
7949	71	13	1	2	1	1	3	3	2	0
7950	26	4	1	3	1	2	2	3	2	0
7951	23	3	1	3	1	2	3	3	2	0
7952	44	7	2	2	1	1	3	3	2	0
7953	35	6	2	2	1	2	3	3	2	0
7954	40	7	1	2	1	1	3	3	2	0
7955	18	2	1	7	1	2	3	3	2	0
7956	58	10	2	2	1	4	3	3	2	0
7957	24	3	1	7	1	2	3	3	2	0
7958	19	2	1	7	1	2	3	3	2	0
7959	16	2	2	7	1	2	3	3	2	0
7960	13	1	2	7	1	2	3	3	2	0
7961	91	15	2	5	1	4	1	3	2	0
7962	62	11	1	2	1	1	3	3	2	0

7963	66	12	1	2	1	1	3	3	2	0
7964	53	9	2	2	1	1	3	3	2	0
7965	18	2	2	7	1	2	3	3	2	0
7966	70	13	1	2	1	1	2	3	2	0
7967	59	10	2	2	1	1	3	3	2	0
7968	39	6	2	4	1	1	4	3	2	0
7969	17	2	1	7	1	2	3	3	2	0
7970	42	7	1	2	1	1	3	3	2	0
7971	68	12	1	2	1	1	2	3	2	0
7972	56	10	2	2	1	1	3	3	2	0
7973	29	4	2	8	1	1	3	3	2	0
7974	46	8	2	2	1	1	3	3	2	0
7975	21	3	2	7	1	2	3	3	2	0
7976	14	1	2	7	1	2	3	3	2	0
7977	33	5	1	3	1	1	3	3	2	0
7978	28	4	2	2	1	1	2	3	2	0
7979	10	1	2	7	1	2	2	3	2	0
7980	15	2	2	3	1	2	3	3	2	0
7981	52	9	2	3	1	1	3	3	2	0
7982	19	2	1	7	1	2	3	3	2	0
7983	14	1	1	7	1	2	3	3	2	0
7984	65	12	1	4	1	1	3	3	2	0
7985	13	1	2	7	1	2	3	3	2	0
7986	12	1	1	7	1	2	2	4	2	0
7987	18	2	1	8	1	2	2	4	2	0
7988	47	8	2	2	1	1	3	4	2	0
7989	11	1	1	7	1	2	2	4	2	0
7990	28	4	2	2	1	1	3	4	2	0
7991	15	1	2	7	1	2	3	4	2	0
7992	30	5	1	2	1	1	2	4	2	0
7993	26	3	1	3	1	1	3	4	2	0

7994	10	1	1	7	1	2	2	4	2	0
7995	60	11	2	2	1	1	3	4	2	0
7996	66	12	1	2	1	1	3	4	2	0
7997	11	1	2	7	1	2	3	4	2	0
7998	32	5	2	2	1	1	3	4	2	0
7999	45	8	2	2	1	1	3	4	2	0
8000	67	12	1	2	1	1	3	4	2	0
8001	10	1	1	7	1	2	2	4	2	0
8002	13	1	2	7	1	2	3	4	2	0
8003	12	1	2	7	1	2	3	4	2	0
8004	14	1	1	7	1	2	3	4	2	0
8005	72	13	1	2	3	1	2	4	2	0
8006	38	6	2	3	1	1	2	4	2	0
8007	70	13	2	2	1	1	2	4	2	0
8008	41	7	2	2	1	2	3	4	2	0
8009	64	11	2	2	1	1	3	4	2	0
8010	37	6	1	3	1	1	3	4	2	0
8011	15	2	2	7	1	2	3	4	2	0
8012	19	2	1	7	1	2	3	4	2	0
8013	24	3	1	8	1	2	3	4	2	0
8014	53	9	2	2	1	1	3	4	2	0
8015	60	11	2	1	1	1	2	4	2	0
8016	46	8	1	2	1	1	3	6	2	0
8017	40	7	2	2	1	1	3	6	2	0
8018	12	1	2	7	1	2	2	4	2	0
8019	18	2	1	7	1	2	3	4	2	0
8020	19	2	2	7	1	2	3	4	2	0
8021	26	4	1	3	1	2	3	4	2	0
8022	33	5	1	1	1	2	3	4	2	0
8023	10	1	1	7	1	2	2	4	2	0
8024	16	2	2	7	1	2	3	4	2	0

8025	18	2	2	7	1	2	3	4	2	0
8026	14	1	1	7	1	2	2	4	2	0
8027	55	10	1	2	1	1	3	4	2	0
8028	46	8	2	2	1	1	3	4	2	0
8029	17	2	1	7	1	2	3	4	2	0
8030	20	3	1	7	1	2	3	4	2	0
8031	14	1	2	7	1	2	3	4	2	0
8032	21	3	2	7	1	2	4	4	2	0
8033	64	11	2	2	1	4	2	4	2	0
8034	40	7	1	2	1	1	3	4	2	0
8035	13	1	2	7	1	2	2	4	2	0
8036	11	1	2	7	1	2	2	4	2	0
8037	35	6	2	2	1	1	3	4	2	0
8038	67	12	1	2	1	1	2	4	2	0
8039	21	3	2	3	1	2	3	4	2	0
8040	19	2	2	7	1	2	3	4	2	0
8041	70	13	1	2	1	1	2	4	2	0
8042	61	11	2	2	1	1	3	4	2	0
8043	17	2	2	7	1	2	3	4	2	0
8044	47	8	1	3	1	1	3	4	2	0
8045	39	6	2	2	1	1	3	4	2	0
8046	16	2	2	7	1	2	3	4	2	0
8047	52	9	1	1	1	1	3	4	2	0
8048	19	2	1	7	1	2	3	4	2	0
8049	11	1	2	7	1	2	2	4	2	0
8050	10	1	1	7	1	2	3	4	2	0
8051	20	3	1	8	1	2	2	4	2	0
8052	47	8	2	2	1	1	3	4	2	0
8053	65	12	1	1	1	1	2	2	2	0
8054	59	10	2	1	1	1	2	2	2	0
8055	13	1	2	7	1	2	2	2	2	0

8056	11	1	2	7	1	2	2	2	2	0
8057	16	2	1	7	1	2	3	2	2	0
8058	14	1	1	7	1	2	3	2	2	0
8059	34	5	1	3	1	1	2	2	2	0
8060	29	4	2	2	1	1	3	2	2	0
8061	44	7	1	2	1	1	3	2	2	0
8062	35	6	2	2	1	1	3	2	2	0
8063	50	9	1	2	1	1	2	3	2	0
8064	18	2	1	7	1	2	3	3	2	0
8065	61	11	1	2	1	1	3	3	2	0
8066	48	8	2	2	1	1	3	3	2	0
8067	17	2	2	7	1	2	3	3	2	0
8068	53	9	2	2	1	1	2	3	2	0
8069	21	3	2	7	1	2	3	3	2	0
8070	30	5	1	3	1	1	3	3	2	0
8071	27	4	2	3	1	1	3	3	2	0
8072	74	13	1	2	1	4	2	3	2	0
8073	38	6	2	2	1	2	3	3	2	0
8074	15	2	1	7	1	2	3	3	2	0
8075	14	1	1	7	1	2	3	3	2	0
8076	27	4	1	8	1	2	3	3	2	0
8077	65	12	2	2	1	1	3	3	2	0
8078	43	7	2	2	1	1	3	3	2	0
8079	15	2	2	7	1	2	3	3	2	0
8080	20	3	1	3	1	2	2	3	2	0
8081	53	9	2	2	1	4	3	4	2	0
8082	30	5	1	2	1	2	3	4	2	0
8083	28	4	2	7	1	2	4	4	2	0
8084	25	4	2	7	1	2	4	4	2	0
8085	22	3	1	7	1	2	3	4	2	0
8086	19	2	1	7	1	2	3	4	2	0

8087	67	12	1	3	1	1	2	4	2	0
8088	58	10	2	2	1	1	2	4	2	0
8089	28	4	1	2	1	2	3	4	2	0
8090	36	6	2	2	1	2	3	4	2	0
8091	30	5	2	3	1	2	3	4	2	0
8092	22	3	1	3	1	2	3	4	2	0
8093	47	8	1	2	1	1	2	6	2	0
8094	34	5	2	2	1	1	2	6	2	0
8095	10	1	1	7	1	2	2	6	2	0
8096	69	12	2	2	1	4	3	3	2	0
8097	42	7	1	2	1	1	3	3	2	0
8098	19	2	1	7	1	2	3	3	2	0
8099	17	2	2	7	1	2	3	3	2	0
8100	26	4	1	3	1	2	3	3	2	0
8101	38	6	2	2	1	1	3	3	2	0
8102	33	5	1	2	1	2	3	3	2	0
8103	36	6	1	2	1	1	3	3	2	0
8104	25	4	2	2	1	1	3	3	2	0
8105	72	13	1	1	1	1	3	3	2	0
8106	25	4	1	7	1	2	4	3	2	0
8107	31	5	1	2	1	2	3	3	2	0
8108	40	7	1	2	1	1	2	3	2	0
8109	34	5	2	2	1	1	3	3	2	0
8110	14	1	2	7	1	2	3	3	2	0
8111	10	1	2	7	1	2	2	3	2	0
8112	63	11	2	2	1	1	2	3	2	0
8113	70	13	1	2	1	1	2	3	2	0
8114	15	2	2	7	1	2	3	3	2	0
8115	18	2	2	7	1	2	3	3	2	0
8116	66	12	1	3	1	1	3	3	2	0
8117	51	9	2	3	1	1	3	3	2	0

8118	17	2	1	7	1	2	3	3	2	0
8119	23	3	1	7	1	2	3	3	2	0
8120	28	4	2	3	1	2	3	3	2	0
8121	22	3	1	3	1	2	3	3	2	0
8122	19	2	1	7	1	2	3	3	2	0
8123	13	1	1	7	1	2	2	3	2	0
8124	24	3	2	8	1	2	3	3	2	0
8125	30	5	2	8	1	1	3	3	2	0
8126	52	9	1	2	1	1	3	3	2	0
8127	38	6	2	3	1	1	3	3	2	0
8128	19	2	2	7	1	2	3	4	2	0
8129	19	2	1	7	1	2	3	4	2	0
8130	10	1	1	7	1	2	3	4	2	0
8131	21	3	2	3	1	2	3	4	2	0
8132	48	8	1	2	1	1	3	4	2	0
8133	40	7	2	2	1	1	3	4	2	0
8134	60	11	1	2	1	4	3	4	2	0
8135	32	5	1	2	1	2	3	4	2	0
8136	28	4	1	3	1	2	3	4	2	0
8137	25	4	2	7	1	2	3	4	2	0
8138	23	3	2	7	1	2	4	4	2	0
8139	68	12	1	3	1	1	2	4	2	0
8140	55	10	2	2	1	1	3	4	2	0
8141	10	1	1	7	1	2	2	4	2	0
8142	16	2	2	7	1	2	3	4	2	0
8143	14	1	1	7	1	2	3	4	2	0
8144	12	1	2	7	1	2	2	4	2	0
8145	21	3	1	7	1	2	4	4	2	0
8146	16	2	1	7	1	2	3	4	2	0
8147	13	1	2	7	1	2	3	4	2	0
8148	19	2	2	7	1	2	3	4	2	0

8149	32	5	1	4	1	2	4	4	2	0
8150	15	2	2	7	1	2	3	4	2	0
8151	27	4	1	7	1	2	4	4	2	0
8152	22	3	2	7	1	2	3	4	2	0
8153	24	3	2	7	1	2	4	4	2	0
8154	40	7	1	2	1	1	3	4	2	0
8155	36	6	2	2	1	1	3	4	2	0
8156	11	1	1	7	1	2	2	4	2	0
8157	73	13	1	2	1	1	2	4	2	0
8158	67	12	2	2	1	1	2	4	2	0
8159	11	1	1	7	1	2	2	4	2	0
8160	18	2	2	7	1	2	3	4	2	0
8161	12	1	2	7	1	2	2	4	2	0
8162	17	2	2	7	1	2	3	4	2	0
8163	31	5	1	2	1	2	3	4	2	0
8164	20	3	2	7	1	2	3	4	2	0
8165	24	3	2	8	1	2	3	4	2	0
8166	53	9	1	2	1	1	2	4	2	0
8167	47	8	2	2	1	1	3	4	2	0
8168	25	4	1	3	1	2	3	4	2	0
8169	22	3	1	7	1	2	3	4	2	0
8170	28	4	2	8	1	2	3	4	2	0
8171	32	5	2	2	1	2	3	4	2	0
8172	60	11	2	2	1	4	2	4	2	0
8173	14	1	1	7	1	2	3	4	2	0
8174	23	3	1	3	1	2	2	3	2	0
8175	39	6	2	2	1	1	3	3	2	0
8176	80	15	1	5	3	1	1	3	2	0
8177	60	11	2	2	1	1	2	3	2	0
8178	10	1	2	7	1	2	2	3	2	0
8179	20	3	1	7	1	2	3	3	2	0

8180	15	2	1	7	1	2	3	3	2	0
8181	23	3	2	7	1	2	3	3	2	0
8182	49	8	2	2	1	1	3	3	2	0
8183	61	11	1	3	1	1	3	3	2	0
8184	54	9	2	2	1	1	3	3	2	0
8185	10	1	2	7	1	2	2	3	2	0
8186	13	1	1	7	1	2	3	3	2	0
8187	17	2	1	7	1	2	3	3	2	0
8188	28	4	1	3	1	2	3	3	2	0
8189	46	8	2	3	1	2	3	3	2	0
8190	68	12	1	2	1	1	2	3	2	0
8191	49	8	2	2	1	1	3	3	2	0
8192	19	2	1	7	1	2	3	3	2	0
8193	37	6	1	3	1	1	2	3	2	0
8194	21	3	2	7	1	2	3	3	2	0
8195	26	4	1	2	1	2	3	3	2	0
8196	13	1	2	7	1	2	3	3	2	0
8197	26	4	1	2	1	2	2	3	2	0
8198	73	13	1	2	1	1	3	4	2	0
8199	31	5	1	3	1	1	3	4	2	0
8200	29	4	2	3	1	1	3	4	2	0
8201	15	2	1	7	1	2	3	4	2	0
8202	51	9	2	2	1	1	3	4	2	0
8203	67	12	2	2	1	1	3	4	2	0
8204	25	4	2	2	1	1	3	4	2	0
8205	32	5	2	3	1	1	3	4	2	0
8206	67	12	1	2	1	1	2	4	2	0
8207	13	1	2	7	1	2	3	4	2	0
8208	20	3	1	7	1	2	3	4	2	0
8209	61	11	2	3	1	1	2	4	2	0
8210	12	1	2	7	1	2	2	4	2	0

8211	10	1	2	7	1	2	2	4	2	0
8212	48	8	1	3	1	1	3	4	2	0
8213	40	7	2	2	1	1	3	4	2	0
8214	31	5	1	3	1	2	3	4	2	0
8215	18	2	1	7	1	2	3	4	2	0
8216	45	8	1	2	1	1	2	4	2	0
8217	38	6	2	2	1	1	3	4	2	0
8218	10	1	2	7	1	2	2	4	2	0
8219	11	1	1	7	1	2	2	4	2	0
8220	15	2	2	7	1	2	3	4	2	0
8221	22	3	2	7	1	2	4	4	2	0
8222	66	12	2	2	1	4	2	4	2	0
8223	19	2	2	7	1	2	3	4	2	0
8224	40	7	1	2	1	1	3	4	2	0
8225	37	6	2	2	1	1	3	4	2	0
8226	35	6	1	2	1	1	3	4	2	0
8227	30	5	2	2	1	1	3	4	2	0
8228	50	9	1	1	1	1	3	4	2	0
8229	18	2	2	7	1	2	3	4	2	0
8230	26	4	1	8	1	2	3	4	2	0
8231	23	3	1	8	1	2	3	4	2	0
8232	46	8	1	2	1	1	3	4	2	0
8233	12	1	1	7	1	2	2	4	2	0
8234	15	2	1	7	1	2	3	4	2	0
8235	17	2	2	7	1	2	3	4	2	0
8236	22	3	2	7	1	2	3	4	2	0
8237	59	10	1	2	1	1	3	4	2	0
8238	16	1	2	7	1	2	3	4	2	0
8239	21	3	2	7	1	2	3	4	2	0
8240	52	9	2	2	1	1	3	4	2	0
8241	31	5	1	2	1	2	2	4	2	0

8242	28	4	1	3	1	2	3	4	2	0
8243	25	4	2	7	1	2	4	4	2	0
8244	20	3	2	7	1	2	3	4	2	0
8245	43	7	2	2	1	1	3	4	2	0
8246	63	11	1	3	1	1	2	4	2	0
8247	14	1	2	7	1	2	3	3	2	0
8248	48	8	2	2	1	1	3	3	2	0
8249	21	3	2	7	1	2	3	3	2	0
8250	27	4	1	3	1	2	3	3	2	0
8251	56	10	2	2	1	2	2	3	2	0
8252	13	1	1	7	1	2	3	3	2	0
8253	61	11	1	1	1	1	3	3	2	0
8254	22	3	2	7	1	2	3	3	2	0
8255	18	2	1	7	1	2	3	3	2	0
8256	37	6	1	3	1	1	3	3	2	0
8257	11	1	1	7	1	2	2	3	2	0
8258	14	1	2	7	1	2	3	3	2	0
8259	15	2	1	7	1	2	3	3	2	0
8260	28	4	2	2	1	1	3	3	2	0
8261	21	3	2	7	1	2	3	3	2	0
8262	57	10	2	2	1	1	3	3	2	0
8263	10	1	2	7	1	2	2	3	2	0
8264	15	2	1	7	1	2	3	3	2	0
8265	17	2	1	7	1	2	3	3	2	0
8266	19	2	1	8	1	2	3	3	2	0
8267	18	2	1	7	1	2	3	3	2	0
8268	62	11	2	4	1	4	4	5	2	0
8269	24	3	1	3	1	2	3	5	2	0
8270	16	2	1	7	1	2	3	5	2	0
8271	52	9	2	2	1	1	3	5	2	0
8272	17	2	2	7	1	2	3	5	2	0

8273	22	3	1	3	1	2	3	5	2	0
8274	57	10	1	2	1	1	3	5	2	0
8275	67	12	1	1	1	1	3	5	2	0
8276	11	1	2	7	1	2	2	5	2	0
8277	16	2	1	7	1	2	3	5	2	0
8278	51	9	2	2	1	1	3	5	2	0
8279	30	5	1	2	1	2	3	5	2	0
8280	26	4	2	7	1	2	3	5	2	0
8281	18	2	2	7	1	2	3	5	2	0
8282	21	3	1	7	1	2	3	5	2	0
8283	28	4	2	7	1	2	3	5	2	0
8284	69	12	1	6	1	1	4	5	2	0
8285	14	1	2	7	1	2	3	5	2	0
8286	21	3	1	8	1	2	3	5	2	0
8287	48	8	1	2	1	1	3	5	2	0
8288	13	1	1	7	1	2	3	5	2	0
8289	39	6	2	2	1	1	3	5	2	0
8290	22	3	1	7	1	2	3	5	2	0
8291	11	1	2	7	1	2	2	5	2	0
8292	12	1	2	7	1	2	2	5	2	0
8293	60	11	2	2	1	4	3	5	2	0
8294	14	1	1	7	1	2	3	5	2	0
8295	54	9	2	1	1	1	3	5	2	0
8296	48	8	1	2	1	1	2	6	2	0
8297	15	2	2	7	1	2	3	6	2	0
8298	43	7	2	2	1	1	3	6	2	0
8299	11	1	2	7	1	2	2	6	2	0
8300	16	2	1	7	1	2	3	6	2	0
8301	13	1	1	7	1	2	3	6	2	0
8302	32	5	1	3	1	1	3	6	2	0
8303	23	3	2	8	1	1	3	6	2	0

8304	16	2	1	7	1	2	3	6	2	0
8305	50	9	1	1	1	1	3	2	2	0
8306	17	2	1	7	1	2	3	2	2	0
8307	21	3	2	7	1	2	3	2	2	0
8308	67	12	2	3	1	4	3	2	2	0
8309	16	2	2	7	1	2	3	2	2	0
8310	23	3	1	3	1	2	3	2	2	0
8311	57	10	2	2	1	1	3	2	2	0
8312	31	5	1	2	1	2	3	2	2	0
8313	27	4	2	7	1	2	4	2	2	0
8314	24	3	1	7	1	2	3	2	2	0
8315	15	2	2	7	1	2	3	2	2	0
8316	14	1	1	7	1	2	3	4	2	0
8317	17	2	2	7	1	2	3	4	2	0
8318	53	9	1	2	1	1	3	4	2	0
8319	45	8	2	2	1	1	3	4	2	0
8320	70	13	1	2	1	4	2	4	2	0
8321	13	1	2	7	1	2	3	4	2	0
8322	47	8	1	2	1	1	3	4	2	0
8323	38	6	2	3	1	1	3	4	2	0
8324	18	2	2	7	1	2	3	4	2	0
8325	25	4	1	7	1	2	4	4	2	0
8326	35	6	1	3	1	2	3	4	2	0
8327	69	12	1	2	3	1	3	4	2	0
8328	10	1	1	7	1	2	2	4	2	0
8329	18	2	2	7	1	2	3	4	2	0
8330	60	11	2	4	1	1	3	4	2	0
8331	37	6	1	3	1	1	3	4	2	0
8332	14	1	2	7	1	2	3	4	2	0
8333	29	4	2	7	1	2	4	4	2	0
8334	13	1	2	7	1	2	2	4	2	0

8335	18	2	1	7	1	2	3	4	2	0
8336	35	6	1	2	1	1	3	4	2	0
8337	49	8	1	1	1	1	3	4	2	0
8338	17	2	1	7	1	2	3	4	2	0
8339	24	3	1	8	1	2	3	4	2	0
8340	28	4	1	3	1	2	3	4	2	0
8341	16	2	2	7	1	2	3	4	2	0
8342	20	3	1	7	1	2	3	4	2	0
8343	23	3	2	7	1	2	4	4	2	0
8344	35	6	2	3	1	1	3	4	2	0
8345	22	3	2	7	1	2	3	4	2	0
8346	35	6	1	2	1	2	3	4	2	0
8347	40	7	2	2	1	4	3	4	2	0
8348	21	3	1	3	1	2	3	4	2	0
8349	15	2	2	7	1	2	3	4	2	0
8350	17	2	2	7	1	2	3	4	2	0
8351	19	2	2	7	1	2	3	4	2	0
8352	58	10	1	2	1	1	3	4	2	0
8353	49	8	2	2	1	1	3	4	2	0
8354	10	1	2	7	1	2	2	4	2	0
8355	26	4	1	7	1	2	4	4	2	0
8356	11	1	2	7	1	2	3	4	2	0
8357	12	1	1	7	1	2	3	4	2	0
8358	24	3	2	7	1	2	4	4	2	0
8359	89	15	2	6	1	4	3	4	2	0
8360	60	11	1	4	1	1	3	4	2	0
8361	52	9	2	4	1	1	4	4	2	0
8362	16	2	2	7	1	2	3	4	2	0
8363	20	3	1	7	1	2	3	4	2	0
8364	32	5	2	3	1	2	3	4	2	0
8365	73	13	1	1	3	1	2	4	2	0

8366	55	10	2	2	1	1	3	4	2	0
8367	66	12	2	2	1	1	2	4	2	0
8368	26	4	2	7	1	2	3	4	2	0
8369	23	3	1	7	1	2	3	4	2	0
8370	36	6	1	4	1	2	4	4	2	0
8371	17	2	1	7	1	2	3	4	2	0
8372	65	12	1	1	1	1	3	4	2	0
8373	54	9	2	2	1	1	3	4	2	0
8374	28	4	1	3	1	1	3	4	2	0
8375	33	5	1	2	1	1	3	4	2	0
8376	16	2	1	7	1	2	3	4	2	0
8377	19	2	2	7	1	2	3	4	2	0
8378	23	3	2	7	1	2	4	4	2	0
8379	18	2	2	7	1	2	3	4	2	0
8380	25	4	1	3	1	2	3	4	2	0
8381	65	12	1	2	1	1	3	4	2	0
8382	10	1	1	7	1	2	2	4	2	0
8383	17	2	1	7	1	2	3	4	2	0
8384	26	4	2	8	1	2	3	4	2	0
8385	39	6	2	3	1	2	3	4	2	0
8386	57	10	1	2	1	1	3	4	2	0
8387	35	6	1	2	1	1	3	4	2	0
8388	15	2	1	7	1	2	3	4	2	0
8389	21	3	1	7	1	2	3	4	2	0
8390	29	4	1	7	1	2	4	4	2	0
8391	30	5	2	2	1	1	3	4	2	0
8392	70	13	1	5	1	1	3	4	2	0
8393	14	1	1	7	1	2	3	4	2	0
8394	18	2	2	7	1	2	3	4	2	0
8395	61	11	2	2	1	1	3	4	2	0
8396	40	7	1	2	1	1	3	4	2	0

8397	21	3	2	7	1	2	3	4	2	0
8398	27	4	1	7	1	2	4	4	2	0
8399	30	5	2	3	1	1	3	4	2	0
8400	57	10	1	3	1	1	3	4	2	0
8401	17	2	1	7	1	2	3	4	2	0
8402	13	1	2	7	1	2	2	4	2	0
8403	50	9	2	3	1	1	3	4	2	0
8404	18	2	1	7	1	2	3	4	2	0
8405	14	1	2	7	1	2	3	4	2	0
8406	53	9	2	4	1	1	3	4	2	0
8407	17	2	1	7	1	2	3	4	2	0
8408	16	2	1	7	1	2	3	4	2	0
8409	17	2	1	7	1	2	3	4	2	0
8410	68	12	1	2	1	1	2	4	2	0
8411	59	10	2	2	1	1	3	4	2	0
8412	23	3	1	7	1	2	3	4	2	0
8413	15	2	1	7	1	2	3	4	2	0
8414	20	3	2	7	1	2	3	4	2	0
8415	13	1	2	7	1	2	3	4	2	0
8416	19	2	1	7	1	2	3	4	2	0
8417	72	13	2	2	1	4	2	4	2	0
8418	51	9	1	3	1	1	2	4	2	0
8419	19	2	1	7	1	2	3	4	2	0
8420	48	8	1	2	1	1	3	4	2	0
8421	43	7	1	4	1	1	4	4	2	0
8422	45	8	2	2	1	1	3	4	2	0
8423	41	7	2	2	1	1	3	4	2	0
8424	37	6	2	3	1	1	3	4	2	0
8425	18	2	1	7	1	2	3	4	2	0
8426	16	2	1	7	1	2	3	4	2	0
8427	23	3	1	7	1	2	3	4	2	0

8428	35	6	2	2	1	2	3	4	2	0
8429	11	1	2	7	1	2	2	4	2	0
8430	12	1	1	7	1	2	2	4	2	0
8431	15	2	1	7	1	2	3	4	2	0
8432	13	1	1	7	1	2	2	4	2	0
8433	22	3	2	7	1	2	4	4	2	0
8434	27	4	1	7	1	2	4	4	2	0
8435	53	9	1	2	1	1	2	2	2	0
8436	48	8	2	2	1	1	3	2	2	0
8437	19	2	1	7	1	2	3	2	2	0
8438	25	4	1	7	1	2	4	2	2	0
8439	22	3	2	7	1	2	3	2	2	0
8440	62	11	2	2	1	4	3	2	2	0
8441	21	3	1	7	1	2	3	2	2	0
8442	26	4	2	3	1	2	3	2	2	0
8443	36	6	1	2	1	1	3	2	2	0
8444	28	4	2	3	1	1	3	2	2	0
8445	20	3	2	7	1	2	3	2	2	0
8446	28	4	1	3	1	2	3	6	2	0
8447	79	14	2	5	1	4	2	2	2	0
8448	59	10	1	4	1	1	4	2	2	0
8449	19	2	1	7	1	2	3	2	2	0
8450	21	3	1	7	1	2	3	2	2	0
8451	41	7	1	2	1	1	3	2	2	0
8452	46	8	1	1	1	1	3	2	2	0
8453	39	6	2	3	1	1	3	2	2	0
8454	17	2	2	7	1	2	3	2	2	0
8455	52	9	1	3	1	1	3	2	2	0
8456	14	1	2	7	1	2	3	2	2	0
8457	16	2	2	7	1	2	3	2	2	0
8458	17	2	2	7	1	2	3	2	2	0

8459	18	2	1	7	1	2	3	2	2	0
8460	61	11	1	2	3	1	2	2	2	0
8461	14	1	1	7	1	2	3	2	2	0
8462	54	9	2	2	1	1	3	2	2	0
8463	20	3	1	7	1	2	3	2	2	0
8464	32	5	1	3	1	2	3	2	2	0
8465	27	4	1	2	1	2	3	2	2	0
8466	39	6	2	2	1	1	3	2	2	0
8467	20	3	1	7	1	2	3	2	2	0
8468	56	10	2	3	1	4	3	2	2	0
8469	37	6	1	3	1	1	3	2	2	0
8470	24	3	2	3	1	2	3	2	2	0
8471	38	6	1	2	1	1	2	6	2	0
8472	26	4	2	2	1	1	3	6	2	0
8473	75	14	1	2	1	1	2	2	2	0
8474	25	4	1	7	1	2	3	2	2	0
8475	18	2	1	7	1	2	3	2	2	0
8476	28	4	2	7	1	2	3	2	2	0
8477	30	5	2	3	1	4	3	2	2	0
8478	65	12	2	1	1	1	3	2	2	0
8479	54	9	2	2	1	1	3	2	2	0
8480	35	6	1	2	1	1	3	2	2	0
8481	61	11	1	2	1	1	2	2	2	0
8482	56	10	2	5	1	1	3	2	2	0
8483	12	1	2	7	1	2	3	2	2	0
8484	20	3	2	7	1	2	3	2	2	0
8485	20	3	1	8	1	2	3	2	2	0
8486	18	2	2	7	1	2	3	2	2	0
8487	27	4	1	3	1	2	3	2	2	0
8488	69	12	1	1	1	1	3	2	2	0
8489	50	9	2	2	1	1	3	2	2	0

8490	17	2	2	7	1	2	3	2	2	0
8491	23	3	1	2	1	2	2	2	2	0
8492	21	3	2	7	1	2	3	2	2	0
8493	45	8	2	4	1	2	3	2	2	0
8494	24	3	1	7	1	2	4	2	2	0
8495	76	14	1	2	1	1	2	2	2	0
8496	12	1	2	7	1	2	2	2	2	0
8497	17	2	1	7	1	2	3	2	2	0
8498	64	11	2	2	1	1	3	2	2	0
8499	42	7	2	3	1	2	3	2	2	0
8500	14	1	1	7	1	2	3	2	2	0
8501	14	1	1	7	1	2	3	2	2	0
8502	13	1	1	7	1	2	2	2	2	0
8503	42	7	1	2	1	1	3	2	2	0
8504	32	5	2	2	1	1	3	2	2	0
8505	17	2	1	7	1	2	3	2	2	0
8506	12	1	1	7	1	2	2	2	2	0
8507	38	6	1	2	1	1	2	2	2	0
8508	25	4	2	3	1	1	3	2	2	0
8509	11	1	2	7	1	2	2	2	2	0
8510	13	1	1	7	1	2	3	2	2	0
8511	35	6	1	2	1	2	3	2	2	0
8512	10	1	1	7	1	2	2	6	2	0
8513	19	2	2	7	1	2	3	2	2	0
8514	23	3	1	7	1	2	4	4	2	0
8515	13	1	1	7	1	2	3	2	2	0
8516	20	3	2	7	1	2	3	2	2	0
8517	17	2	1	7	1	2	3	2	2	0
8518	52	9	1	3	1	1	2	6	2	0
8519	19	2	1	7	1	2	3	6	2	0
8520	16	2	2	7	1	2	3	6	2	0

8521	43	7	2	2	1	1	3	6	2	0
8522	14	1	1	7	1	2	3	6	2	0
8523	34	5	1	3	1	2	2	6	2	0
8524	53	9	2	2	1	4	3	2	2	0
8525	73	13	1	1	1	1	2	2	2	0
8526	61	11	2	1	1	1	3	2	2	0
8527	12	1	2	7	1	2	2	2	2	0
8528	14	1	1	7	1	2	3	2	2	0
8529	44	7	1	2	1	1	3	2	2	0
8530	17	2	1	7	1	2	3	2	2	0
8531	34	5	1	2	1	2	3	2	2	0
8532	33	5	2	3	1	2	3	2	2	0
8533	27	4	2	3	1	1	3	2	2	0
8534	16	2	2	7	1	2	3	2	2	0
8535	47	8	1	3	1	1	3	2	2	0
8536	19	2	1	7	1	2	3	2	2	0
8537	20	3	2	7	1	2	4	4	2	0
8538	26	4	1	7	1	2	4	2	2	0
8539	15	2	2	7	1	2	3	2	2	0
8540	22	3	1	8	1	2	3	2	2	0
8541	41	7	2	2	1	1	3	2	2	0
8542	30	5	1	2	1	2	3	2	2	0
8543	25	4	1	7	1	2	4	2	2	0
8544	13	1	2	7	1	2	3	2	2	0
8545	31	5	1	2	1	1	3	2	2	0
8546	67	12	2	2	1	4	2	2	2	0
8547	37	6	1	2	1	1	3	2	2	0
8548	40	7	1	2	1	1	3	2	2	0
8549	25	4	2	2	1	1	3	2	2	0
8550	38	6	2	2	1	1	3	2	2	0
8551	15	2	2	7	1	2	3	2	2	0

8552	35	6	2	3	1	2	3	2	2	0
8553	14	1	1	7	1	2	3	2	2	0
8554	17	2	2	7	1	2	3	2	2	0
8555	13	1	1	7	1	2	3	2	2	0
8556	20	3	1	7	1	2	4	4	2	0
8557	73	13	2	2	1	4	2	2	2	0
8558	51	9	1	2	1	1	3	2	2	0
8559	22	2	1	3	1	2	3	2	2	0
8560	43	7	1	2	1	1	3	2	2	0
8561	49	8	1	2	1	1	3	2	2	0
8562	45	8	2	2	1	1	3	2	2	0
8563	19	2	2	7	1	2	3	2	2	0
8564	25	4	2	7	1	2	4	2	2	0
8565	27	4	1	7	1	2	4	2	2	0
8566	41	7	2	2	1	1	3	2	2	0
8567	17	2	1	7	1	2	3	2	2	0
8568	20	3	1	3	1	2	2	2	2	0
8569	27	4	1	2	1	2	3	2	2	0
8570	66	12	1	2	1	1	3	2	2	0
8571	53	9	2	2	1	1	3	2	2	0
8572	52	9	1	2	1	1	2	2	2	0
8573	42	7	2	2	1	1	3	2	2	0
8574	13	1	1	7	1	2	3	2	2	0
8575	17	2	1	7	1	2	3	2	2	0
8576	11	1	1	7	1	2	2	2	2	0
8577	50	9	2	2	1	1	3	2	2	0
8578	12	1	1	7	1	2	2	2	2	0
8579	16	2	1	7	1	2	3	2	2	0
8580	28	4	1	3	1	2	3	2	2	0
8581	59	10	1	2	1	1	3	2	2	0
8582	42	7	2	5	1	2	1	2	2	0

8583	14	1	2	7	1	2	3	2	2	0
8584	36	6	2	2	1	2	3	2	2	0
8585	19	2	1	7	1	2	3	2	2	0
8586	22	3	1	8	1	2	3	2	2	0
8587	18	2	1	7	1	2	3	2	2	0
8588	49	8	2	2	1	1	3	2	2	0
8589	58	10	2	2	1	4	2	2	2	0
8590	13	1	2	7	1	2	3	2	2	0
8591	16	2	2	7	1	2	3	2	2	0
8592	21	3	2	7	1	2	3	2	2	0
8593	41	7	2	2	1	1	3	2	2	0
8594	19	2	2	7	1	2	3	2	2	0
8595	60	11	2	1	1	4	2	2	2	0
8596	21	3	2	8	1	2	3	2	2	0
8597	44	7	1	2	1	1	3	2	2	0
8598	37	6	2	2	1	1	3	2	2	0
8599	13	1	1	7	1	2	2	2	2	0
8600	18	2	1	7	1	2	3	2	2	0
8601	11	1	2	7	1	2	2	2	2	0
8602	16	2	2	7	1	2	3	2	2	0
8603	69	12	1	6	1	1	4	2	2	0
8604	10	1	1	7	1	2	2	2	2	0
8605	18	2	1	7	1	2	3	2	2	0
8606	57	10	2	4	1	1	4	2	2	0
8607	19	2	2	7	1	2	3	2	2	0
8608	16	2	2	7	1	2	3	2	2	0
8609	21	3	2	7	1	2	4	2	2	0
8610	26	4	1	7	1	2	4	2	2	0
8611	15	2	2	7	1	2	3	2	2	0
8612	16	2	2	7	1	2	3	2	2	0
8613	12	1	2	7	1	2	2	2	2	0

8614	17	2	2	7	1	2	3	2	2	0
8615	30	5	2	2	1	4	3	2	2	0
8616	65	12	1	1	1	1	1	2	2	0
8617	11	1	1	7	1	2	2	2	2	0
8618	16	2	2	7	1	2	3	2	2	0
8619	18	2	1	7	1	2	3	2	2	0
8620	27	4	1	7	1	2	4	2	2	0
8621	13	1	1	7	1	2	3	2	2	0
8622	71	13	2	5	1	4	2	2	2	0
8623	44	7	1	2	1	1	3	2	2	0
8624	36	6	2	2	1	1	3	2	2	0
8625	20	3	1	7	1	2	3	2	2	0
8626	48	8	1	2	1	1	2	2	2	0
8627	42	7	2	3	1	1	3	2	2	0
8628	15	2	2	7	1	2	3	2	2	0
8629	67	12	2	2	1	4	1	4	2	0
8630	42	7	2	7	1	2	4	4	2	0
8631	38	6	1	2	1	1	2	4	2	0
8632	28	4	2	8	1	1	3	4	2	0
8633	35	6	1	3	1	2	3	4	2	0
8634	32	5	2	3	1	2	3	4	2	0
8635	28	4	2	3	1	1	3	4	2	0
8636	30	5	1	3	1	1	3	4	2	0
8637	24	3	2	2	1	1	3	4	2	0
8638	60	11	1	2	1	1	2	4	2	0
8639	56	10	2	1	1	1	1	4	2	0
8640	14	1	2	7	1	2	3	4	2	0
8641	16	2	1	7	1	2	3	4	2	0
8642	22	3	2	7	1	2	3	4	2	0
8643	20	3	2	7	1	2	3	4	2	0
8644	25	4	1	2	1	2	3	4	2	0

8645	45	8	1	2	1	1	3	4	2	0
8646	36	6	2	2	1	1	3	4	2	0
8647	10	1	2	7	1	2	2	4	2	0
8648	67	12	1	1	1	1	3	4	2	0
8649	14	1	2	7	1	2	3	4	2	0
8650	50	9	2	2	1	1	2	4	2	0
8651	20	3	2	7	1	2	3	4	2	0
8652	23	3	2	7	1	2	4	4	2	0
8653	18	2	1	7	1	2	3	4	2	0
8654	36	6	2	3	1	2	3	4	2	0
8655	21	3	1	3	1	2	3	4	2	0
8656	41	7	2	3	1	2	3	4	2	0
8657	17	2	2	7	1	2	3	4	2	0
8658	63	11	1	2	1	1	1	4	2	0
8659	14	1	1	7	1	2	3	4	2	0
8660	20	3	1	7	1	2	3	4	2	0
8661	19	2	2	7	1	2	3	4	2	0
8662	29	4	1	3	1	1	2	4	2	0
8663	56	10	2	2	1	1	3	4	2	0
8664	40	7	2	1	1	2	3	4	2	0
8665	16	2	2	7	1	2	3	4	2	0
8666	24	3	2	7	1	2	4	4	2	0
8667	48	8	1	2	1	1	2	4	2	0
8668	39	6	2	2	1	1	3	4	2	0
8669	17	2	1	7	1	2	3	4	2	0
8670	21	3	1	8	1	2	2	4	2	0
8671	51	9	2	2	1	4	2	4	2	0
8672	19	2	1	7	1	2	3	4	2	0
8673	27	4	1	3	1	2	3	4	2	0
8674	13	1	1	7	1	2	2	4	2	0
8675	17	2	2	7	1	2	3	4	2	0

8676	58	10	1	3	1	1	2	4	2	0
8677	53	9	2	2	1	1	3	4	2	0
8678	36	6	1	2	1	1	3	4	2	0
8679	15	2	2	7	1	2	3	4	2	0
8680	30	5	2	2	1	1	3	4	2	0
8681	48	8	1	2	1	1	2	4	2	0
8682	41	7	2	1	1	1	3	4	2	0
8683	16	2	1	7	1	2	3	4	2	0
8684	21	3	1	8	1	2	2	4	2	0
8685	14	1	1	7	1	2	3	4	2	0
8686	17	2	1	7	1	2	3	4	2	0
8687	19	2	1	7	1	2	3	4	2	0
8688	25	4	1	3	1	2	2	4	2	0
8689	71	13	2	2	1	4	1	4	2	0
8690	46	8	1	2	1	1	3	4	2	0
8691	39	6	2	2	1	1	3	4	2	0
8692	44	7	1	3	1	1	3	4	2	0
8693	36	6	2	2	1	1	3	4	2	0
8694	39	6	1	2	1	1	3	4	2	0
8695	11	1	1	7	1	2	2	4	2	0
8696	13	1	1	7	1	2	3	4	2	0
8697	18	2	1	7	1	2	3	4	2	0
8698	16	2	1	7	1	2	3	4	2	0
8699	77	14	2	5	1	4	3	4	2	0
8700	40	7	1	2	1	1	3	4	2	0
8701	57	10	1	4	1	1	4	4	2	0
8702	36	6	2	2	1	1	3	4	2	0
8703	16	2	1	7	1	1	3	4	2	0
8704	48	8	1	2	1	1	4	4	2	0
8705	43	7	2	4	1	1	4	4	2	0
8706	15	2	2	7	1	2	3	4	2	0

8707	23	3	1	7	1	2	4	4	2	0
8708	20	3	2	7	1	2	4	4	2	0
8709	38	6	2	8	1	2	4	4	2	0
8710	52	9	1	2	1	1	3	4	2	0
8711	46	8	2	2	1	1	3	4	2	0
8712	13	1	2	7	1	2	3	4	2	0
8713	11	1	1	7	1	2	2	4	2	0
8714	41	7	1	3	1	1	2	6	2	0
8715	36	6	2	2	1	1	3	6	2	0
8716	12	1	1	7	1	2	2	6	2	0
8717	47	8	1	2	1	1	3	1	2	0
8718	39	6	2	3	1	1	3	1	2	0
8719	23	3	1	8	1	2	2	6	2	0
8720	53	9	2	2	1	4	3	1	2	0
8721	13	1	1	7	1	2	3	1	2	0
8722	16	2	2	7	1	2	3	1	2	0
8723	45	8	2	2	1	1	3	1	2	0
8724	19	2	2	7	1	2	3	1	2	0
8725	23	3	2	7	1	2	4	1	2	0
8726	60	11	1	1	1	1	3	2	2	0
8727	19	2	1	7	1	2	3	1	2	0
8728	40	7	2	2	1	2	3	1	2	0
8729	12	1	1	7	1	2	2	1	2	0
8730	42	7	1	2	1	1	3	1	2	0
8731	37	6	2	2	1	1	3	1	2	0
8732	28	4	1	7	1	2	4	1	2	0
8733	58	10	2	3	1	1	2	1	2	0
8734	20	3	1	7	1	2	3	1	2	0
8735	24	3	2	8	1	2	3	1	2	0
8736	15	2	2	7	1	2	3	1	2	0
8737	39	6	1	2	1	1	2	6	2	0

8738	26	4	2	8	1	1	3	6	2	0
8739	68	12	2	2	1	4	2	4	2	0
8740	31	5	1	3	1	2	3	4	2	0
8741	28	4	1	7	1	2	4	4	2	0
8742	25	4	2	7	1	2	3	4	2	0
8743	20	3	2	7	1	2	3	4	2	0
8744	52	9	2	3	1	1	3	4	2	0
8745	25	4	1	3	1	2	3	4	2	0
8746	18	2	1	7	1	2	3	4	2	0
8747	21	3	1	7	1	2	3	4	2	0
8748	23	3	2	7	1	2	3	4	2	0
8749	40	7	1	3	1	1	3	4	2	0
8750	10	1	2	7	1	2	2	4	2	0
8751	11	1	1	7	1	2	2	4	2	0
8752	20	3	2	7	1	2	3	4	2	0
8753	36	6	2	2	1	1	3	4	2	0
8754	18	2	1	7	1	2	3	4	2	0
8755	69	12	1	2	1	1	3	4	2	0
8756	24	3	2	7	1	2	3	4	2	0
8757	17	2	2	7	1	2	3	4	2	0
8758	12	1	1	7	1	2	2	4	2	0
8759	19	2	1	7	1	2	3	4	2	0
8760	15	2	2	7	1	2	3	4	2	0
8761	13	1	2	7	1	2	3	4	2	0
8762	20	3	2	7	1	2	3	4	2	0
8763	61	11	2	2	1	1	3	4	2	0
8764	14	1	2	7	1	2	3	4	2	0
8765	29	4	1	3	1	1	3	4	2	0
8766	26	4	1	2	1	2	3	4	2	0
8767	24	3	2	3	1	2	3	4	2	0
8768	82	15	1	6	1	1	4	4	2	0

8769	70	13	2	6	1	1	4	4	2	0
8770	50	9	1	2	1	1	4	4	2	0
8771	42	7	2	4	1	1	4	4	2	0
8772	45	8	1	2	1	1	4	4	2	0
8773	28	4	2	8	1	1	4	4	2	0
8774	32	5	1	5	1	2	1	4	2	0
8775	30	5	1	2	1	2	4	4	2	0
8776	13	1	2	7	1	2	3	4	2	0
8777	11	1	1	7	1	2	3	4	2	0
8778	14	1	1	7	1	2	3	4	2	0
8779	12	1	2	7	1	2	2	4	2	0
8780	56	10	1	2	1	1	3	4	2	0
8781	42	7	2	4	1	1	3	4	2	0
8782	15	2	1	7	1	2	3	4	2	0
8783	13	1	1	7	1	2	3	4	2	0
8784	10	1	1	7	1	2	2	4	2	0
8785	52	9	1	2	1	1	3	4	2	0
8786	43	7	2	8	1	1	3	4	2	0
8787	10	1	1	7	1	2	2	4	2	0
8788	49	8	2	2	1	4	3	4	2	0
8789	18	2	1	7	1	2	3	4	2	0
8790	16	2	1	7	1	2	3	4	2	0
8791	13	1	2	7	1	2	3	4	2	0
8792	11	1	1	7	1	2	2	4	2	0
8793	28	4	2	3	1	2	3	4	2	0
8794	66	12	1	2	1	1	3	4	2	0
8795	60	11	2	2	1	1	3	4	2	0
8796	38	6	1	2	1	1	3	4	2	0
8797	44	7	2	3	1	1	3	4	2	0
8798	13	1	2	7	1	2	2	2	2	0
8799	11	1	2	7	1	2	2	2	2	0

8800	34	5	1	2	1	1	3	2	2	0
8801	28	4	2	2	1	1	3	2	2	0
8802	55	10	1	1	1	1	3	1	2	0
8803	45	8	2	2	1	1	3	1	2	0
8804	11	1	2	7	1	2	2	1	2	0
8805	16	2	1	7	1	2	3	1	2	0
8806	19	2	1	7	1	2	3	1	2	0
8807	42	7	1	1	1	1	2	6	2	0
8808	34	5	2	1	1	1	2	6	2	0
8809	19	2	1	7	1	2	3	6	2	0
8810	13	1	1	7	1	2	2	6	2	0
8811	10	1	1	7	1	2	2	6	2	0
8812	71	13	1	5	1	1	3	5	2	0
8813	58	10	2	3	1	1	3	5	2	0
8814	17	2	1	7	1	2	3	5	2	0
8815	15	2	1	7	1	2	3	5	2	0
8816	20	3	1	7	1	2	3	5	2	0
8817	43	7	1	2	1	1	3	5	2	0
8818	27	4	1	3	1	2	3	5	2	0
8819	12	1	2	7	1	2	3	5	2	0
8820	36	6	2	2	1	1	3	5	2	0
8821	21	3	1	8	1	2	3	5	2	0
8822	65	12	1	2	1	1	3	5	2	0
8823	59	10	2	2	1	1	2	5	2	0
8824	41	7	1	3	1	1	2	5	2	0
8825	11	1	1	7	1	2	2	5	2	0
8826	33	5	2	2	1	1	3	5	2	0
8827	24	3	2	7	1	2	3	5	2	0
8828	14	1	2	7	1	2	3	5	2	0
8829	15	2	2	7	1	2	3	5	2	0
8830	20	3	1	7	1	2	3	5	2	0

8831	46	8	2	3	1	4	3	5	2	0
8832	10	1	1	7	1	2	2	5	2	0
8833	16	2	1	7	1	2	3	5	2	0
8834	13	1	2	7	1	2	2	5	2	0
8835	61	11	2	5	1	1	2	5	2	0
8836	70	13	1	1	1	1	2	5	2	0
8837	30	5	2	4	1	2	3	5	2	0
8838	27	4	1	7	1	2	4	5	2	0
8839	24	3	1	3	1	2	3	5	2	0
8840	64	11	2	2	1	1	2	5	2	0
8841	43	7	1	2	1	1	3	6	2	0
8842	10	1	1	7	1	2	2	6	2	0
8843	31	5	2	2	1	1	3	5	2	0
8844	55	10	1	2	1	1	2	5	2	0
8845	48	8	2	3	1	1	3	5	2	0
8846	11	1	1	7	1	2	2	5	2	0
8847	13	1	2	7	1	2	2	5	2	0
8848	20	3	1	7	1	2	3	5	2	0
8849	35	6	1	3	1	2	3	6	2	0
8850	68	12	2	2	1	4	3	5	2	0
8851	18	2	2	7	1	2	3	5	2	0
8852	15	2	2	7	1	2	3	5	2	0
8853	54	9	1	3	1	1	2	5	2	0
8854	12	1	1	7	1	2	2	5	2	0
8855	19	2	2	7	1	2	3	5	2	0
8856	35	6	2	3	1	2	3	5	2	0
8857	46	8	2	2	1	1	3	5	2	0
8858	14	1	1	7	1	2	3	5	2	0
8859	69	12	1	2	3	1	2	5	2	0
8860	56	10	2	2	1	1	3	5	2	0
8861	42	7	2	4	1	2	3	5	2	0

8862	15	2	2	7	1	2	3	5	2	0
8863	27	4	1	2	1	2	3	5	2	0
8864	63	11	1	1	1	1	3	5	2	0
8865	52	9	2	2	1	1	3	5	2	0
8866	19	2	1	7	1	2	3	5	2	0
8867	16	2	2	7	1	2	3	5	2	0
8868	14	1	2	7	1	2	3	5	2	0
8869	23	3	1	7	1	2	3	5	2	0
8870	65	12	2	6	1	4	4	5	2	0
8871	32	5	2	4	1	2	4	5	2	0
8872	21	3	1	7	1	2	4	5	2	0
8873	24	3	1	7	1	2	4	5	2	0
8874	48	8	1	2	1	1	3	5	2	0
8875	32	5	2	2	1	1	3	5	2	0
8876	56	10	1	2	1	1	3	5	2	0
8877	38	6	1	2	1	1	3	5	2	0
8878	16	2	1	7	1	2	3	5	2	0
8879	47	8	2	3	1	1	3	5	2	0
8880	17	2	2	7	1	2	3	5	2	0
8881	19	2	1	7	1	2	3	5	2	0
8882	42	7	2	2	1	1	3	5	2	0
8883	50	9	1	3	1	1	3	5	2	0
8884	43	7	1	3	1	1	3	5	2	0
8885	18	2	1	8	1	2	2	5	2	0
8886	37	6	2	2	1	1	3	5	2	0
8887	24	3	1	8	1	2	3	5	2	0
8888	53	9	1	2	1	1	2	5	2	0
8889	46	8	2	2	1	1	3	5	2	0
8890	13	1	2	7	1	2	3	5	2	0
8891	17	2	2	7	1	2	3	5	2	0
8892	19	2	1	7	1	2	3	5	2	0

8893	67	12	1	2	1	1	2	5	2	0
8894	20	3	1	7	1	2	3	5	2	0
8895	47	8	2	2	1	1	3	5	2	0
8896	16	2	2	7	1	2	3	5	2	0
8897	22	3	2	7	1	2	3	5	2	0
8898	70	13	1	1	1	1	2	5	2	0
8899	60	11	2	2	1	1	3	5	2	0
8900	56	10	1	2	1	1	3	5	2	0
8901	47	8	2	2	1	1	3	5	2	0
8902	19	2	1	7	1	2	3	5	2	0
8903	23	3	1	3	1	2	2	5	2	0
8904	37	6	2	3	1	2	3	5	2	0
8905	34	5	1	2	1	2	3	5	2	0
8906	54	9	2	2	1	4	3	5	2	0
8907	23	3	2	7	1	2	3	5	2	0
8908	16	2	1	7	1	2	3	5	2	0
8909	18	2	1	7	1	2	3	5	2	0
8910	20	3	2	7	1	2	3	5	2	0
8911	61	11	1	3	1	1	2	5	2	0
8912	52	9	2	2	1	1	3	5	2	0
8913	30	5	1	2	1	1	3	5	2	0
8914	26	4	2	7	1	1	4	5	2	0
8915	95	15	1	6	1	4	3	5	2	0
8916	67	12	1	2	1	1	3	5	2	0
8917	59	10	2	2	1	1	3	5	2	0
8918	64	11	1	2	1	1	3	5	2	0
8919	56	10	2	4	1	1	4	5	2	0
8920	60	11	1	2	1	1	3	5	2	0
8921	48	8	2	4	1	1	3	5	2	0
8922	30	5	1	3	1	1	3	5	2	0
8923	24	3	2	3	1	1	3	5	2	0

8924	58	10	2	4	1	2	4	5	2	0
8925	47	8	1	2	1	1	3	5	2	0
8926	25	4	2	7	1	2	4	5	2	0
8927	37	6	2	4	1	1	4	5	2	0
8928	32	5	2	4	1	2	3	5	2	0
8929	72	13	1	2	1	4	3	5	2	0
8930	12	1	2	7	1	2	2	5	2	0
8931	15	2	2	7	1	2	3	5	2	0
8932	14	1	1	7	1	2	3	5	2	0
8933	16	2	2	7	1	2	3	5	2	0
8934	69	12	1	3	1	1	2	6	2	0
8935	56	10	2	2	1	1	3	6	2	0
8936	40	7	1	1	1	1	3	6	2	0
8937	35	6	2	2	1	1	3	6	2	0
8938	13	1	2	7	1	2	3	6	2	0
8939	74	13	1	6	1	1	4	6	2	0
8940	24	3	2	8	1	2	3	6	2	0
8941	64	11	2	4	1	1	4	6	2	0
8942	27	4	1	3	1	1	2	6	2	0
8943	22	3	2	3	1	1	2	6	2	0
8944	19	2	1	7	1	2	3	6	2	0
8945	22	3	2	7	1	2	3	6	2	0
8946	18	2	2	7	1	2	3	6	2	0
8947	49	8	1	2	1	1	3	6	2	0
8948	11	1	2	7	1	2	2	6	2	0
8949	29	4	1	2	1	2	3	6	2	0
8950	14	1	1	7	1	2	3	6	2	0
8951	58	10	1	2	1	1	3	6	2	0
8952	23	3	1	7	1	2	3	6	2	0
8953	15	2	1	7	1	2	3	6	2	0
8954	70	13	2	1	1	4	2	6	2	0

8955	64	11	1	2	1	1	3	6	2	0
8956	38	6	1	3	1	2	3	6	2	0
8957	46	8	2	2	1	1	3	6	2	0
8958	17	2	1	7	1	2	3	6	2	0
8959	15	2	1	7	1	2	3	6	2	0
8960	22	3	1	7	1	2	3	6	2	0
8961	24	3	1	7	1	2	3	6	2	0
8962	25	4	1	7	1	2	3	6	2	0
8963	72	13	2	5	1	4	2	6	2	0
8964	81	15	1	5	1	1	3	6	2	0
8965	70	13	2	2	1	1	3	6	2	0
8966	62	11	2	2	1	1	3	6	2	0
8967	47	8	2	2	1	2	3	6	2	0
8968	20	3	2	7	1	2	3	6	2	0
8969	17	2	2	7	1	2	3	6	2	0
8970	17	2	2	7	1	2	3	6	2	0
8971	19	2	2	7	1	2	3	6	2	0
8972	22	3	2	8	1	2	3	6	2	0
8973	21	3	2	7	1	2	3	6	2	0
8974	28	4	2	3	1	1	3	2	2	0
8975	60	11	2	2	1	1	2	2	2	0
8976	74	13	1	2	1	1	2	2	2	0
8977	37	6	1	1	1	1	2	2	2	0
8978	25	4	1	7	1	2	4	2	2	0
8979	31	5	1	3	1	2	3	2	2	0
8980	30	5	1	2	1	2	2	2	2	0
8981	37	6	2	2	1	1	3	2	2	0
8982	47	8	1	2	1	1	3	2	2	0
8983	18	2	1	7	1	2	3	2	2	0
8984	72	13	1	2	1	1	3	4	2	0
8985	65	12	2	2	1	1	3	4	2	0

8986	27	4	1	7	1	2	4	4	2	0
8987	65	12	1	2	1	1	2	4	2	0
8988	62	11	2	2	1	1	2	4	2	0
8989	60	11	1	5	1	1	3	4	2	0
8990	53	9	2	2	1	1	3	4	2	0
8991	22	3	2	7	1	2	3	4	2	0
8992	21	3	1	7	1	2	3	4	2	0
8993	15	2	1	7	1	2	3	4	2	0
8994	13	1	1	7	1	2	3	4	2	0
8995	27	4	1	8	1	2	4	4	2	0
8996	16	2	2	7	1	2	3	4	2	0
8997	34	5	1	3	1	1	3	4	2	0
8998	17	2	1	7	1	2	3	4	2	0
8999	18	2	1	7	1	2	3	4	2	0
9000	47	8	2	2	1	1	3	4	2	0

KEYS

Sex: 1= Male; 2= Female

Age Range: 1= 10-14; 2= 15-19; 3=20-24; 4=25-29; 5=30-34; 6=35-39; 7=40-44; 8=45-49; 9=50-54; 10=55-59; 11=60-64; 12=65-69; 13=70-74; 14=75-79; 15=80 and above

Residence: 1= Umunachi; 2= Umudioka ; 3= Ifitedunu ; 4= Ukwulu ; 5= Ukpo; 6= Nawgu

Occupation: 1= Farmer; 2=Trader; 3=Artisan; 4=Civil servant; 5=Dependant; 6=Retiree; 7=Student; 8= Others

Religion: 1= Christianity; 2= Islam; 3= African traditional religions; 4=Atheism; 5= No response

Marital Status: 1= Married; 2= Single; 3= Divorced; 4= Widow/widower; 5=Separated; 6= No response

Highest educational Level attained (HELA): 1= None; 2= Primary; 3=Secondary; 4= Tertiary

Shaking (8)/ Fall (9): 1= Yes; 2= No

Outcome: 0= Do not have epilepsy; 1= Confirmed case of active epilepsy; 2= Confirmed case of inactive epilepsy
