# Supplementary material for: Prevalence of active convulsive epilepsy in Dunukofia County in South East Nigeria: a door-to-door survey
Source: Acta Epileptol. 2025 Mar 24;7:21. doi: 10.1186/s42494-024-00200-4 (PMC11960363; doi:10.1186/s42494-024-00200-4)
Supplement: Supplementary file 2 — Supplementary Material 2. [file 42494_2024_200_MOESM2_ESM.pdf]

Age and sex distribution of the study population in the six towns of Dunkofia LGA.

**Age range**	**Umunachi** Number (%)			**Umudioka** Number (%)			**Ifitedunu** Number (%)			**Ukwulu** Number (%)			**Ukpo** Number (%)			**Nawgu** Number (%)			**Total** Number (%)		
	**M**	**F**	**T**	**M**	**F**	**T**	**M**	**F**	**T**	**M**	**F**	**T**	M	F	T	M	F	T	M	F	T
10-19	141 (11.8)	116 (9.7)	257 (21.5)	84 (8.2)	409 (10.6)	193 (18.7)	130 (8.6)	98 (6.5)	228 (15.0)	359 (11.8)	351 (11.6)	710 (23.4)	92 (6.6)	91 (6.5)	183 (13.1)	80 (9.7)	60 (7.3)	140 (17.1)	886 (9.8)	825 (9.2)	1711 (19.0)
20-29	91 (7.6)	87 (7.3)	178 (14.9)	68 (6.6)	68 (6.6)	136 (13.2)	66 (4.3)	78 (5.1)	144 (9.5)	211 (7.0)	242 (14.9)	453 (14.9)	74 (5.3)	64 (4.6)	138 (9.9)	48 (5.8)	61 (7.4)	109 (13.3)	558 (6.2)	600 (6.7)	1158 (12.9)
30-39	85 (7.1)	96 (8.0)	181 (15.1)	79 (7.7)	66 (6.4)	145 (14.1)	165 (10.9)	93 (6.1)	258 (17.0)	239 (7.9)	222 (7.3)	461 (15.2)	111 (7.9)	74 (5.3)	185 (13.2)	71 (8.6)	75 (9.1)	146 (17.8)	750 (8.3)	626 (7.0)	1376 (15.3)
40-49	124 (10.4)	82 (6.8)	206 (17.2)	107 (10.4)	57 (5.5)	164 (15.9)	169 (11.1)	76 (5.0)	245 (16.1)	238 (7.8)	191 (6.3)	429 (14.1)	152 (19.9)	76 (5.4)	228 (16.3)	95 (11.6)	43 (5.2)	138 (16.8)	885 (9.8)	525 (5.8)	1410 (15.7)
50-59	75 (6.3)	60 (5.0)	135 (11.3)	81 (7.9)	82 (8.0)	163 (15.8)	129 (8.5)	87 (5.7)	216 (14.2)	196 (6.5)	170 (5.6)	366 (12.1)	139 (9.9)	116 (8.3)	255 (18.2)	55 (6.7)	49 (6.0)	104 (12.7)	675 (7.5)	564 (6.3)	1239 (13.8)
60-69	82 (6.8)	65 (5.4)	147 (12.3)	75 (7.3)	63 (6.1)	138 (13.4)	178 (11.7)	70 (4.6)	248 (16.3)	194 (6.4)	154 (5.1)	348 (11.5)	127 (9.1)	95 (6.8)	222 (15.9)	56 (6.8)	44 (5.4)	100 (12.2)	712 (7.9)	491 (5.5)	1203 (13.4)
≥ 70	64 (5.3)	30 (2.5)	94 (7.8)	57 (5.6)	34 (3.3)	91 (8.8)	128 (8.4)	51 (3.3)	179 (11.8)	165 (5.4)	101 (3.4)	266 (8.8)	111 (7.9)	78 (5.5)	189 (13.5)	47 (5.7)	37 (4.5)	84 (10.2)	572 (6.3)	331 (3.7)	903 (10)
Total	662 (55.3)	536 (44.7)	1198 (100)	551 (53.5)	479 (46.5)	1030 (100)	965 (63.6)	553 (36.4)	1518 (100)	1620 (52.8)	1431 (47.2)	3033 (100)	806 (57.6)	594 (42.4)	1400 (100)	452 (55.1)	369 (44.9)	821 (100)	5038 (56.0)	3962 (44.0)	9000 (100)
